# Supplementary material for: Multicomponent Synthesis of Unsaturated γ-Lactam Derivatives. Applications as Antiproliferative Agents through the Bioisosterism Approach: Carbonyl vs. Phosphoryl Group
Source: Pharmaceuticals (Basel). 2022 Apr 22;15(5):511. doi: 10.3390/ph15050511 (PMC9144317; doi:10.3390/ph15050511)
Supplement: Supplementary file 1 [file pharmaceuticals-15-00511-s001.zip › pharmaceuticals-1663647-supplementary.pdf]

pharmaceuticals

MDPI

Multicomponent Syntheses of Unsaturated γ-Lactam Derivatives. Applications as Antiproliferative Agents through the Bioisosterism Approach: Carbonyl *vs* Phosphoryl Group.

Xabier del Corte[1], Adrián López-Francés[1], Ilia Villate-Beitia[2,3,4], Myriam Sainz-Ramos[2,3,4], Edorta Martínez de Marigorta[1], Francisco Palacios[1], Concepción Alonso[1], Jesús M. de los Santos[1], José Luis Pedraz[2,3,4,*], and Javier Vicario[1,*]

[1] *Department of Organic Chemistry I, Faculty of Pharmacy, University of the Basque Countrys, UPV/EHU Paseo de la Universidad 7, 01006 Vitoria-Gasteiz, Spain*

[2] *NanoBioCel Group, University of the Basque Country (UPV/EHU), 01006 Vitoria-Gasteiz, Spain*

[3] *Biomedical Research Networking Center in Bioengineering, Biomaterials and Nanomedicine (CIBER-BBN), Faculty of Pharmacy, University of the Basque Country (UPV/EHU), 01006 Vitoria-Gasteiz, Spain*

[4] Bioaraba, NanoBioCel Research Group, Faculty of Pharmacy, University of the Basque Country (UPV/EHU), 01006 Vitoria-Gasteiz, Spain

* Correspondence: *joseluis.pedraz@ehu.eus (J.L.P.); javier.vicario@ehu.eus (J.V.)*

Supporting Information

Table of contents

1. Experimental procedures and characterization data for compounds **4, 8, 9, 12** and **13.** **S2**

2. ^{1}H NMR, ^{13}C NMR, ^{31}P NMR and ^{19}F NMR spectra of compounds **4, 8, 9, 12** and **13.** **S12**

3. HPLC chromatograms of compounds **4, 8, 9, 12** and **13.** **S70**

4. Flow cytometric assays on A-549 cells after addition of compounds **4l** and **12a**. **S83**

5. Antiproliferative activity in RKO cell line **S86**

6. Calculation of Lipinski's rule of five and prediction of ADME properties.

1. Experimental procedures and characterization data for compounds 4, 8, 9, 12 and 13.

General procedure for the synthesis of 3-amino 3-pyrrolin-2-ones **4** and **8**.

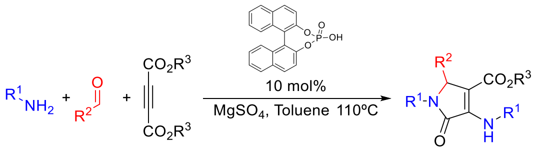

1 **2** **3** **4**

A solution of amine **1** (4 mmol), aldehyde **2** (2 mmol), acetylene dicarboxylate derivative **3** (2 mmol) and BINOL derived phosphoric acid (70 mg, 0.2 mmol) were stirred in the presence of anhydrous $MgSO_4$ in Toluene (10 mL) at 110°C for 24-72 hours. Then, the volatiles were distilled off at reduced pressure and the crude residue was purified by column chromatography (Hexanes /AcOEt) to afford pure lactams **4** and **8**.

*Ethyl 5-oxo-2-phenyl-1-(p-tolyl)-4-(p-tolylamino)-2,5-dihydro-1H-pyrrole-3-carboxylate (**4a**).* The general procedure was followed using *p*-toluidine (**1a**) (429 mg, 4 mmol), benzaldehyde (**2a**) (204 μL, 2 mmol) and diethyl acetylenedicarboxylate (**3a**) (320 μL, 2 mmol). The residue was purified by column chromatography (Hexanes /AcOEt 9:1) affording 656 mg (77%) of **4a** as a white solid. Physical and spectroscopic data are in agreement with literature data.[1]

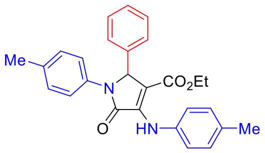

4a

*Ethyl 1-(4-methoxyphenyl)-4-((4-methoxyphenyl)amino)-5-oxo-2-phenyl-2,5-dihydro-1H-pyrrole-3-carboxylate (**4b**).* The general procedure was followed using *p*-anisidine (**1b**) (492 mg, 4 mmol), benzaldehyde (**2a**) (204 μL, 2 mmol) and diethyl acetylenedicarboxylate (**3a**) (320 μL, 2 mmol). The residue was purified by column chromatography (Hexanes /AcOEt 8:2) affording 695 mg (76%) of **4b** as a yellow solid. Physical and spectroscopic data are in agreement with literature data.[1]

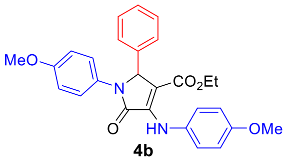

4b

*Ethyl 1-benzyl-4-(benzylamino)-5-oxo-2-phenyl-2,5-dihydro-1H-pyrrole-3-carboxylate (**4c**).* The general procedure was followed using benzylamine (**1c**) (437 μL, 4 mmol), benzaldehyde (**2a**) (204 μL, 2 mmol) and diethyl acetylenedicarboxylate (**3a**) (320 μL, 2 mmol). The residue was purified by column chromatography (Hexanes /AcOEt 9:1) affording 494 mg (58%) of **4c** as a white solid. Physical and spectroscopic data are in agreement with literature data.[1]

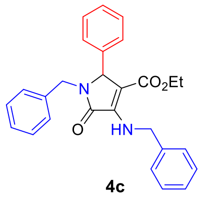

4c

*Isopropyl 5-oxo-2-phenyl-1-(p-tolyl)-4-(p-tolylamino)-2,5-dihydro-1H-pyrrole-3-carboxylate (**4d**).* The general procedure was followed using *p*-toluidine (**1a**) (429 mg, 4 mmol), benzaldehyde (**2a**) (204 μL, 2 mmol) and di-*iso*-propyl acetylenedicarboxylate (**3b**) (396 mg, 2 mmol). The residue was purified by column chromatography (Hexanes /AcOEt 9:1) affording 493 mg (56%) of **4d** as a white solid. M.p. (Et_2O) = 156-157 °C. ^1H NMR (400 MHz, $CDCl_3$) δ 8.26 (*s*, H, NH), 7.35 (*d*, $^3J_{HH}$ = 8.6 Hz, 2H), 7.24-7.21 (*m*, 3H), 7.20-7.17 (*m*, 2H), 7.11 (*d*, $^3J_{HH}$ = 8.5 Hz, 2H), 7.09 (*d*, $^3J_{HH}$ = 8.5 Hz, 2H), 7.02 (*d*, $^3J_{HH}$ = 8.6 Hz, 2H), 5.74

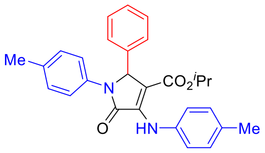

4d

---

[1] del Corte, X.; Martinez de Marigorta, E.; Palacios, F.; Vicario, J. A Brønsted Acid-Catalyzed Multicomponent Reaction for the Synthesis of Highly Functionalized γ-Lactam Derivatives. *Molecules* **2019**, *24*, 2951. DOI: 10.3390/molecules24162951.

(*s*, 1H), 4.90 (*hept*, $^3J_{HH}$ = 6.3 Hz, 1H), 2.33 (*s*, 3H), 2.22 (*s*, 3H), 1.18 (*d*, $^3J_{HH}$ = 6.3 Hz, 3H), 0.86 (d, $^3J_{HH}$ = 6.2 Hz, 3H) ppm. ^{13}C {^1H} NMR (101 MHz, $CDCl_3$): δ 164.4 (C=O), 164.0 (C=O), 143.0 (C_{quat}), 137.3 (C_{quat}), 136.1 (C_{quat}), 135.5 (C_{quat}), 134.5 (C_{quat}), 134.2 (C_{quat}), 129.5 (2xCH), 129.2 (2xCH), 128.4 (2xCH), 128.1 (CH), 128.0 (2xCH), 123.2 (2xCH), 122.9 (2xCH), 109.4 (C_{quat}), 67.8 (CH) 63.2 (CHN), 22.1 (CH_3), 21.5 (CH_3), 21.1 (CH_3), 21.0 (CH_3) ppm. FTIR (neat) ν_{max}: 3312 (N-H), 1701(C=O), 1679 (C=O), 1635 (C=C) cm^{-1}. HRMS (ESI-TOF) m/z calcd for $C_{28}H_{29}N_2O_3$ $[M+H]^+$ 441.5508, found 441.5506.

*Methyl 5-oxo-2-phenyl-1-(p-tolyl)-4-(p-tolylamino)-2,5-dihydro-1H-pyrrole-3-carboxylate (**4e**).* The general procedure was followed using *p*-toluidine (**1a**) (429 mg, 4 mmol), benzaldehyde (**2a**) (204 µL, 2 mmol) and dimethyl acetylenedicarboxylate (**3c**) (246 µL, 2 mmol). The residue was purified by column chromatography (Hexanes /AcOEt 9:1) affording 675 mg (82%) of **4e** as a white solid. M.p. (Et_2O) = 179-181 °C. ^1H NMR (400 MHz, $CDCl_3$) δ 8.14 (*s*, 1H), 7.33 (d, $^3J_{HH}$ = 8.5Hz, 2H), 7.25 (*m*, 4H), 7.20 (*m*, 1H), 7.13 (*d*, $^3J_{HH}$ = 8,3 Hz, 2H), 7.09 (*d*, $^3J_{HH}$ = 8,5 Hz, 2H), 7.03 (*d*, $^3J_{HH}$ = 8,3 Hz, 2H), 5.77 (*s*, 1H), 3.54 (*s*, 3H), 2.34 (*s*, 3H), 2.23 (*s*, 3H) ppm. ^{13}C {^1H} NMR (101 MHz, $CDCl_3$): δ 164.9 (C=O), 163.9 (C=O), 142.8 (C_{quat}), 137.2 (C_{quat}), 136.0 (C_{quat}), 135.5 (C_{quat}), 134.7 (C_{quat}), 134.1 (C_{quat}), 129.5 (2xCH), 129.1 (2xCH), 128.5 (2xCH), 128.1 (CH), 127.7 (2xCH), 123.3 (2xCH), 122.8 (2xCH), 108.9 (C_{quat}), 63.2 (CH), 51.2 (CH_3), 21.1 (CH_3), 21.0 (CH_3) ppm. FTIR (neat) ν_{max}: 3286 (N-H), 1705 (C=O), 1677 (C=O), 1630 (C=C) cm^{-1}. HRMS (ESI-TOF) m/z calcd for $C_{26}H_{25}N_2O_3$ $[M+H]^+$ 413.1865, found 413.1863.

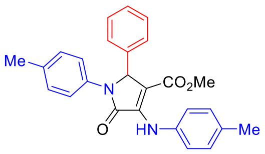

4e

*Methyl 5-oxo-1-(p-tolyl)-4-(p-tolylamino)-2-(4-(trifluoromethyl)phenyl)-2,5-dihydro-1H-pyrrole-3-carboxylatecarboxylate (**4f**).* The general procedure was followed using *p*-toluidine (**1a**) (429 mg, 4 mmol), *p*-(trifluoromethyl)benzaldehyde (**2b**) (273 µL, 2 mmol) and dimethyl acetylenedicarboxylate (**3c**) (246 µL, 2 mmol). The residue was purified by column chromatography (Hexanes /AcOEt 9:1) affording 701 mg (73%) of **4f** as a white solid. M.p. (Et_2O) = 207-208 °C. ^1H NMR (400 MHz, $CDCl_3$) 8.16 (*bs*, 1H), 7.50 (*d*, $^3J_{HH}$ = 7.9 Hz, 2H), 7.36 (*d*, $^3J_{HH}$ = 7.9 Hz, 2H), 7.31 (*d*, $^3J_{HH}$ = 8.5 Hz, 2H), 7.13 (*d*, $^3J_{HH}$ = 8.4 Hz, 2H), 7.08 (*d*, $^3J_{HH}$ = 8.4 Hz, 2H), 7.08 – 7.01 (*m*, 2H), 5.82 (*s*, 1H), 3.55 (*s*, 3H), 2.34 (*s*, 3H), 2.24 (*s*, 3H) ppm. ^{13}C {^1H} NMR (101 MHz, $CDCl_3$): δ 164.7 (C=O), 163.8 (C=O), 143.0 (C_{quat}), 141.5 (*d*, $^5J_{FC}$ = 1.6 Hz, C_{quat}), 135.8 (C_{quat}), 135.7 (C_{quat}), 135.0 (C_{quat}), 133.7 (C_{quat}), 130.2 (*q*, $^2J_{FC}$ = 32.4 Hz, C_{quat}), 129.6 (2xCH), 129.1 (2xCH), 128.0 (2xCH), 125.5 (*q*, $^3J_{FC}$ = 3.7 Hz, 2xCH), 124.0 (*q*, $^1J_{FC}$ = 273.4 Hz, CF_3), 123.5 (2xCH), 122.6 (2xCH), 107.3 (C_{quat}), 62.5 (CH), 51.2 (CH_3), 21.1 (CH_3), 21.0 (CH_3) ppm. ^{19}F NMR (282 MHz, $CDCl_3$) δ -63.0 ppm. FTIR (neat) ν_{max}: 3345 (N-H), 1704 (C=O), 1685 (C=O), 1631 (C=C), 1330 (C-F) cm^{-1}. HRMS (ESI-TOF) m/z calcd for $C_{27}H_{23}F_3N_2O_3$ $[M+H]^+$ 481.1739, found 481.1756.

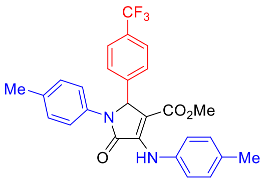

4f

*Methyl 1-benzyl-4-(benzylamino)-5-oxo-2-(4-(trifluoromethyl)phenyl)-2,5-dihydro-1H-pyrrole-3-carboxylate (**4g**).* The general procedure was followed using benzylamine (**1c**) (437 µL, 4 mmol), *p*-(trifluoromethyl)benzaldehyde (**2b**) (273 µL, 2 mmol) and dimethyl acetylenedicarboxylate (**3c**) (246 µL, 2 mmol). The residue was purified by column chromatography (Hexanes /AcOEt 9:1) affording 134 mg (17%) of **4g** as a white solid. M.p. (Et_2O) = 128-129 °C. ^1H NMR (400 MHz, DMSO-d_6, 60°C) δ 7.62 (*d*, $^3J_{HH}$ = 8.1 Hz, 2H), 7.40-7.34 (*m*, 5H), 7.29 (*d*, $^3J_{HH}$ = 8.1 Hz, 3H), 7.25 (*d*, $^3J_{HH}$ = 7.5 Hz, 2H), 7.05 (*dd*, $^3J_{HH}$ = 7.5, $^3J_{FH}$ = 1.8 Hz, 2H), 5.09 (*d*, $^3J_{HH}$ = 6.9 Hz, 2H), 5.08 (*s*, 1H), 4.80 (*d*, $^2J_{HH}$ = 15.2 Hz, 1H), 3.79 (*d*, $^2J_{HH}$ = 15.2 Hz, 1H), 3.44 (*s*, 1H) ppm. ^{13}C {^1H} NMR (101 MHz, DMSO-d_6, 60°C): δ 164.6 (C=O), 163.6 (C=O), 145.4 (C_{quat}), 142.0 (C_{quat}), 139.7 (C_{quat}), 136.0 (C_{quat}), 128.35 (*d*, $^2J_{FC}$ = 31.5 Hz, C_{quat}) 128.0 (4xCH), 127.9 (2xCH), 127.3 (2xCH), 126.8 (CH), 126.7 (2xCH), 126.4 (CH), 124.8 (*q*, $^3J_{FC}$ = 3.9 Hz, 2xCH), 123.7 (*q*, $^1J_{FC}$ = 272.1 Hz, CF_3), 102.5 (C_{quat}), 60.3 (CH), 49.9 (CH_3), 45.3 (CH_2), 43.8 (CH_2) ppm. ^{19}F

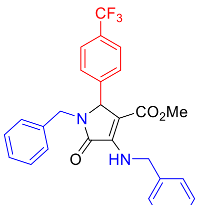

4g

NMR (282 MHz, $CDCl_3$) δ -63.0 ppm. FTIR (neat) ν_{max} : 3331 (N-H), 1698 (C=O), 1682 (C=O), 1625 (C=C), 1321 (C-F) cm^{-1}. HRMS (ESI-TOF) m/z calcd for $C_{27}H_{23}F_3N_2O_3$ $[M+H]^+$ 481.1739, found 481.1714.

*Methyl 2-(4-hydroxyphenyl)-5-oxo-1-(p-tolyl)-4-(p-tolylamino)-2,5-dihydro-1H-pyrrole-3-carboxylate (**4h**).* The general procedure was followed using *p*-toluidine (**1a**) (429 mg, 4 mmol), *p*-hydroxybenzaldehyde (**2c**) (244 mg, 2 mmol) and dimethyl acetylenedicarboxylate (**3c**) (246 μL, 2 mmol). The residue was purified by column chromatography (Hexanes /AcOEt 1:1) affording 205 mg (24%) of **4h** as a yellow solid. M.p. (MeOH) = 253 °C (dec.). ^{1}H NMR (400 MHz, $CDCl_3$) 8.08 (*bs*, 1H), 7.29 (*d*, $^3J_{HH}$ = 8.4 Hz, 2H), 7.13 – 7.01 (*m*, 8H), 6.67 (d, $^3J_{HH}$ = 8.4 Hz, 2H), 5.70 (s, 1H, CH), 4.77 (*s*, 1H), 3.54 (*s*, 3H), 2.33 (*s*, 3H), 2.23 (*s*, 3H) ppm. ^{13}C {^{1}H} NMR (101 MHz, $CDCl_3$): δ 165.0 (C=O), 163.9 (C=O), 155.3 (C_{quat}) , 142.6 (C_{quat}), 136.1 (C_{quat}), 135.6 (C_{quat}), 134.7 (C_{quat}),134.0 (C_{quat}), 129.5 (2xCH), 129.2 (C_{quat}), 129.2 (2xCH), 129.1 (2xCH), 123.3 (2xCH), 123.0 (2xCH), 115.50 (2xCH), 108.5 (C_{quat}), 62.8 (CH), 51.2 (CH_3), 21.1 (CH_3), 21.1 (CH_3) ppm. FTIR (neat) ν_{max} : 3407 (O-H), 3259 (N-H), 1698 (C=O), 1672 (C=O), 1653 (C=C), cm^{-1}. HRMS (ESI-TOF) m/z calcd for $C_{26}H_{25}N_2O_4$ $[M+H]^+$ 429.1806, found 429.1833.

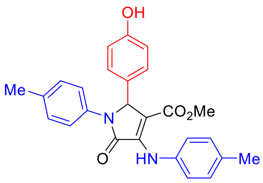

4h

*Methyl 1-benzyl-4-(benzylamino)-2-(4-hydroxyphenyl)-5-oxo-2,5-dihydro-1H-pyrrole-3-carboxylate (**4i**).* The general procedure was followed using benzylamine (**1c**) (437 μL, 4 mmol), *p*-hydroxybenzaldehyde (**2c**) (244 mg, 2 mmol) and dimethyl acetylenedicarboxylate (**3c**) (246 μL, 2 mmol). The residue was purified by column chromatography (Hexanes /AcOEt 1:1) affording 625 mg (73%) of **4i** as a white solid. M.p. (MeOH) = 216-217 °C. ^{1}H NMR (400 MHz, DMSO-d_6, 60°C) δ 9.13 (*s*, 1H), 7.35-7.32 (*m*, 4H), 7.31 – 7.21 (*m*, 4H), 7.06 (*d*, $^3J_{HH}$ = 6.5 Hz, 2H), 6.85 (*d*, $^3J_{HH}$ = 8.6 Hz, 2H), 6.71 (*d*, $^3J_{HH}$ = 8.6 Hz, 2H), 5.05 (*d*, $^3J_{HH}$ = 6.8 Hz, 2H), 4.83 (*bs*, 1H), 4.81 (*d*, $^2J_{HH}$ = 15.2 Hz, 1H), 3.61 (*d*, $^2J_{HH}$ = 15.2 Hz, 1H), 3.43 (*s*, 3H) ppm. ^{13}C {^{1}H} NMR (101 MHz, DMSO-d_6, 60°C): δ 164.4 (C=O), 164.0 (C=O), 156.8 (C_{quat}), 145.2 (C_{quat}), 139.8 (C_{quat}), 136.4 (C_{quat}), 128.1 (4xCH), 127.9 (2xCH), 127.2 (2xCH), 126.8 (CH), 126.8 (C_{quat}), 126.7 (2xCH), 126.4 (CH), 115.0 (2xCH), 103.4 (C_{quat}), 60.2 (CH), 49.9 (CH_3), 45.3 (CH_2), 43.2 (CH_2)) ppm. FTIR (neat) ν_{max} : 3430 (O-H), 3284 (N-H), 1672 (C=O), 1612 (C=C) cm^{-1}. HRMS (ESI-TOF) m/z calcd for $C_{26}H_{25}N_2O_4$ $[M+H]^+$ 429.1814, found 429.1833.

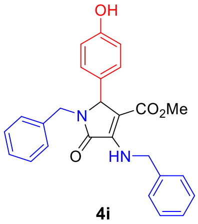

4i

*Methyl 2-(3-methoxyphenyl)-5-oxo-1-(p-tolyl)-4-(p-tolylamino)-2,5-dihydro-1H-pyrrole-3-carboxylate (**4j**).* The general procedure was followed using *p*-toluidine (**1a**) (429 mg, 4 mmol), *m*-anisaldehyde (**2d**) (243 μL, 2 mmol) and dimethyl acetylenedicarboxylate (**3c**) (246 μL, 2 mmol). The residue was purified by column chromatography (Hexanes /AcOEt 8:2) affording 688 mg (78%) of **4j** as a yellow solid. M.p. (Et_2O) = 171-172 °C. ^{1}H NMR (400 MHz, $CDCl_3$) δ 8.15 (*bs*, 1H, 7.33 (*d*, $^3J_{HH}$ = 8.5 Hz, 2H), 7.19 – 7.01 (*m*, 7H), 6.85 (*m*, 1H), 6.76 (*t*, $^3J_{HH}$ = 2.1 Hz, 1H), 6.72 (*m*, 1H), 5.73 (*s*, 1H), 3.73 (*s*, 3H), 3.56 (*s*, 3H), 2.33 (*s*, 3H), 2.23 (*s*, 3H) ppm. ^{13}C {^{1}H} NMR (101 MHz, $CDCl_3$): δ 164.8 (C=O), 164.0 (C=O), 159.6 (C_{quat}), 142.7 (C_{quat}), 138.7 (C_{quat}), 135.9 (C_{quat}), 135.4 (C_{quat}), 134.6 (C_{quat}), 134.0 (C_{quat}), 129.4 (2xCH), 129.3 (CH), 129.0 (2xCH), 123.2 (2xCH), 122.7 (2xCH), 120.1 (CH), 113.4 (CH), 113.2 (CH), 108.2 (C_{quat}), 63.0 (CH), 55.2 (CH_3) , 51.1 (CH_3), 21.0 (CH_3), 20.9 (CH_3) ppm. FTIR (neat) ν_{max} : 3382 (N-H), 1694 (C=O), 1625 (C=O), 1609 (C=C), cm^{-1}. HRMS (ESI-TOF) m/z calcd for $C_{27}H_{27}N_2O_4$ $[M+H]^+$ 443.1971, found 443.1981.

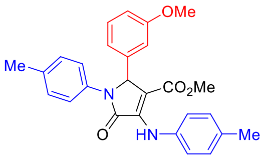

4j

*Methyl 1-benzyl-4-(benzylamino)-2-(3-methoxyphenyl)-5-oxo-2,5-dihydro-1H-pyrrole-3-carboxylate (**4k**).* The general procedure was followed using benzylamine (**1c**) (437 µL, 4 mmol), *m*-anisaldehyde (**2d**) (243 µL, 2 mmol) and dimethyl acetylenedicarboxylate (**3c**) (246 µL, 2 mmol). The residue was purified by column chromatography (Hexanes /AcOEt 9:1) affording 327 mg (37%) of **4k** as a white solid. M.p. (Et_2O) = 111-112 °C. ^{1}H NMR (400 MHz, DMSO-*d*$_6$, 60°C) δ 7.38 – 7.34 (*m*, 4H), 7.36 – 7.19 (*m*, 6H), 7.10 – 7.06 (*m*, 2H), 6.86 (*ddd*, $^3J_{HH}$ = 8.2 Hz, $^4J_{HH}$ = 2.5, 0.9 Hz, 1H), 6.66 (*dt*, $^3J_{HH}$ = 7.7, $^4J_{HH}$ = 1.2 Hz, 1H), 6.60 (*t*, $^4J_{HH}$ = 2.0 Hz, 1H), 5.11 (*dd*, $^2J_{HH}$ = 15.0, $^3J_{HH}$ = 6.8 Hz, 1H), 5.07 (*dd*, $^2J_{HH}$ = 15.0, $^3J_{HH}$ = 6.8 Hz, 1H), 4.94 (*s*, 1H), 4.83 (*d*, $^2J_{HH}$ = 15.1 Hz, 1H), 3.72 (*s*, 3H), 3.69 (*d*, $^2J_{HH}$ = 15.1 Hz, 1H), 3.45 (*s*, 3H) ppm. ^{13}C {^{1}H} NMR (101 MHz, DMSO-d$_6$, 60°C): δ (101 MHz, DMSO-*d*$_6$, 60°C) δ 164.5 (C=O), 163.9 (C=O), 159.1 (C_{quat}), 145.3 (C_{quat}), 139.8 (C_{quat}), 138.6 (C_{quat}), 136.2 (C_{quat}), 129.1 (CH), 128.0 (2xCH), 127.9 (2xCH), 127.3 (2xCH), 126.8 (CH), 126.7 (2xCH), 126.4 (CH), 119.2 (CH), 113.2 (CH), 112.9 (CH), 103.0 (C_{quat}), 60.7 (CH), 54.7 (CH_3), 49.9 (CH_3), 45.2 (CH_2), 43.5 (CH_2) ppm. FTIR (neat) ν_{max} : 3338 (N-H), 1701 (C=O), 1672 (C=O), 1622 (C=C) cm^{-1}. HRMS (ESI-TOF) m/z calcd for $C_{27}H_{27}N_2O_4$ [M+H]$^+$ 443.1971, found 443,1942.

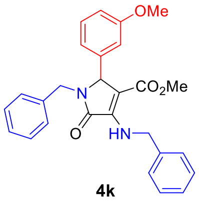

4k

*Methyl 2-(4-hydroxy-3-methoxyphenyl)-5-oxo-1-(p-tolyl)-4-(p-tolylamino)-2,5-dihydro-1H-pyrrole-3-carboxylate (**4l**).* The general procedure was followed using *p*-toluidine **(1a)** (429 mg, 4 mmol), vanilin **(2e)** (304 mg, 2 mmol) and dimethyl acetylenedicarboxylate **(3c)** (246 µL, 2 mmol). The residue was purified by column chromatography (Hexanes /AcOEt 1:1) affording 394 mg (43%) of **4l** as a yellow solid. M.p. (Et_2O) = 160-162 °C. ^{1}H NMR (400 MHz, $CDCl_3$) δ 8.14 (*bs*, 1H), 7.32 (*d*, $^3J_{HH}$ = 8.4 Hz, 2H), 7.12 (*d*, $^3J_{HH}$ = 8.5 Hz, 2H), 7.08 (*d*, $^3J_{HH}$ = 8.5 Hz, 2H), 7.05 (*d*, $^3J_{HH}$ = 8.4 Hz, 2H), 6.81 (*dd*, $^3J_{HH}$ = 8.2 Hz, $^4J_{HH}$ = 1.9 Hz, 1H), 6.77 (*d*, $^3J_{HH}$ = 8.2 Hz, 1H), 6.62 (*d*, $^4J_{HH}$ = 1.9 Hz, 1H), 5.71 (*s*, 1H, CH), 5.61 (*s*, 1H), 3.77 (*s*, 3H), 3.56 (*s*, 3H), 2.34 (*s*, 3H), 2.24 (*s*, 3H) ppm. ^{13}C {^{1}H} NMR (101 MHz, $CDCl_3$): δ 165.0 (C=O), 163.9 (C=O), 146.6 (C_{quat}), 145.4 (C_{quat}), 142.5 (C_{quat}), 136.0 (C_{quat}), 135.6 (C_{quat}), 134.6 (C_{quat}), 134.0 (C_{quat}), 129.5 (2xCH), 129.1 (2xCH), 128.6 (C_{quat}), 123.1 (2xCH), 123.0 (2xCH), 121.5 (CH), 114.2 (CH), 109.3 (CH), 108.5 (C_{quat}), 63.2 (CH), 56.0 (CH_3), 51.2 (CH_3), 21.1 (CH_3), 21.0 (CH_3) ppm. FTIR (neat) ν_{max} : 3404 (O-H), 3281 (N-H), 1694 (C=O), 1637 (C=O), 1609 (C=C), cm^{-1}. HRMS (ESI-TOF) m/z calcd for $C_{27}H_{27}N_2O_5$ [M+H]$^+$ 459.1920, found 459.1930.

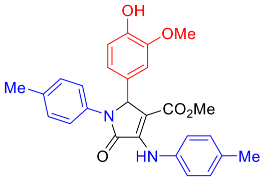

4l

*Methyl 1-benzyl-4-(benzylamino)-2-(4-hydroxy-3-methoxyphenyl)-5-oxo-2,5-dihydro-1H-pyrrole-3-carboxylate (**4m**).* The general procedure was followed using benzylamine (**1c**) (437 µL, 4 mmol), vanilin (**2e**) (304 mg, 2 mmol) and dimethyl acetylenedicarboxylate (**3c**) (246 µL, 2 mmol). The residue was purified by column chromatography (Hexanes /AcOEt 1:1) affording 513 mg (56%) of **4m** as a white solid. M.p. (Et_2O) = 154-156 °C. ^{1}H NMR (400 MHz, DMSO-*d*$_6$, 60°C) δ 8.64 (*s*, 1H), 7.40 – 7.19 (*m*, 10H), 7.09 (*d*, $^3J_{HH}$ = 7.5 Hz, 1H), 6.76 (*d*, $^3J_{HH}$ = 7.5 Hz, 1H), 6.54 (*s*, 1H), 6.52 (*bs*, 1H), 5.09 (*m*, 2H), 4.88 (*s*, 1H), 4.83 (*d*, $^2J_{HH}$ = 15.1 Hz, 1H), 3.74 (*d*, $^2J_{HH}$ = 15.1 Hz, 1H), 3.69 (*s*, 3H), 3.46 (*s*, 3H) ppm. ^{13}C {^{1}H} NMR (101 MHz, DMSO-*d*$_6$, 60°C) δ 164.3 (C_{quat} , 163.9 (C_{quat}), 147.2 (C_{quat}), 146.2 (C_{quat}), 145.1 (C_{quat}), 139.8 (C_{quat}), 136.3 (C_{quat}), 127.9 (2xCH), 127.8 (2xCH), 127.4 (C_{quat}), 127.2 (2xCH), 126.6 (CH), 126.5 (2xCH), 126.3 (CH), 119.9 (CH), 115.2 (CH), 111.5 (CH), 103.2 (C_{quat}), 60.5 (CH), 55.5 (CH_3), 49.7 (CH_3), 45.1 (CH_2), 43.2 (CH_2) ppm. FTIR (neat) ν_{max} : 3512 (O-H), 3347 (N-H), 1688 (C=O), 1672 (C=O), 1619 (C=C) cm^{-1}. HRMS (ESI-TOF) m/z calcd for $C_{27}H_{26}N_2O_5$ [M+H]$^+$ 459.1920, found 459.1928.

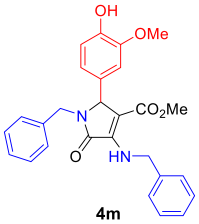

4m

*5-oxo-2-phenyl-N,1-di-p-tolyl-4-(p-tolylamino)-2,5-dihydro-1H-pyrrole-3-carboxamide (**8a**).* The general procedure was followed using *p*-toluidine (**1a**) (429 mg, 4 mmol), benzaldehyde (**2a**) (204 µL, 2 mmol) and di-*iso*-propyl acetylenedicarboxylate (**3b**) (396 mg, 2 mmol). The residue was purified by column chromatography (Hexanes /AcOEt 8:2) affording 214 mg (22%) of **8a** as a white solid. Physical and spectroscopic data are in agreement with literature data.[1]

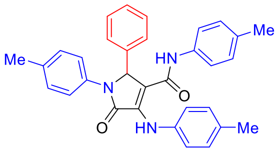

8a

*N,1-bis(4-methoxyphenyl)-4-((4-methoxyphenyl)amino)-5-oxo-2-phenyl-2,5-dihydro-1H-pyrrole-3-carboxamide (**8b**).* The general procedure was followed using *p*-anisidine (**1b**) (492 mg, 4 mmol), benzaldehyde (**2a**) (204 µL, 2 mmol) and di-*iso*-propyl acetylenedicarboxylate (**3b**) (396 mg, 2 mmol). The residue was purified by column chromatography (Hexanes /AcOEt 7:3) affording 139 mg (13%) of **8b** as a white solid. Physical and spectroscopic data are in agreement with literature data.[1]

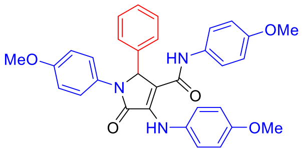

8b

Procedure for the synthesis of *ethyl 4-amino-1-benzyl-5-oxo-2-phenyl-2,5-dihydro-1H-pyrrole-3-carboxylate* **9**.

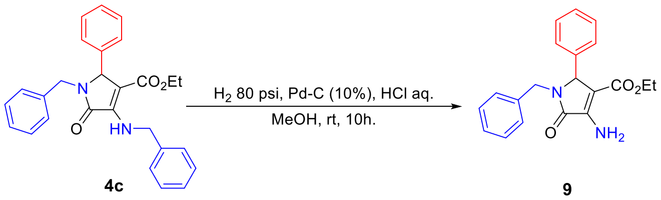

A mixture of **4c** (21.3 mg, 0.5 mmol), 10% palladium on carbon (276 mg, 0.025 mmol), and 37% HCl (0.05 mL, 0.5 mmol) was stirred for 10 hours in methanol (30 mL) under hydrogen pressure (80 psi). The reaction mixture was filtered through Celite, and the filtered solution was treated with $NaHCO_3$ until neutral and extracted with dichloromethane (3x15 mL). The combined organic fractions were dried with anhydrous $MgSO_4$, and distilled off at reduced pressure. The residue was crystallized in Et_2O/pentane (1:2) to afford 163 mg (99%) of **9** as a white solid. Physical and spectroscopic data are in agreement with literature data.[1]

General procedure for the synthesis 3-hidroxy 3-pyrrolin-2-ones **12**.

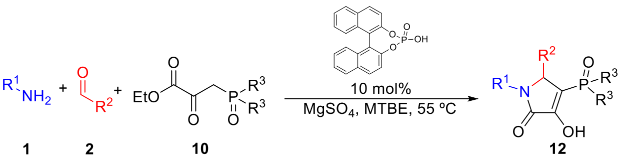

1 **2** **10** **12**

A solution of amine **1** (4 mmol), aldehyde **2** (2 mmol), ethyl pyruvate derivative **10** (6 mmol), BINOL derived phosphoric acid (70 mg, 0.2 mmol) and anhydrous $MgSO_4$ were stirred in MTBE (10 mL) at 55 °C for 48 hours. The volatiles were distilled off at reduced pressure and the crude residue was purified by column chromatography (Hexanes /AcOEt) to afford pure compounds **12**.

*Diethyl (4-hydroxy-5-oxo-2-phenyl-1-(p-tolyl)-2,5-dihydro-1H-pyrrol-3-yl)phosphonate (**12a**).* The general procedure was followed using *p*-toluidine (**1a**) (429 mg, 4 mmol), benzaldehyde (**2a**) (204 μL, 2 mmol) and ethyl 3-(diethoxyphosphoryl)-2-oxopropanoate (**10a**) (1.513 g, 6 mmol). The residue was purified by column chromatography (Hexanes /AcOEt 3:7) affording 770 mg (96%) of **12a** as a white solid. Physical and spectroscopic data are in agreement with literature data.[2]

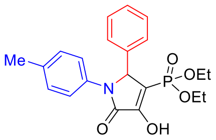

12a

*Diethyl (4-hydroxy-1-(4-methoxyphenyl)-5-oxo-2-phenyl-2,5-dihydro-1H-pyrrol-3-yl)phosphonate (**12b**).* The general procedure was followed using *p*-anisidine (**1b**) (492 mg, 4 mmol), benzaldehyde (**2a**) (204 μL, 2 mmol) and ethyl 3-(diethoxyphosphoryl)-2-oxopropanoate (**10a**) (1.513 g, 6 mmol). The residue was purified by column chromatography (Hexanes /AcOEt 3:7) affording 751 mg (90%) of **12b** as a yellow solid. Physical and spectroscopic data are in agreement with literature data.[2]

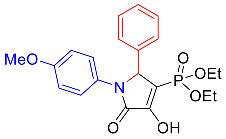

12b

*Diethyl (1-(2-fluorophenyl)-4-hydroxy-5-oxo-2-phenyl-2,5-dihydro-1H-pyrrol-3-yl)phosphonate (**12c**).* The general procedure was followed using *o*-fluoroaniline (**1d**) (386 μL, 4 mmol), benzaldehyde (**2a**) (204 μL, 2 mmol) and ethyl 3-(diethoxyphosphoryl)-2-oxopropanoate (**10a**) (1.513 g, 6 mmol). The residue was purified by column chromatography (Hexanes /AcOEt 3:7) affording 687 mg (85%) of **12c** as a light orange solid. Physical and spectroscopic data are in agreement with literature data.[2]

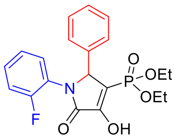

12c

*Diethyl (4-hydroxy-2-(4-nitrophenyl)-5-oxo-1-(p-tolyl)-2,5-dihydro-1H-pyrrol-3-yl)phosphonate (**12d**).* The general procedure was followed using *p*-toluidine (**1a**) (429 mg, 4 mmol), *p*-nitrobenzaldehyde (**2f**) (302 mg, 2 mmol) and ethyl 3-(diethoxyphosphoryl)-2-oxopropanoate (**10a**) (1.513 g, 6 mmol). The residue was purified by column chromatography (Hexanes /AcOEt 3:7) affording 829 mg (93%) of **12d** as a light orange solid. Physical and spectroscopic data are in agreement with literature data.[2]

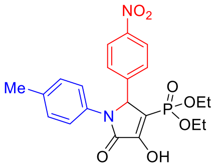

12d

*Diethyl (2-(4-fluorophenyl)-4-hydroxy-5-oxo-1-(p-tolyl)-2,5-dihydro-1H-pyrrol-3-yl)phosphonate (**12e**).* The general procedure was followed using *p*-toluidine (**1a**) (429 mg, 4 mmol), *p*-fluorobenzaldehyde (**2g**) (214 μL, 2 mmol) and ethyl 3-(diethoxyphosphoryl)-2-oxopropanoate (**10a**) (1.513 g, 6 mmol). The residue was purified by column chromatography (Hexanes /AcOEt 3:7) affording 604 mg (72%) of **12e** as a light yellow solid. Physical and spectroscopic data are in agreement with literature data.[2]

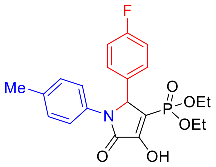

12e

*Diethyl (4-hydroxy-5-oxo-2-(thiophen-2-yl)-1-(p-tolyl)-2,5-dihydro-1H-pyrrol-3-yl)phosphonate (**12f**).* The general procedure was followed using *p*-toluidine (**1a**) (429 mg, 4 mmol), 2-thiophenecarboxaldehyde (**2h**) (187 μL, 2 mmol) and ethyl 3-(diethoxyphosphoryl)-2-oxopropanoate (**10a**) (1.513 g, 6 mmol). The residue was purified by column chromatography (Hexanes /AcOEt 3:7) affording 724 mg (89%) of **12f** as a white solid. Physical and spectroscopic data are in agreement with literature data.[2]

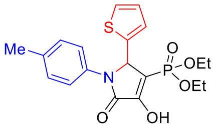

12f

[2] del Corte, X.; López-Francés, A.; Maestro, A.; Martinez de Marigorta, E.; Palacios, F.; Vicario. J. Brönsted Acid Catalyzed Multicomponent Synthesis of Phosphorated and Fluorinated γ-Lactam Derivatives. *J. Org. Chem.* **2020**, *85*, 14369-14383. DOI: 10.1021/acs.joc.0c00280.

*Ethyl 3-(diethoxyphosphoryl)-4-hydroxy-5-oxo-1-(p-tolyl)-2,5-dihydro-1H-pyrrole-2-carboxylate (**12g**).*

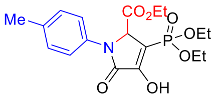

12g

The general procedure was followed using *p*-toluidine (**1a**) (429 mg, 4 mmol), 50% ethyl glyoxalate (**2i**) solution in toluene (396 µL, 2 mmol) and ethyl 3-(diethoxyphosphoryl)-2-oxopropanoate (**10a**) (1.513 g, 6 mmol). The residue was purified by column chromatography (Hexanes /AcOEt 2:8) affording 682 mg (86%) of **12g** as a light orane solid. Physical and spectroscopic data are in agreement with literature data.[2]

*Diethyl (4-hydroxy-2-isopropyl-5-oxo-1-(p-tolyl)-2,5-dihydro-1H-pyrrol-3-yl)phosphonate (**12h**).* The general procedure was followed using *p*-toluidine (**1a**) (429 mg, 4 mmol), isobutyraldehyde (**2j**) (182 µL, 2 mmol) and ethyl 3-(diethoxyphosphoryl)-2-oxopropanoate (**10a**) (1.513 g, 6 mmol). The residue was purified by column chromatography (Hexanes /AcOEt 2:8) affording 550 mg (75%) of **12h** as a white solid. Physical and spectroscopic data are in agreement with literature data.[2]

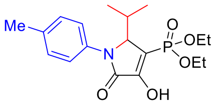

12h

*Diisopropyl (4-hydroxy-5-oxo-2-phenyl-1-(p-tolyl)-2,5-dihydro-1H-pyrrol-3-yl)phosphonate (**12i**).* The general procedure was followed using *p*-toluidine (**1a**) (429 mg, 4 mmol), benzaldehyde (**2a**) (204 µL, 2 mmol) and ethyl 3-(diisopropoxyphosphoryl)-2-oxopropanoate (**10b**) (1.681 g, 6 mmol). The residue was purified by column chromatography (Hexanes /AcOEt 3:7) affording 788 mg (92%) of **12i** as a white solid. Physical and spectroscopic data are in agreement with literature data.[2]

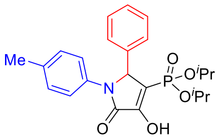

12i

*Diisopropyl (2-(4-fluorophenyl)-4-hydroxy-5-oxo-1-(p-tolyl)-2,5-dihydro-1H-pyrrol-3-yl)phosphonatephosphonate (**12j**).* The general procedure was followed using *p*-toluidine (**1a**) (429 mg, 4 mmol), *p*-fluorobenzaldehyde (**2g**) (214 µL, 2 mmol) and ethyl 3-(diisopropoxyphosphoryl)-2-oxopropanoate (**10b**) (1.681 g, 6 mmol). The residue was purified by column chromatography (Hexanes /AcOEt 3:7) affording 670 mg (75%) of **12j** as a white solid. Physical and spectroscopic data are in agreement with literature data.[2]

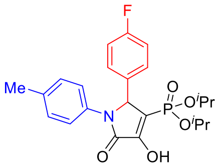

12j

*Ethyl 3-(diisopropoxyphosphoryl)-4-hydroxy-5-oxo-1-(p-tolyl)-2,5-dihydro-1H-pyrrole-2-carboxylate (**12k**).* The general procedure was followed using *p*-toluidine (**1a**) (429 mg, 4 mmol), 50% ethyl glyoxalate (**2i**) solution in toluene (396 µL, 2 mmol) and ethyl 3-(diisopropoxyphosphoryl)-2-oxopropanoate (**10b**) (1.681 g, 6 mmol). The residue was purified by column chromatography (Hexanes /AcOEt 2:8) affording 654 mg (77%) of **12k** as a white solid. Physical and spectroscopic data are in agreement with literature data.[2]

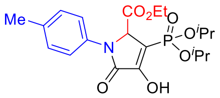

12k

*4-(diphenylphosphoryl)-3-hydroxy-5-phenyl-1-(p-tolyl)-1,5-dihydro-2H-pyrrol-2-onephosphonate (**12l**).* The general procedure was followed using *p*-toluidine (**1a**) (429 mg, 4 mmol), benzaldehyde (**2a**) (204 µL, 2 mmol) and ethyl 3-(diphenylphosphoryl)-2-oxopropanoate (**10c**) (1.897 g, 6 mmol). The residue was purified by column chromatography (Hexanes /AcOEt 3:7) affording 642 mg (69%) of **12i** as light yellow solid. Physical and spectroscopic data are in agreement with literature data.[2]

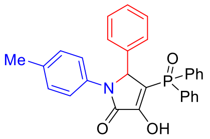

12l

4-(diphenylphosphoryl)-3-hydroxy-1-(4-methoxyphenyl)-5-phenyl-1,5-dihydro-2H-pyrrol-2-one (***12m***). The general procedure was followed using *p*-anisidine (**1b**) (492 mg, 4 mmol), benzaldehyde (**2a**) (204 µL, 2 mmol) and ethyl 3-(diphenylphosphoryl)-2-oxopropanoate (**10c**) (1.897 g, 6 mmol). The residue was purified by column chromatography (Hexanes /AcOEt 3:7) affording 770 mg (80%) of **12m** as white solid. Physical and spectroscopic data are in agreement with literature data.[2]

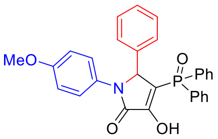

12m

4-(diphenylphosphoryl)-1-(2-fluorophenyl)-3-hydroxy-5-phenyl-1,5-dihydro-2H-pyrrol-2-one (***12n***). The general procedure was followed using *o*-fluoroaniline (**1d**) (386 µL, 4 mmol), benzaldehyde (**2a**) (204 µL, 2 mmol) and ethyl 3-(diphenylphosphoryl)-2-oxopropanoate (**10c**) (1.897 g, 6 mmol). The residue was purified by column chromatography (Hexanes /AcOEt 3:7) affording 722 mg (77%) of **12n** as light yellow solid. Physical and spectroscopic data are in agreement with literature data.[2]

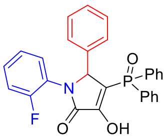

12n

4-(diphenylphosphoryl)-5-(4-fluorophenyl)-3-hydroxy-1-(p-tolyl)-1,5-dihydro-2H-pyrrol-2-one (***12o***). The general procedure was followed using *p*-toluidine (**1a**) (429 mg, 4 mmol), *p*-fluorobenzaldehyde (**2g**) (214 µL, 2 mmol) and ethyl 3-(diphenylphosphoryl)-2-oxopropanoate (**10c**) (1.897 g, 6 mmol). The residue was purified by column chromatography (Hexanes /AcOEt 3:7) affording 830 mg (87%) of **12o** as white solid. Physical and spectroscopic data are in agreement with literature data.[2]

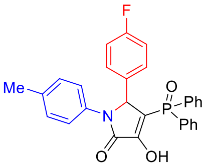

12o

4-(diphenylphosphoryl)-3-hydroxy-5-(perfluorophenyl)-1-(p-tolyl)-1,5-dihydro-2H-pyrrol-2-one (***12p***). The general procedure was followed using *p*-toluidine (**1a**) (429 mg, 4 mmol), 2,3,4,5,6-pentafluorobenzaldehyde (**2k**) (247 µL, 2 mmol) and ethyl 3-(diphenylphosphoryl)-2-oxopropanoate (**10c**) (1.897 g, 6 mmol). The residue was purified by column chromatography (Hexanes /AcOEt 3:7) affording 920 mg (83%) of **12p** as white solid. Physical and spectroscopic data are in agreement with literature data.[2]

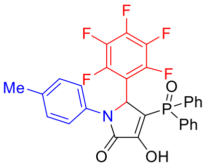

12o

General procedure for hydrolysis of 3-amino 3-pyrrolin-2-ones **4**. Synthesis of 3-hidroxy 3-pyrrolin-2-ones **13**.

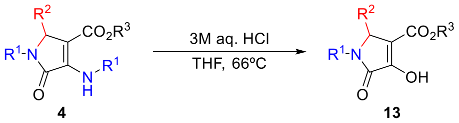

To 10 mL solution of a 3M HCl/THF (1:1), compound **4** (0.5 mmol) was added, and the mixture was heated to 66°C and stirred overnight. The reaction was monitored by TLC and, once it finished, the mixture was concentrated under reduced pressure to eliminate the THF, washed with 3M NaOH (2x5 mL) and H_2O (2x5mL), and extracted with ethyl acetate. The combined organic phases were dried with anhydrous $MgSO_4$, and the crude residue was crystalized in Et_2O: pentane.

Ethyl 4-hydroxy-5-oxo-2-phenyl-1-(p-tolyl)-2,5-dihydro-1H-pyrrole-3-carboxylate (***13a***). The general procedure was followed using γ-lactam **4a**, affording 160 mg (95%) of **13a** as a white solid. Physical and spectroscopic data are in agreement with literature data.[1]

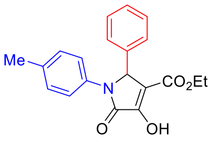

13a

*Ethyl 4-hydroxy-1-(4-methoxyphenyl)-5-oxo-2-phenyl-2,5-dihydro-1H-pyrrole-3-carboxylate (**13b**).* The general procedure was followed using γ-lactam **4b**, affording 143 mg (81%) of **13b** as a white solid. Physical and spectroscopic data are in agreement with literature data.[1]

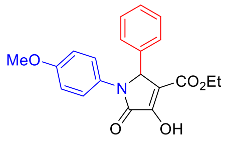

13b

*Ethyl 1-benzyl-4-hydroxy-5-oxo-2-phenyl-2,5-dihydro-1H-pyrrole-3-carboxylate (**13c**).* The general procedure was followed using γ-lactam **4c**, affording 157 mg (94%) of **13c** as a white solid. Physical and spectroscopic data are in agreement with literature data.[1]

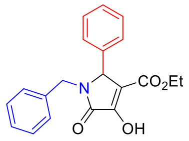

13c

*Methyl 1-benzyl-4-hydroxy-5-oxo-2-(4-(trifluoromethyl)phenyl)-2,5-dihydro-1H-pyrrole-3-carboxylate (**13d**).* The general procedure was followed using γ-lactam **4g**, affording 174 mg (89%) of **13d** as a white solid. M.p. (Et_2O) = 207-208 °C. ^1H NMR (400 MHz, $CDCl_3$) δ 9.07 (*bs*, 1H), 7.62 (*d*, $^3J_{HH}$ = 8.6 Hz, 2H), 7.34 – 7.27 (*m*, 3H), 7.23 (*d*, $^3J_{HH}$ = 7.4 Hz, 2H), 7.13 – 7.03 (*m*, 2H), 5.19 (*d*, $^2J_{HH}$ = 15.0 Hz, 1H), 4.94 (*s*, 1H), 3.64 (*s*, 3H), 3.56 (*d*, $^2J_{HH}$ = 15.0 Hz, 1H) ppm. ^{13}C {^1H} NMR (101 MHz, $CDCl_3$) δ 165.3 (C=O), 163.6 (C=O), 157.6 (C_{quat}), 139.0 (C_{quat}), 135.9 (C_{quat}), 131.3 (q, $^2J_{FH}$ = 31.5 Hz, C_{quat}), 129.1 (2xCH_{Ar}), 128.6 (2xCH), 128.4 (2xCH), 128.2 (CH), 126.1 (q, $^3J_{FH}$ = 3.9 Hz, 2xCH), 123.9 (d, $^1J_{FH}$ = 272.4 Hz, CF_3), 112.6 (C_{quat}), 59.3 (CH), 52.2 (CH_3), 44.4 (CH_2) ppm. ^{19}F NMR (282 MHz, $CDCl_3$) δ -63.1 ppm. FTIR (neat) ν_{max} : 3407 (O-H), 1685 (C=O), 1672 (C=O), 1641 (C=C), 1326 (C-F) cm^{-1}. HRMS (ESI-TOF) m/z calcd for $C_{20}H_{17}F_3NO_4$ $[M+H]^+$ 392.1101, found 392.1119.

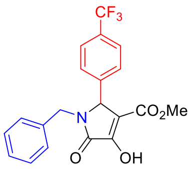

13d

*Methyl 4-hydroxy-2-(3-methoxyphenyl)-5-oxo-1-(p-tolyl)-2,5-dihydro-1H-pyrrole-3-carboxylate (**13e**).* The general procedure was followed using γ-lactam **4j**, affording 161 mg (91%) of **13e** as a white solid. M.p. (Et_2O) = 170 °C (dec.). ^1H NMR (400 MHz, $CDCl_3$) δ 8.93 (*bs*, 1H), 7.32 (*d*, $^3J_{HH}$ = 8.5 Hz, 2H), 7.17 (*t*, $^3J_{HH}$ = 7.9 Hz, 1H), 7.07 (*d*, $^3J_{HH}$ = 8.5 Hz, 2H), 6.83 (*m*, 1H), 6.75 (*ddd*, $^3J_{HH}$ = 8.2 Hz, $^4J_{HH}$ = 2.6, 0.9 Hz, 1H), 6.71 (*m*, 1H), 5.66 (*s*, 1H), 3.75 (*s*, 3H), 3.72 (*s*, 3H), 2.25 (*s*, 3H) ppm. ^{13}C {^1H} NMR (101 MHz, $CDCl_3$) δ 165.5 (C=O), 162.8 (C=O), 159.4 (C_{quat}), 156.2 (C_{quat}), 136.8 (C_{quat}), 136.0 (C_{quat}), 133.7 (C_{quat}), 129.8 (CH), 129.7 (2xCH), 122.6 (2xCH), 120.2 (CH), 113.9 (CH), 113.2 (CH), 112.7 (C_{quat}), 61.8 (CH), 55.4 (CH_3), 52.2 (CH_3), 21.1 (CH_3) ppm. FTIR (neat) ν_{max} : 3453 (O-H), 1685 (C=O), 1674 (C=O), 1640 (C=C) cm^{-1}. HRMS (ESI-TOF) m/z calcd for $C_{20}H_{20}NO_5$ $[M+H]^+$ 354.1333, found 354.1348.

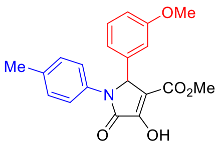

13e

*Methyl 1-benzyl-4-hydroxy-2-(3-methoxyphenyl)-5-oxo-2,5-dihydro-1H-pyrrole-3-carboxylate (**13f**).* The general procedure was followed using γ-lactam **4k**, affording 154 mg (87%) of **13f** as a white solid. M.p. (Et_2O) = 189-190 °C. ^1H NMR (400 MHz, $CDCl_3$) δ 9.09 (*bs*, 1H), 7.36 – 7.27 (*m*, 4H), 7.12 (*d*, $^3J_{HH}$ = 7.8, 1.8 Hz, 1H), 7.11 (*d*, $^3J_{HH}$ = 6.9 Hz, 1H), 6.89 (*ddd*, $^3J_{HH}$ = 8.2 Hz, $^4J_{HH}$ = 2.6 Hz, 1H), 6.72 (*dt*, $^3J_{HH}$ = 7.6, $^4J_{HH}$ = 1.2 Hz, 1H), 6.60 (*t*, $^4J_{HH}$ = 2.1 Hz, 1H), 5.18 (*d*, $^2J_{HH}$ = 14.8 Hz, 1H), 4.86 (*s*, 1H), 3.78 (*s*, 3H), 3.64 (*s*, 3H), 3.57 (*d*, $^2J_{HH}$ = 14.8 Hz, 1H) ppm. ^{13}C {^1H} NMR (101 MHz, $CDCl_3$) δ 165.7 (C=O), 163.5(C=O), 160.1 (C_{quat}), 157.6 (C_{quat}), 136.4 (C_{quat}), 136.1

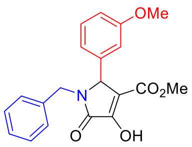

13f

(C$_{quat}$), 130.1 (CH), 129.0 (CH), 128.7 (2xCH), 128.0 (2xCH), 120.4 (CH), 114.4 (CH), 113.2 (CH), 112.9 (C$_{quat}$), 59.8 (CH), 55.5 (CH_3), 52.0 (CH_3), 44.2 (CH_2) ppm.ppm. FTIR (neat) ν_{max}: 3226 (O-H), 1688 (C=O), 1669 (C=O), 1640 (C=C) cm^{-1}. HRMS (ESI-TOF) m/z calcd for $C_{20}H_{20}NO_5$ [M+H]$^+$ 354.1333, found 354.1347.

*Methyl 4-hydroxy-2-(4-hydroxy-3-methoxyphenyl)-5-oxo-1-(p-tolyl)-2,5-dihydro-1H-pyrrole-3-carboxylate (**13g**)*. The general procedure was followed using γ-lactam **4l**, affording 150 mg (81%) of **13g** as a yellow solid. M.p. (Et_2O) = 158 °C (dec.).

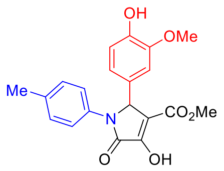

13g

^1H NMR (400 MHz, $CDCl_3$) δ 9.04 (*bs*, 1H), 7.29 (*d*, $^3J_{HH}$ = 8.1 Hz, 2H), 7.07 (*d*, $^3J_{HH}$ = 8.1 Hz, 2H), 6.79 (*bs*, 2H), 6.54 (*s*, 1H), 5.63 (*s*, 1H), 5.58 (*bs*, 1H), 3.78 (*s*, 3H), 3.75 (*s*, 3H), 2.26 (*s*, 3H) ppm. ^{13}C {^1H} NMR (101 MHz, $CDCl_3$) δ 165.6 (C=O), 162.8 (C=O), 156.4 (C$_{quat}$), 146.9 (C$_{quat}$), 145.9 (C$_{quat}$), 136.1 (C$_{quat}$), 133.7 (C$_{quat}$), 129.7 (2xCH$_{Ar}$), 126.7 (C$_{quat}$), 122.8 (2xCH), 121.8 (CH), 114.4 (CH), 112.8 (C$_{quat}$), 108.8 (CH), 61.9 (CH), 56.1 (CH_3), 52.2 (CH_3), 21.1 (CH_3) ppm. FTIR (neat) ν_{max}: 3420 (2xO-H), 1656 (C=O), 1637 (C=O), 1625 (C=C) cm^{-1}. HRMS (ESI-TOF) m/z calcd for $C_{20}H_{20}NO_6$ [M+H]$^+$ 370.1282, found 370.1292.

2. ^{1}H NMR, ^{13}C NMR, ^{31}P NMR and ^{19}F NMR spectra of compounds 4, 8, 9, 12 and 13.

*Ethyl 5-oxo-2-phenyl-1-(p-tolyl)-4-(p-tolylamino)-2,5-dihydro-1H-pyrrole-3-carboxylate (**4a**).*

^{1}H NMR (400 MHz, $CDCl_3$)

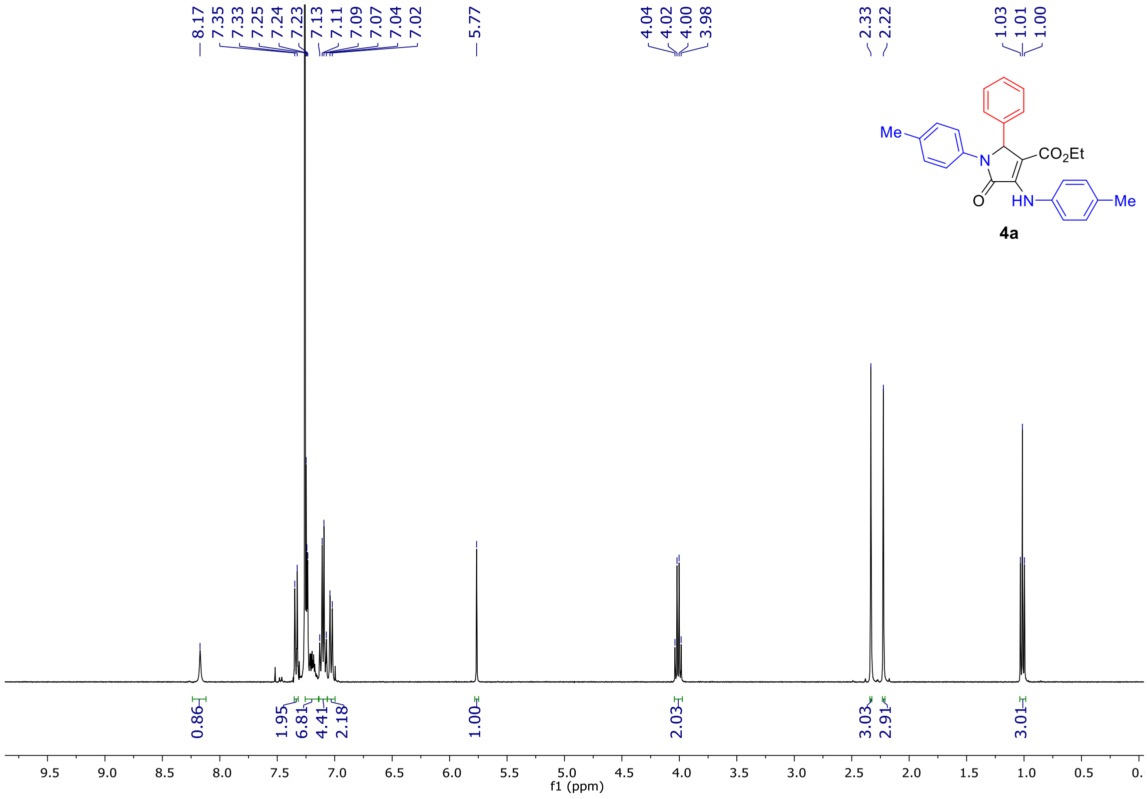

^{13}C {^{1}H} NMR (101 MHz, $CDCl_3$)

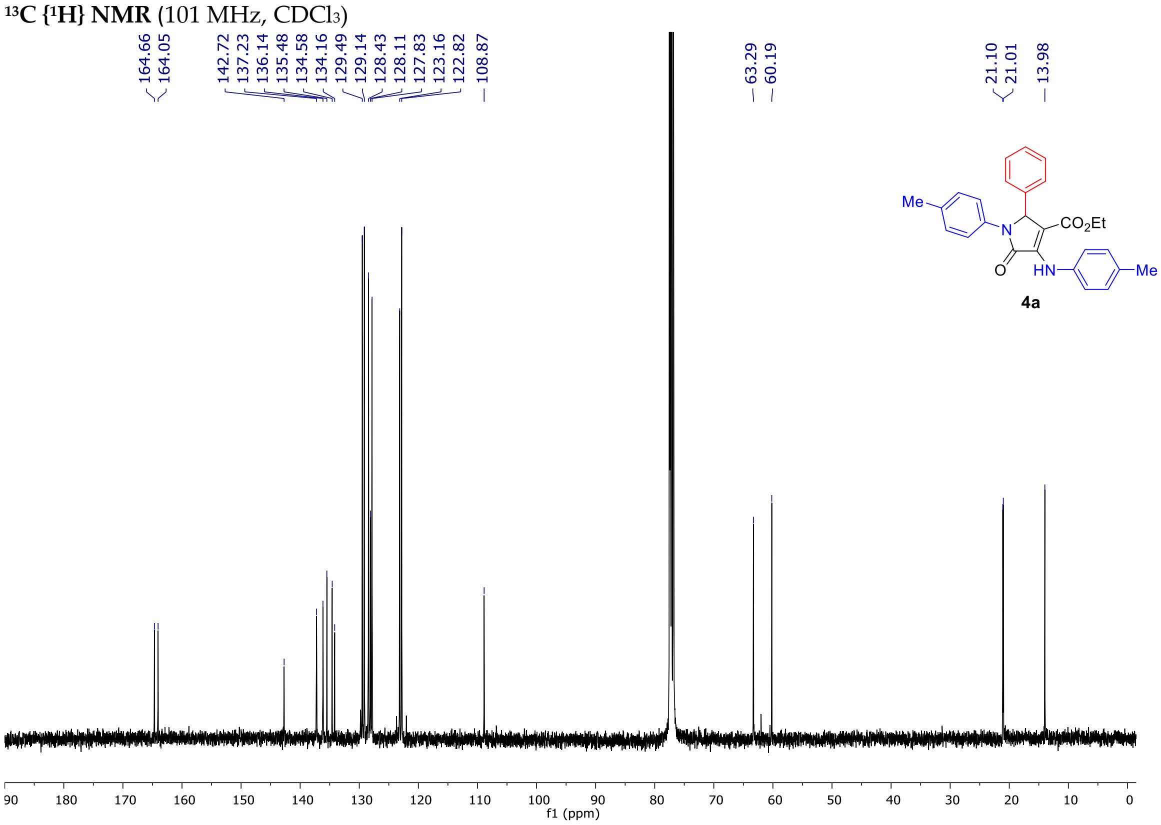

*Ethyl 1-(4-methoxyphenyl)-4-((4-methoxyphenyl)amino)-5-oxo-2-phenyl-2,5-dihydro-1H-pyrrole-3-carboxylate (**4b**).*

^{1}**H NMR** (300 MHz, $CDCl_3$)

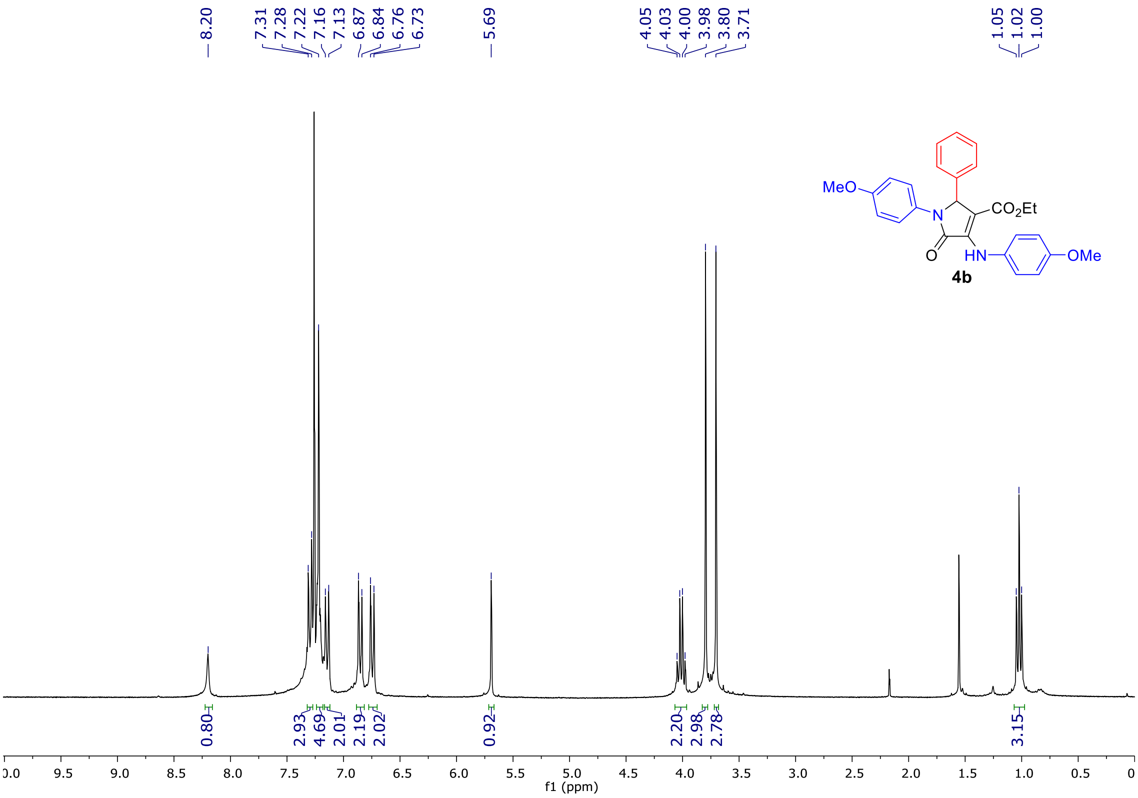

^{13}**C {^{1}H} NMR** (75 MHz, $CDCl_3$)

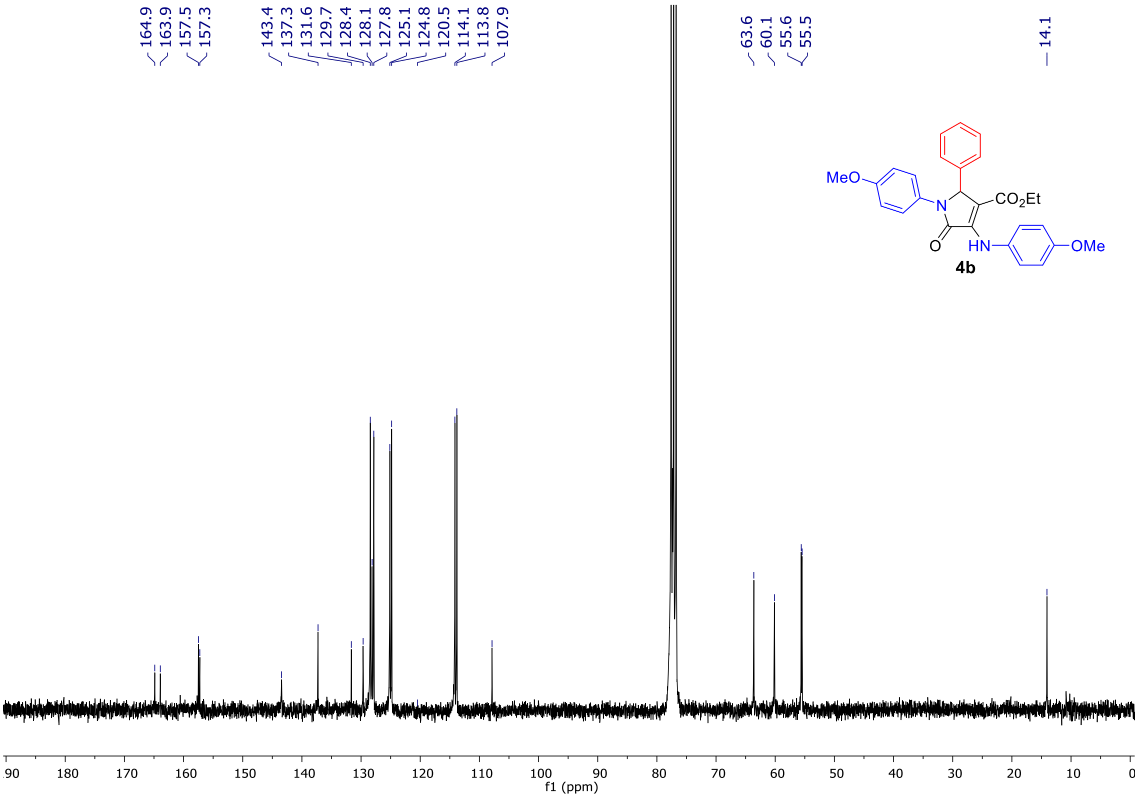

*Ethyl 1-benzyl-4-(benzylamino)-5-oxo-2-phenyl-2,5-dihydro-1H-pyrrole-3-carboxylate (**4c**).*

^{1}H NMR (400 MHz, $CDCl_3$)

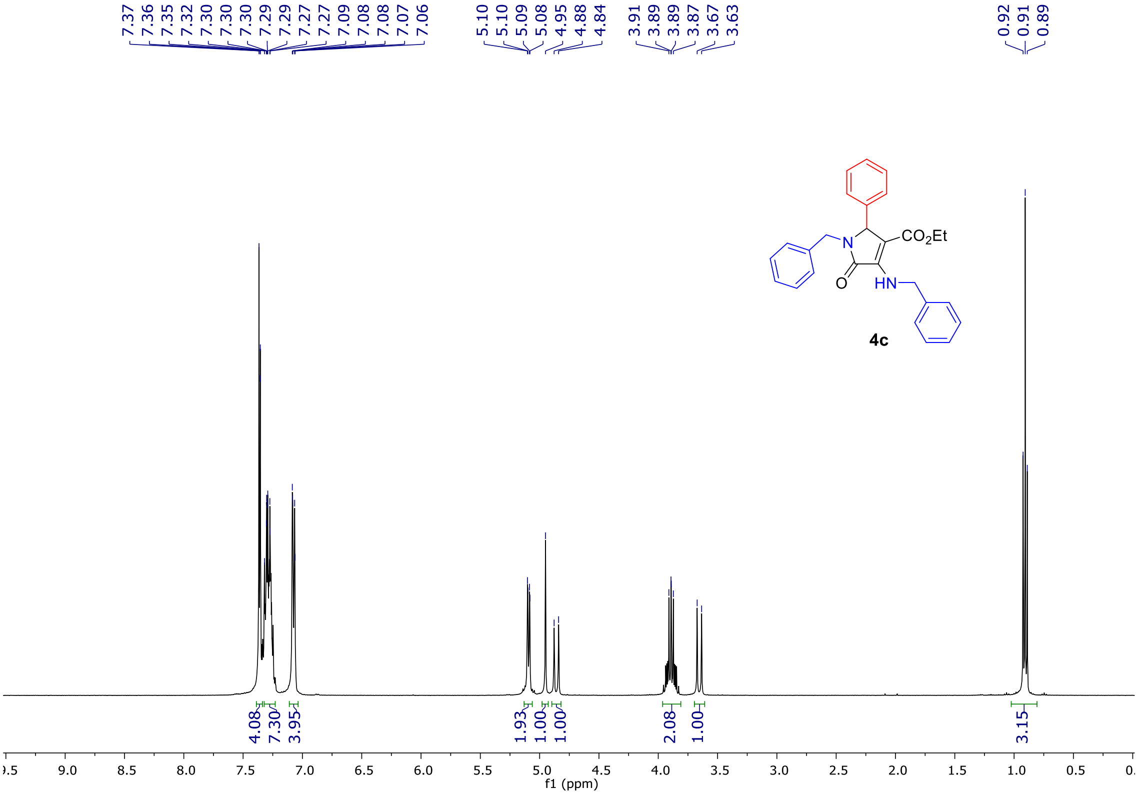

^{13}C {^{1}H} NMR (101 MHz, DMSO *d*$_6$)

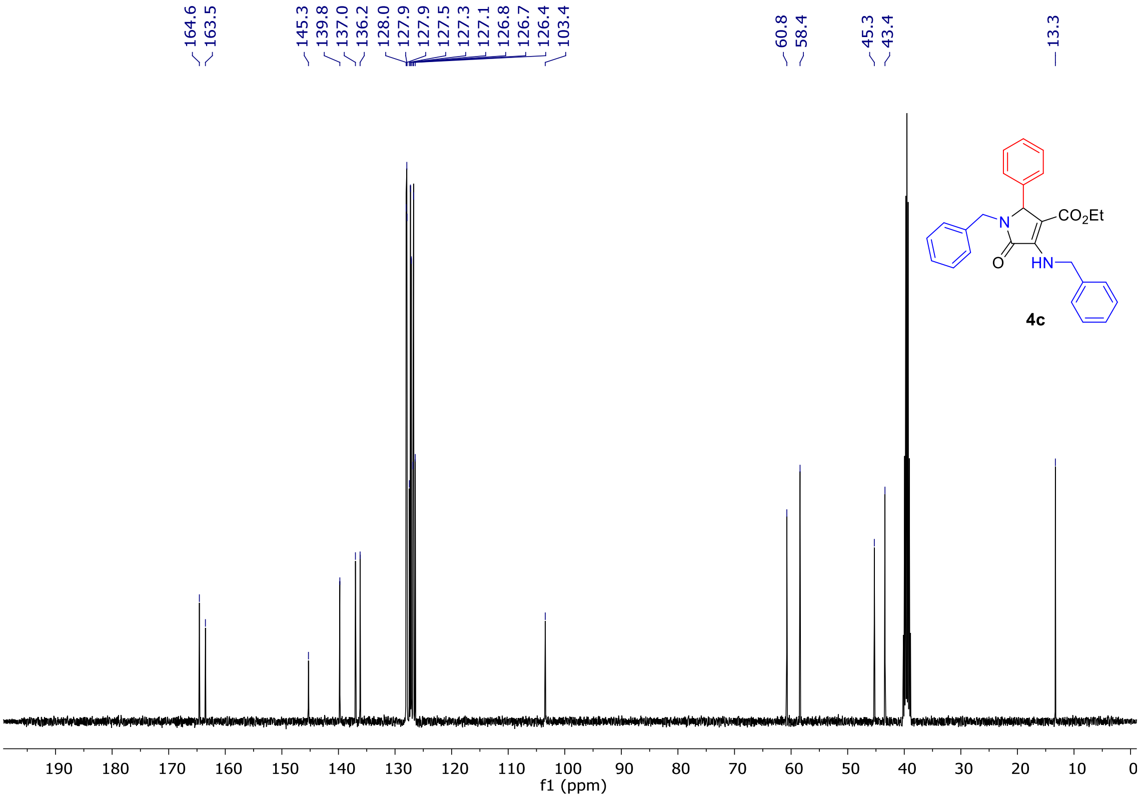

*Isopropyl 5-oxo-2-phenyl-1-(p-tolyl)-4-(p-tolylamino)-2,5-dihydro-1H-pyrrole-3-carboxylate (**4d**).*

^{1}H NMR (400 MHz, $CDCl_3$)

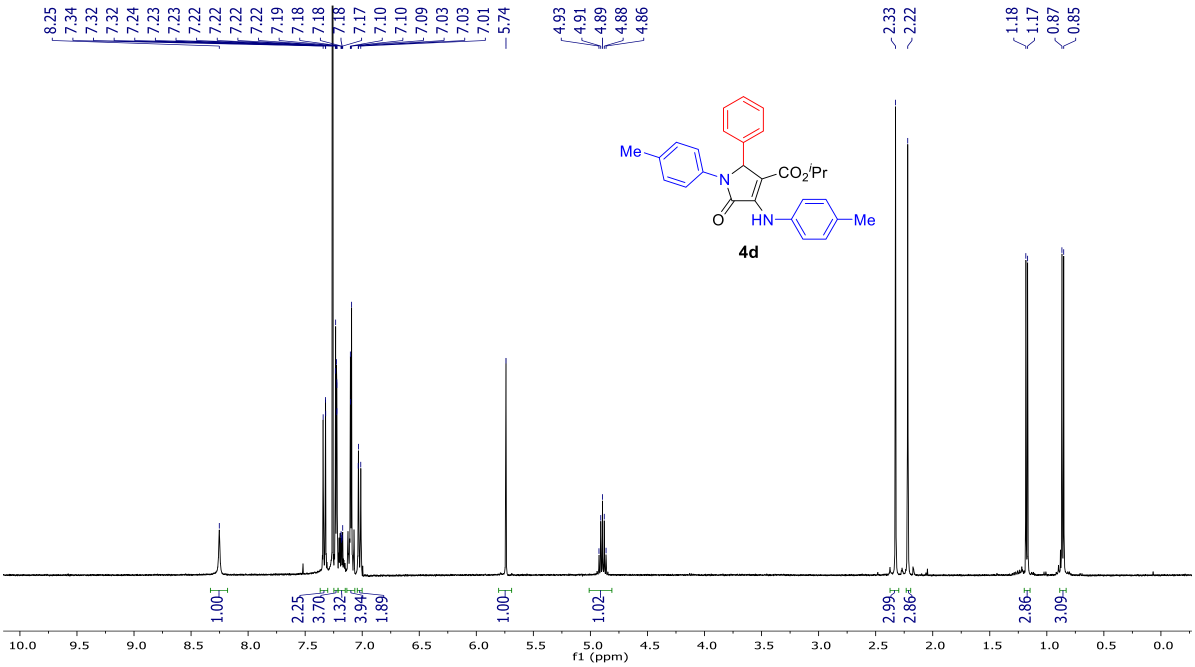

^{13}C {^{1}H} NMR (101 MHz, $CDCl_3$)

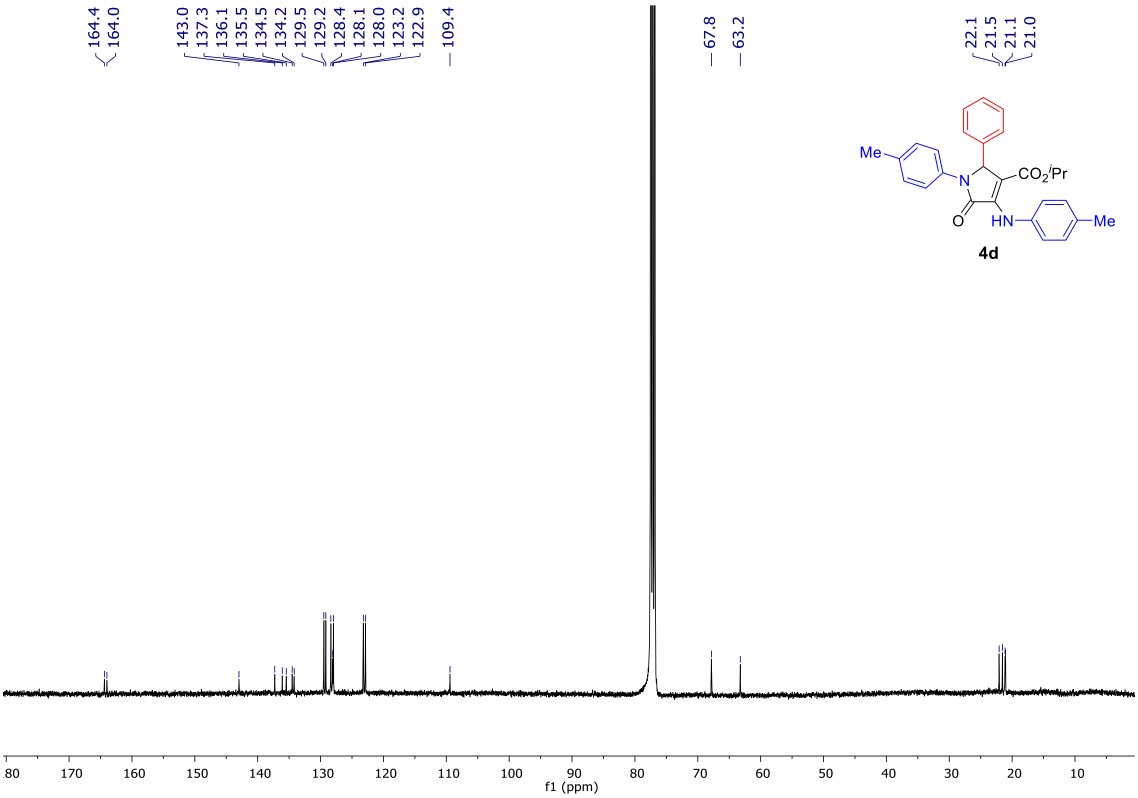

*Methyl 5-oxo-2-phenyl-1-(p-tolyl)-4-(p-tolylamino)-2,5-dihydro-1H-pyrrole-3-carboxylate (**4e**).*

^{1}H NMR (400 MHz, $CDCl_3$)

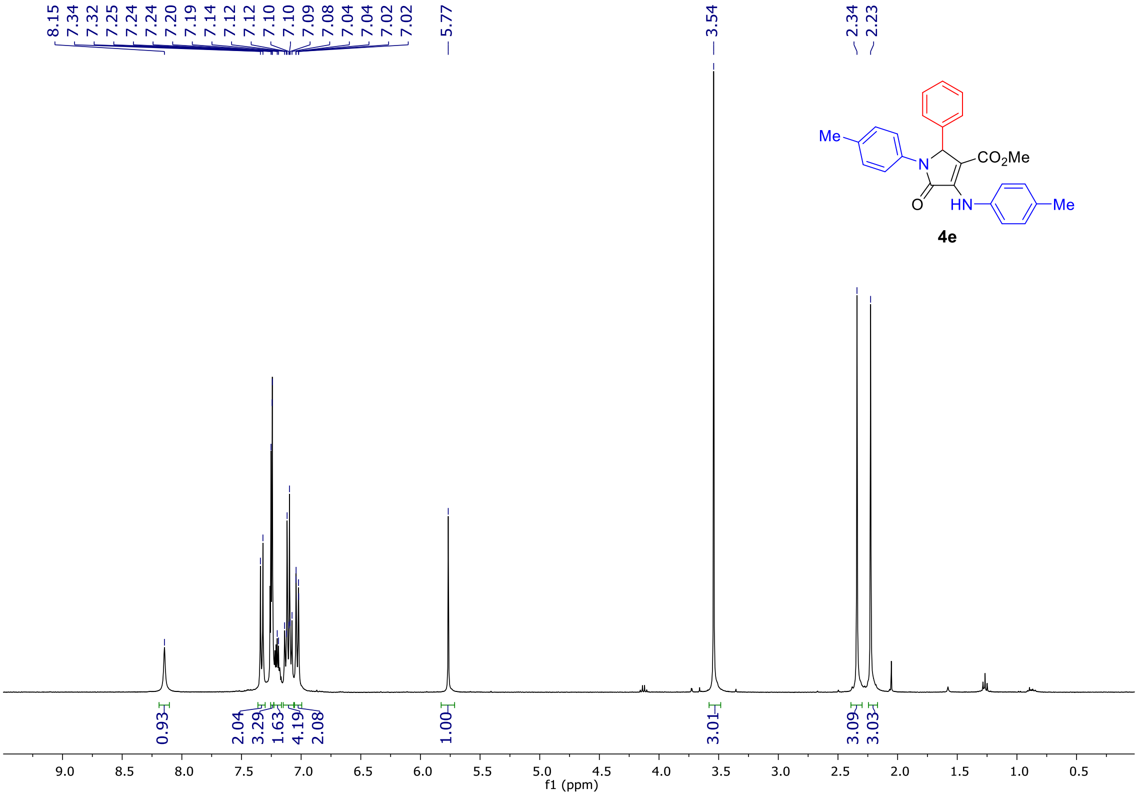

^{13}C {^{1}H} NMR (101 MHz, $CDCl_3$)

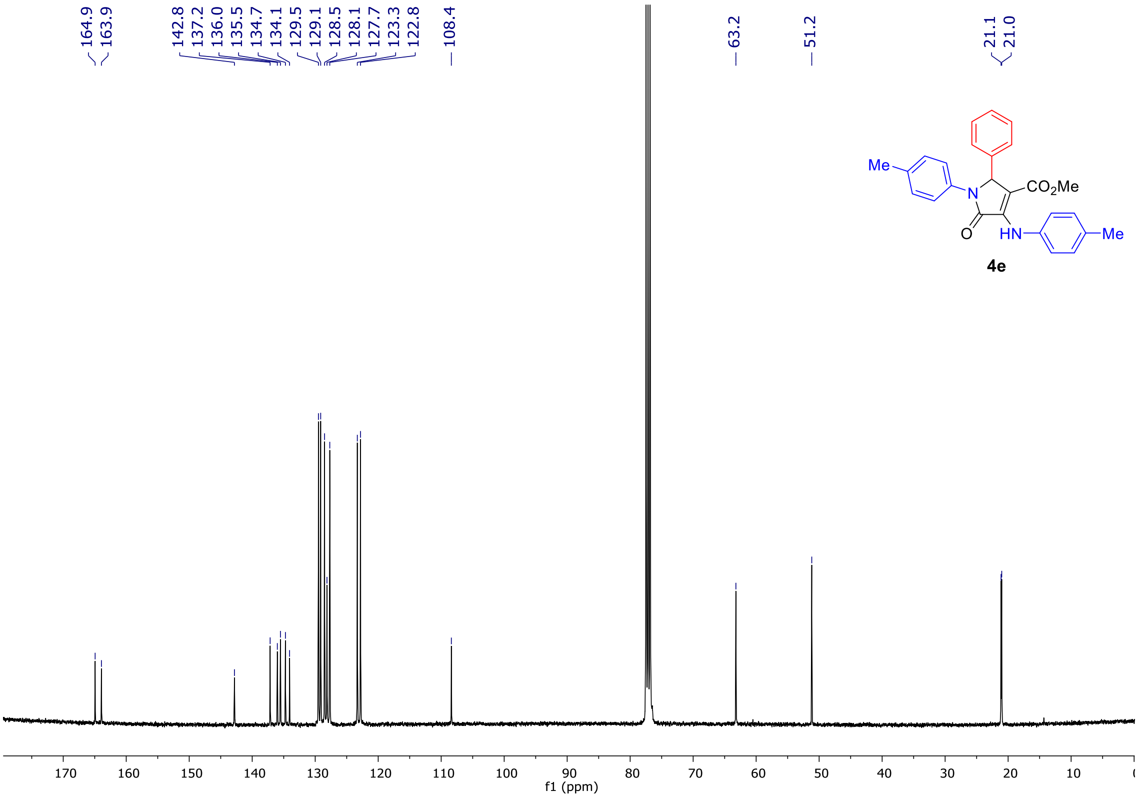

*Methyl 5-oxo-1-(p-tolyl)-4-(p-tolylamino)-2-(4-(trifluoromethyl)phenyl)-2,5-dihydro-1H-pyrrole-3-carboxylatecarboxylate (**4f**).*

¹H NMR (400 MHz, $CDCl_3$)

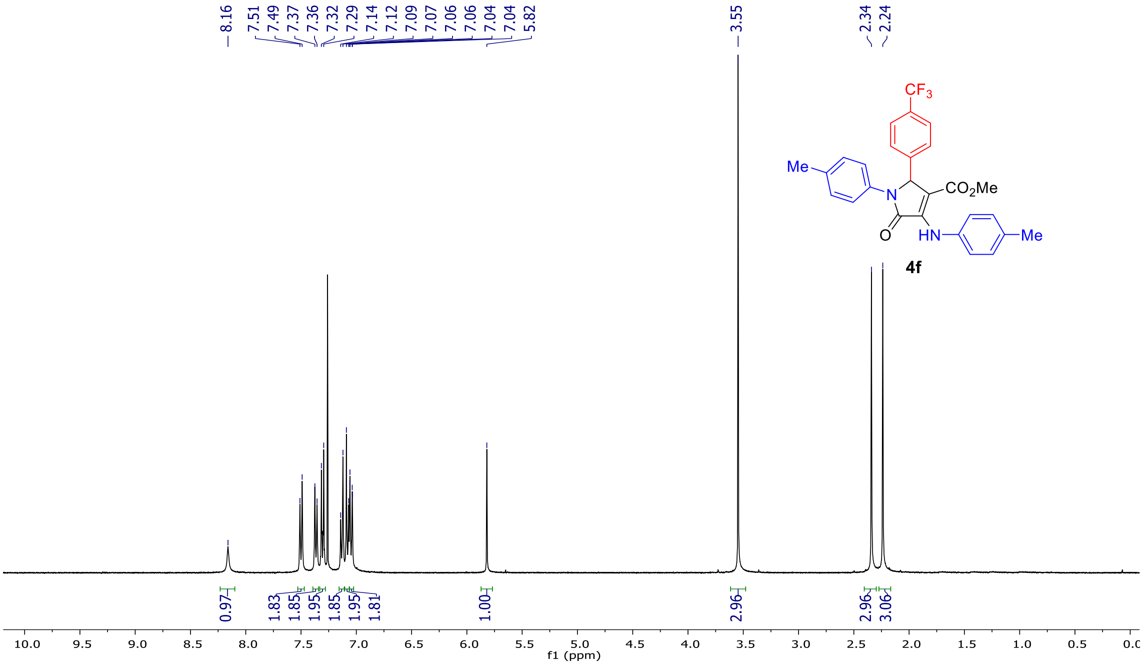

¹³C {¹H} NMR (101 MHz, $CDCl_3$)

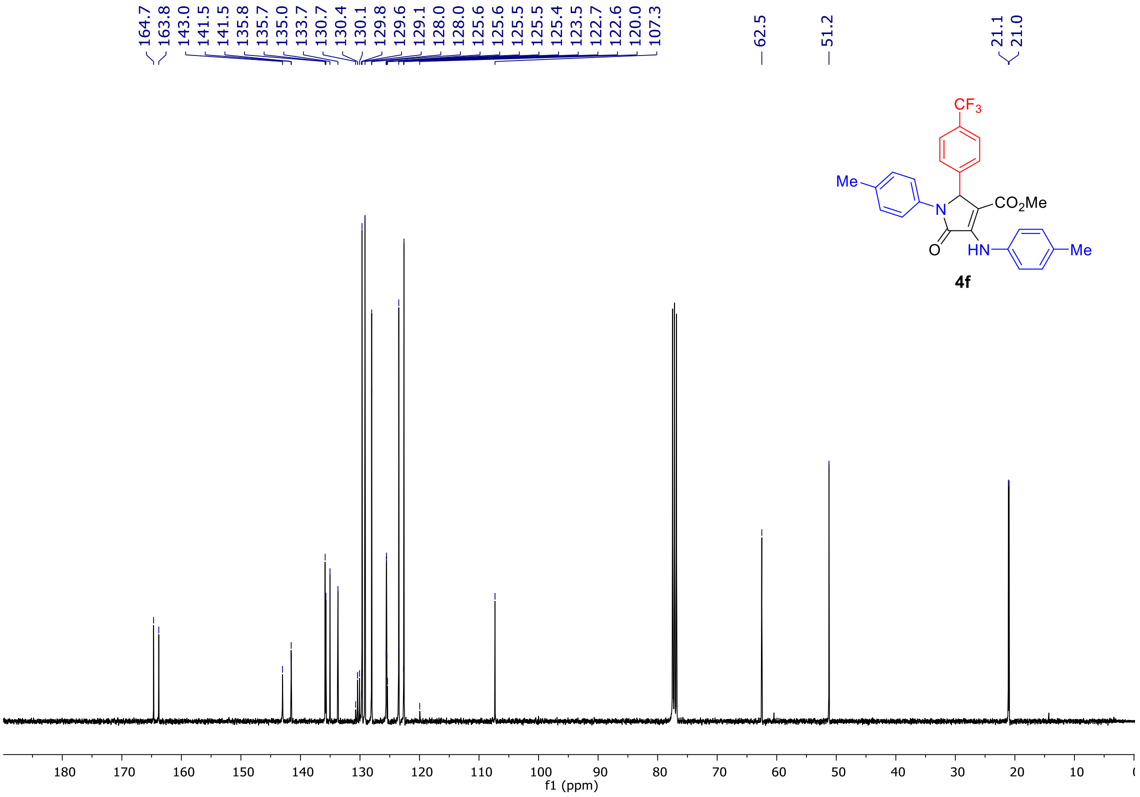

^{19}F NMR (282 MHz, $CDCl_3$)

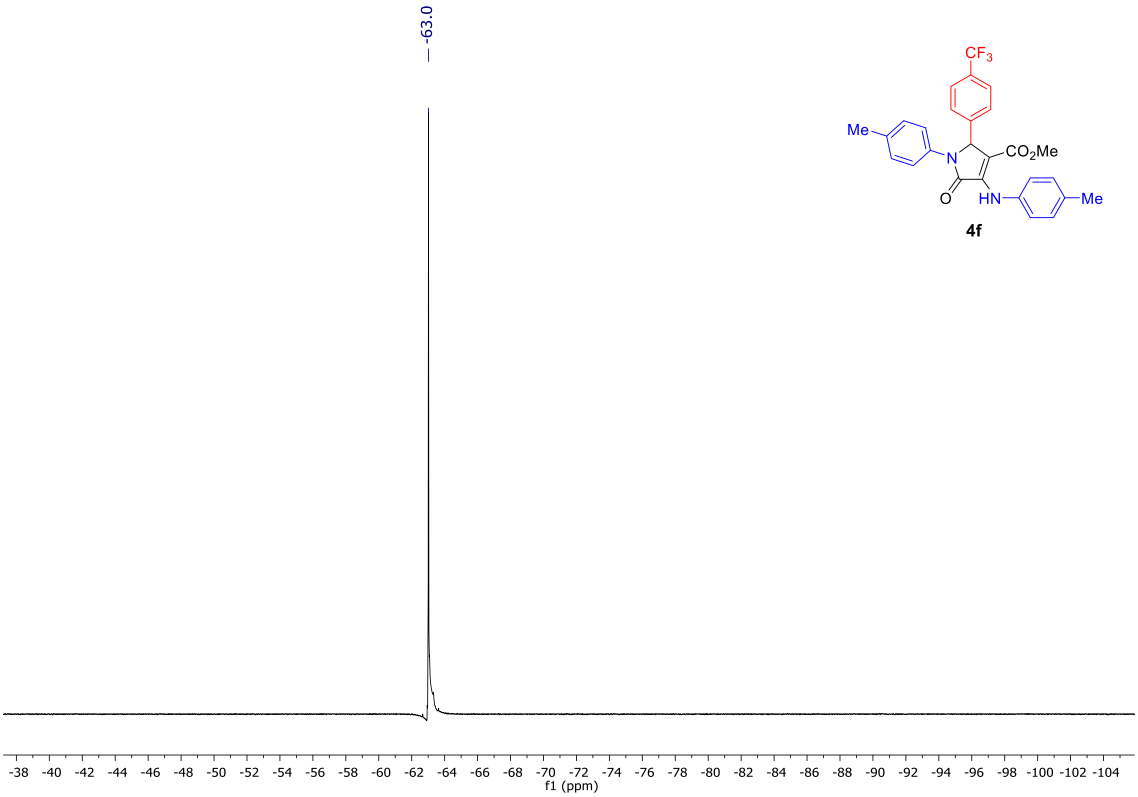

4f

*Methyl 1-benzyl-4-(benzylamino)-5-oxo-2-(4-(trifluoromethyl)phenyl)-2,5-dihydro-1H-pyrrole-3-carboxylate (**4g**).*

^{1}H NMR (400 MHz, DMSO-d6, 60°C)

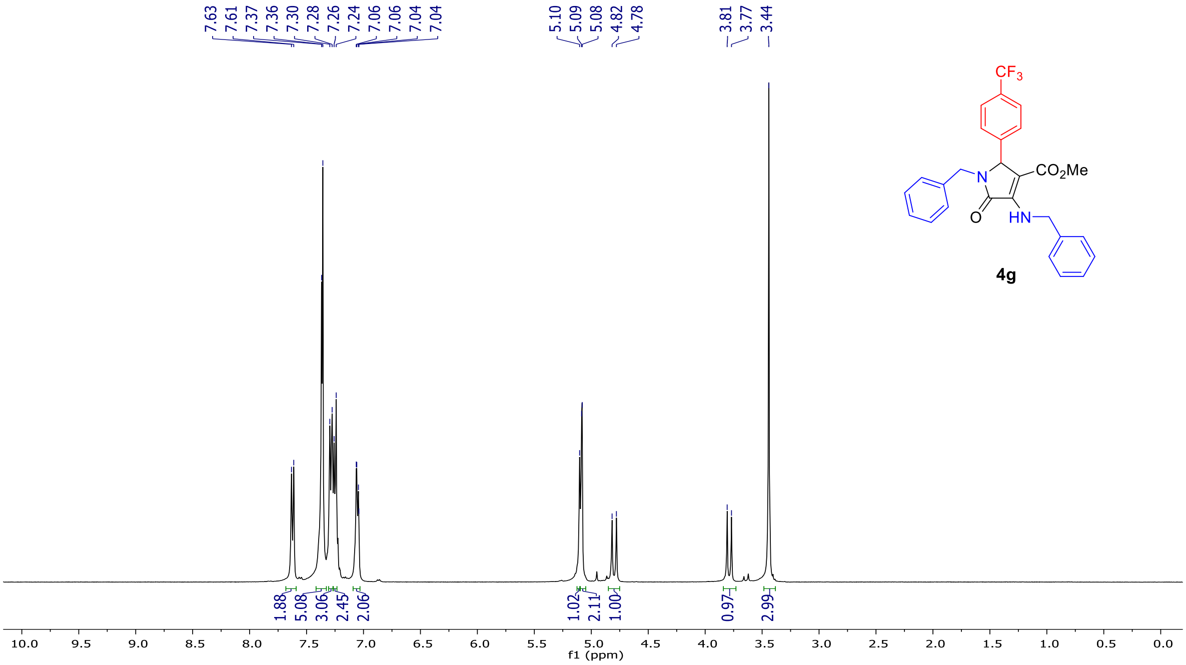

^{13}C {^{1}H} NMR (101 MHz, DMSO-d6, 60°C)

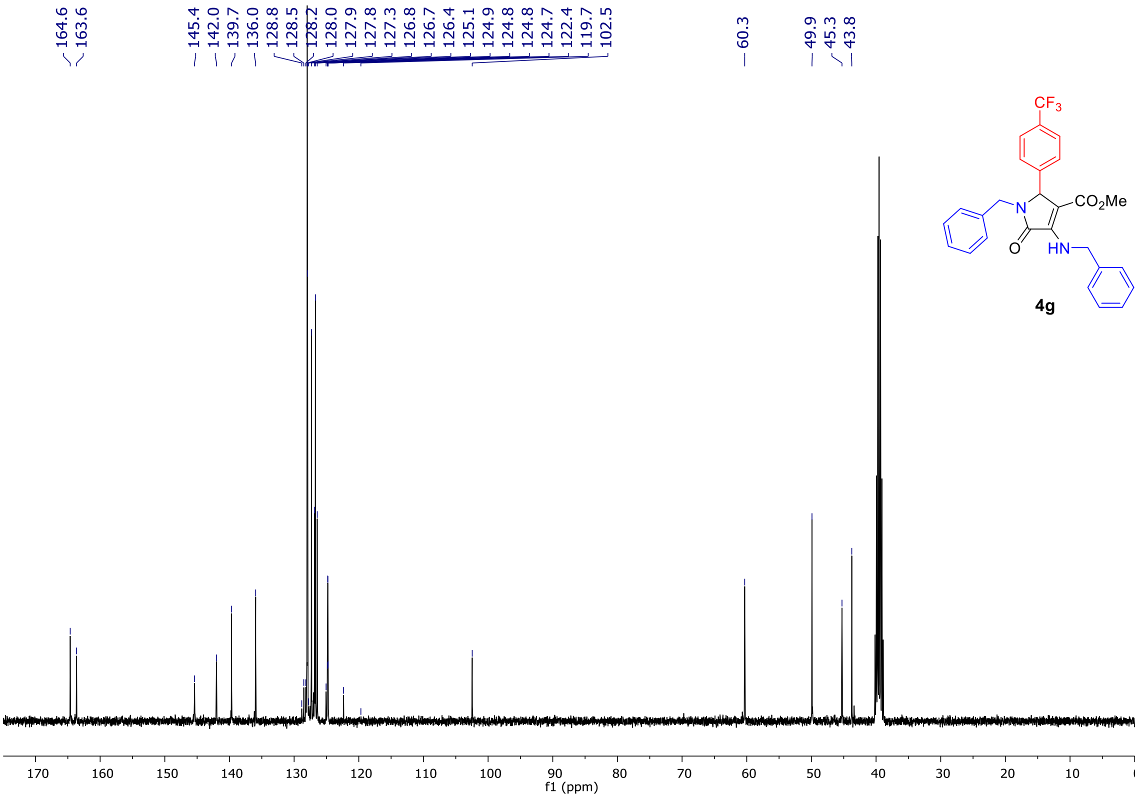

^{19}F NMR (282 MHz, DMSO-d$_6$, 60°C)

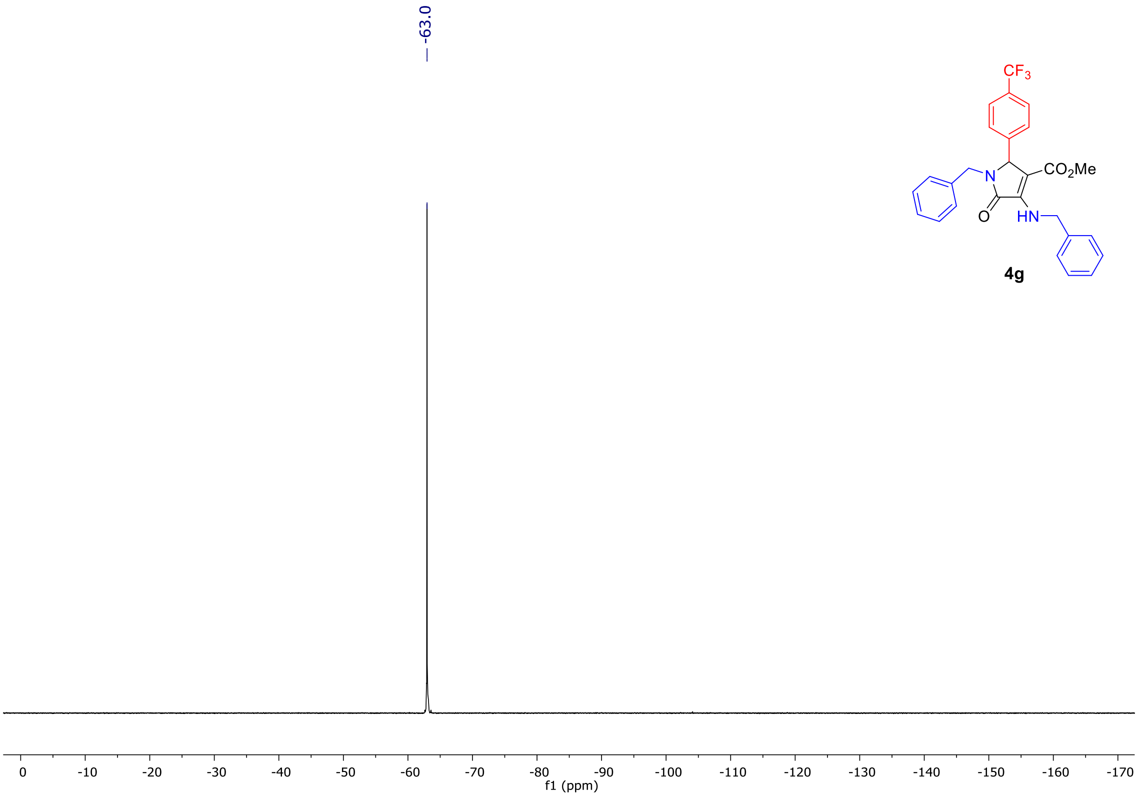

*Methyl 2-(4-hydroxyphenyl)-5-oxo-1-(p-tolyl)-4-(p-tolylamino)-2,5-dihydro-1H-pyrrole-3-carboxylate (**4h**).*

^{1}H NMR (400 MHz, $CDCl_3$)

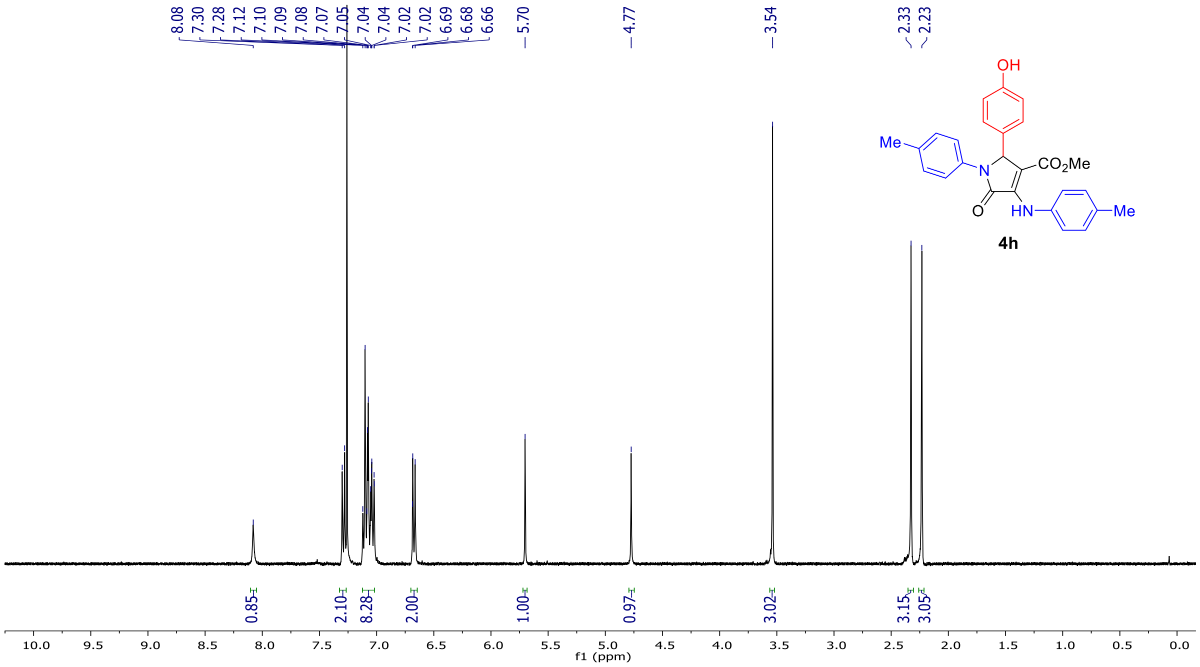

^{13}C {^{1}H} NMR (101 MHz, $CDCl_3$)

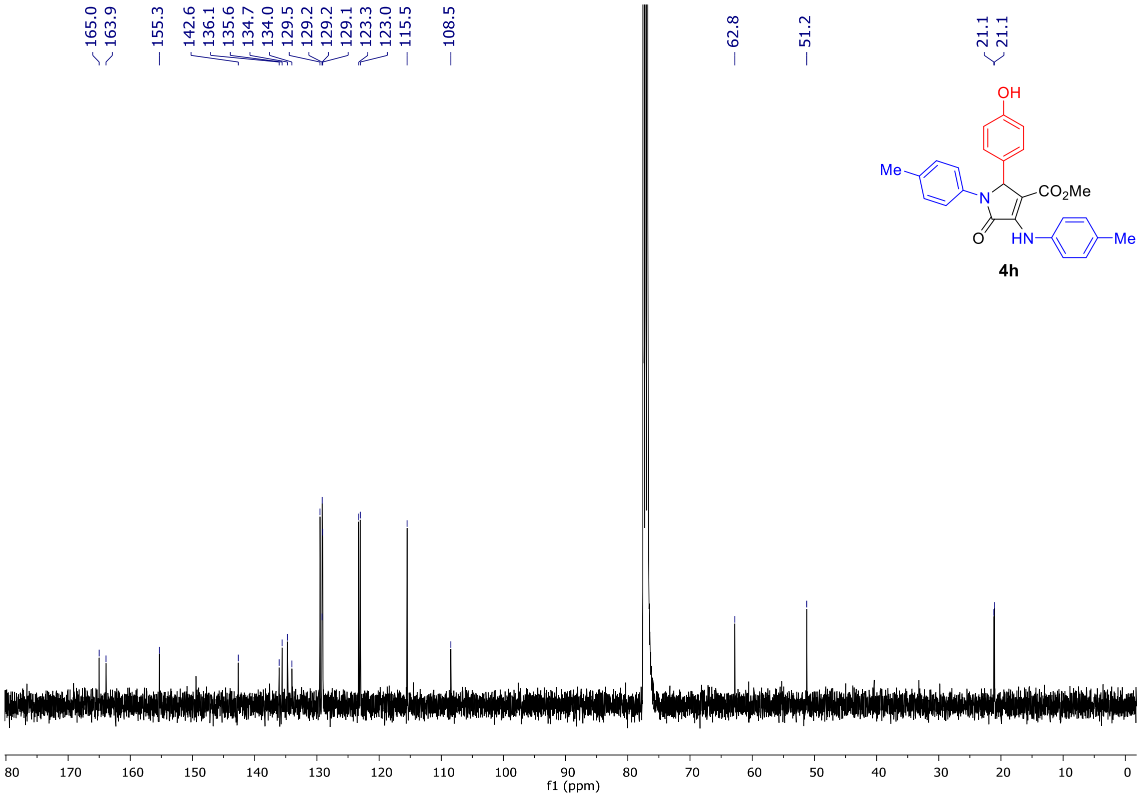

*Methyl 1-benzyl-4-(benzylamino)-2-(4-hydroxyphenyl)-5-oxo-2,5-dihydro-1H-pyrrole-3-carboxylate (**4i**).*

^{1}H NMR (400 MHz, DMSO-d6, 60°C

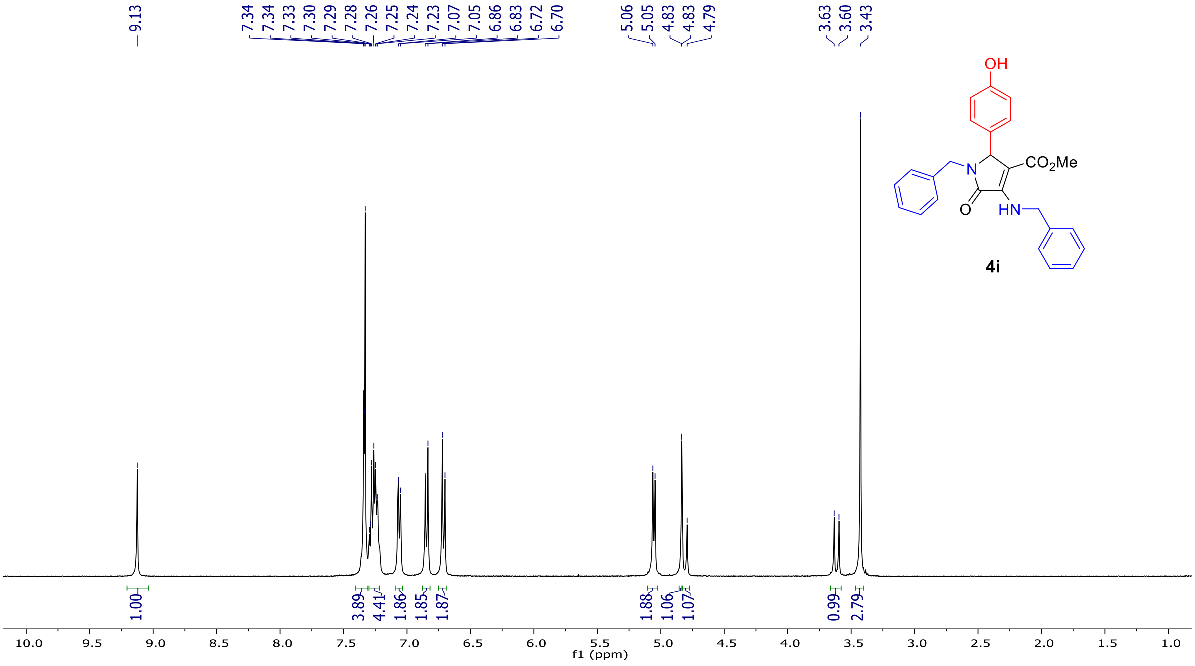

^{13}C {^{1}H} NMR (101 MHz, DMSO-d6, 60°C)

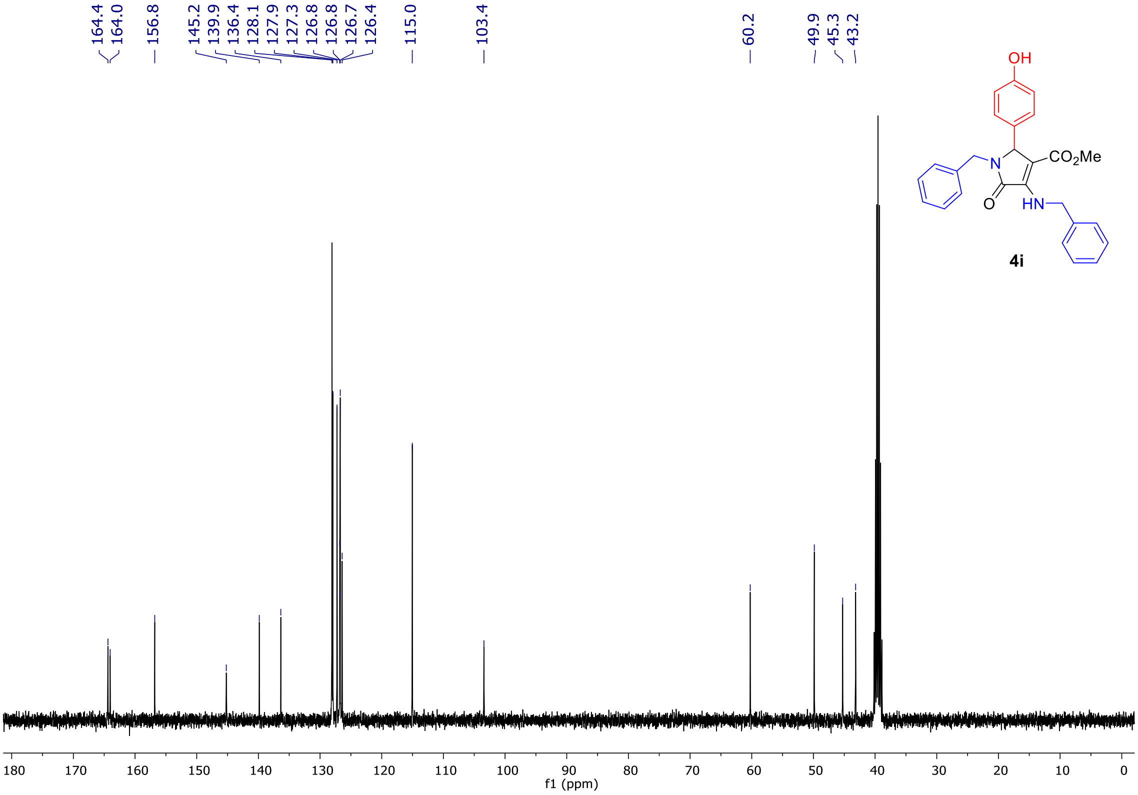

*Methyl 2-(3-methoxyphenyl)-5-oxo-1-(p-tolyl)-4-(p-tolylamino)-2,5-dihydro-1H-pyrrole-3-carboxylate (**4j**).*

^{1}H NMR (400 MHz, $CDCl_3$)

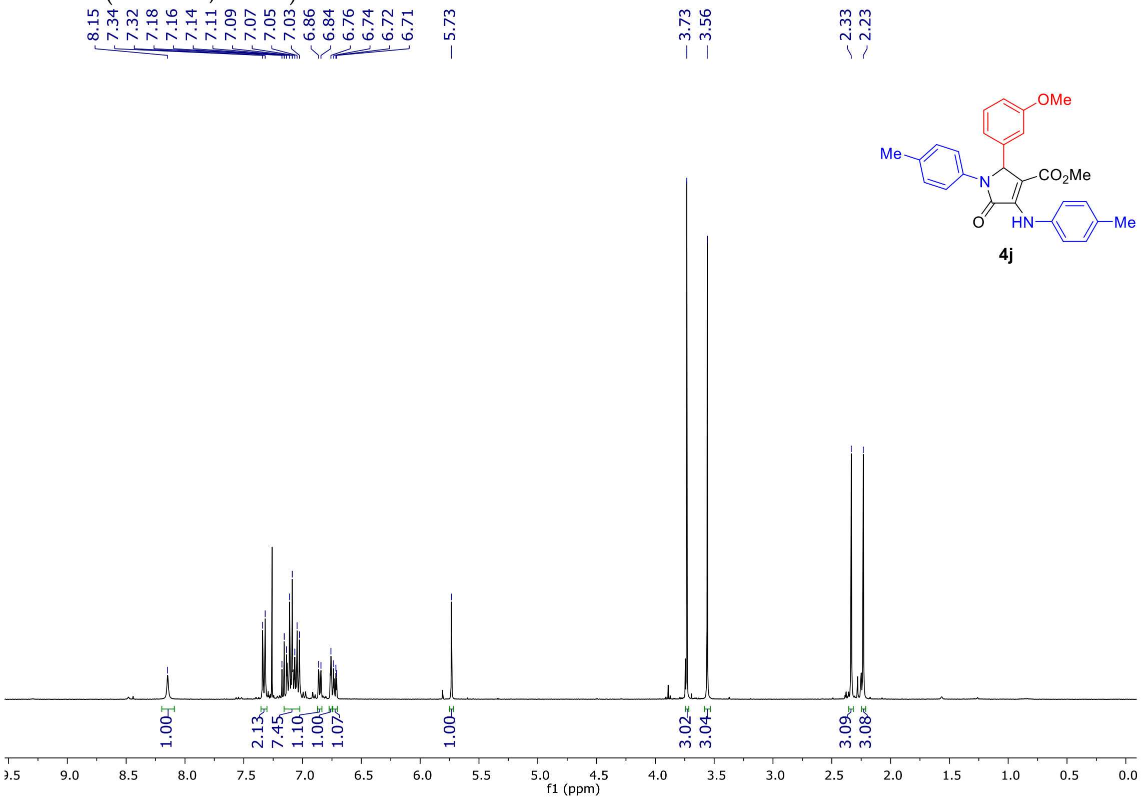

^{13}C {^{1}H} NMR (101 MHz, $CDCl_3$)

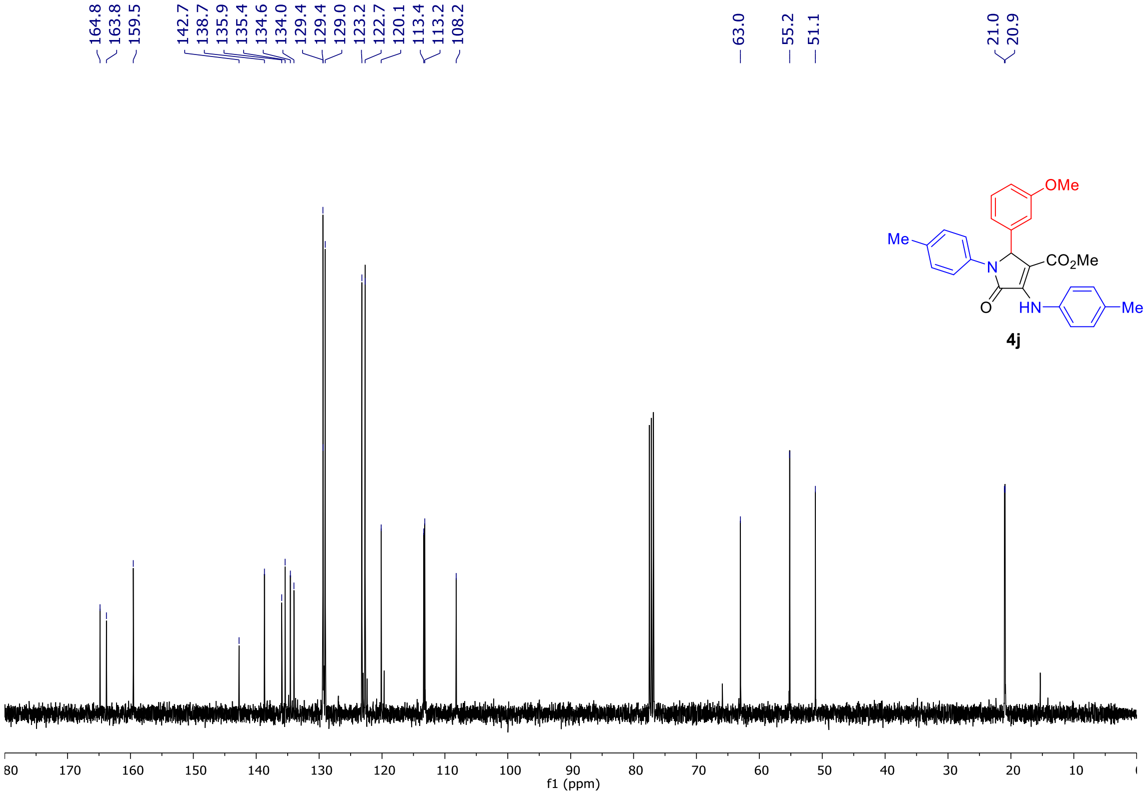

*Methyl 1-benzyl-4-(benzylamino)-2-(3-methoxyphenyl)-5-oxo-2,5-dihydro-1H-pyrrole-3-carboxylate (**4k**).*

^{1}H NMR (400 MHz, DMSO-d_6, 60°C)

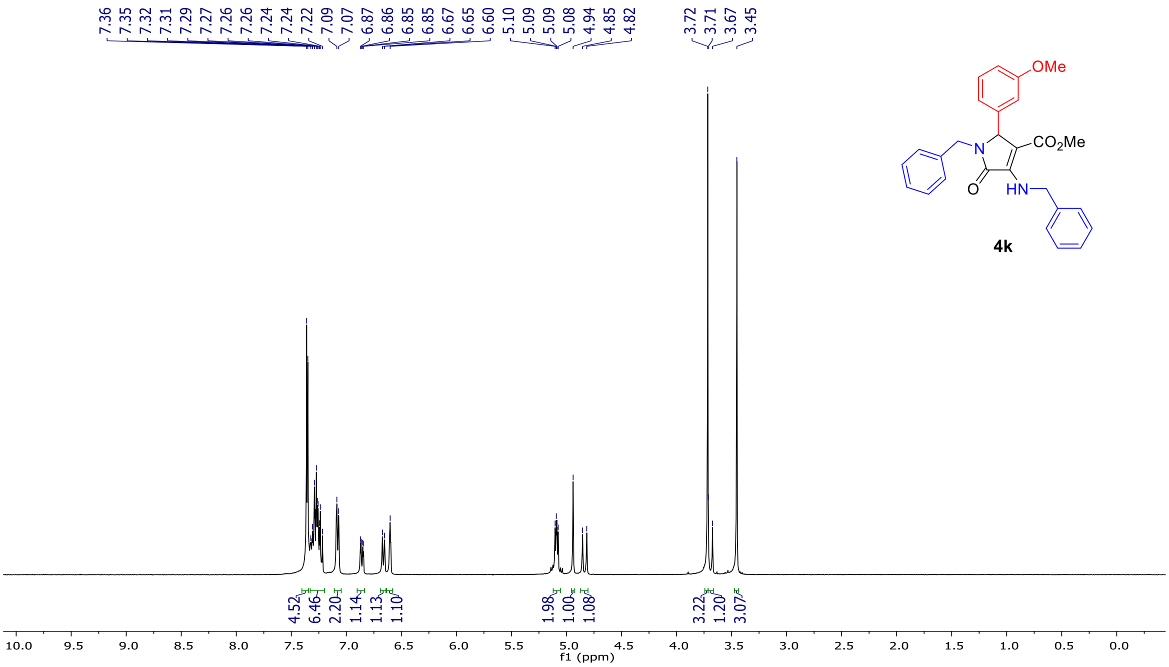

^{13}C {^{1}H} NMR (101 MHz, DMSO-d$_6$, 60°C)

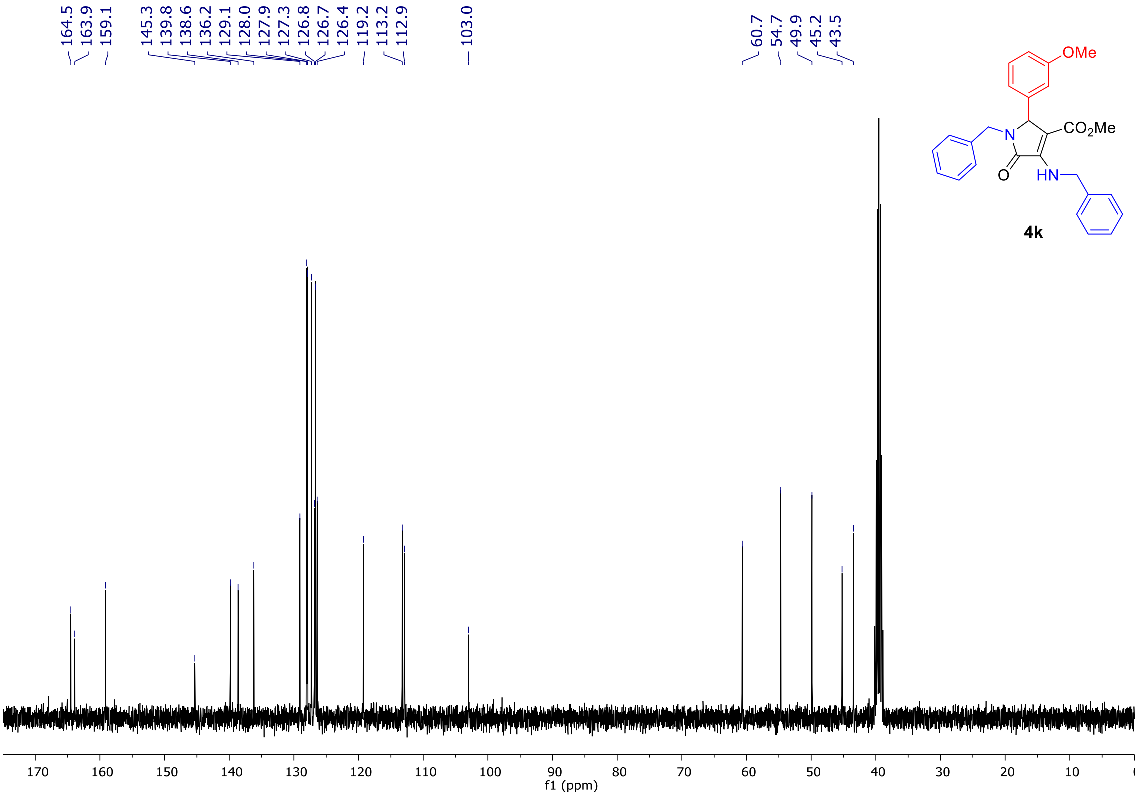

*Methyl 2-(4-hydroxy-3-methoxyphenyl)-5-oxo-1-(p-tolyl)-4-(p-tolylamino)-2,5-dihydro-1H-pyrrole-3-carboxylate (**4l**).*

^{1}H NMR (400 MHz, $CDCl_3$)

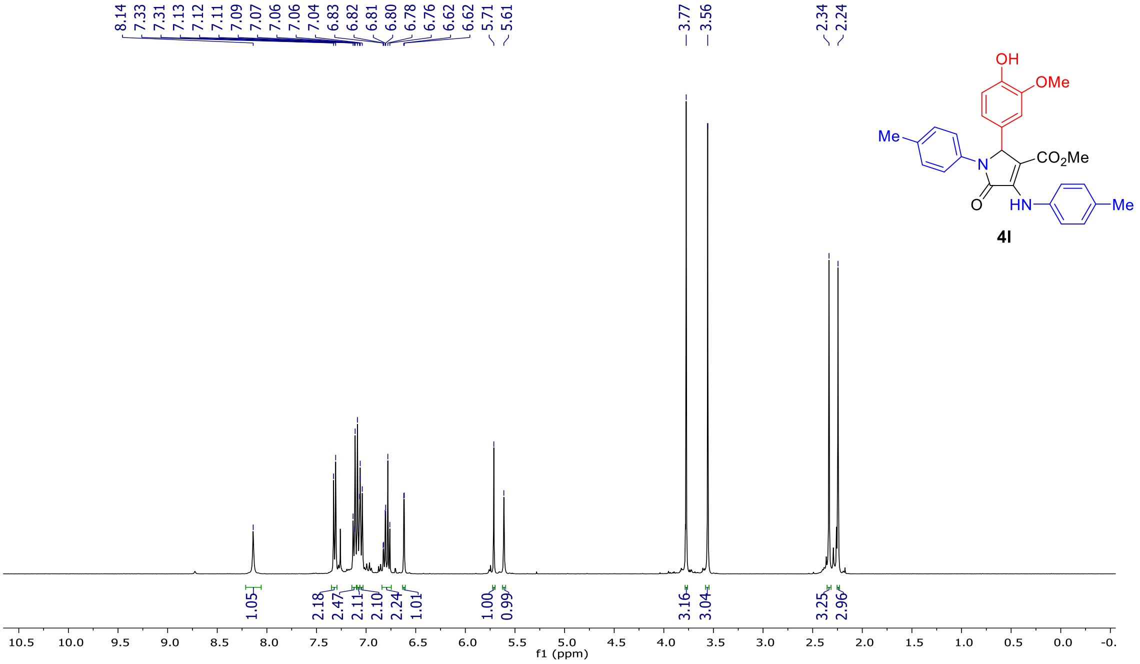

^{13}C {^{1}H} NMR (101 MHz, $CDCl_3$)

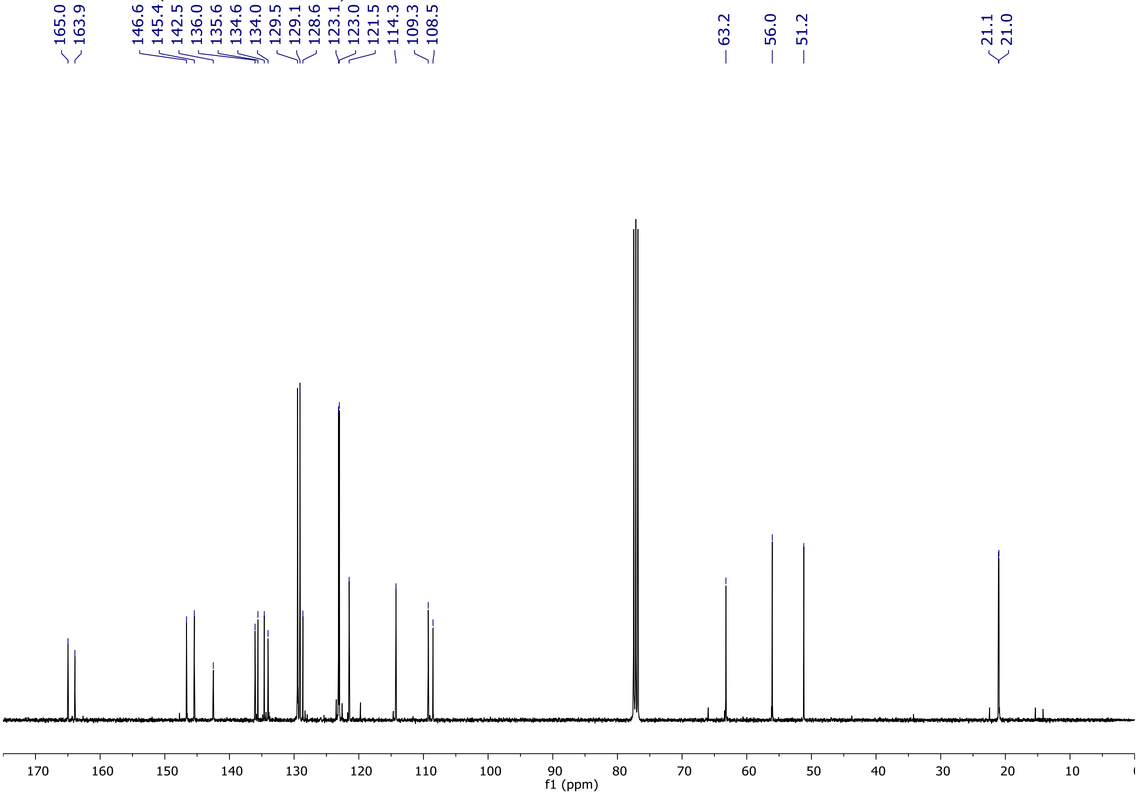

*Methyl 1-benzyl-4-(benzylamino)-2-(4-hydroxy-3-methoxyphenyl)-5-oxo-2,5-dihydro-1H-pyrrole-3-carboxylate (**4m**).*

^{1}H NMR (400 MHz, DMSO-d_6, 60°C)

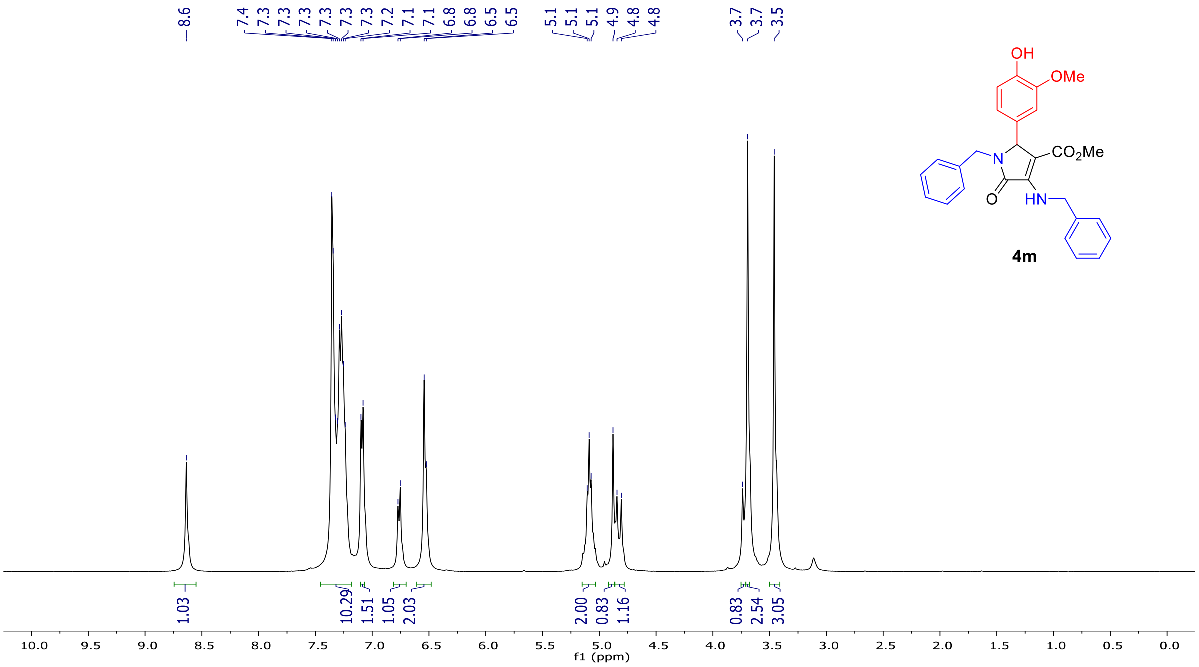

^{13}C {^{1}H} NMR (101 MHz, DMSO-d_6, 60°C)

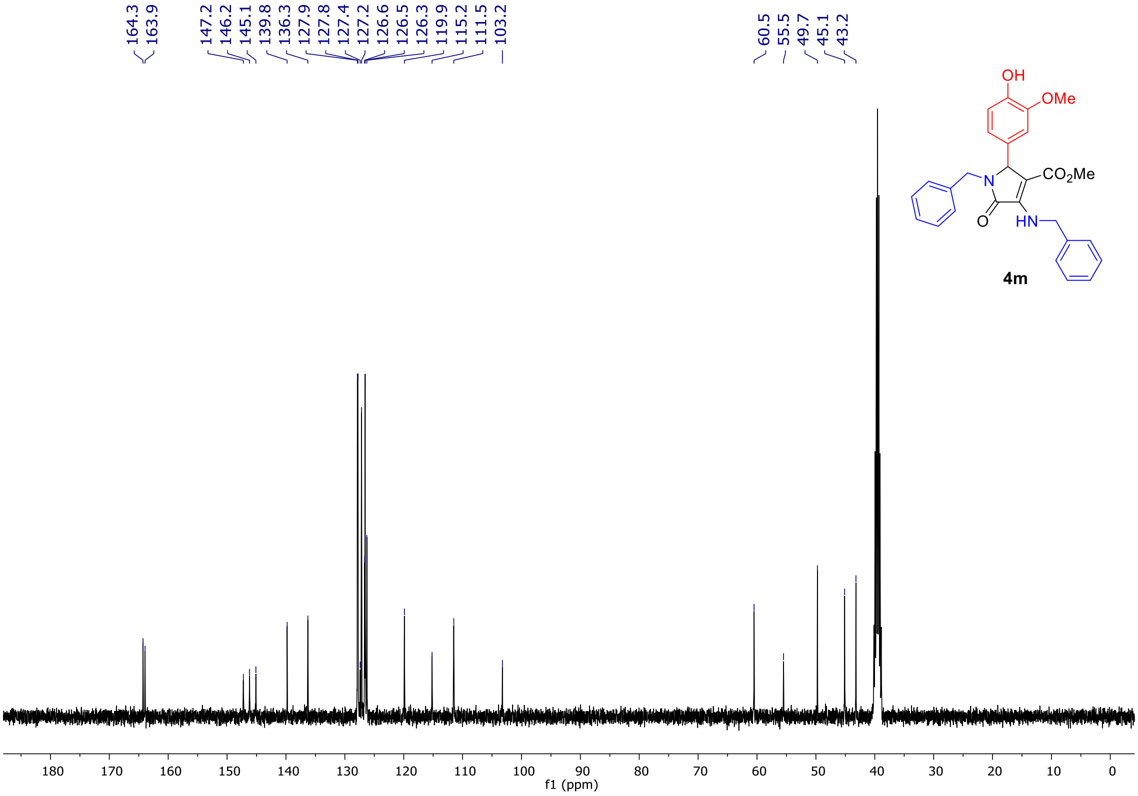

*5-oxo-2-phenyl-N,1-di-p-tolyl-4-(p-tolylamino)-2,5-dihydro-1H-pyrrole-3-carboxamide (**8a**).*

^{1}H NMR (300 MHz, $CDCl_3$)

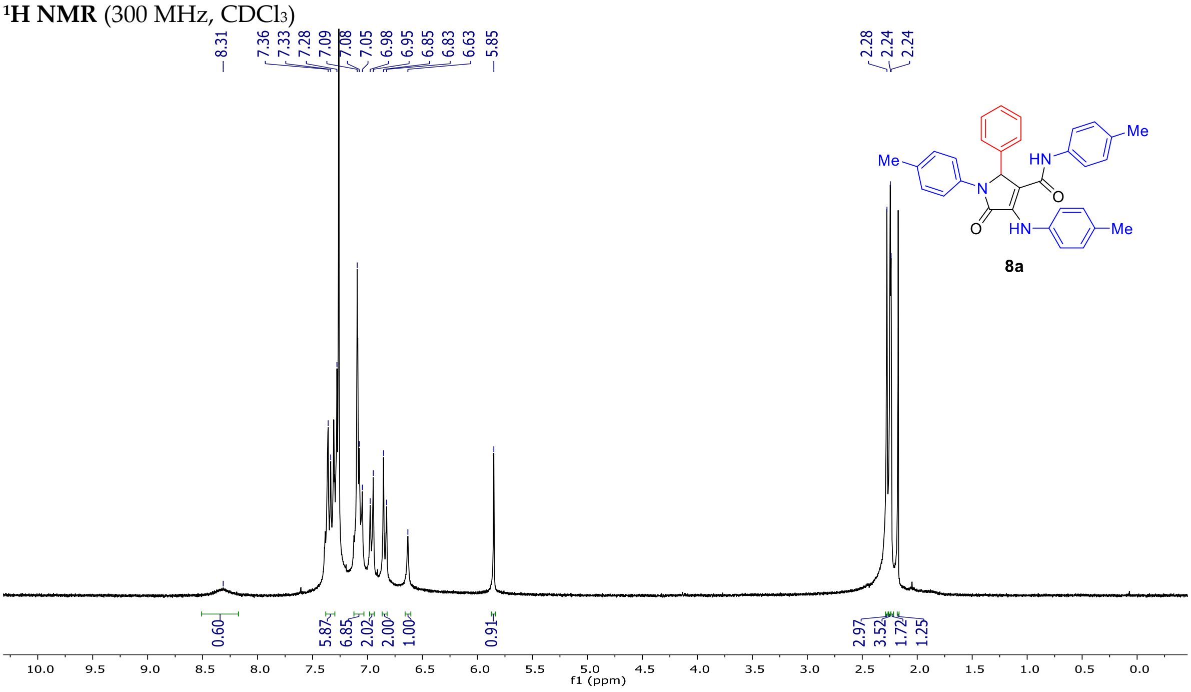

^{13}C {^{1}H} NMR (75 MHz, $CDCl_3$)

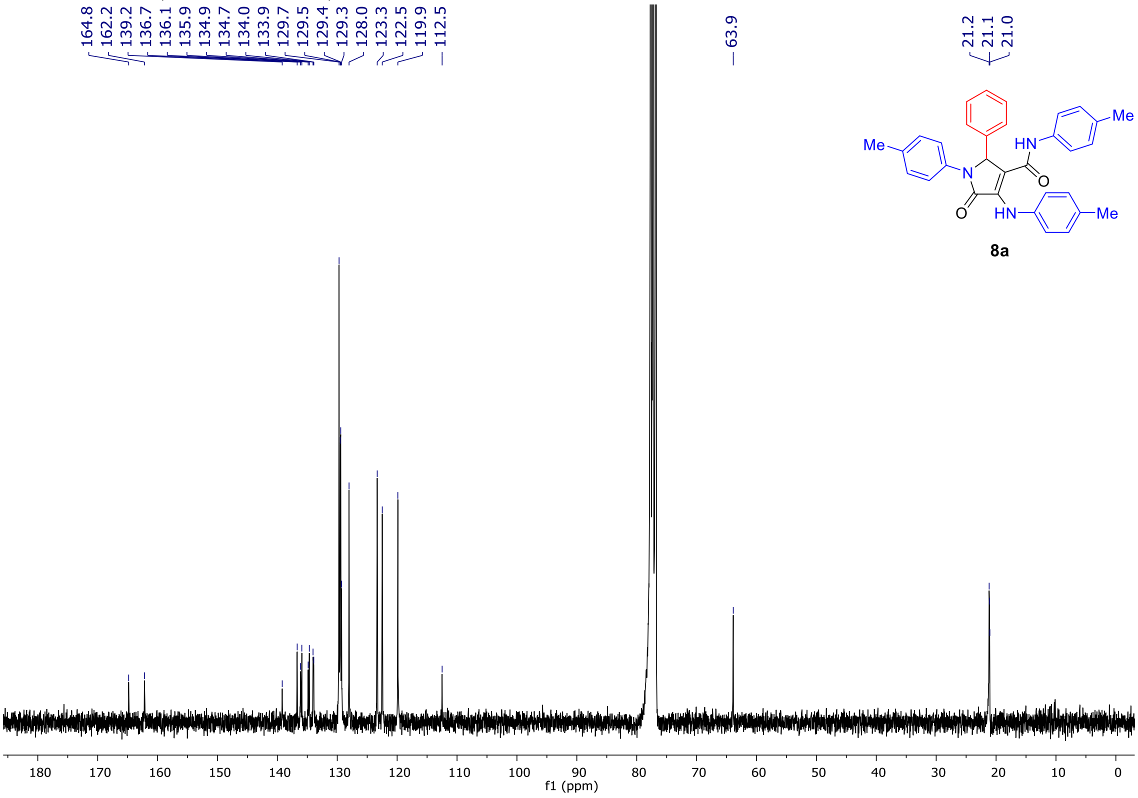

*N,1-bis(4-methoxyphenyl)-4-((4-methoxyphenyl)amino)-5-oxo-2-phenyl-2,5-dihydro-1H-pyrrole-3-carboxamide (**8b**).*

^{1}H NMR (400 MHz, $CDCl_3$)

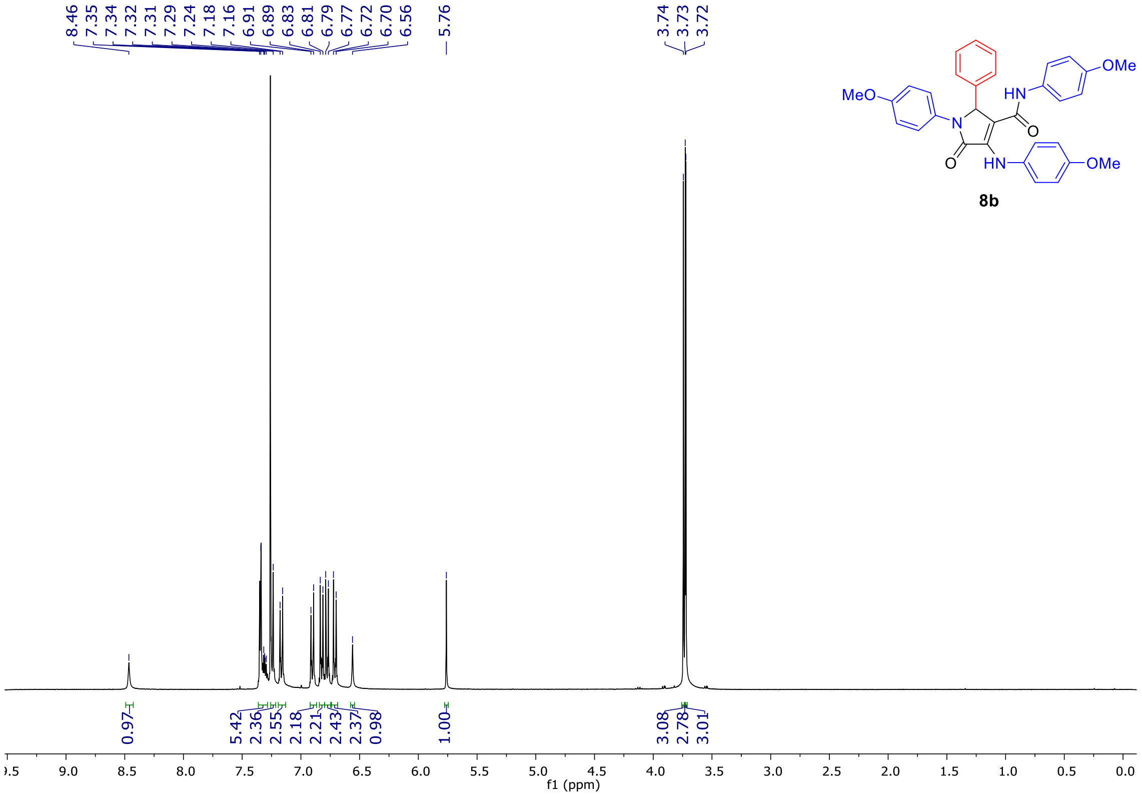

^{13}C {^{1}H} NMR (101 MHz, $CDCl_3$)

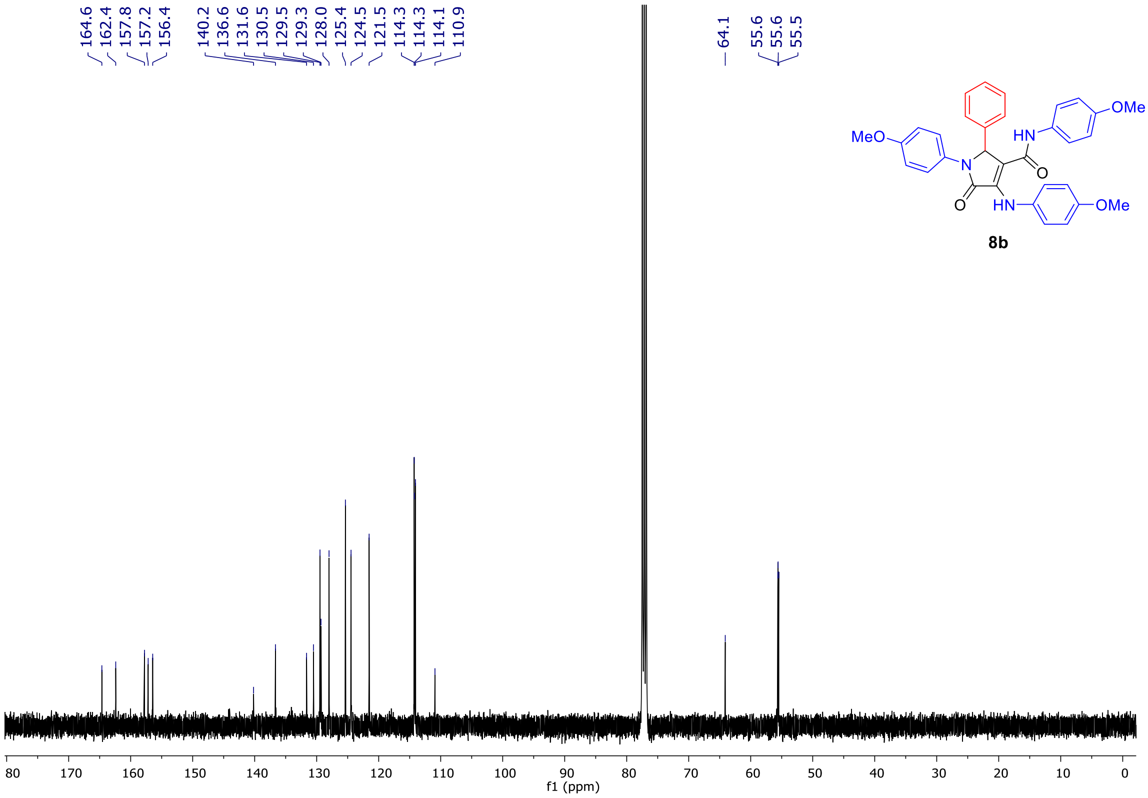

Ethyl 4-amino-1-benzyl-5-oxo-2-phenyl-2,5-dihydro-1H-pyrrole-3-carboxylate (**9**).

^{1}H NMR (400 MHz, $CDCl_3$)

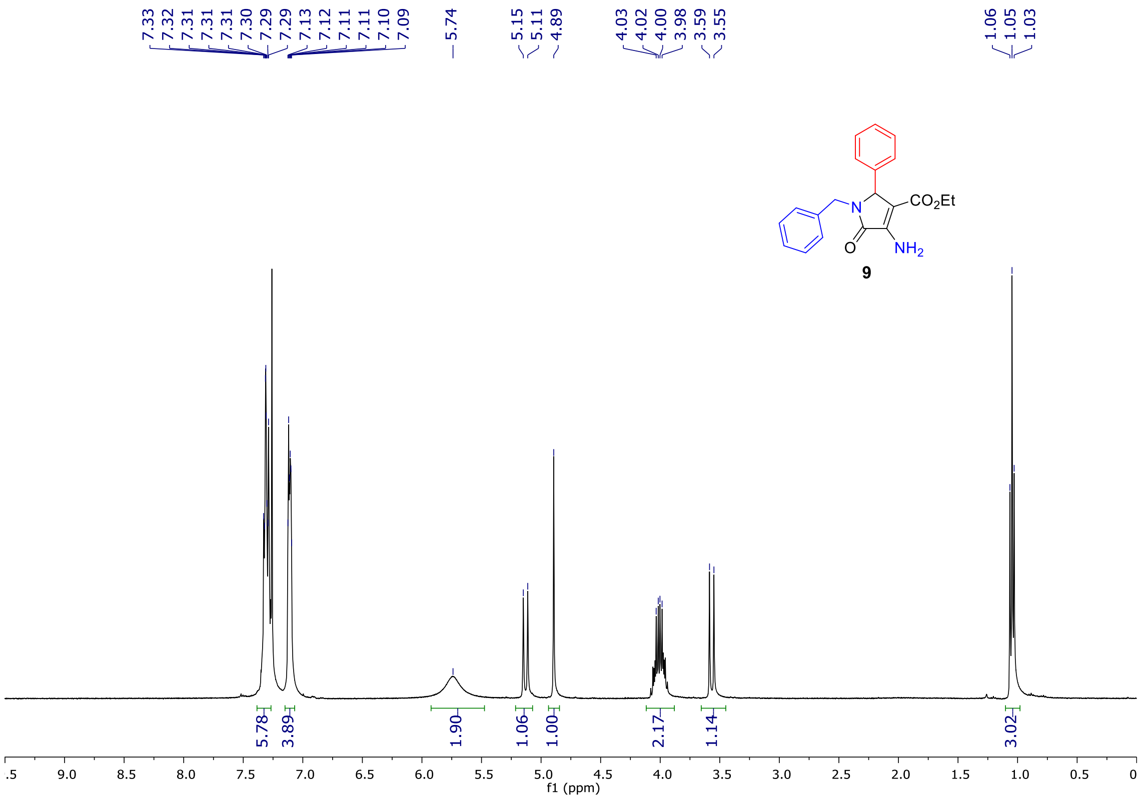

^{13}C {^{1}H} NMR (75 MHz, $CDCl_3$)

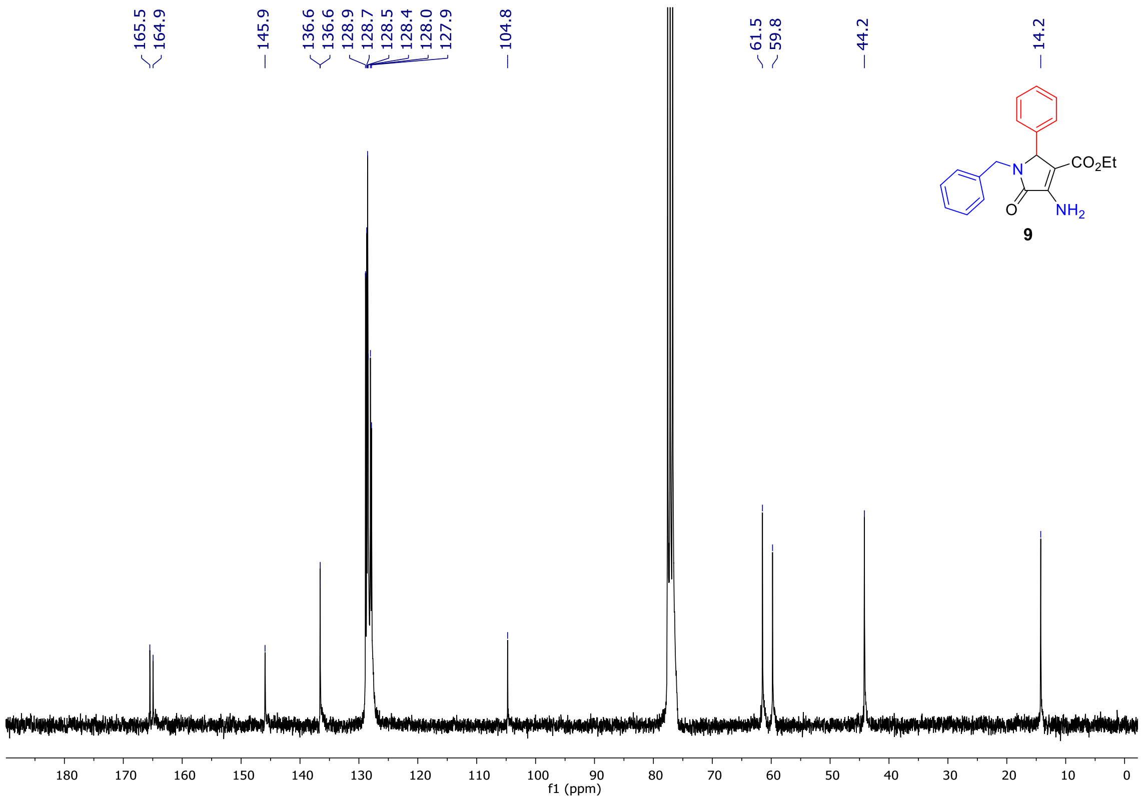

*Diethyl (4-hydroxy-5-oxo-2-phenyl-1-(p-tolyl)-2,5-dihydro-1H-pyrrol-3-yl)phosphonate (**12a**).*

^{1}H NMR (400 MHz, $CDCl_3$)

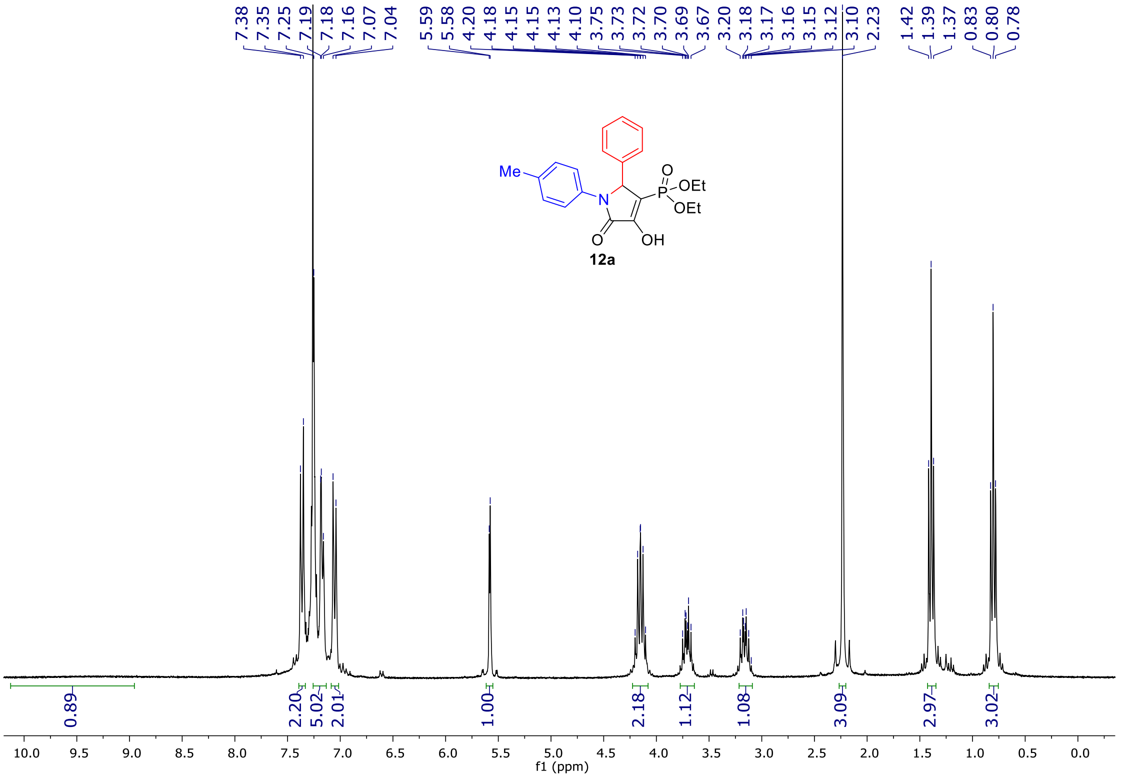

^{13}C {^{1}H} NMR (75 MHz, $CDCl_3$)

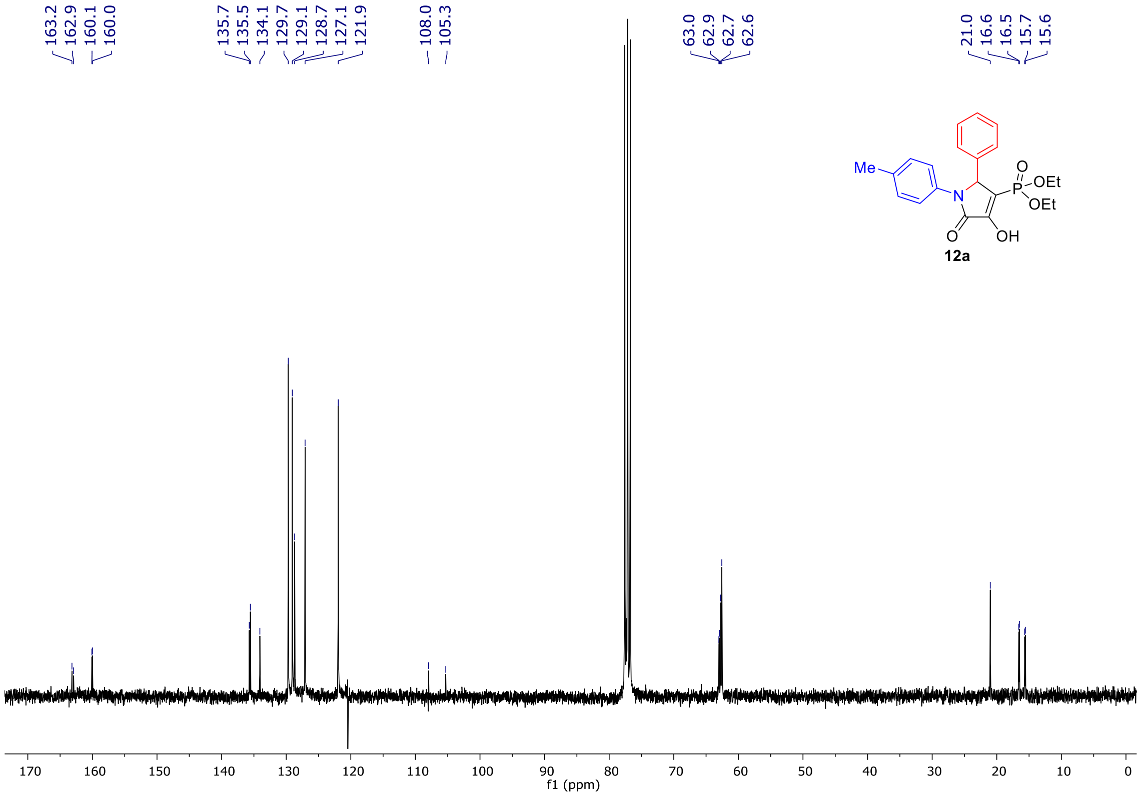

^{31}P NMR (121 MHz, $CDCl_3$)

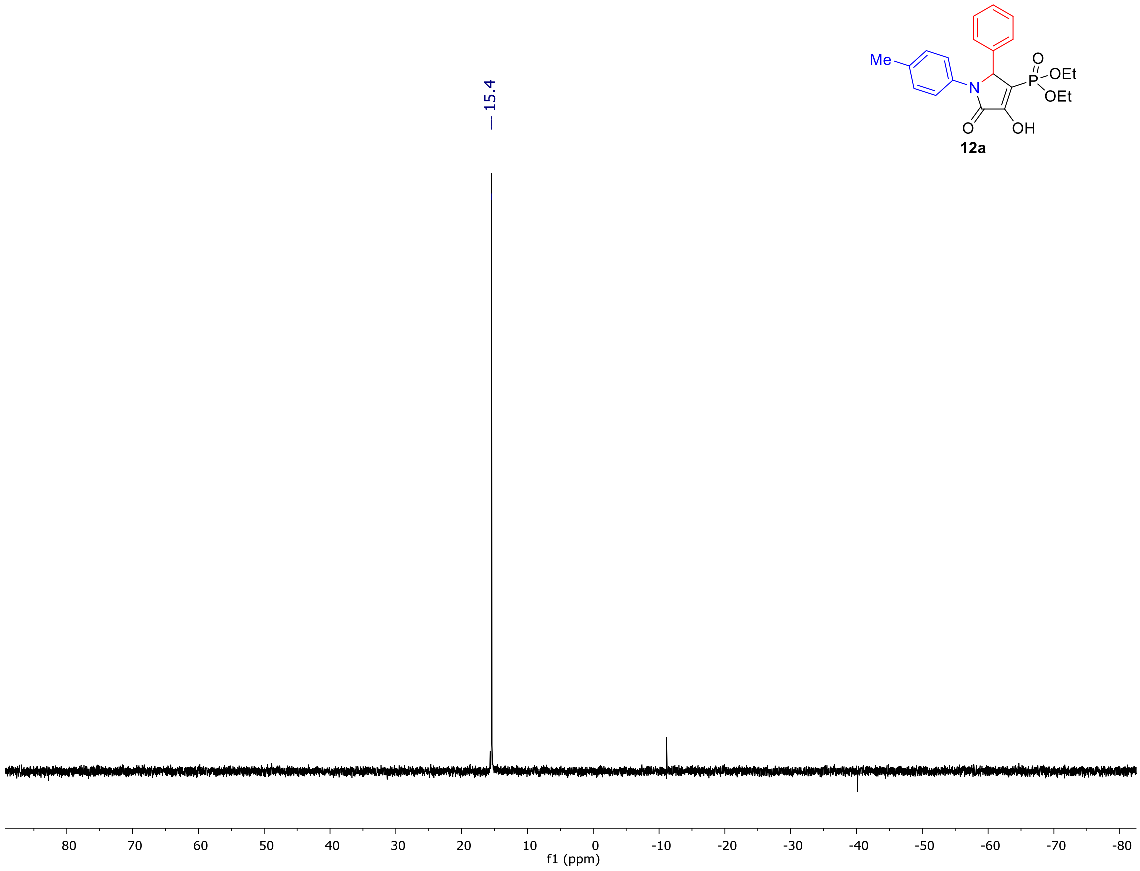

*Diethyl (4-hydroxy-1-(4-methoxyphenyl)-5-oxo-2-phenyl-2,5-dihydro-1H-pyrrol-3-yl)phosphonate (**12b**).*

^{1}H NMR (400 MHz, $CDCl_3$)

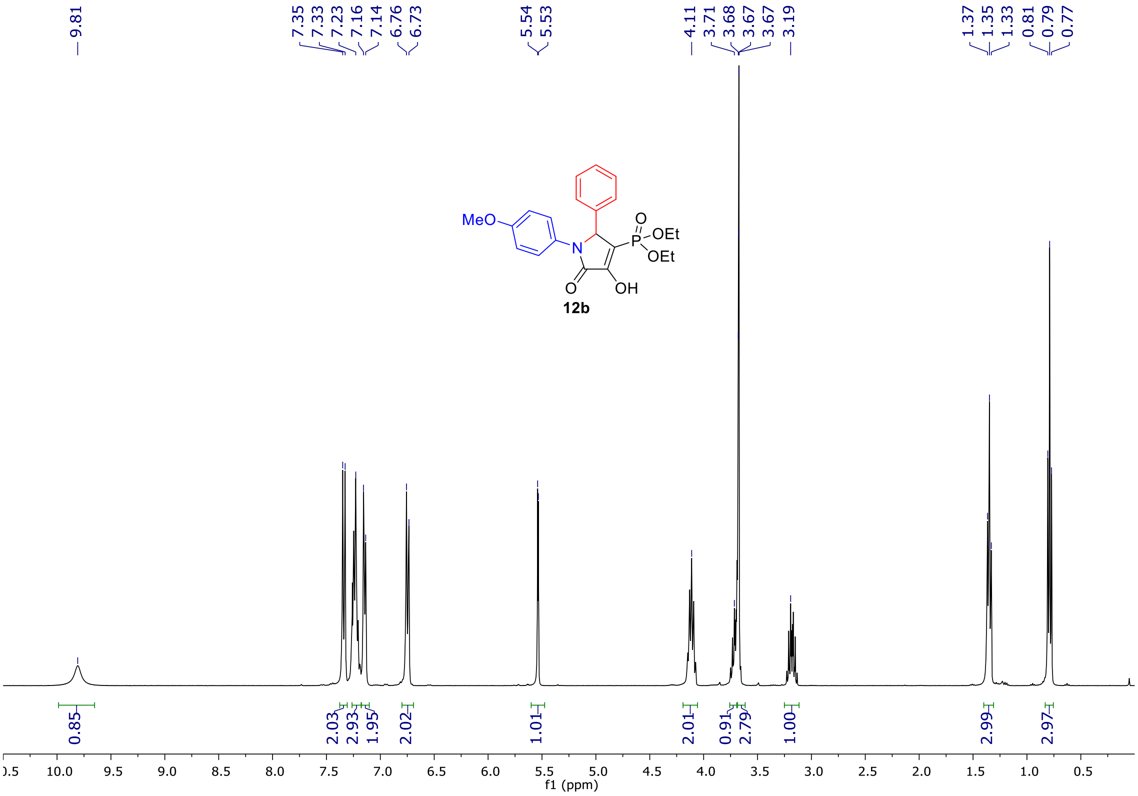

^{13}C {^{1}H} NMR (101 MHz, $CDCl_3$)

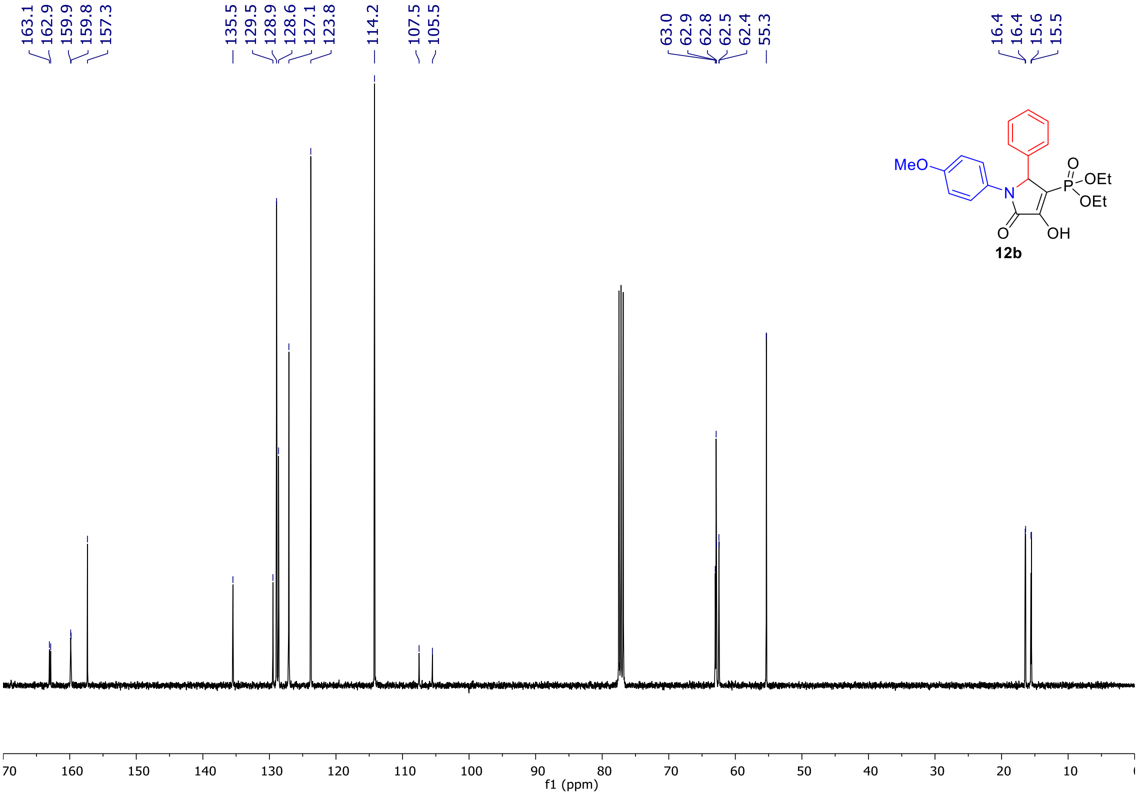

^{31}P NMR (162 MHz, $CDCl_3$)

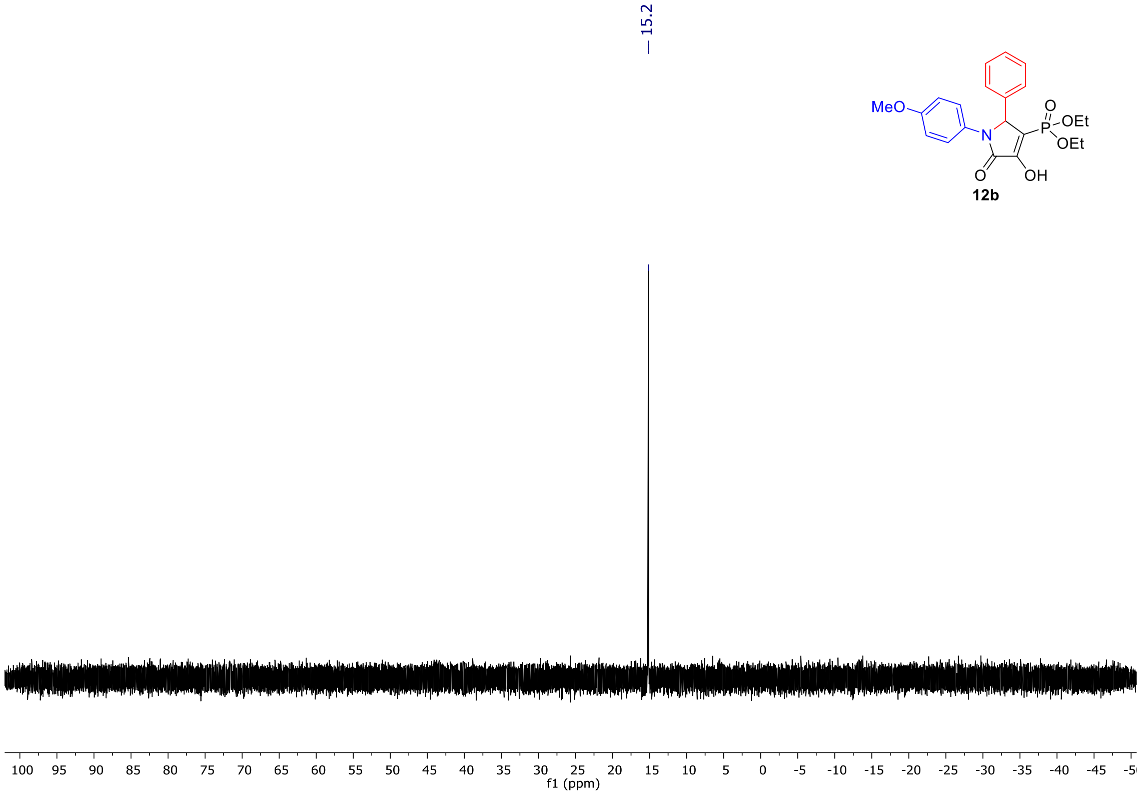

*Diethyl (1-(2-fluorophenyl)-4-hydroxy-5-oxo-2-phenyl-2,5-dihydro-1H-pyrrol-3-yl)phosphonate (**12c**).*

^{1}H NMR (400 MHz, $CDCl_3$)

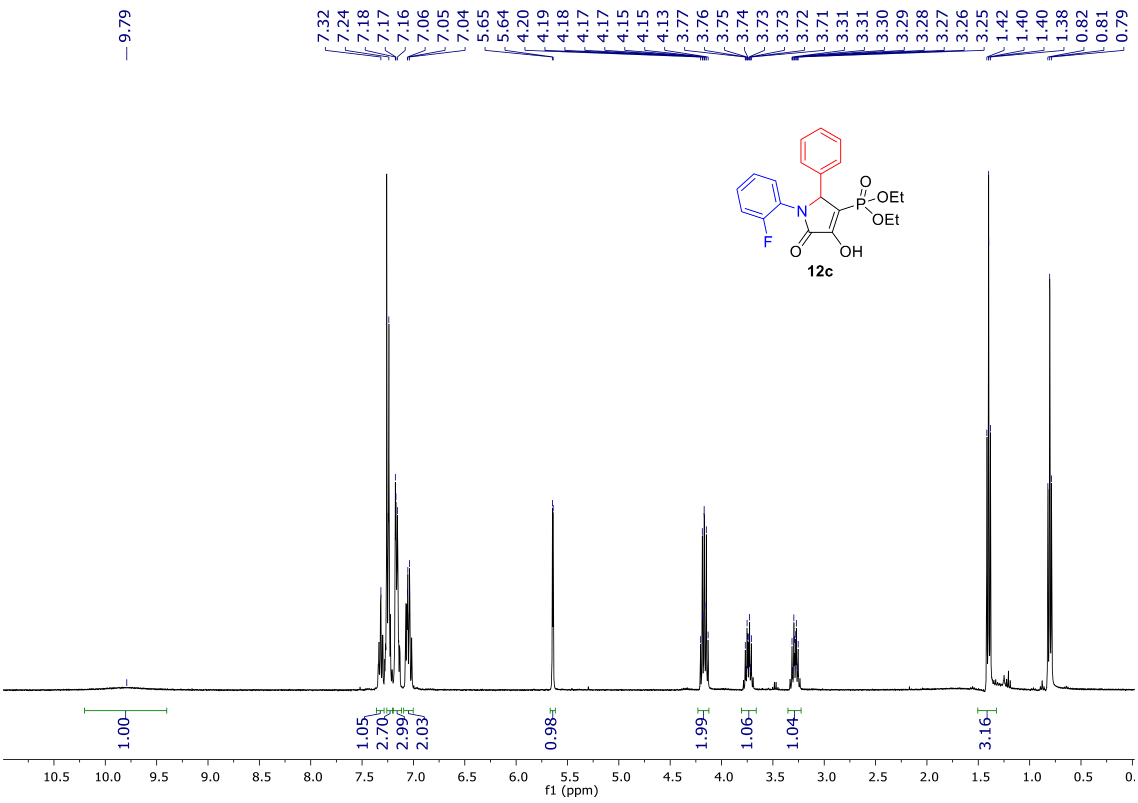

^{13}C {^{1}H} NMR (101 MHz, $CDCl_3$)

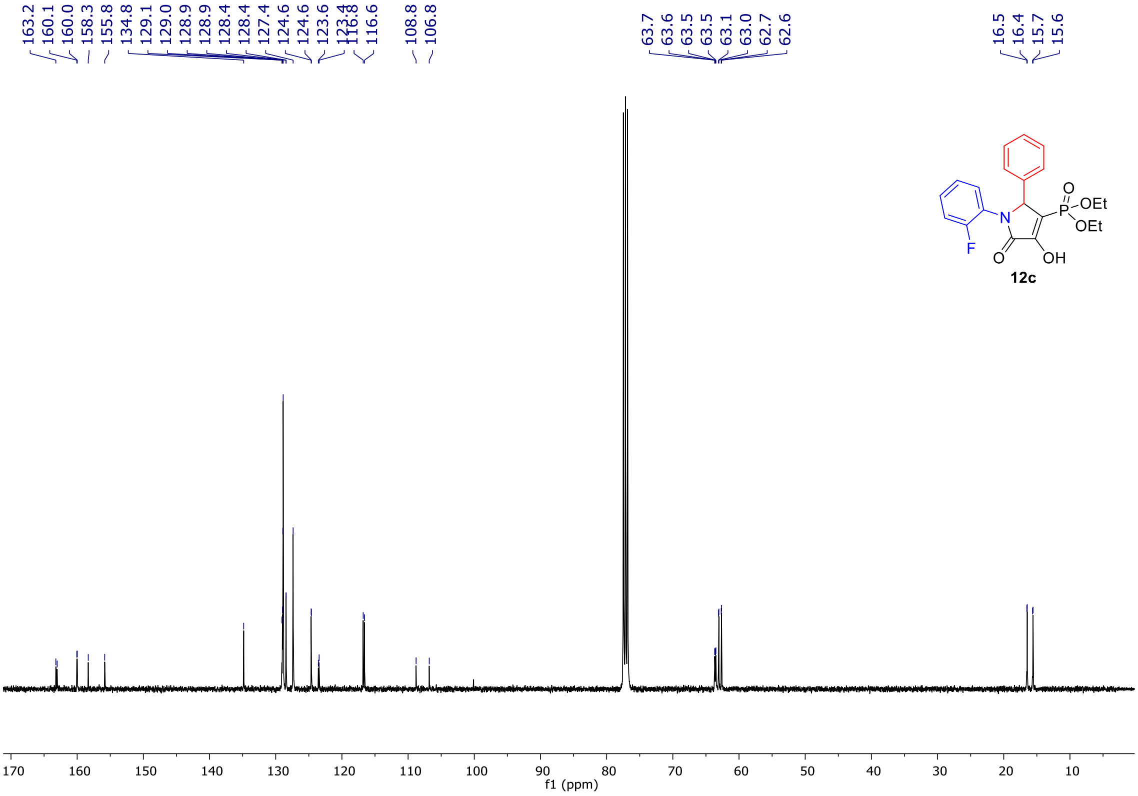

^{19}F NMR (282 MHz, $CDCl_3$)

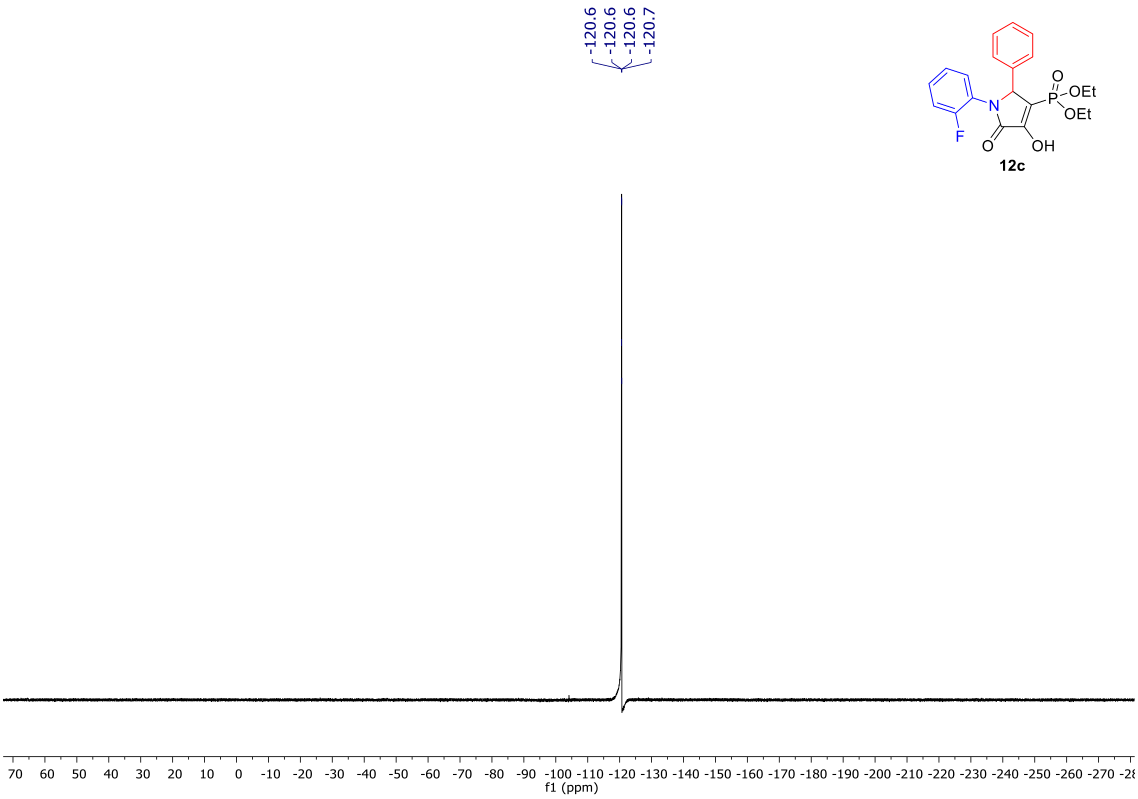

^{31}P NMR (121 MHz, $CDCl_3$)

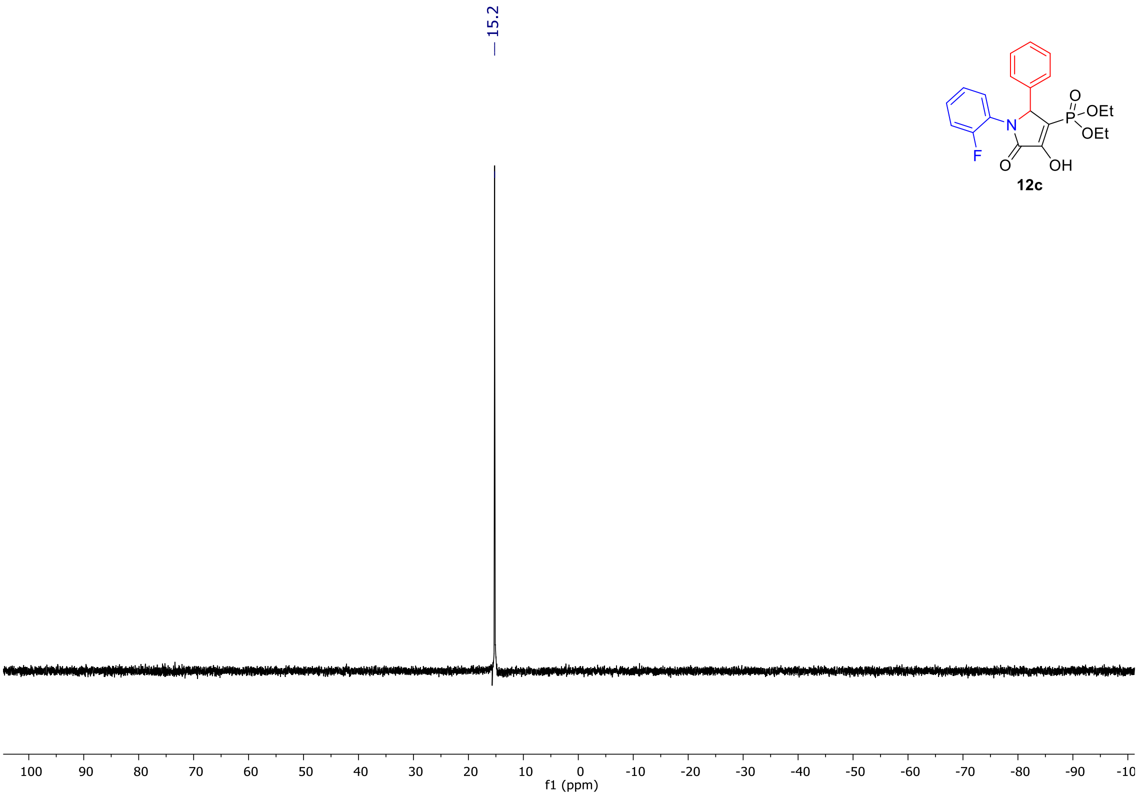

*Diethyl (4-hydroxy-2-(4-nitrophenyl)-5-oxo-1-(p-tolyl)-2,5-dihydro-1H-pyrrol-3-yl)phosphonate (**12d**).*

^{1}H NMR (400 MHz, DMSO-d_6, 60°C)

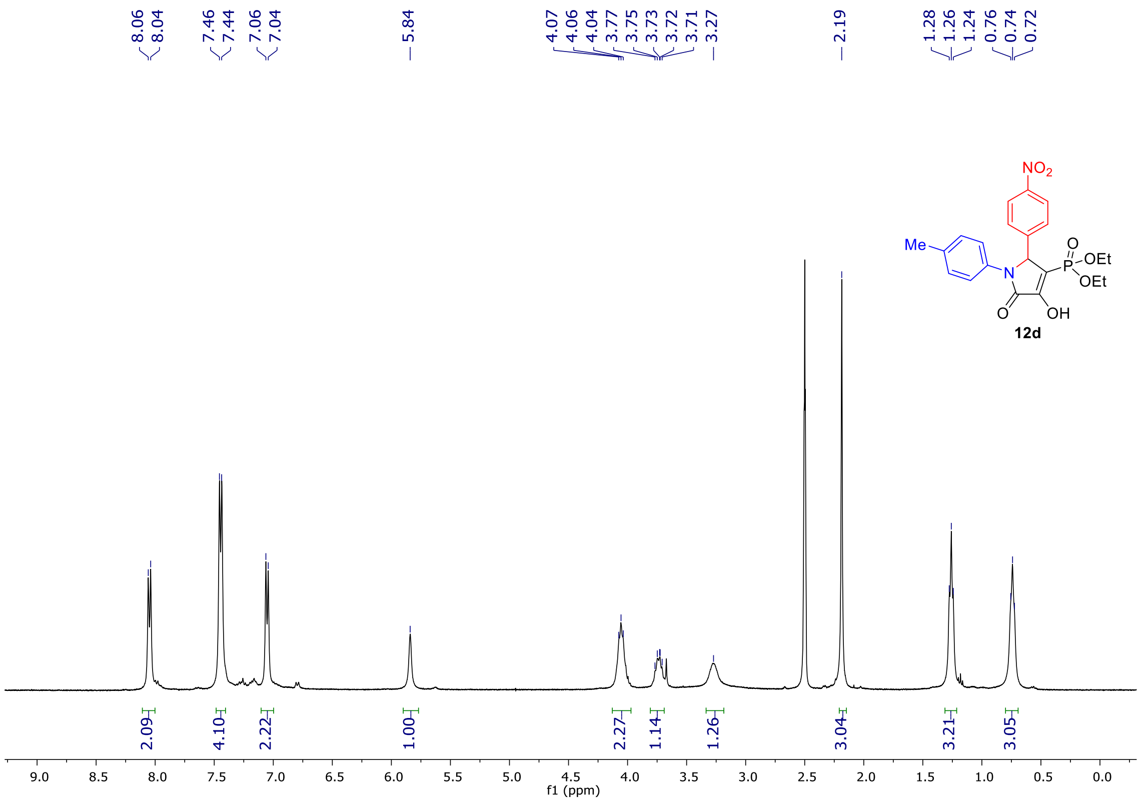

^{13}C NMR (101 MHz, DMSO-d_6, 60°C)

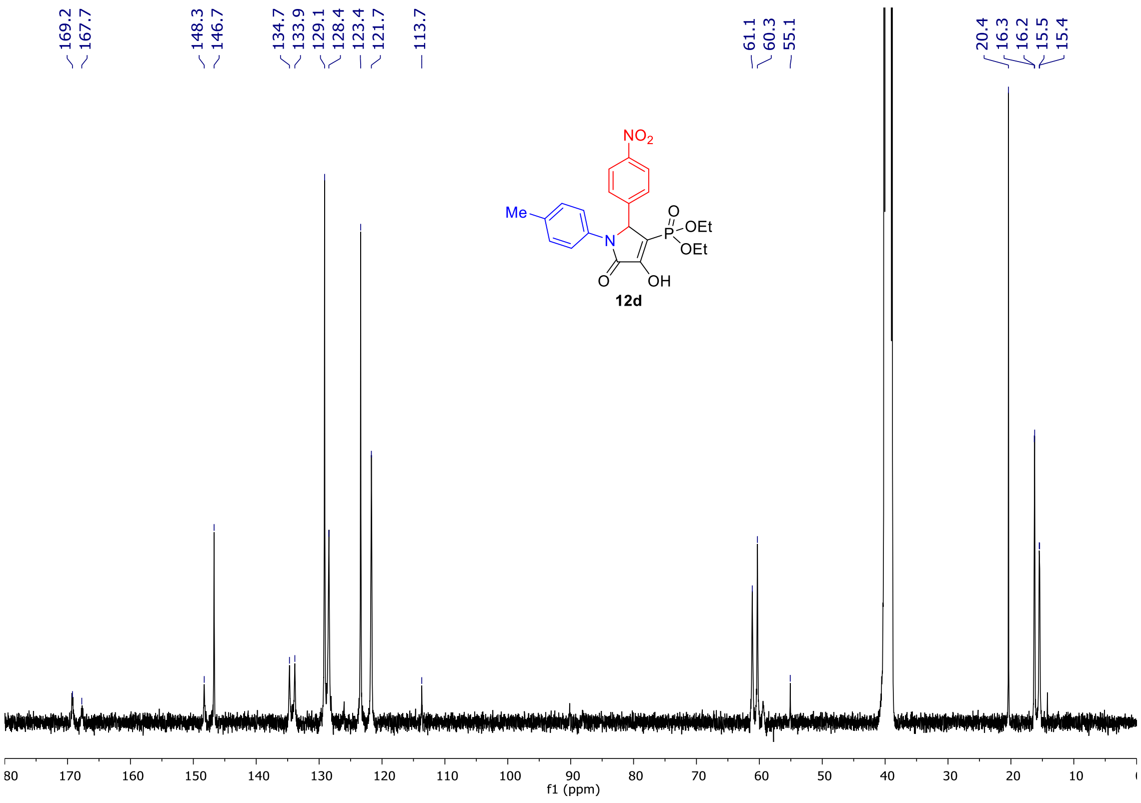

^{31}P NMR (162 MHz, DMSO-d_6, 60°C)

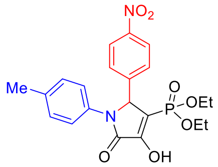

12d

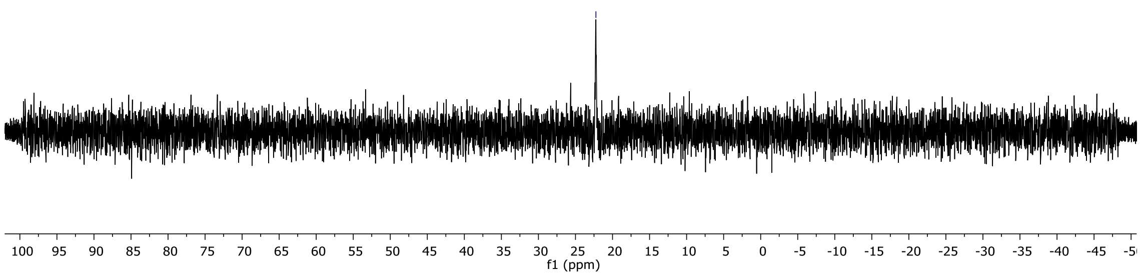

*Diethyl (2-(4-fluorophenyl)-4-hydroxy-5-oxo-1-(p-tolyl)-2,5-dihydro-1H-pyrrol-3-yl)phosphonate (**12e**).*

^{1}H NMR (400 MHz, DMSO-d_6, 70°C)

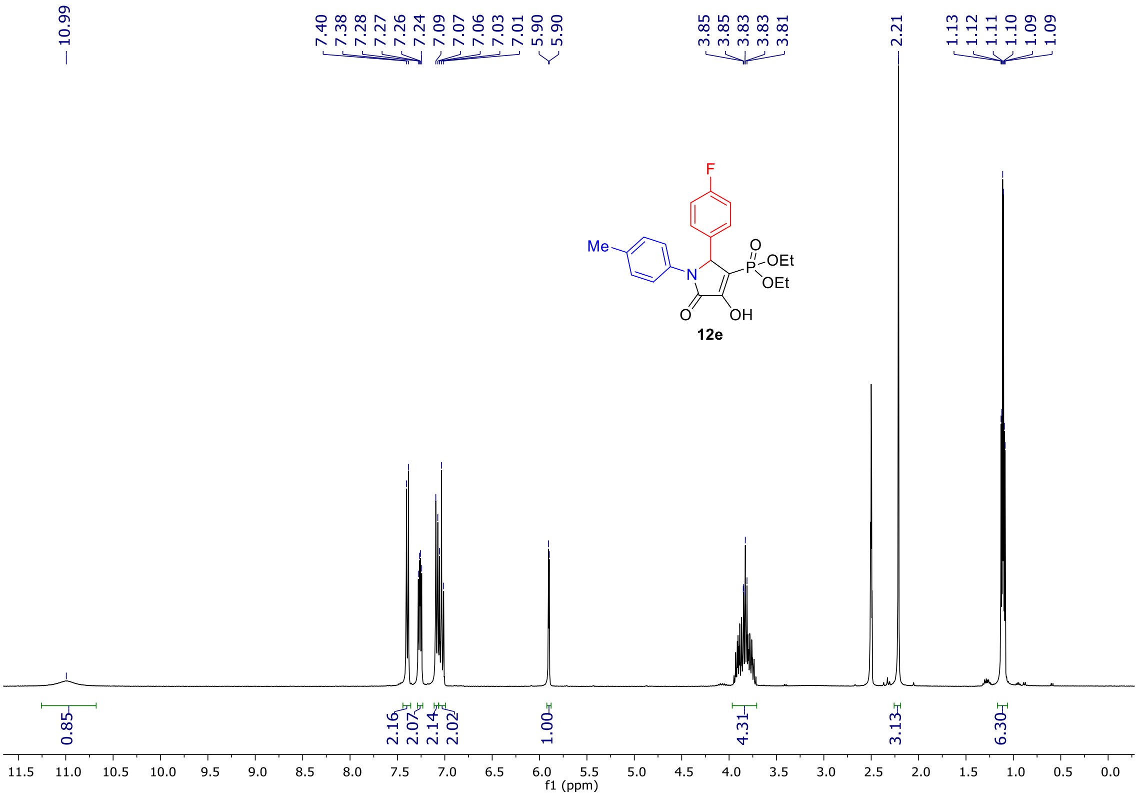

^{13}C {^{1}H} NMR (101 MHz, DMSO-d_6, 70°C)

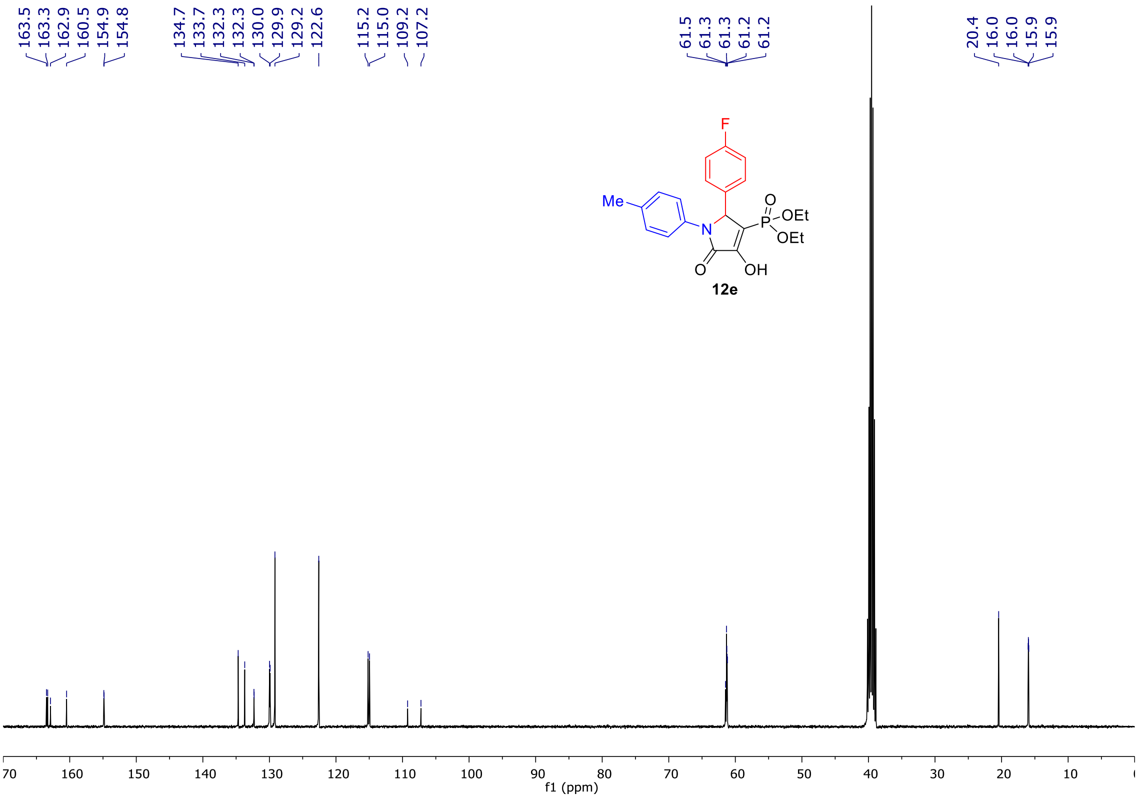

^{19}F NMR (282 MHz, DMSO-d_6, 70°C)

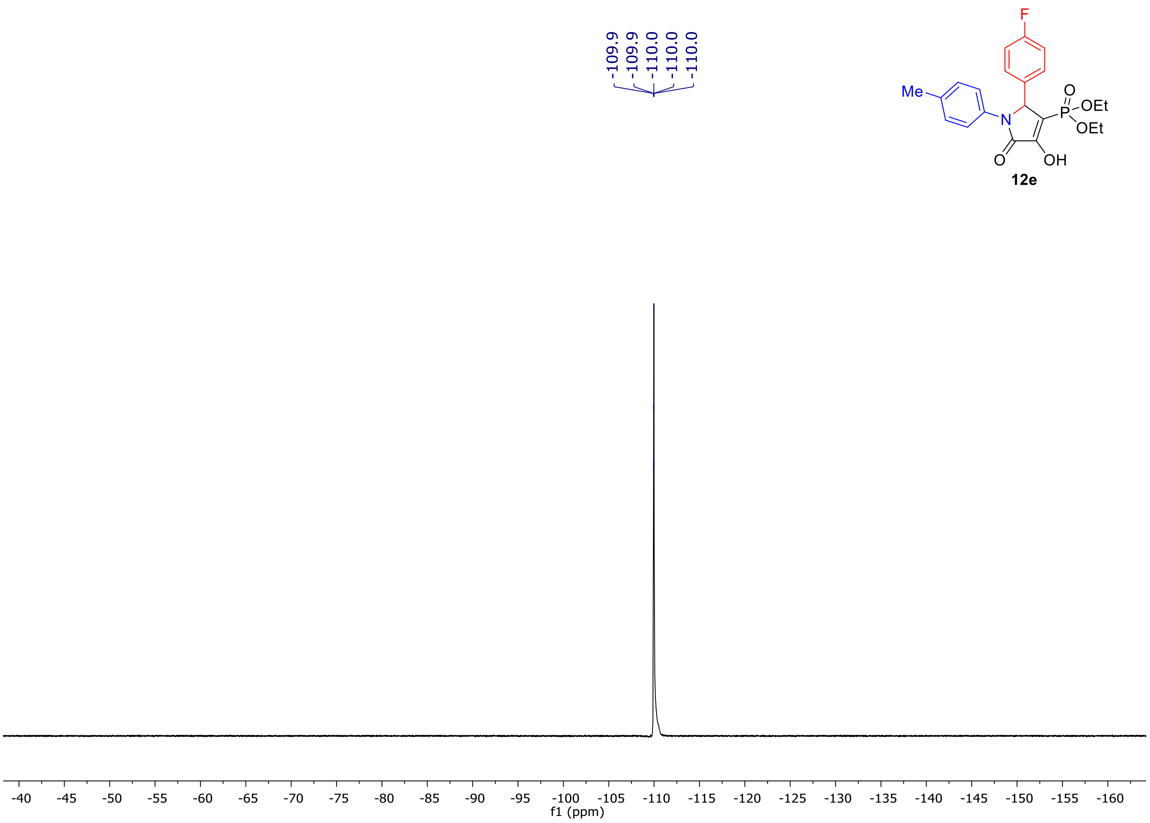

^{31}P NMR (162 MHz, DMSO-d_6, 70°C)

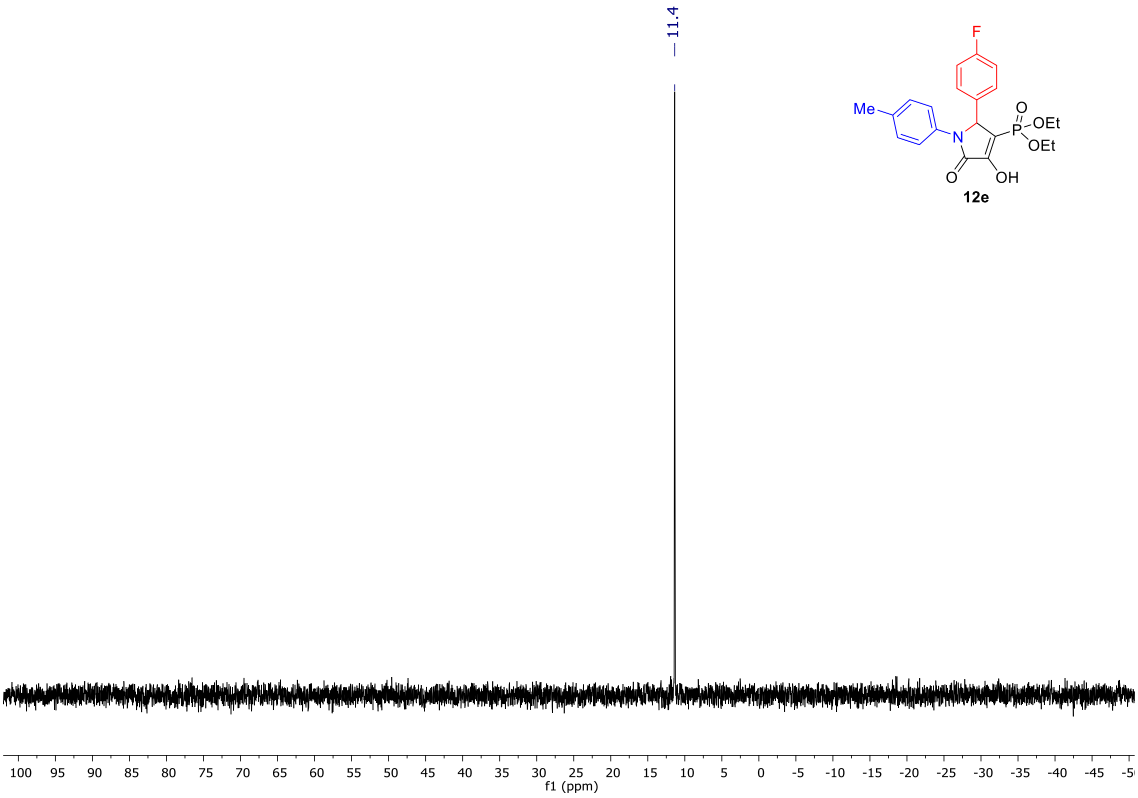

*Diethyl (4-hydroxy-5-oxo-2-(thiophen-2-yl)-1-(p-tolyl)-2,5-dihydro-1H-pyrrol-3-yl)phosphonate (**12f**).*

^{1}H NMR (400 MHz, $CDCl_3$)

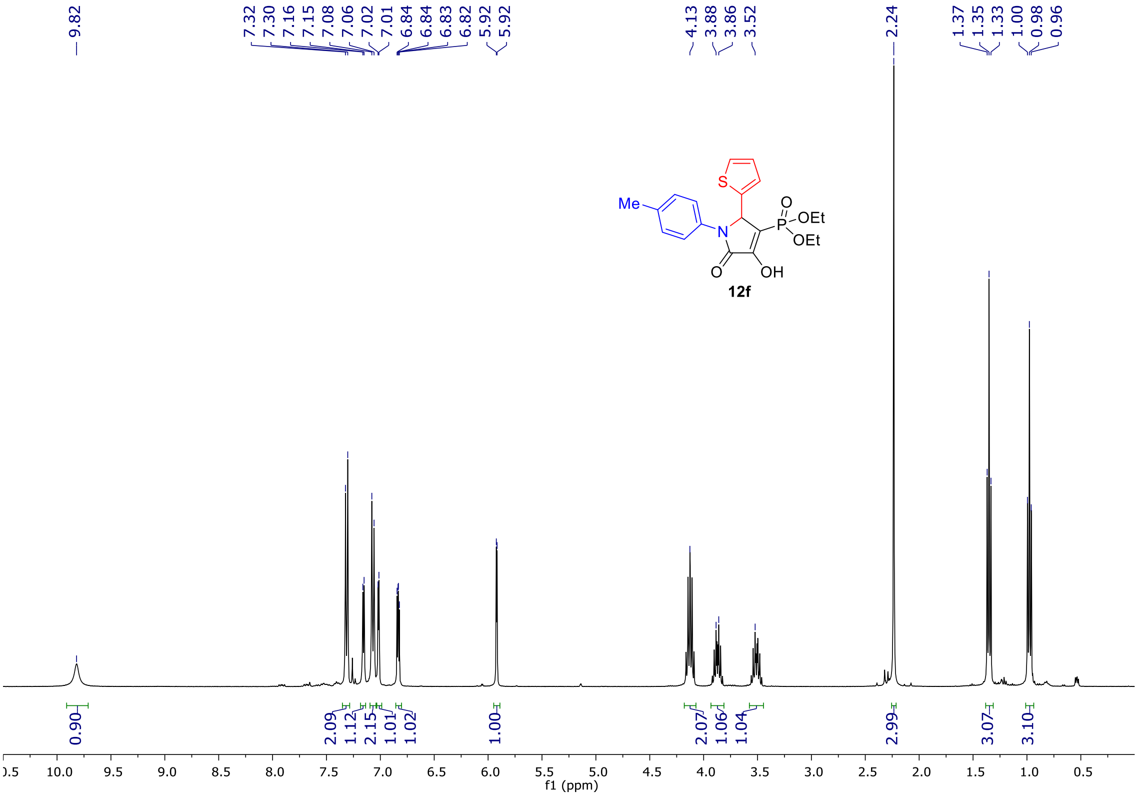

^{13}C {^{1}H} NMR (101 MHz, $CDCl_3$)

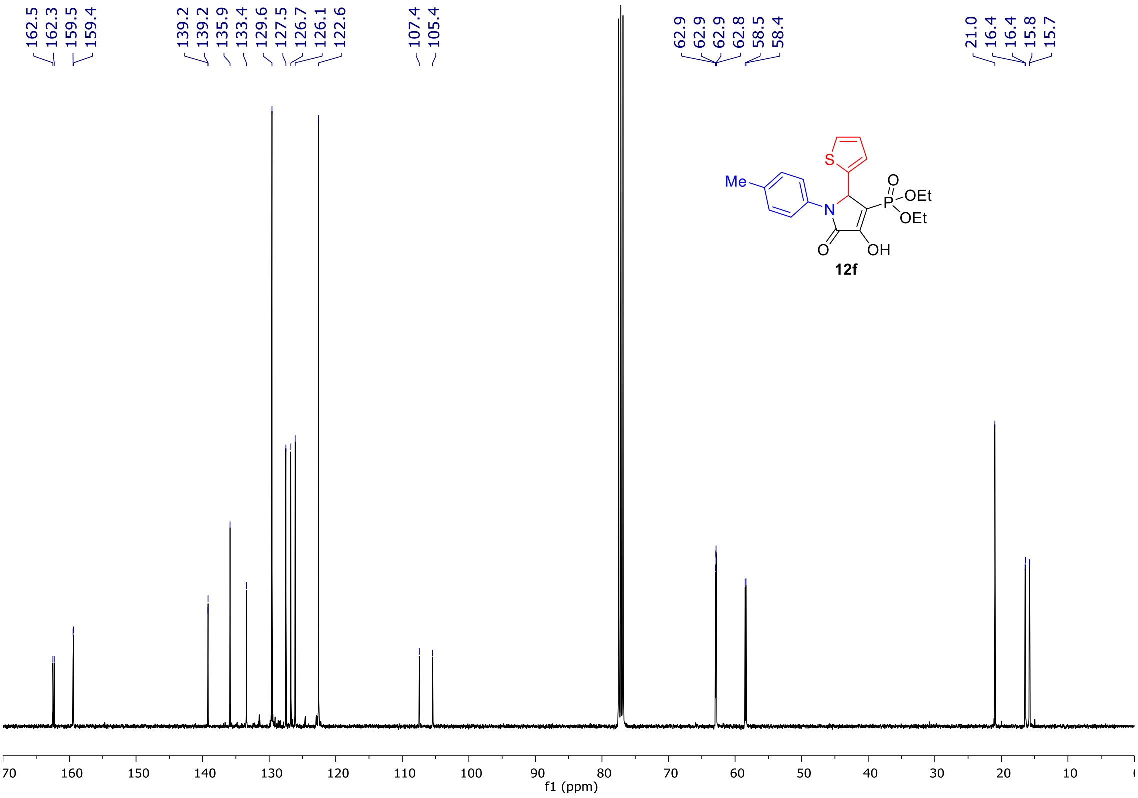

^{31}P NMR (162 MHz, $CDCl_3$)

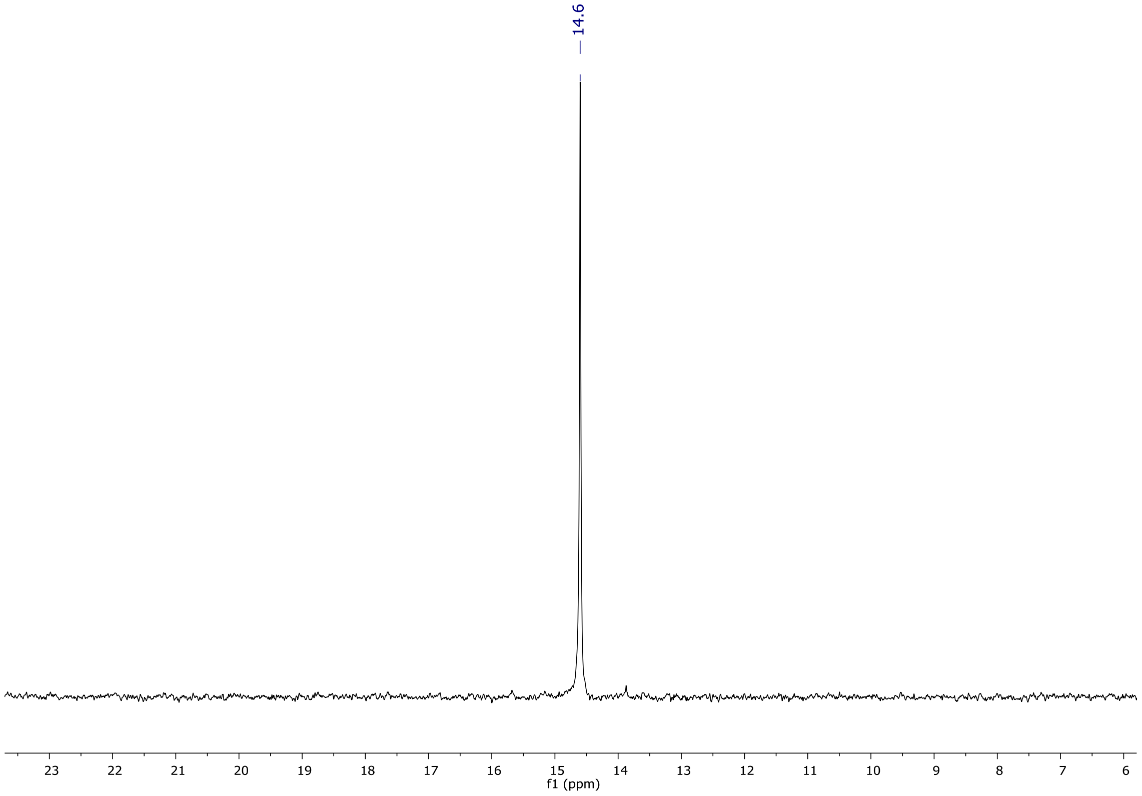

*Ethyl 3-(diethoxyphosphoryl)-4-hydrxy-5-oxo-1-(p-tolyl)-2,5-dihydro-1H-pyrrole-2-carboxylate (**12g**).*

^{1}H NMR (400 MHz, DMSO-d_6, 60°C)

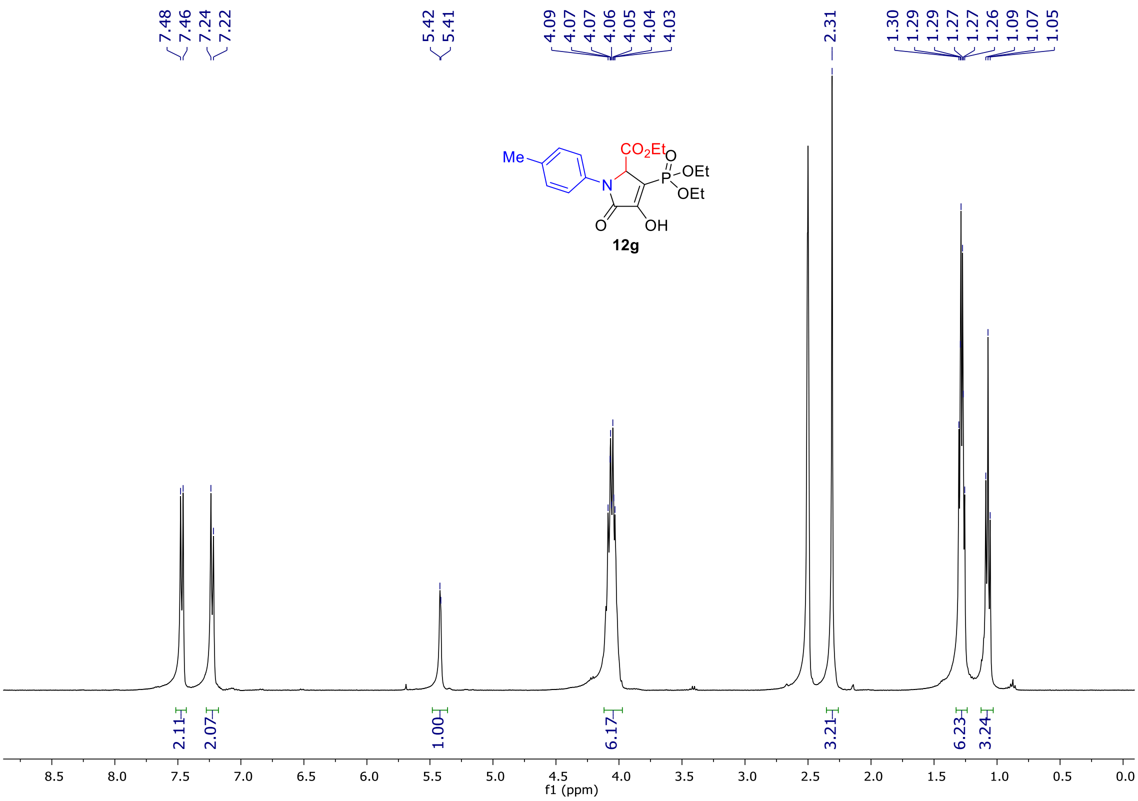

^{13}C {^{1}H} NMR (101 MHz, DMSO-d_6, 60°C)

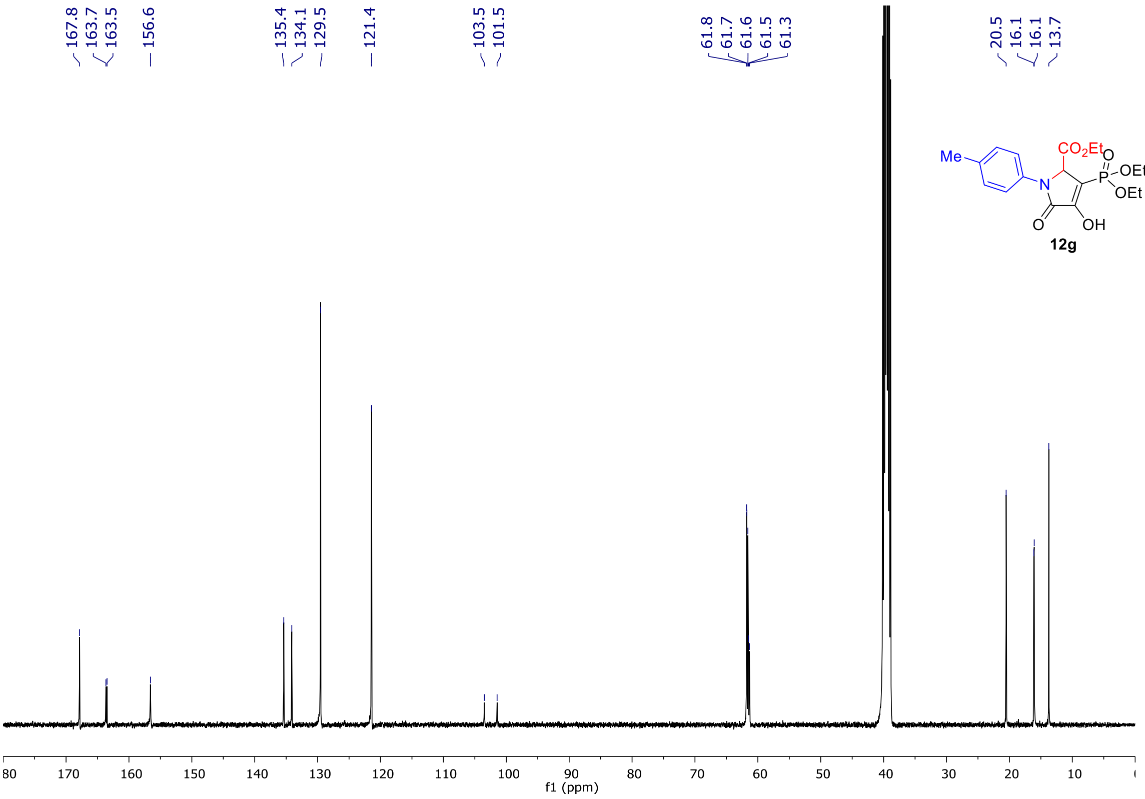

^{31}P NMR (162 MHz, DMSO-d_6, 60°C)

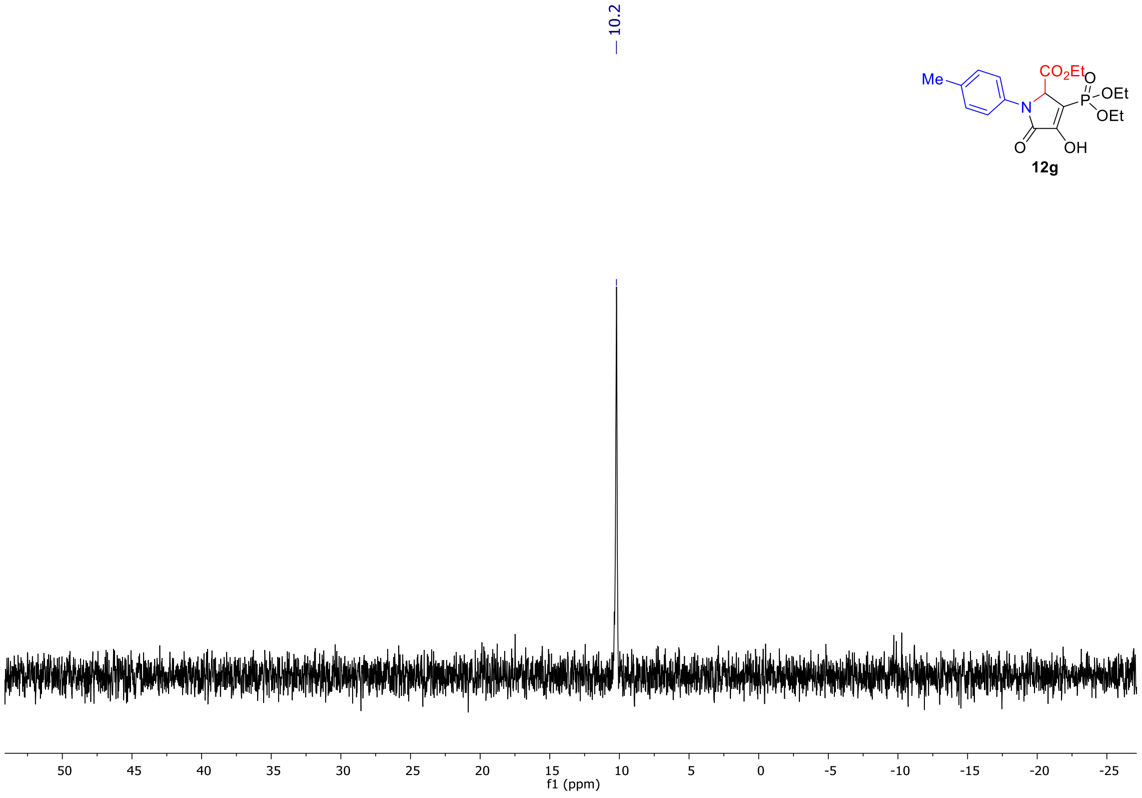

*Diethyl (4-hydroxy-2-isopropyl-5-oxo--(p-tolyl)-2,5-dihydro-1H-pyrrol-3-yl)phosphonate (**12h**).*

^{1}H NMR (400 MHz, $CDCl_3$)

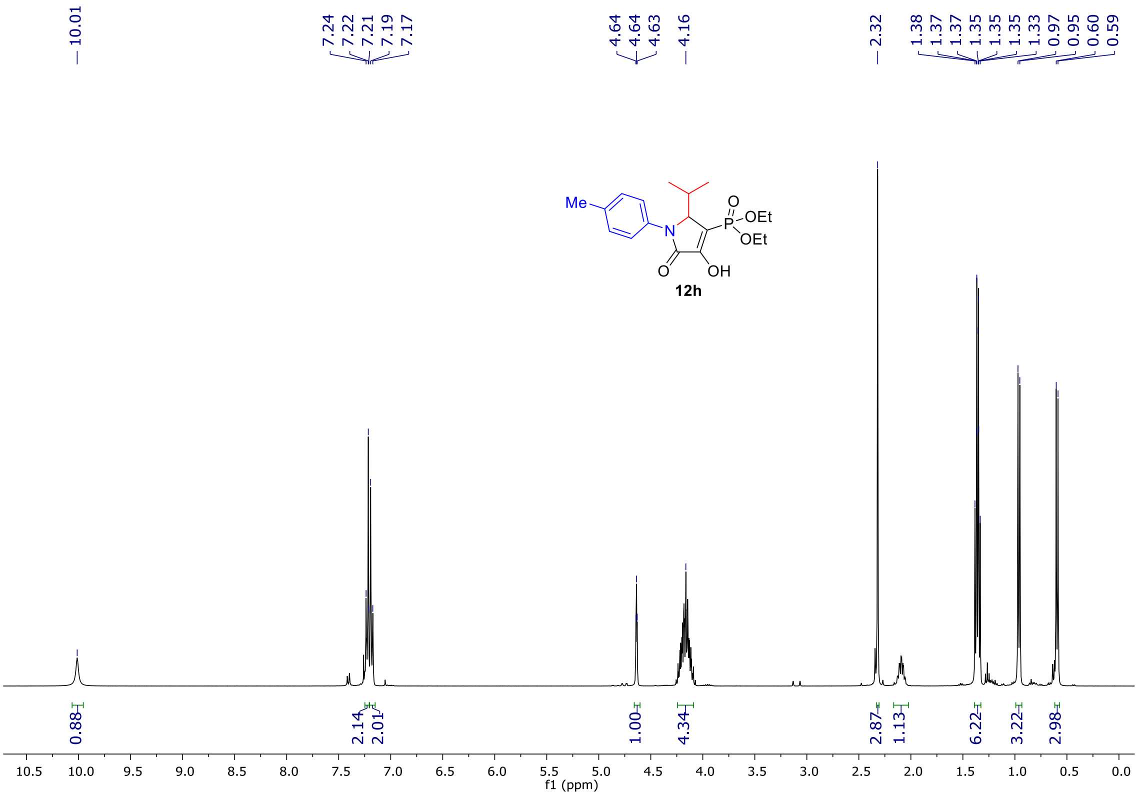

^{13}C {^{1}H} NMR (101 MHz, $CDCl_3$)

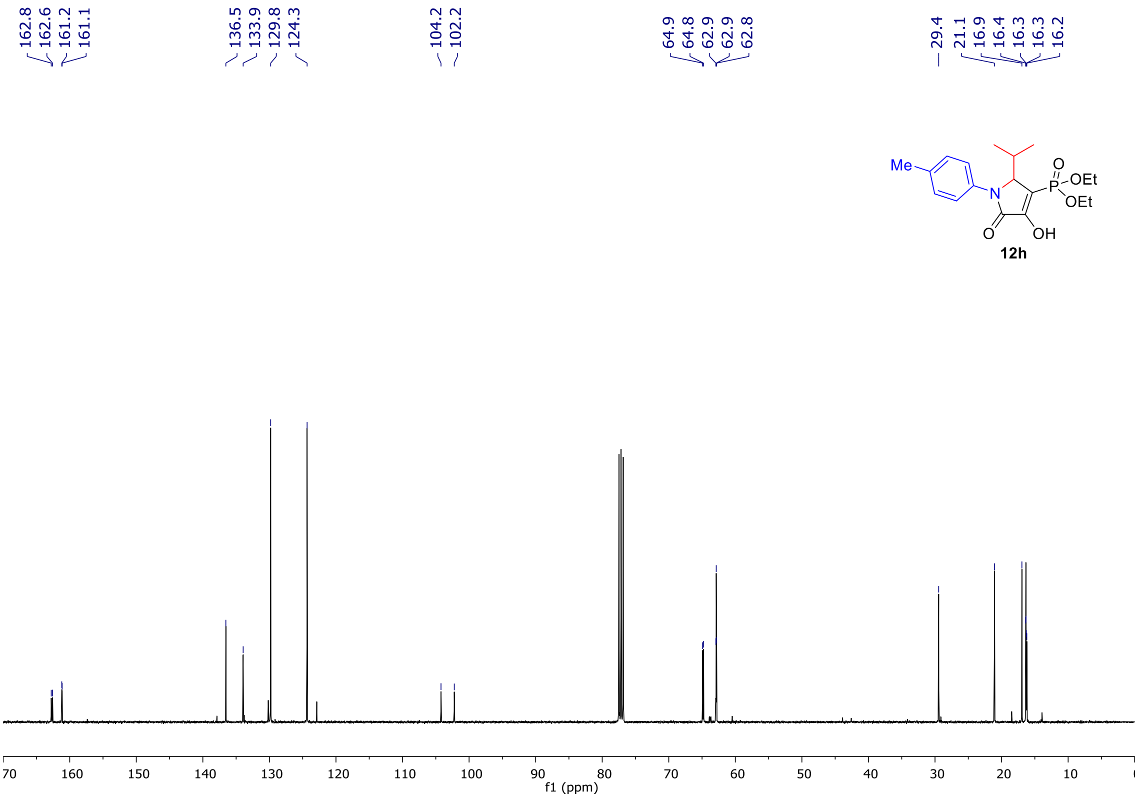

^{31}P NMR (162 MHz, $CDCl_3$)

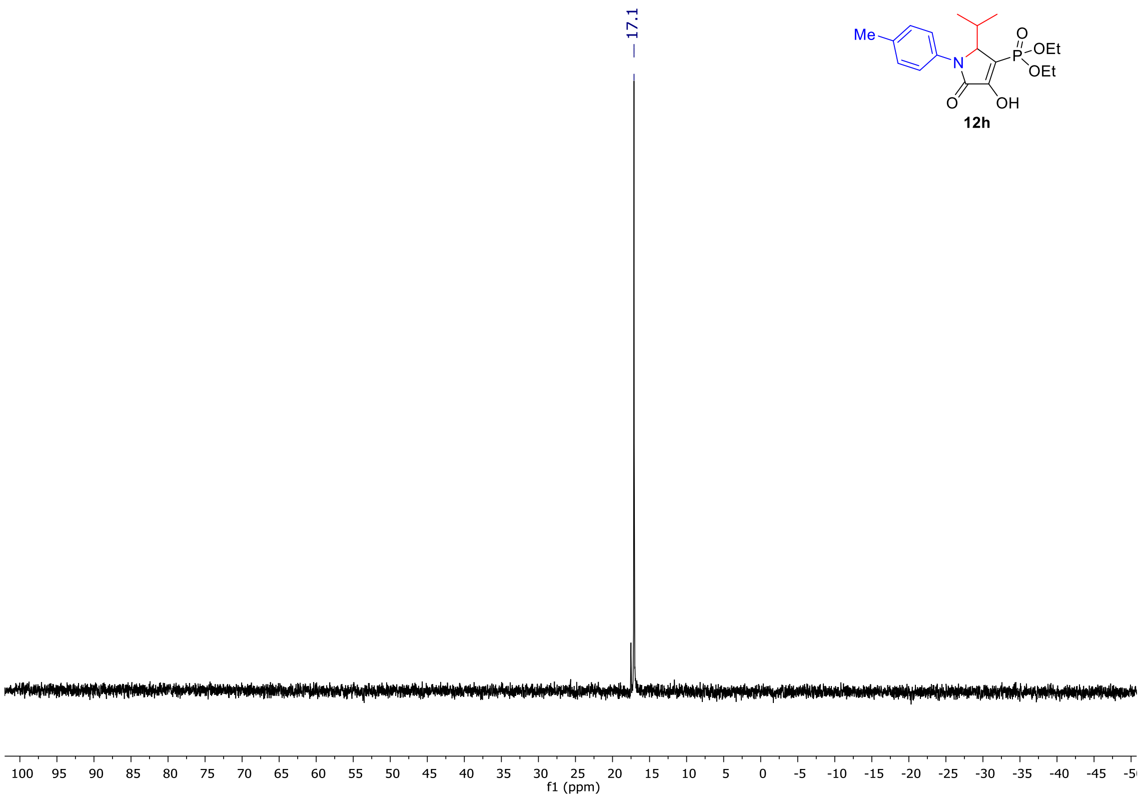

*Diisopropyl (4-hydroxy-5-oxo-2-penyl-1-(p-tolyl)-2,5-dihydro-1H-pyrrol-3-yl)phosphonate (**12i**).*

¹H NMR (400 MHz, CDCl₃)

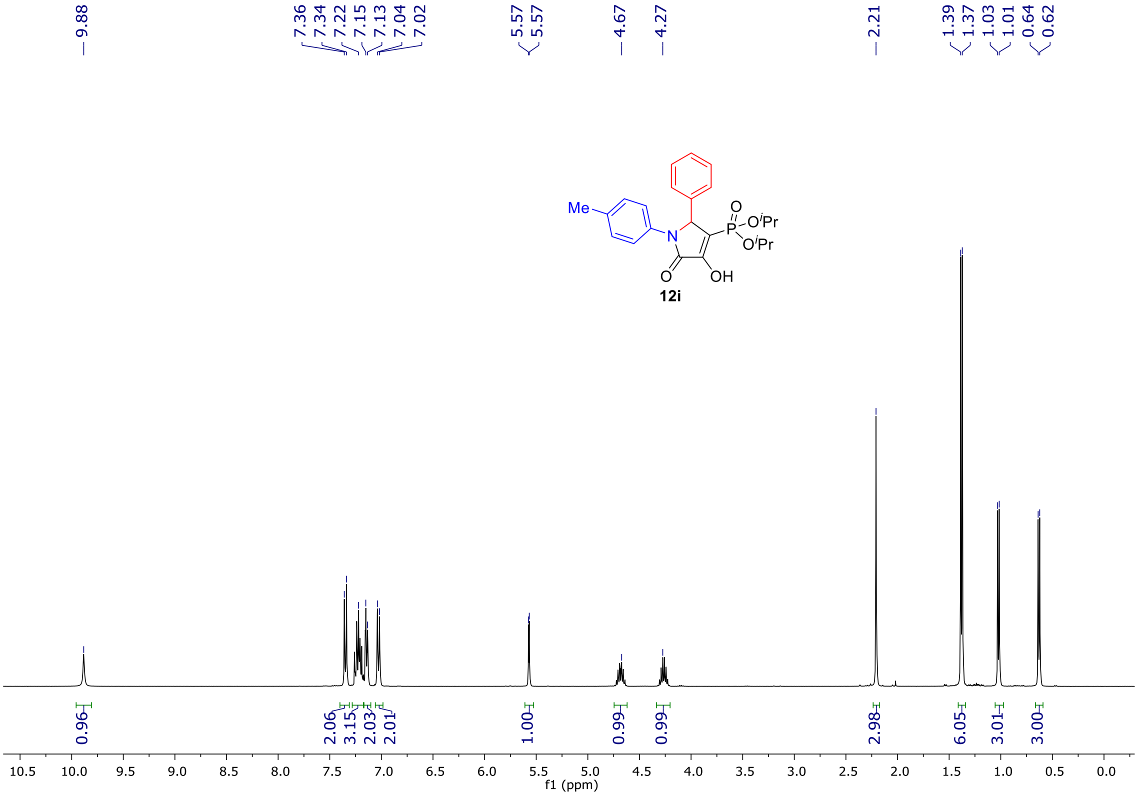

¹³C {¹H} NMR (101 MHz, CDCl₃)

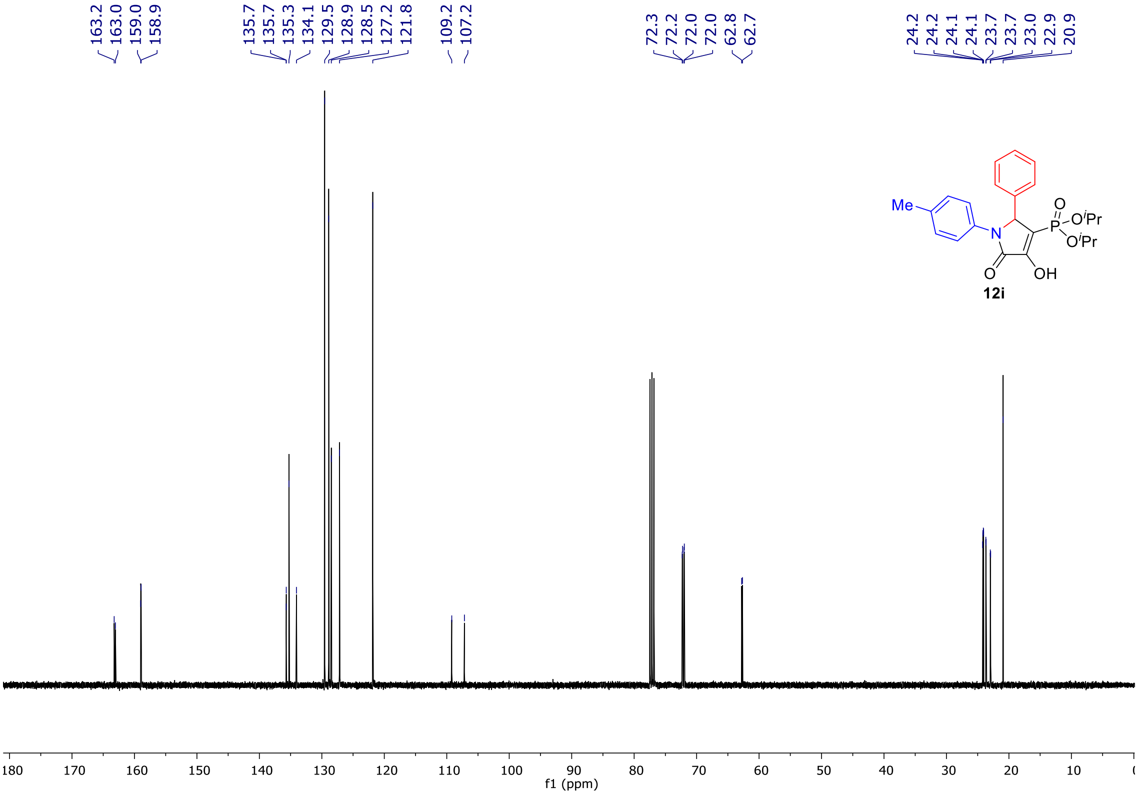

^{31}P NMR (162 MHz, $CDCl_3$)

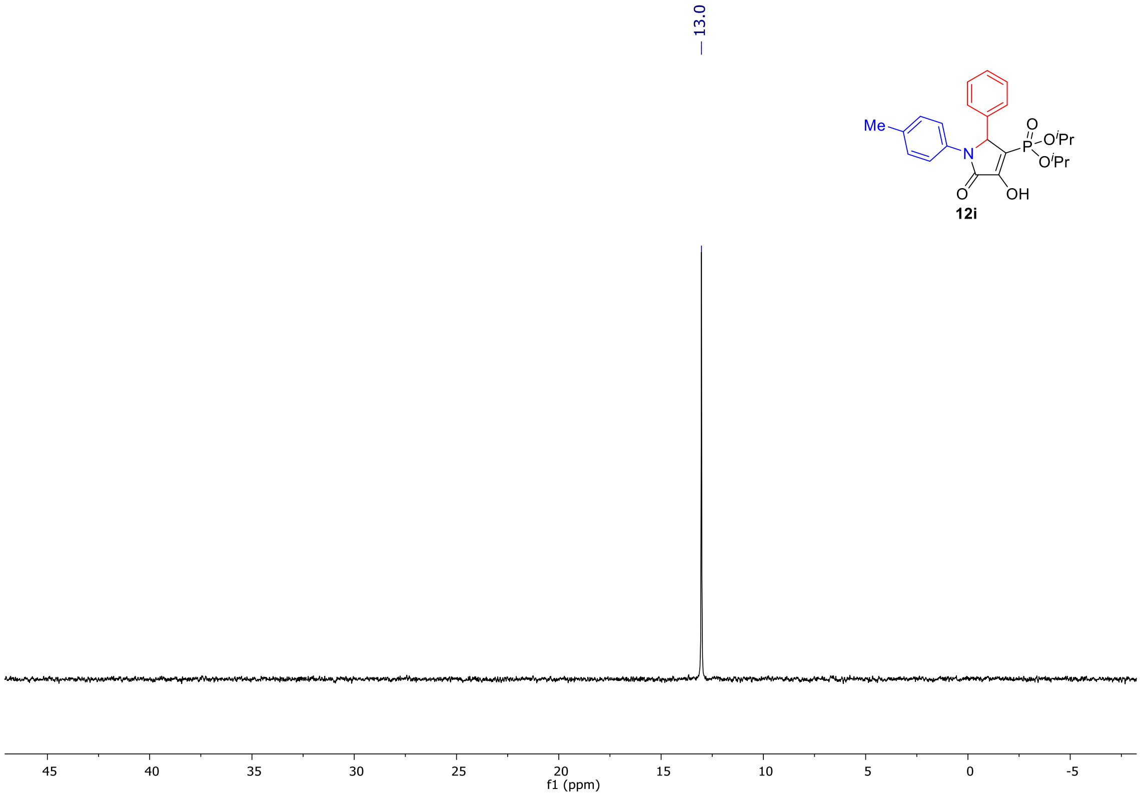

*Diisopropyl (2-(4-fluorophenyl)-4-hydroxy-5-oxo-1-(p-tolyl)-2,5-dihydro-1H-pyrrol-3-yl)phosphonatephosphonate (**12j**).*

¹H NMR (400 MHz, DMSO-d_6)

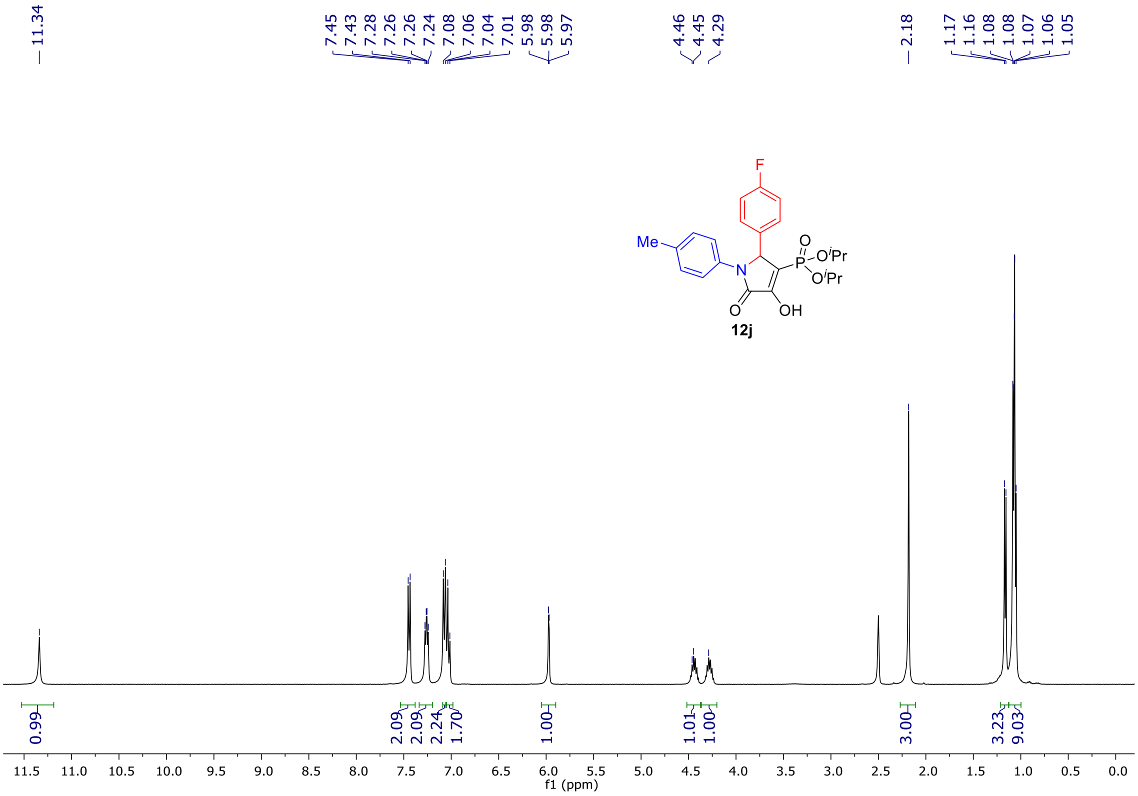

¹³C {¹H} NMR (101 MHz, DMSO-d_6)

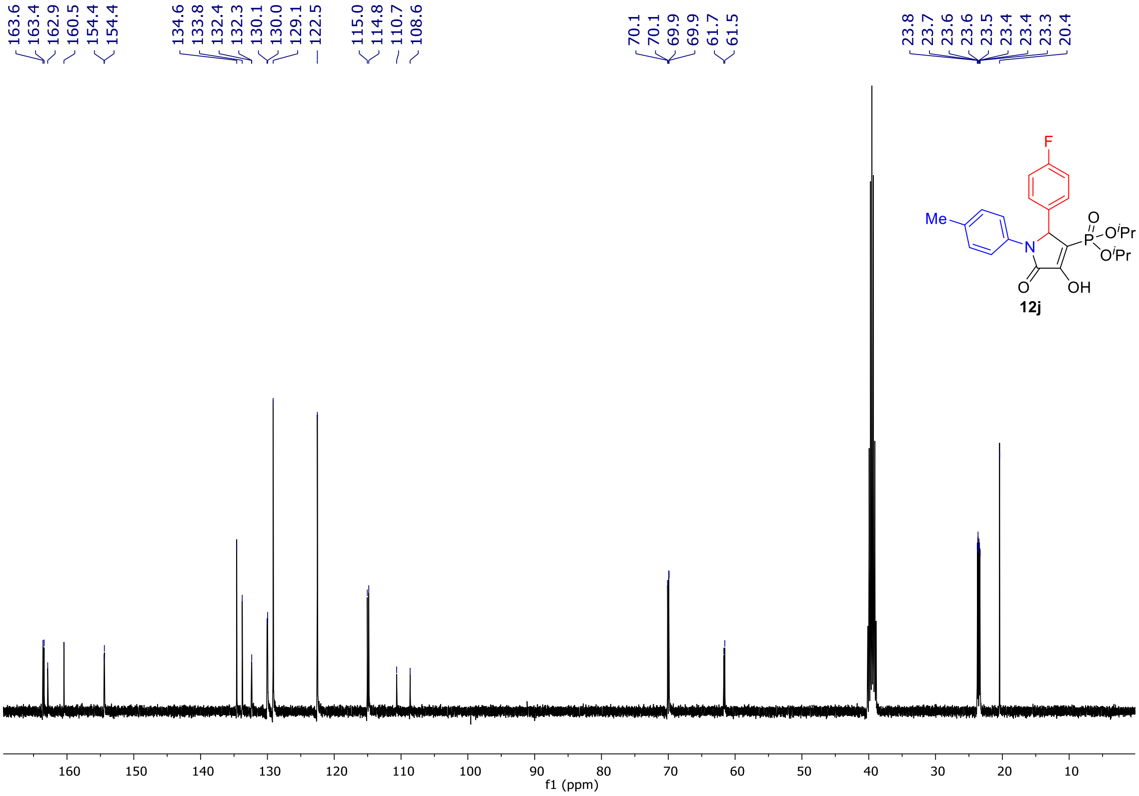

^{19}F NMR (282 MHz, $CDCl_3$)

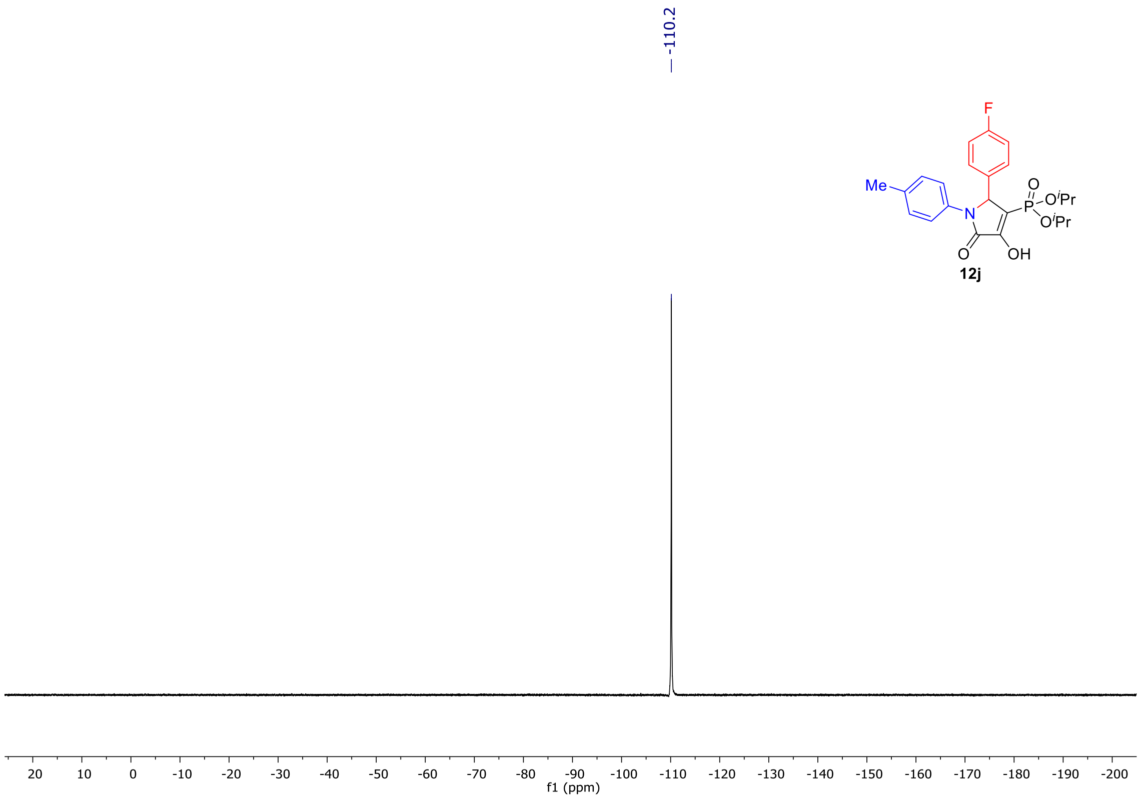

^{31}P NMR (162 MHz, DMSO-d_6)

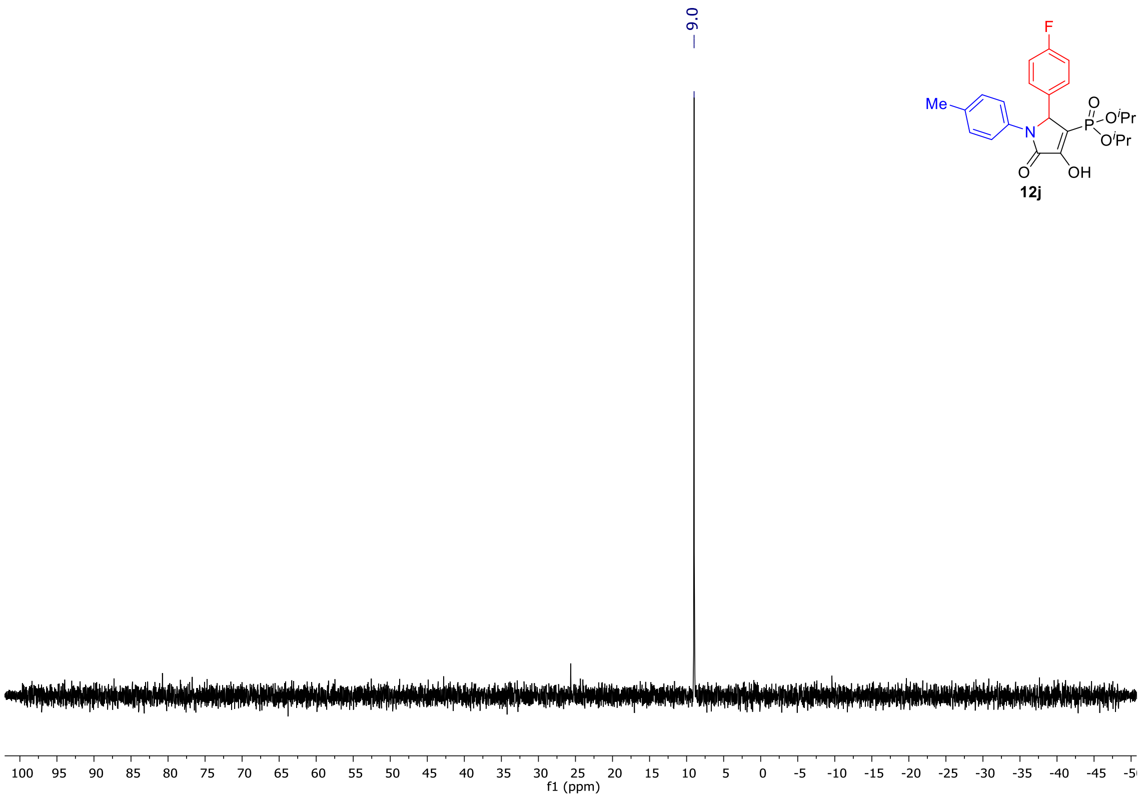

*Ethyl 3-(diisopropoxyphosphorl)-4-hydroxy-5-oxo-1-(p-tolyl)-2,5-dihydro-1H-pyrrole-2-carboxylate (**12k**).*

¹H NMR (400 MHz, $CDCl_3$)

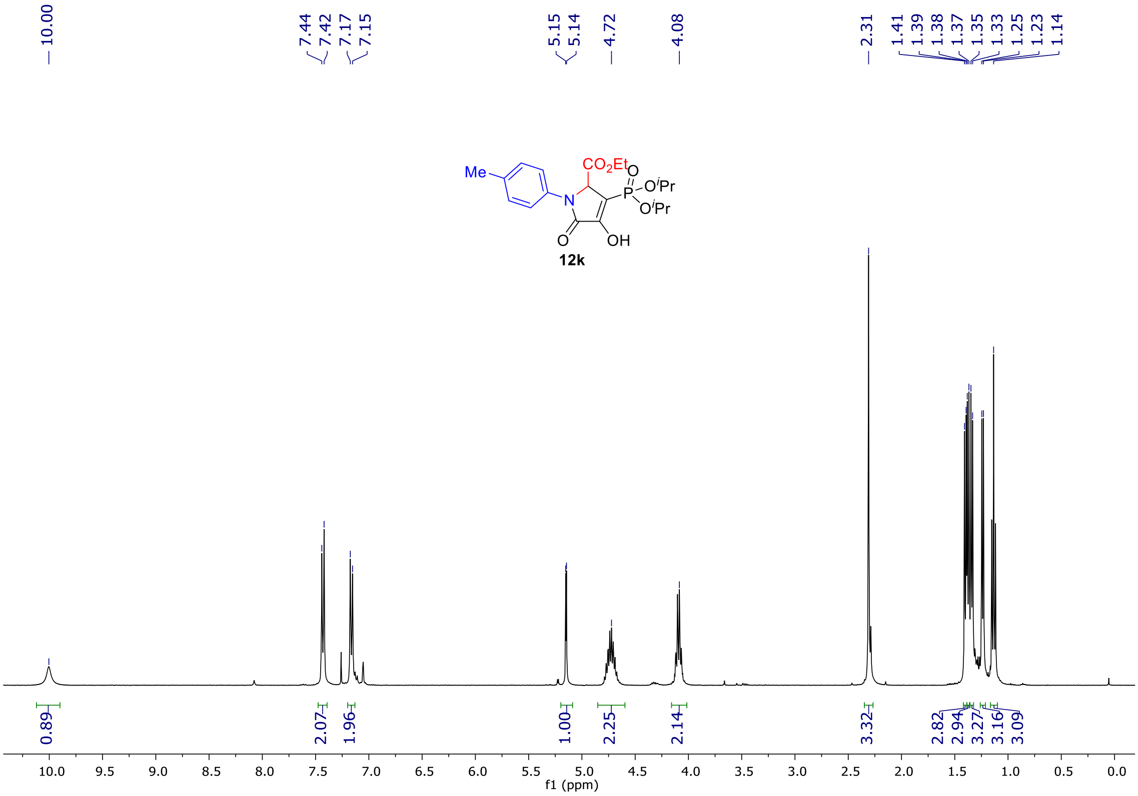

¹³C NMR (101 MHz, $CDCl_3$)

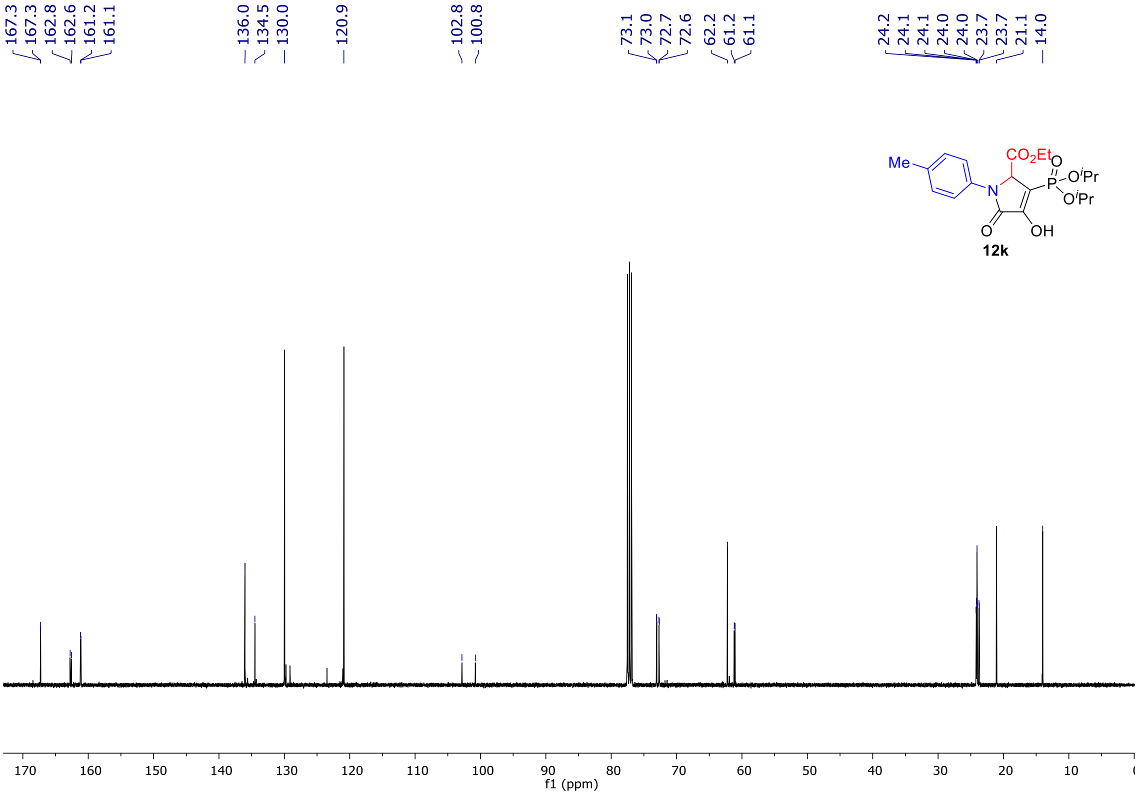

^{31}P NMR (162 MHz, $CDCl_3$)

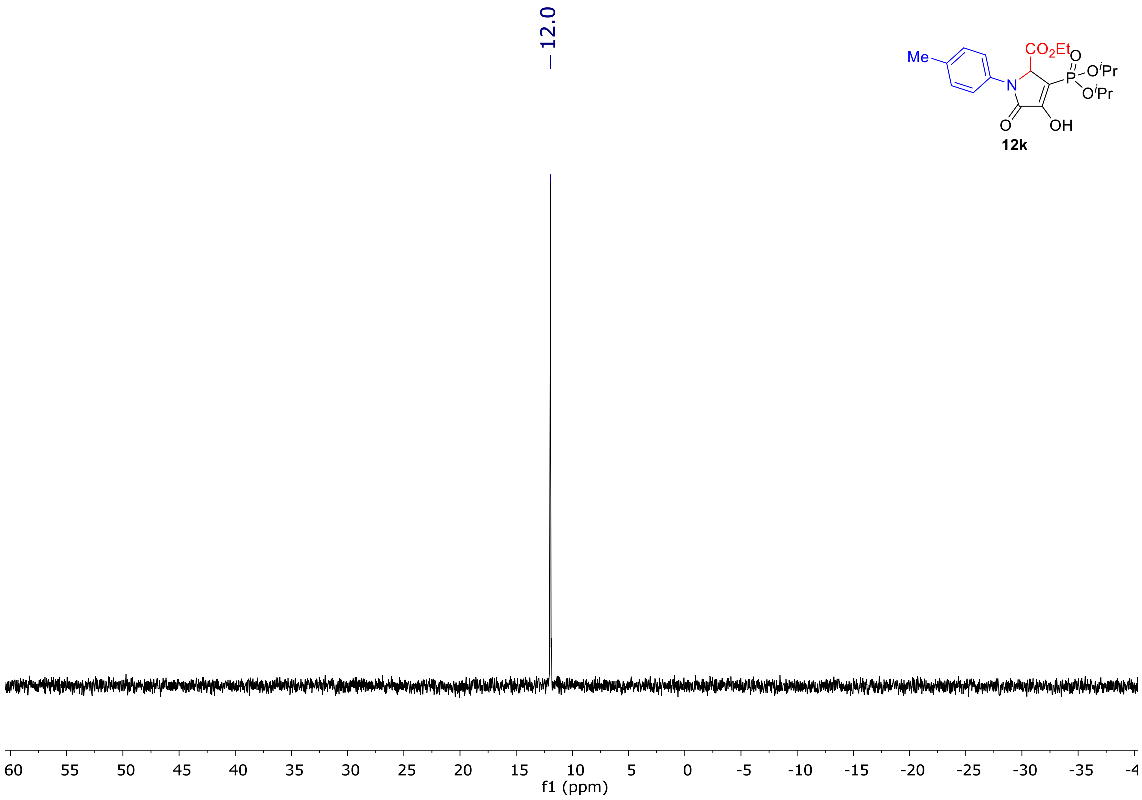

12k

*4-(diphenylphosphoryl)-3-hydroxy-5-phenyl-1-(p-tolyl)-1,5-dihydro-2H-pyrrol-2-onephosphonate (**12l**).*

^{1}H NMR (400 MHz, $CDCl_3$)

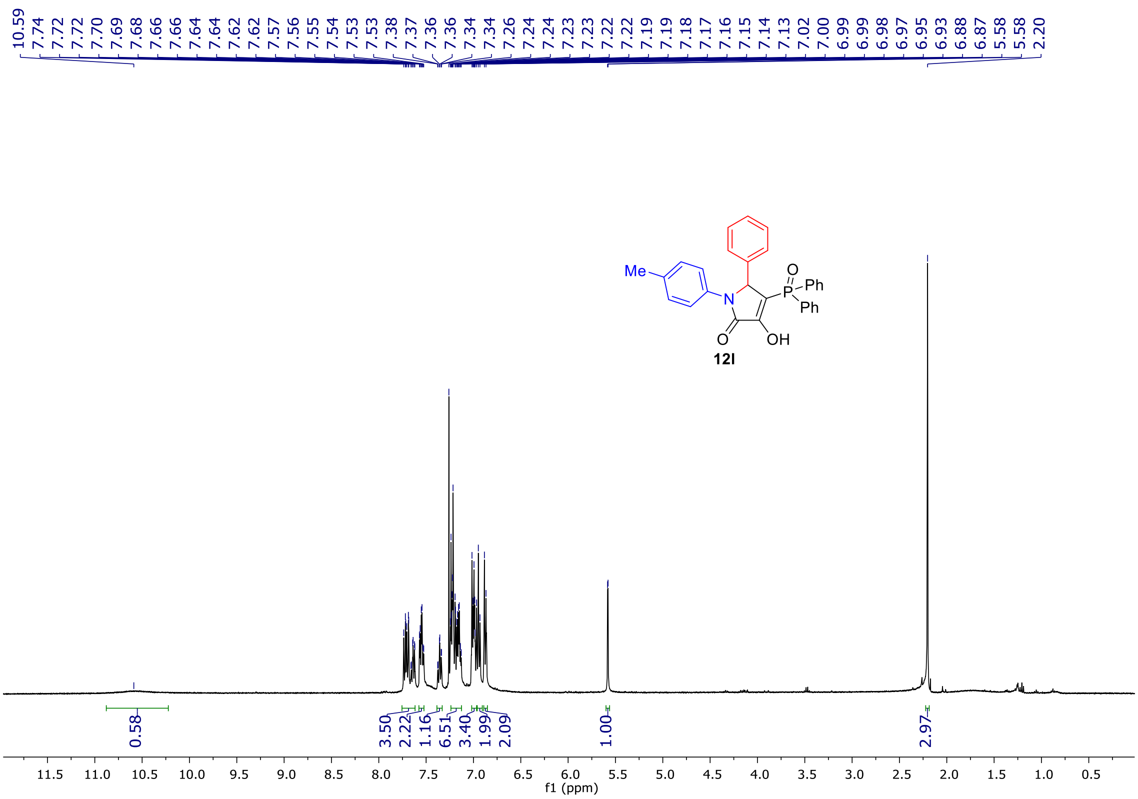

^{13}C NMR (101 MHz, $CDCl_3$)

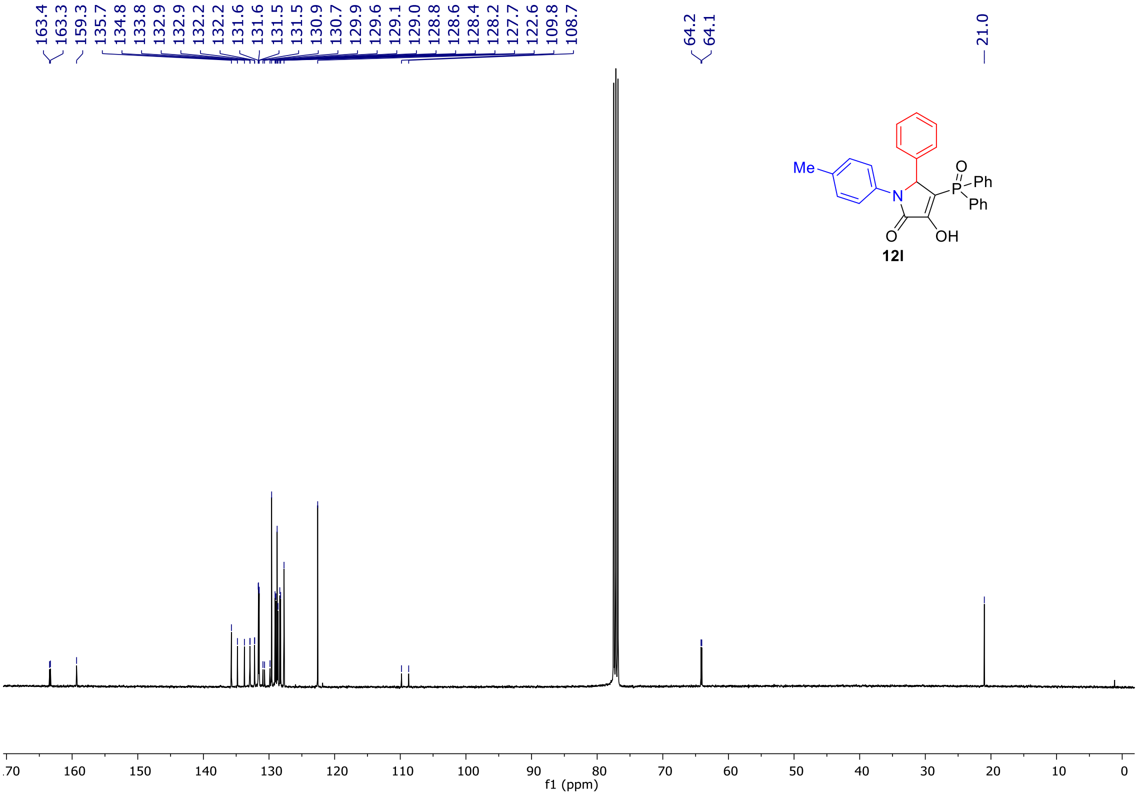

^{31}P NMR (121 MHz, $CDCl_3$)

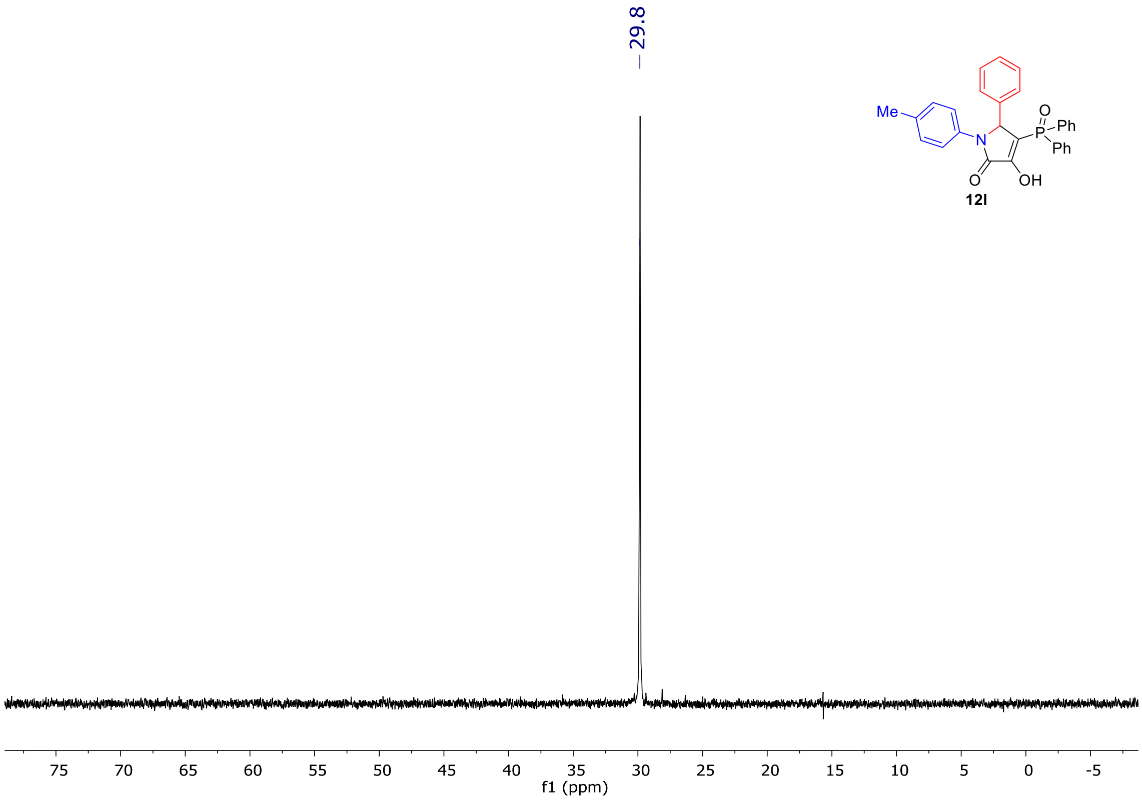

12l

*4-(diphenylphosphoryl)-3-hydroxy-1-(4-ethoxyphenyl)-5-phenyl-1,5-dihydro-2H-pyrrol-2-one (**12m**).*

¹H NMR (400 MHz, CDCl₃)

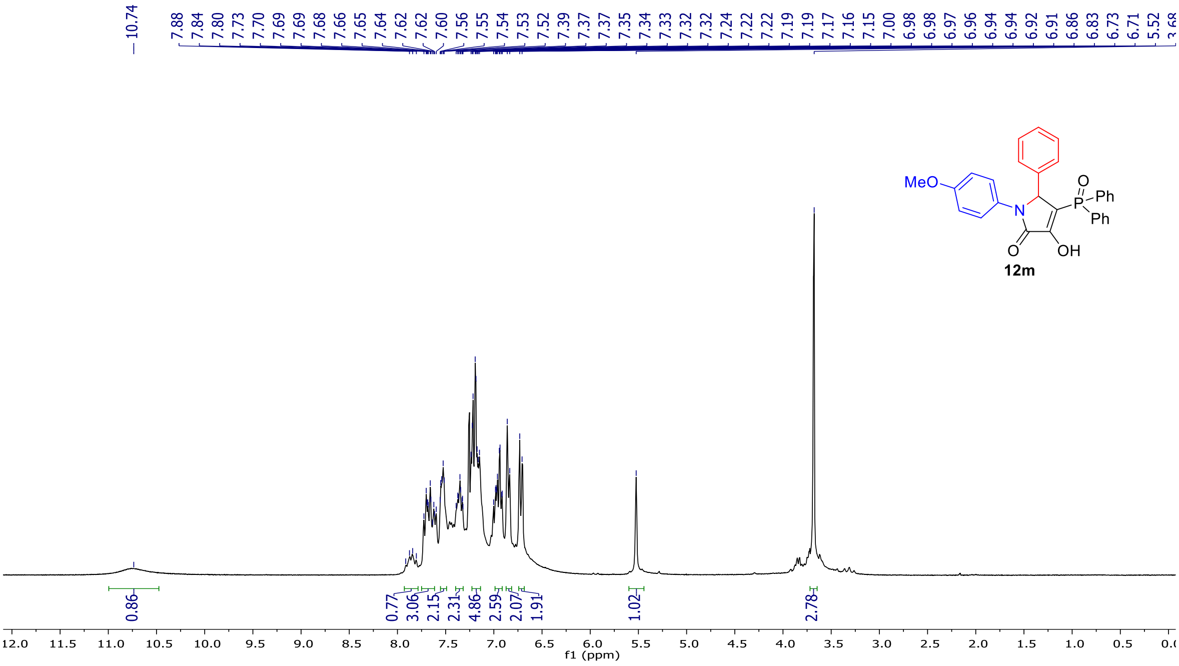

¹³C NMR (75 MHz, CDCl₃)

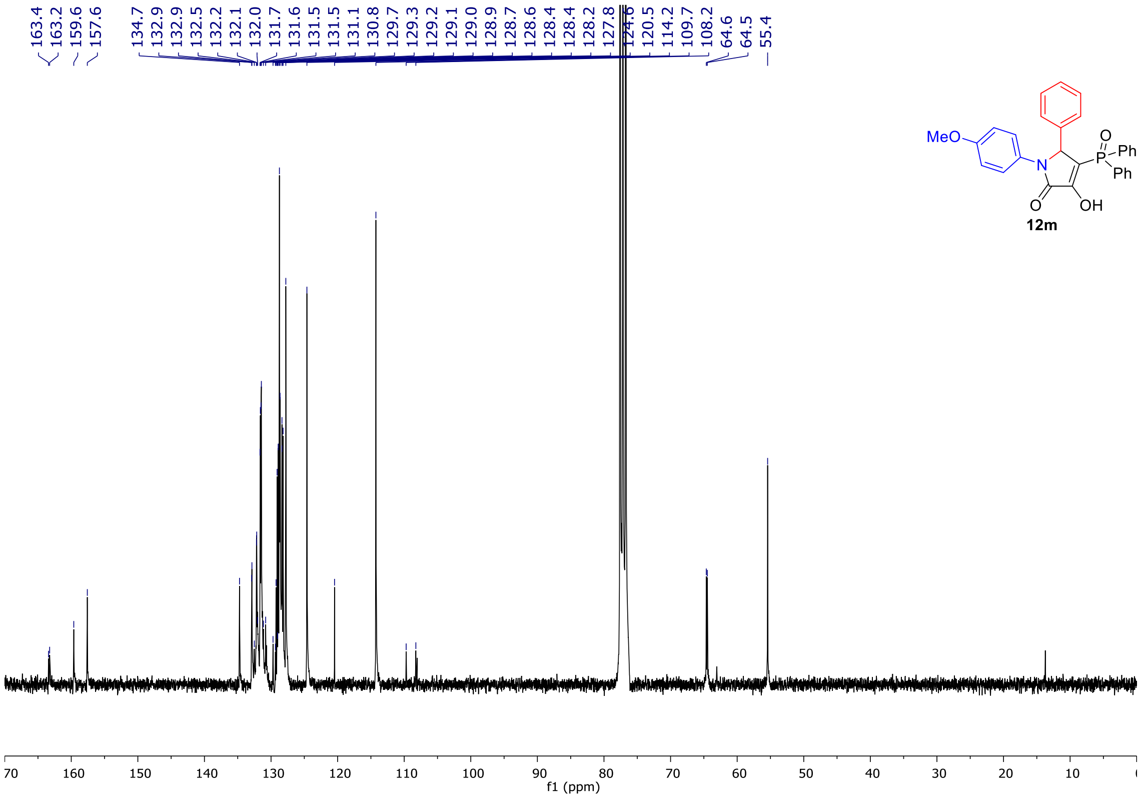

^{31}P NMR (121 MHz, $CDCl_3$)

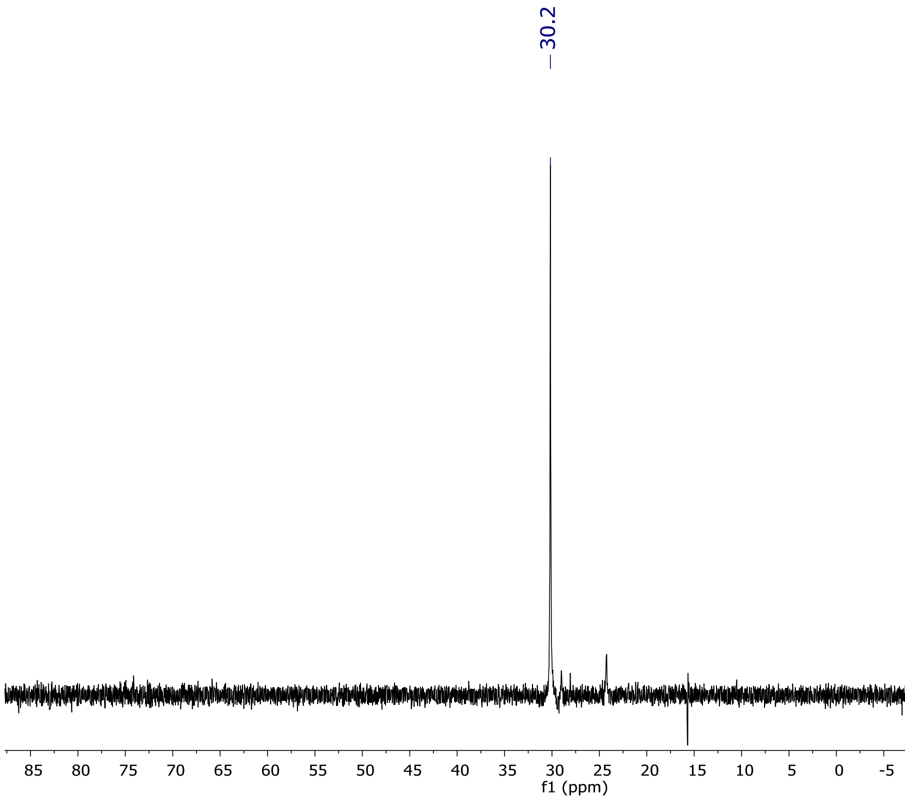

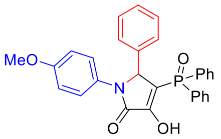

12m

*4-(diphenylphosphoryl)-1-(2-fluorophenyl)--hydroxy-5-phenyl-1,5-dihydro-2H-pyrrol-2-one (**12n**).*

^{1}H NMR (400 MHz, $CDCl_3$)

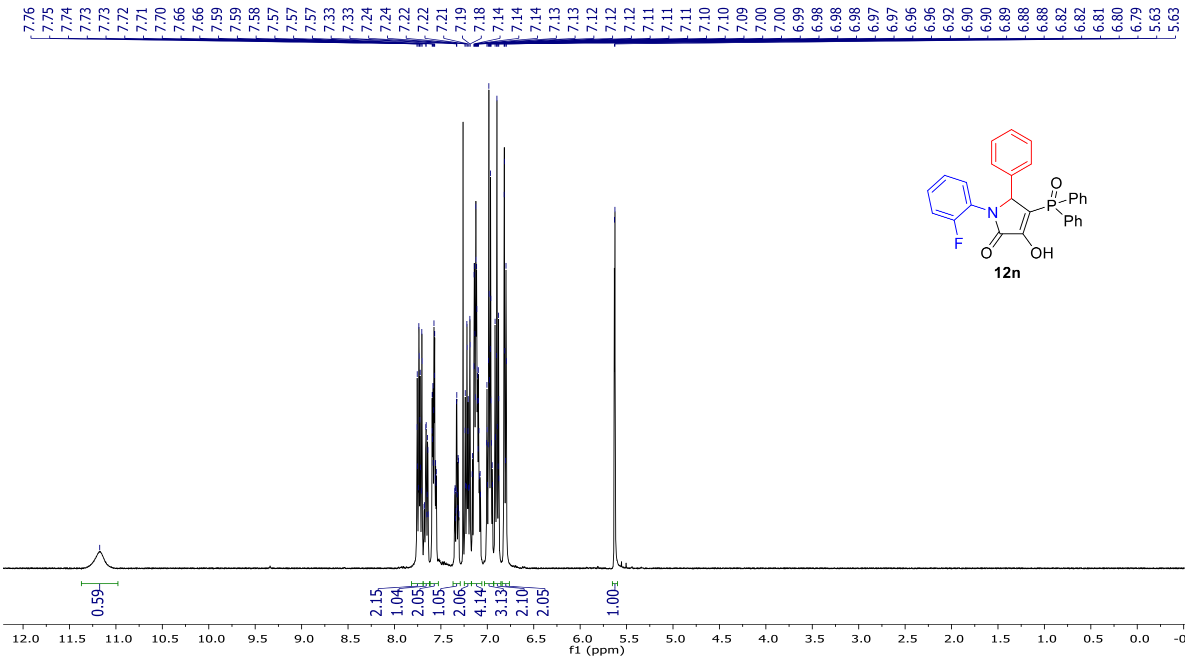

^{13}C NMR (75 MHz, $CDCl_3$)

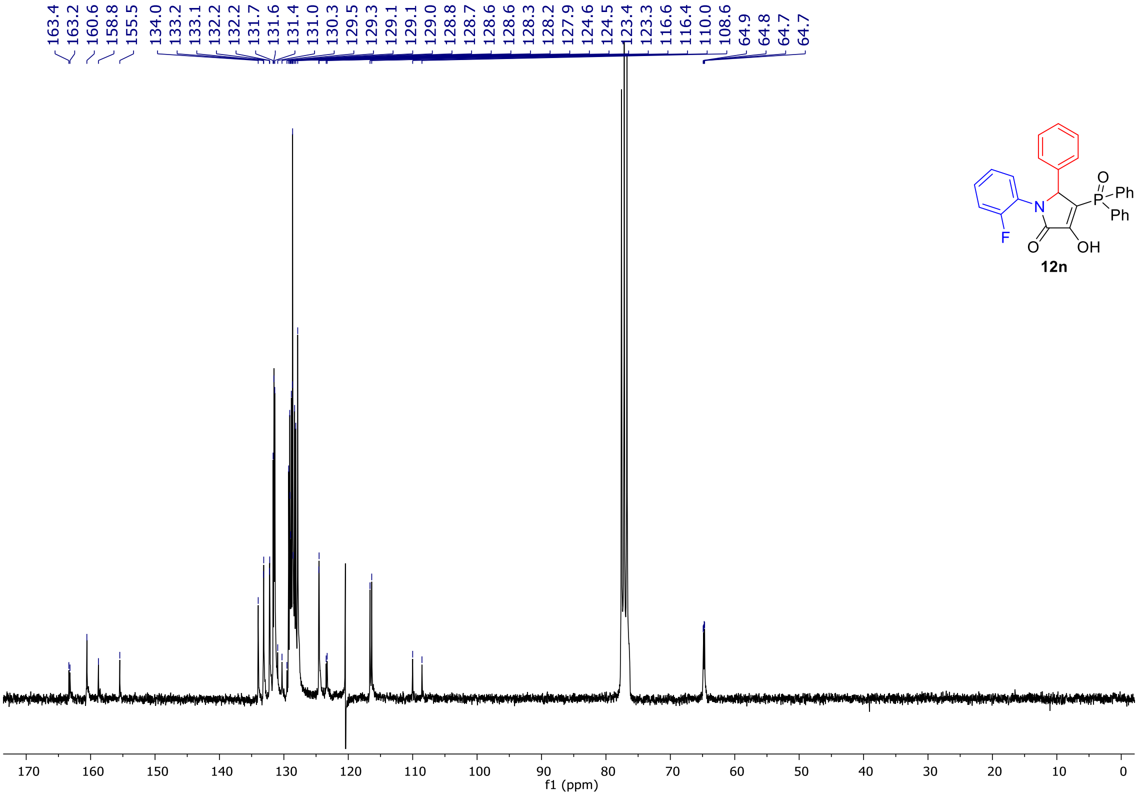

^{19}F NMR (282 MHz, $CDCl_3$)

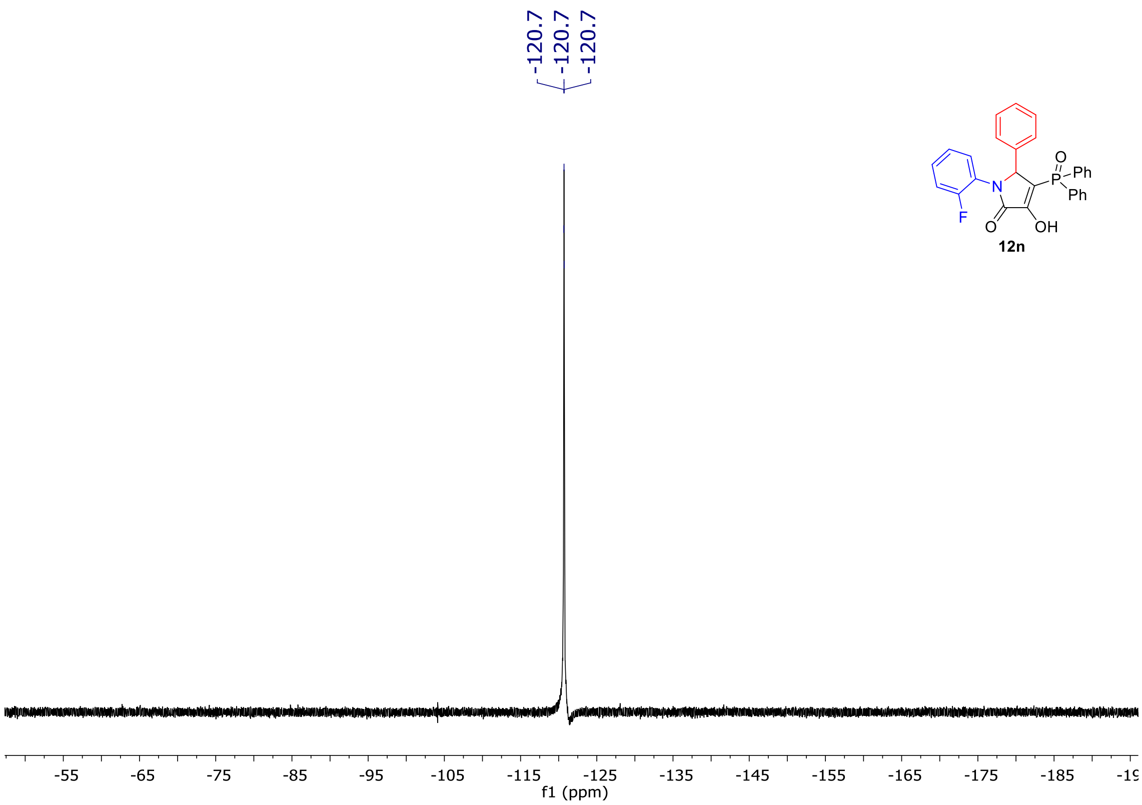

^{31}P NMR (121 MHz, $CDCl_3$)

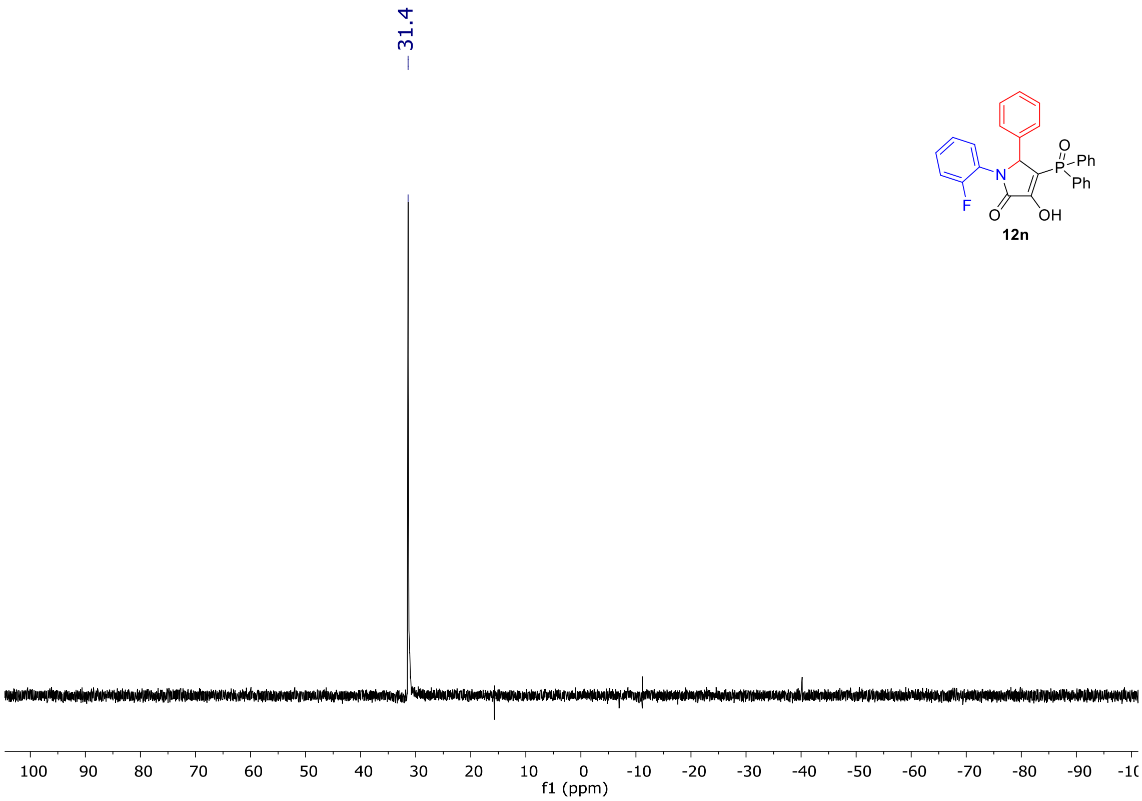

*4-(diphenylphosphoryl)-5-(4-fluorophenyl)-3-hydroxy-1-(p-tolyl)-1,5-dihydro-2H-pyrrol-2-one (**12o**).*

[1]**H NMR** (400 MHz, $CDCl_3$)

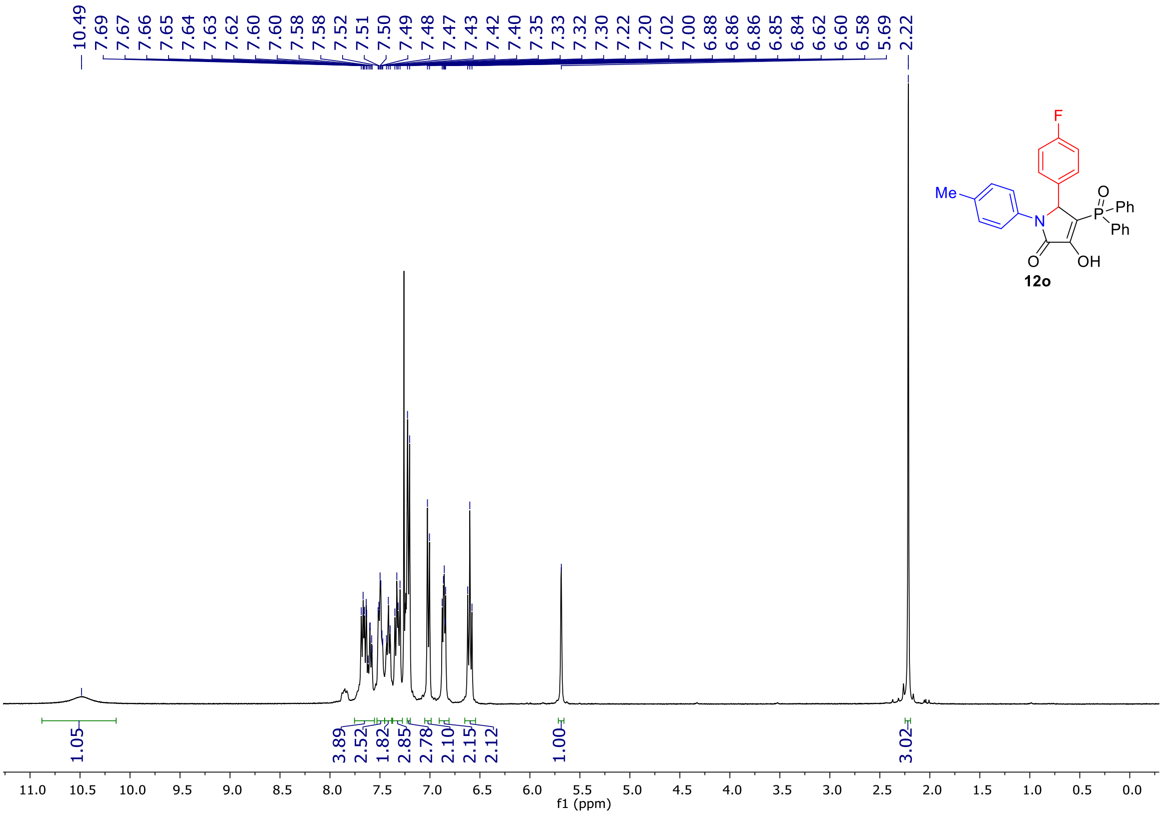

[13]**C NMR** (101 MHz, $CDCl_3$)

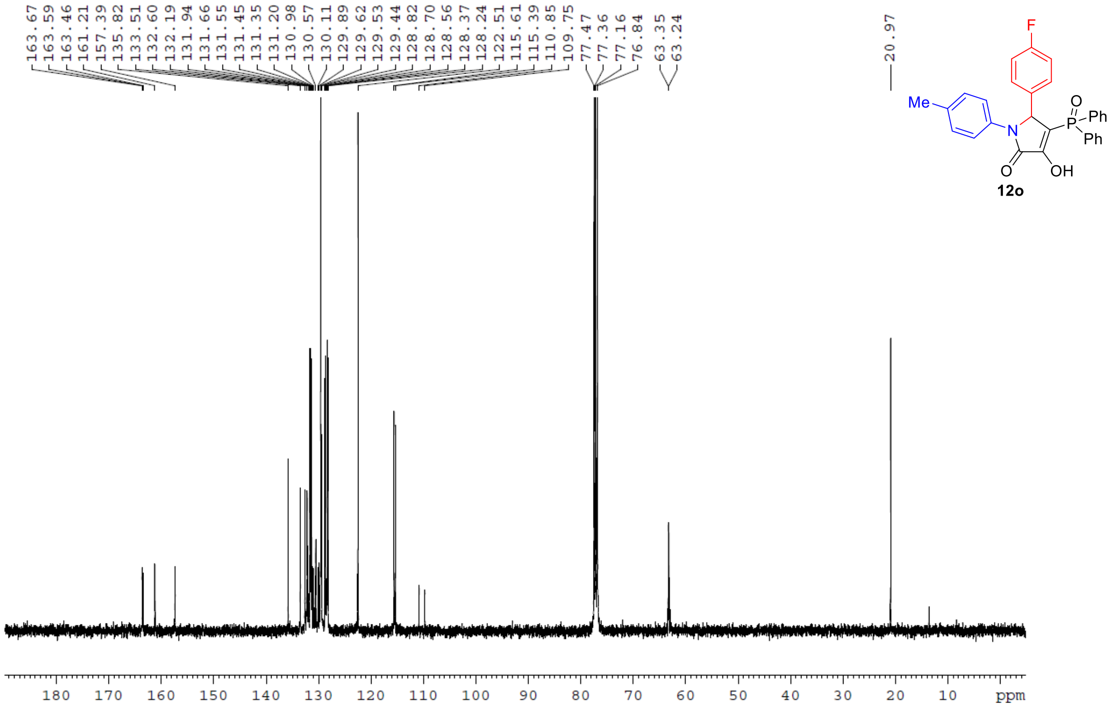

^{19}F NMR (282 MHz, $CDCl_3$)

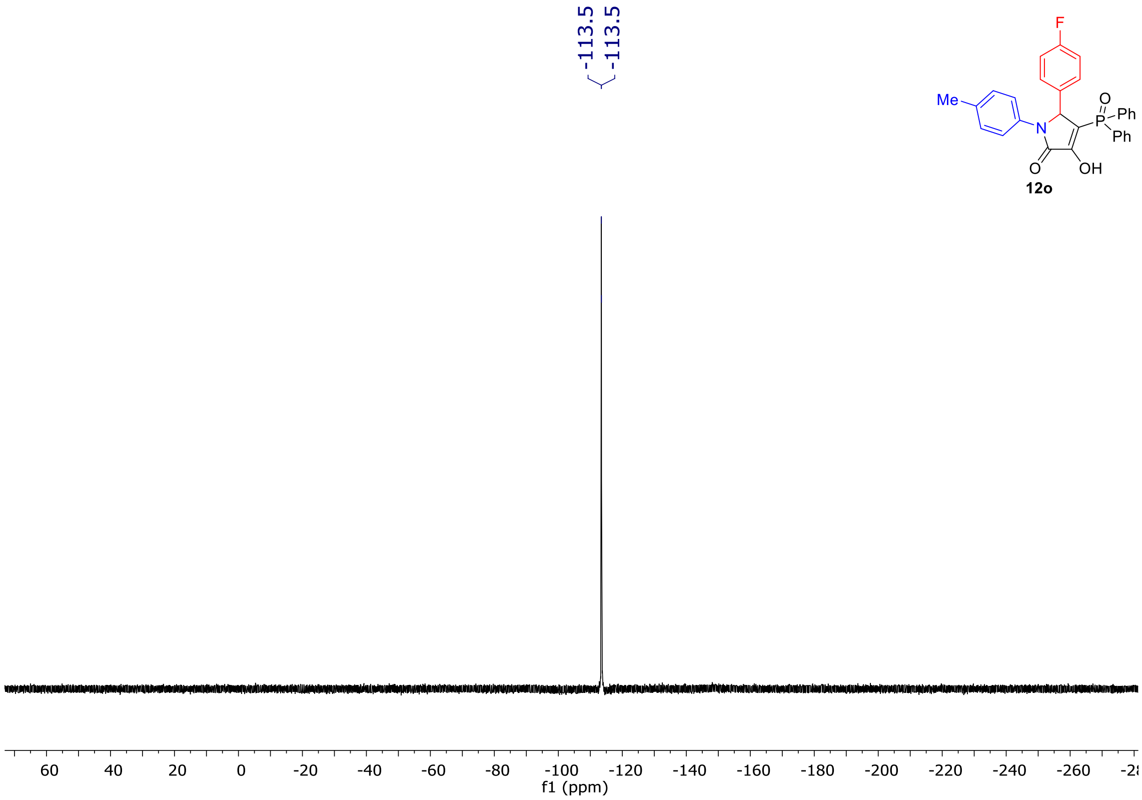

^{31}P NMR (162 MHz, $CDCl_3$)

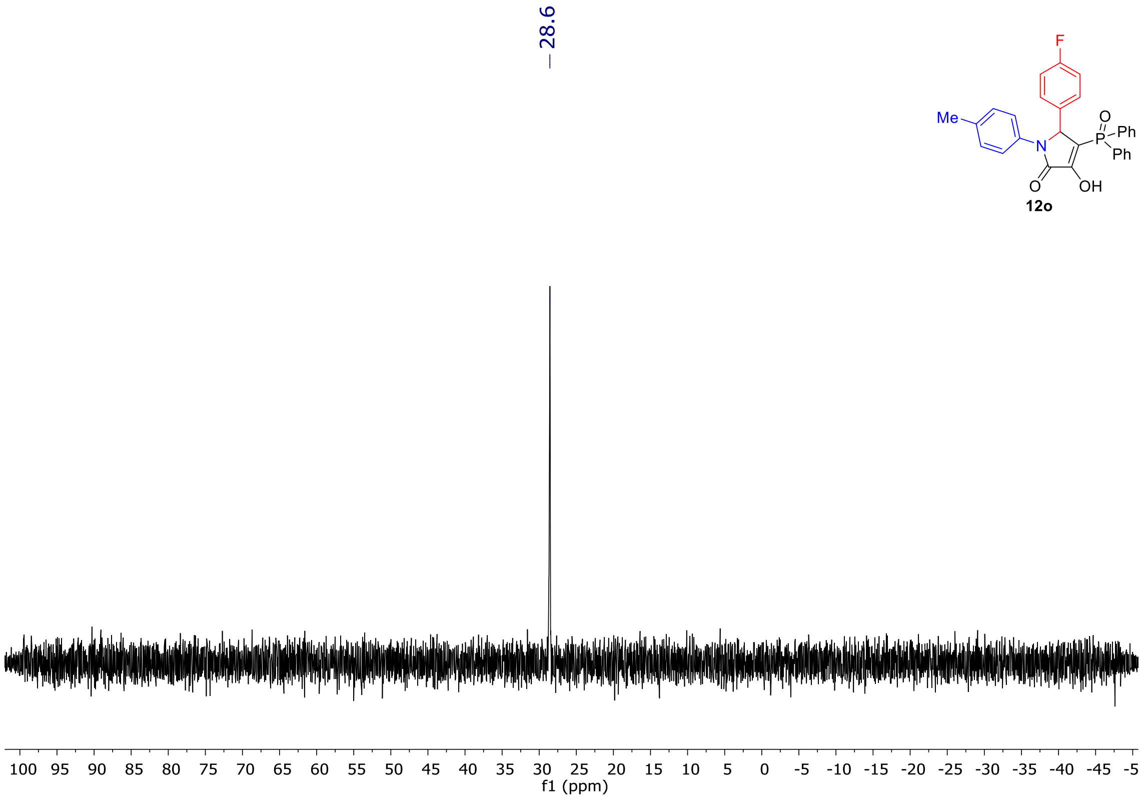

*4-(diphenylphosphoryl)-3-hydroxy-5-(perfluorophenyl)-1-(p-tolyl)-1,5-dihydro-2H-pyrrol-2-one (**12p**).*

^{1}H NMR (400 MHz, MeOD-*d*$_4$)

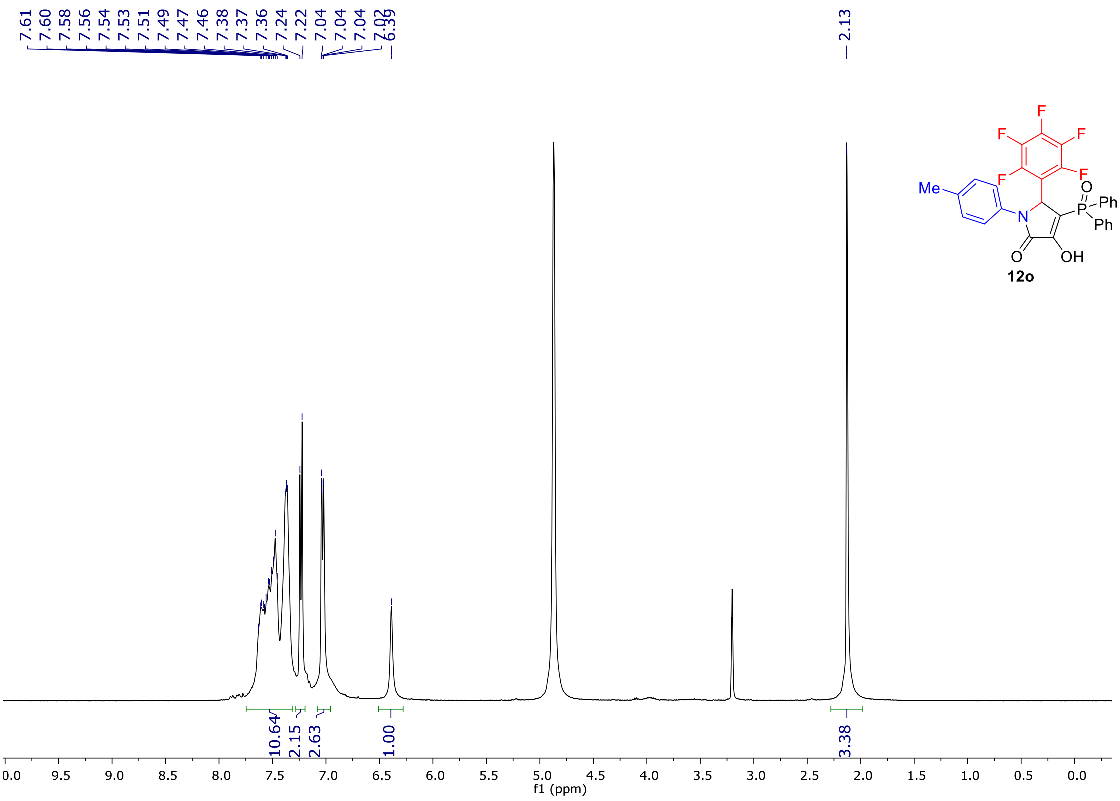

^{13}C NMR (101 MHz, MeOD-*d*$_4$)

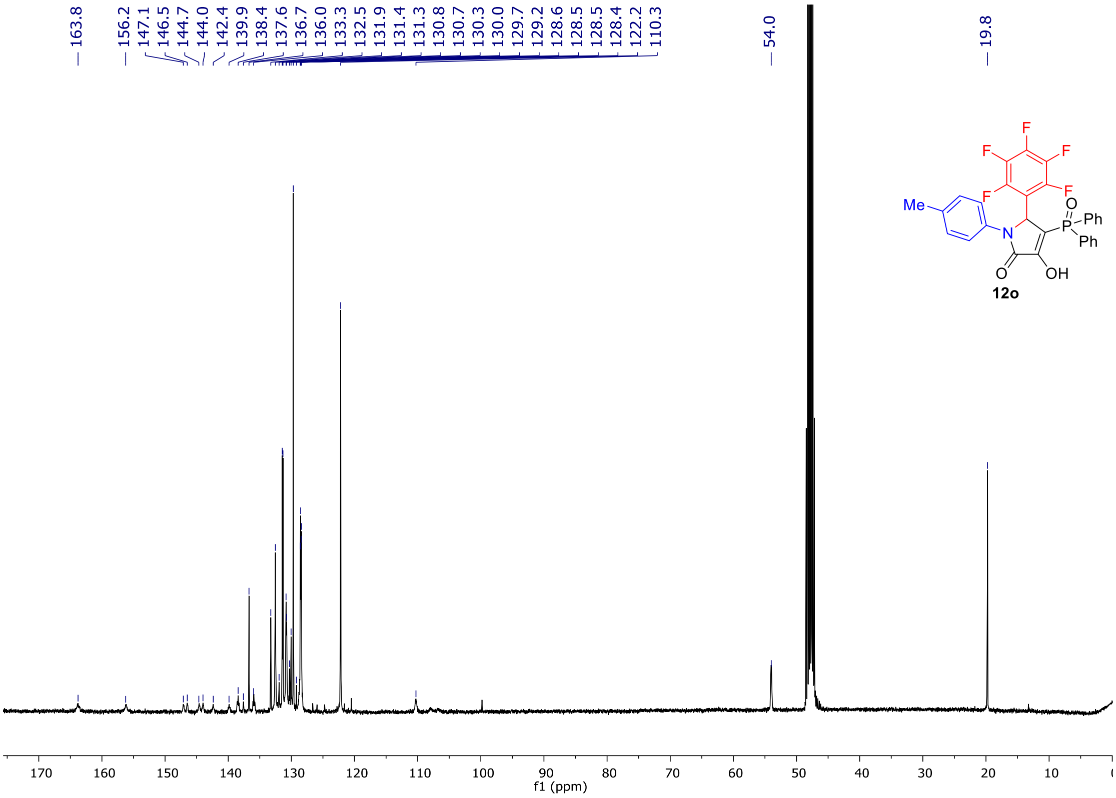

^{19}F NMR (282 MHz, MeOD-d_4)

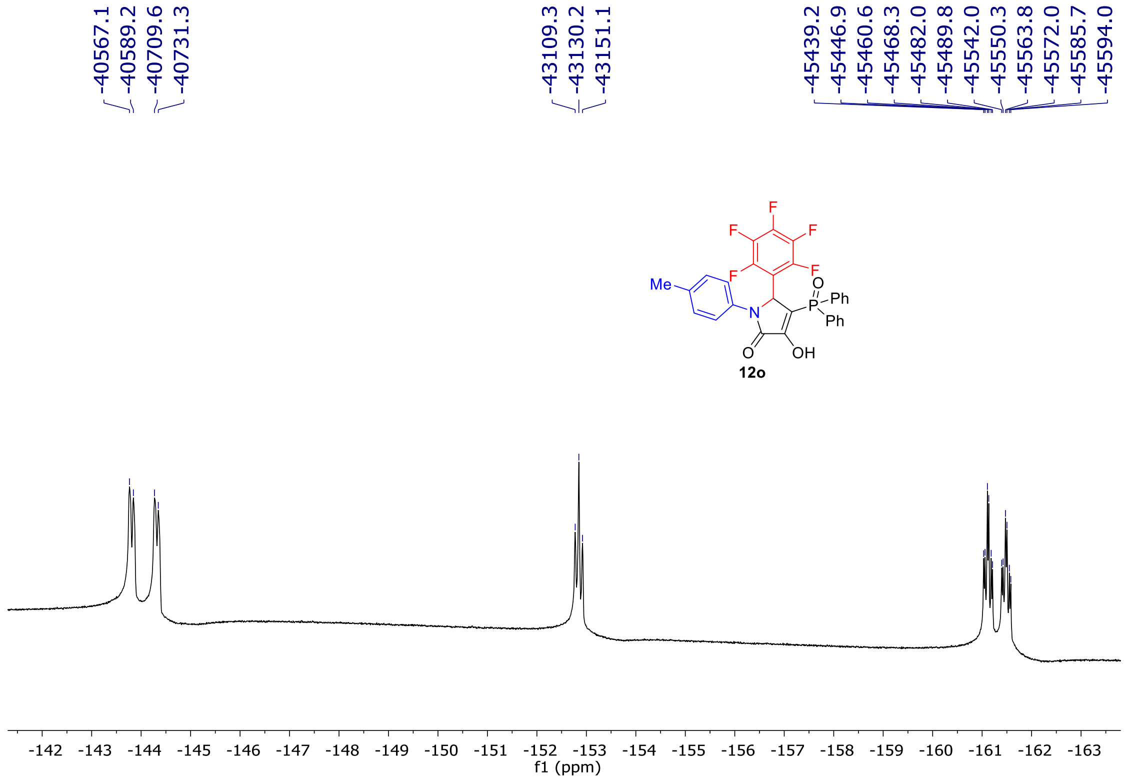

^{31}P NMR (121 MHz, MeOD-d_4)

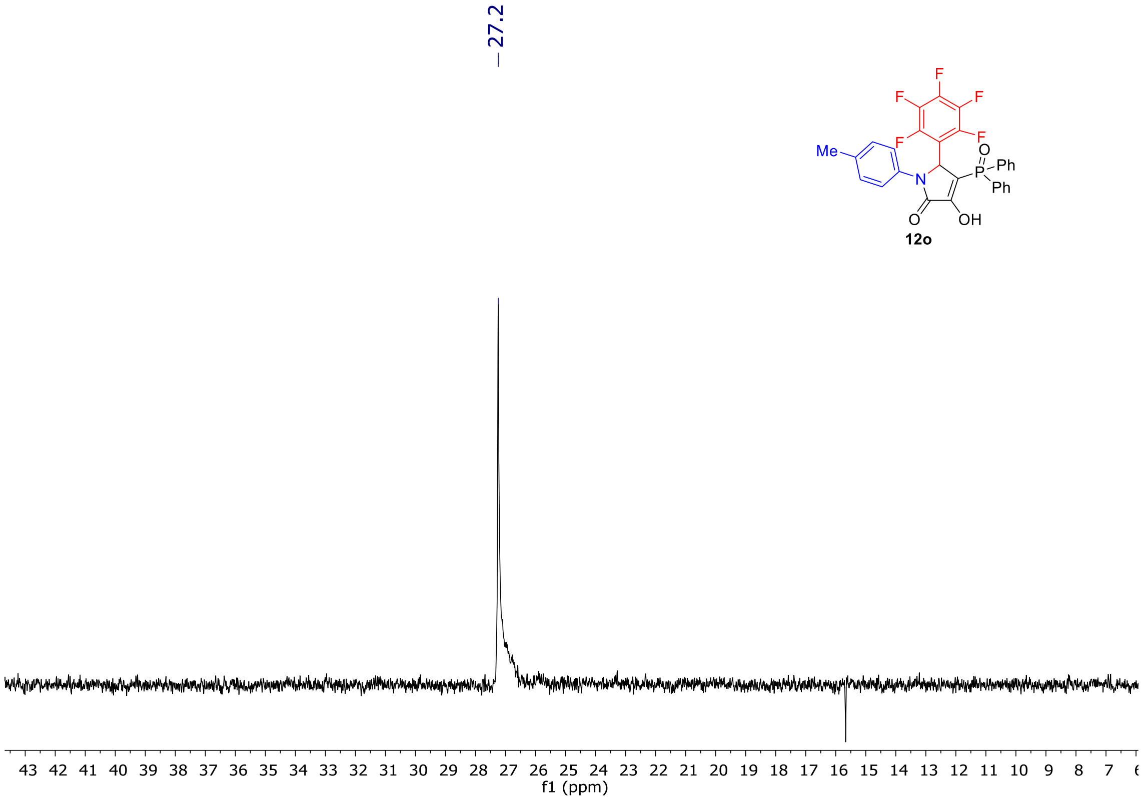

*Ethyl 4-hydroxy-5-oxo-2-phenyl-1-(p-tolyl)-2,5-dihydro-1H-pyrrole-3-carboxylate (**13a**).*

^{1}H NMR (400 MHz, $CDCl_3$)

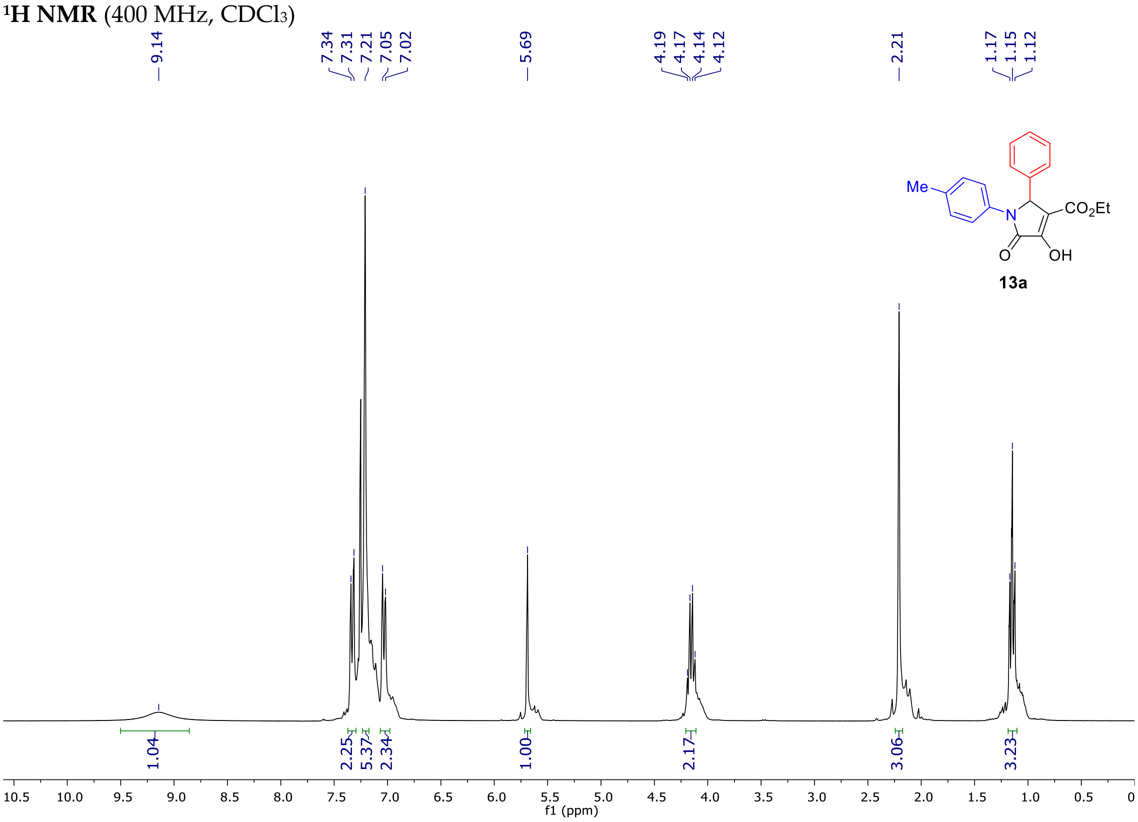

^{13}C {^{1}H} NMR (101 MHz, $CDCl_3$)

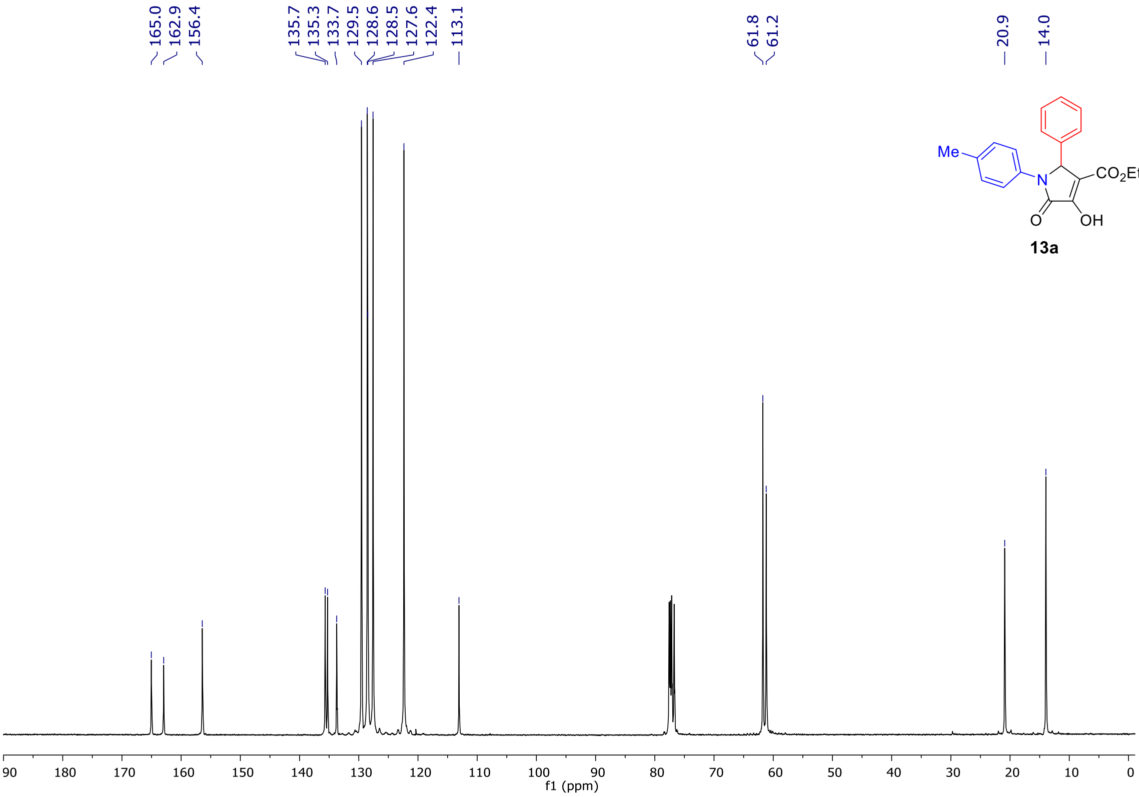

*Ethyl 4-hydroxy-1-(4-methoxyphenyl)-5-oxo-2-phenyl-2,5-dihydro-1H-pyrrole-3-carboxylate (**13b**).*

^{1}H NMR (400 MHz, $CDCl_3$)

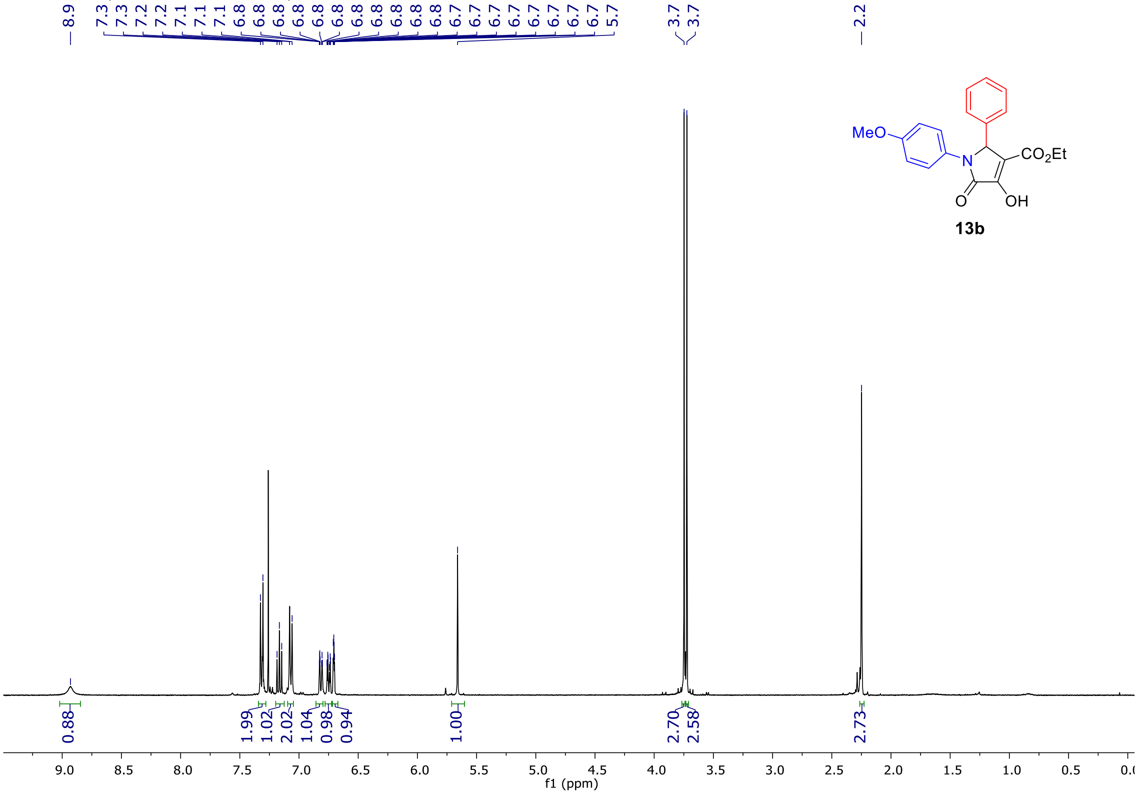

^{13}C {^{1}H} NMR (101 MHz, $CDCl_3$)

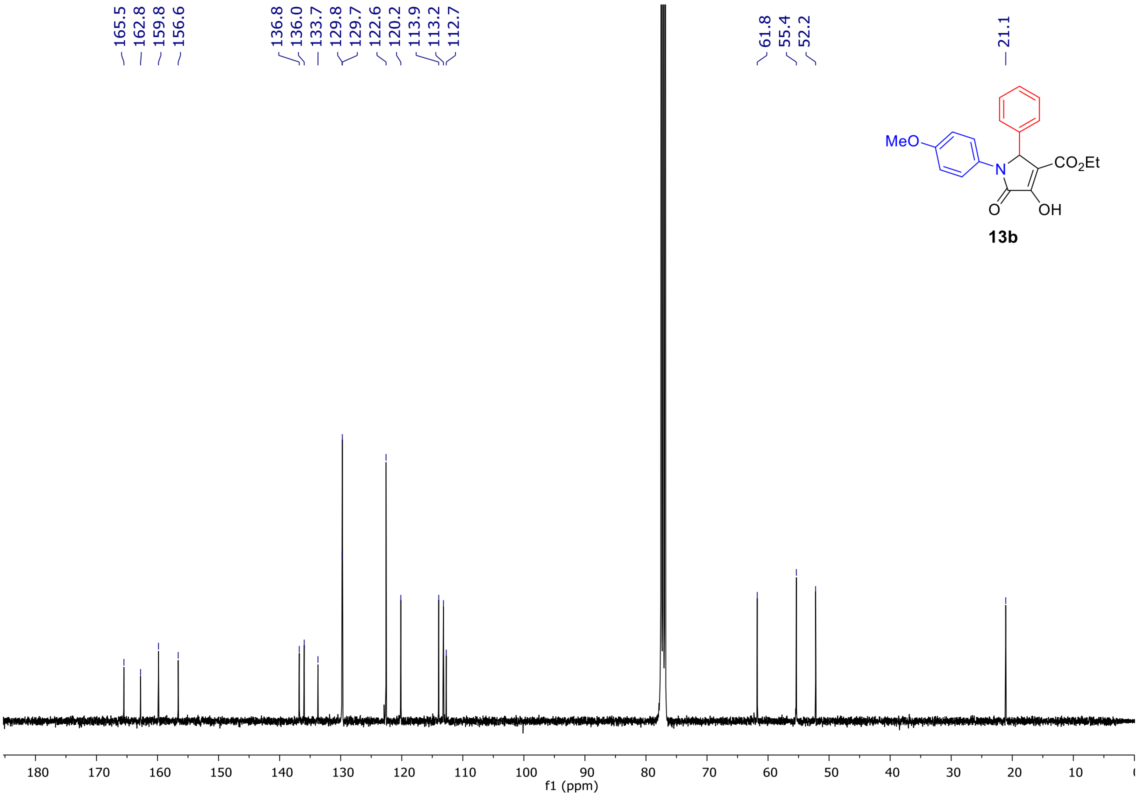

*Ethyl 1-benzyl-4-hydroxy-5-oxo-2-phenyl-2,5-dihydro-1H-pyrrole-3-carboxylate (**13c**).*

^{1}H NMR (300 MHz, $CDCl_3$)

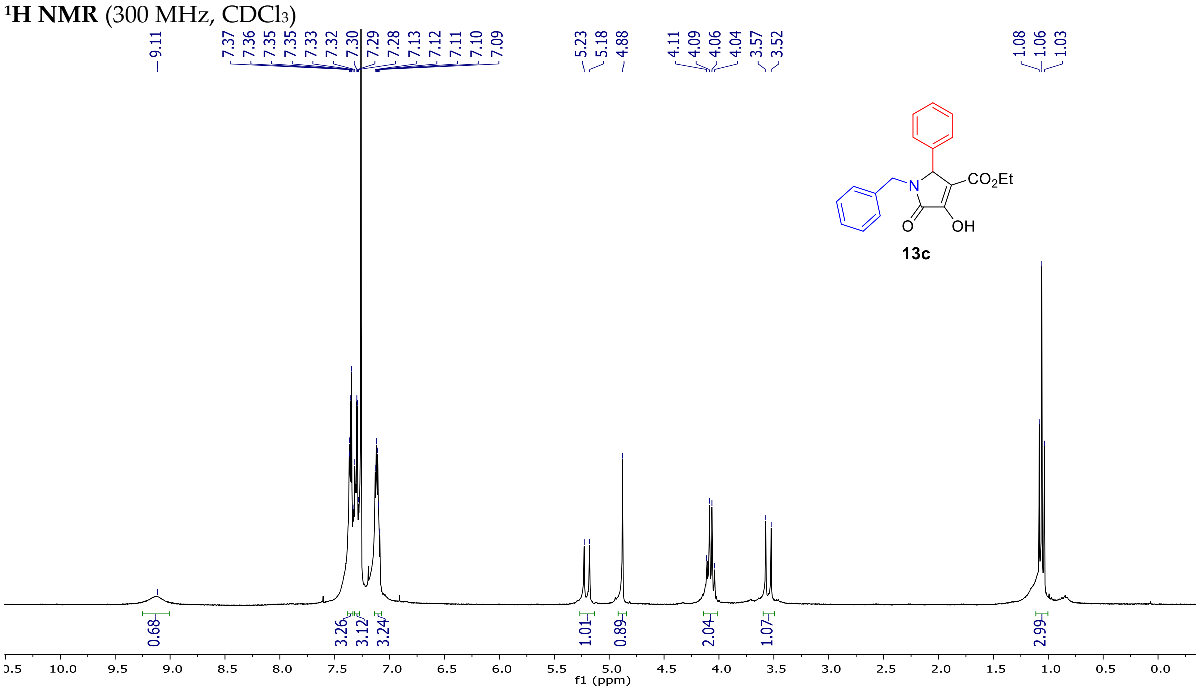

^{13}C {^{1}H} NMR (75 MHz, $CDCl_3$)

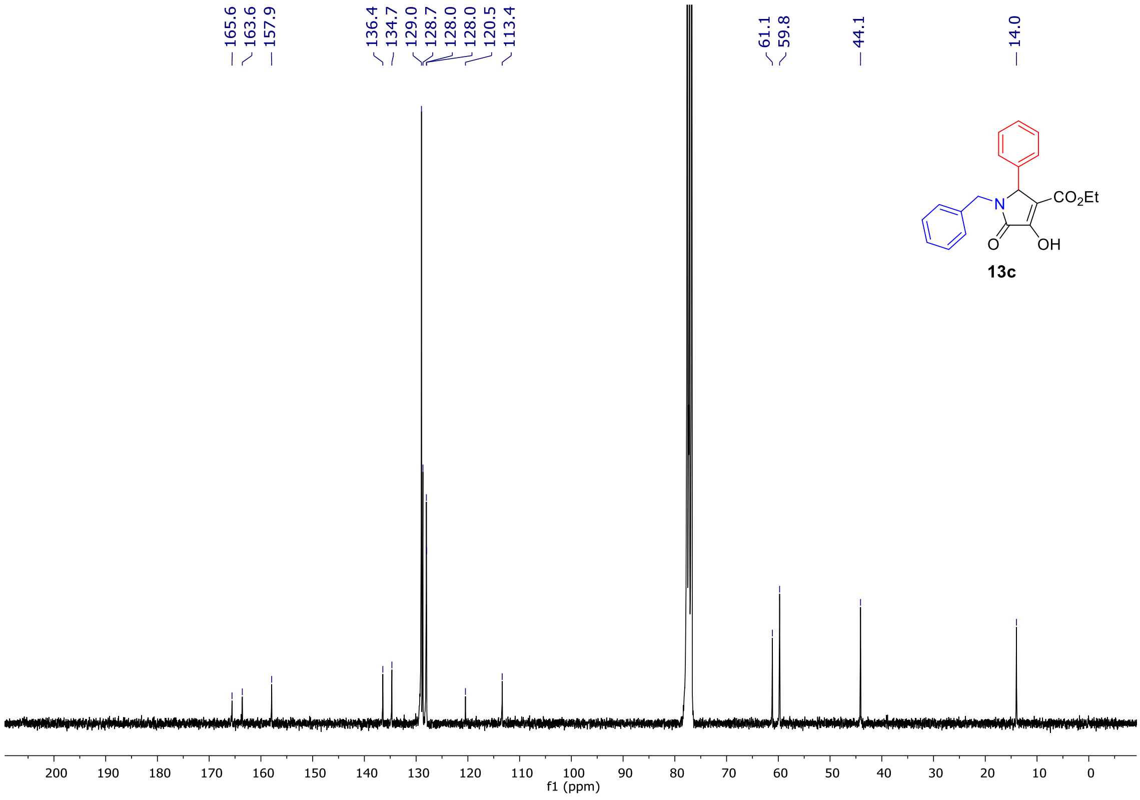

Methyl 1-benzyl-4-hydroxy-5-oxo-2-(4-(trifluoromethyl)phenyl)-2,5-dihydro-1H-pyrrole-3-carboxylate (***13d***).

^{1}H NMR (400 MHz, $CDCl_3$)

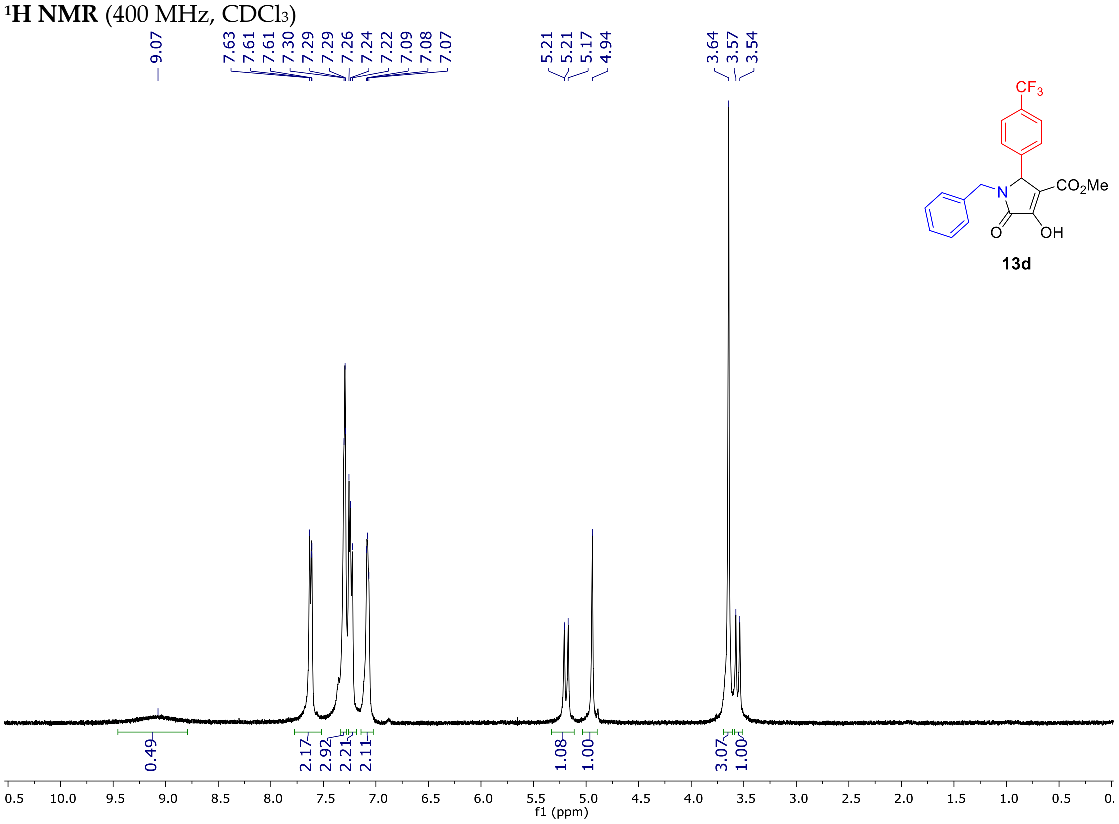

^{13}C {^{1}H} NMR (101 MHz, $CDCl_3$)

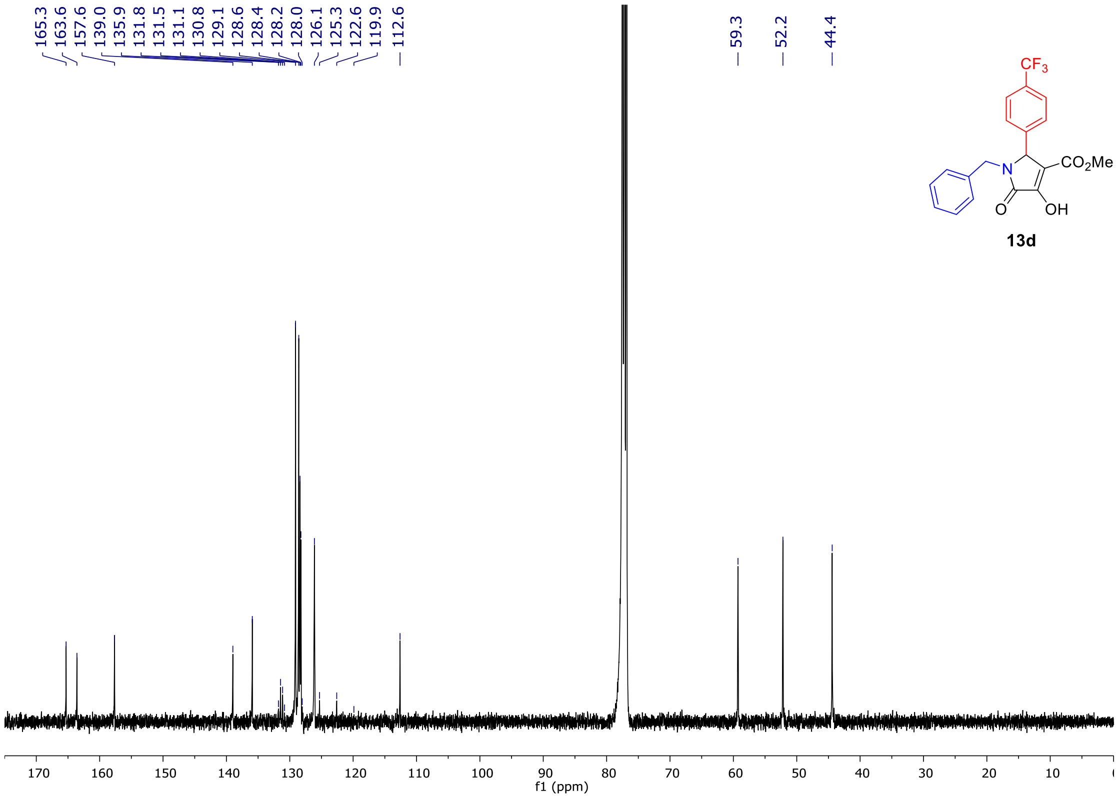

^{19}F NMR (282 MHz, $CDCl_3$)

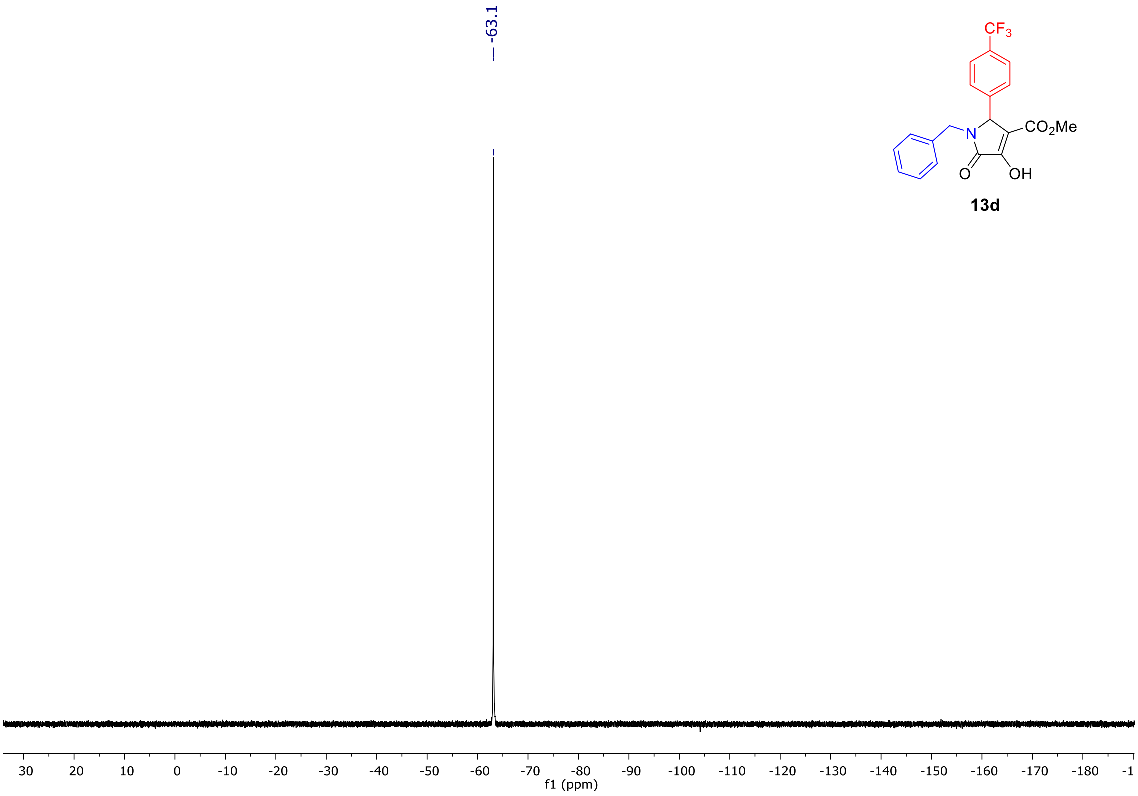

13d

*Methyl 4-hydroxy-2-(3-methoxyphenyl)-5-oxo-1-(p-tolyl)-2,5-dihydro-1H-pyrrole-3-carboxylate (**13e**).*

¹H NMR (400 MHz, $CDCl_3$)

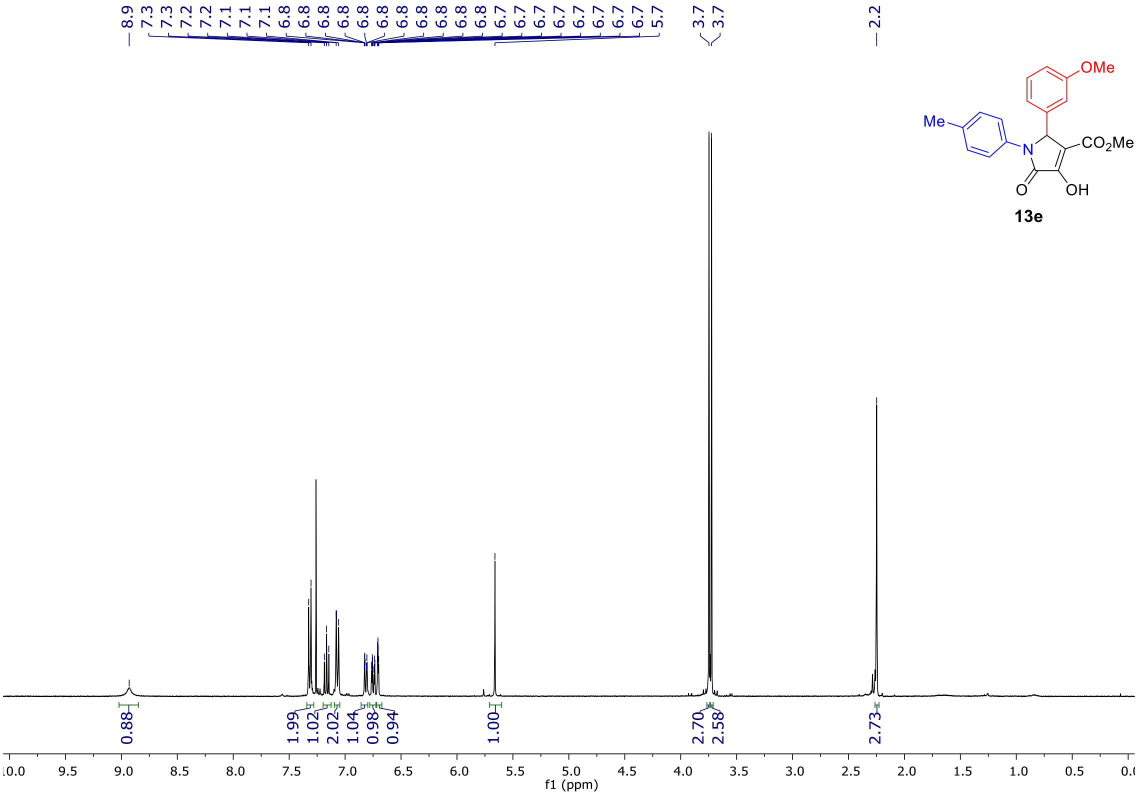

¹³C {¹H} NMR (101 MHz, $CDCl_3$)

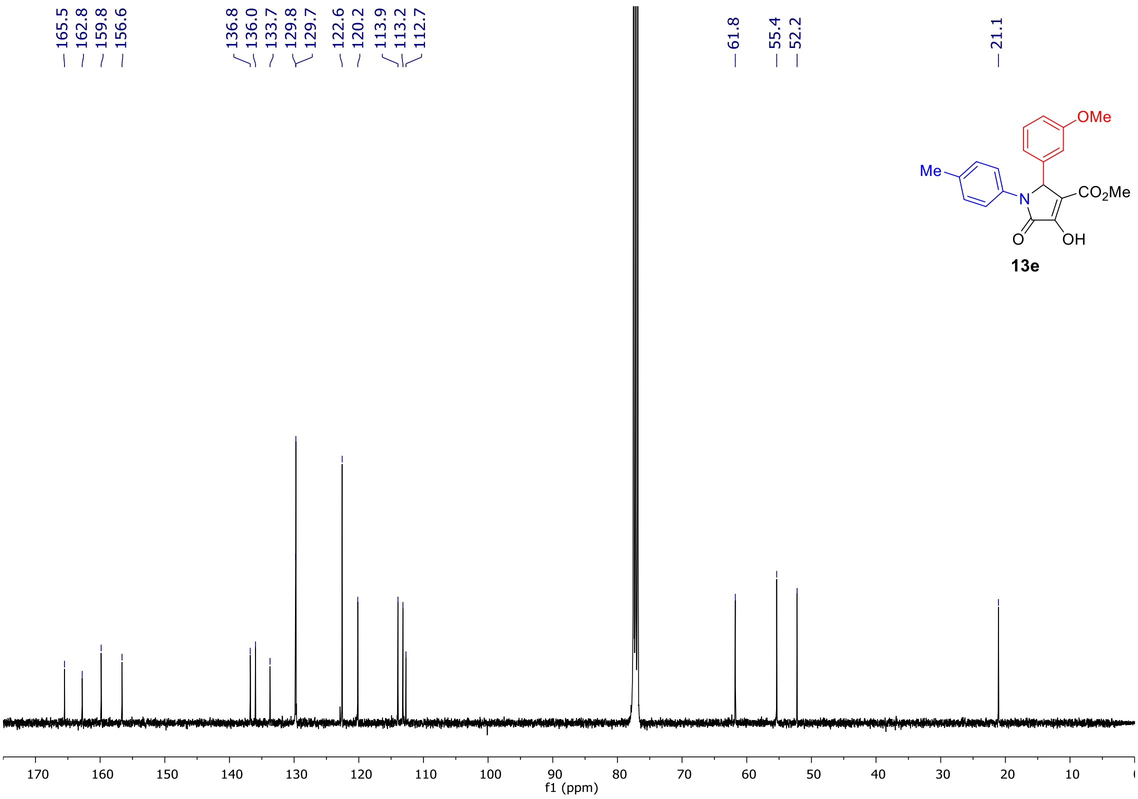

*Methyl 1-benzyl-4-hydroxy-2-(3-methoxyphenyl)-5-oxo-2,5-dihydro-1H-pyrrole-3-carboxylate (**13f**).*

¹H NMR (400 MHz, $CDCl_3$)

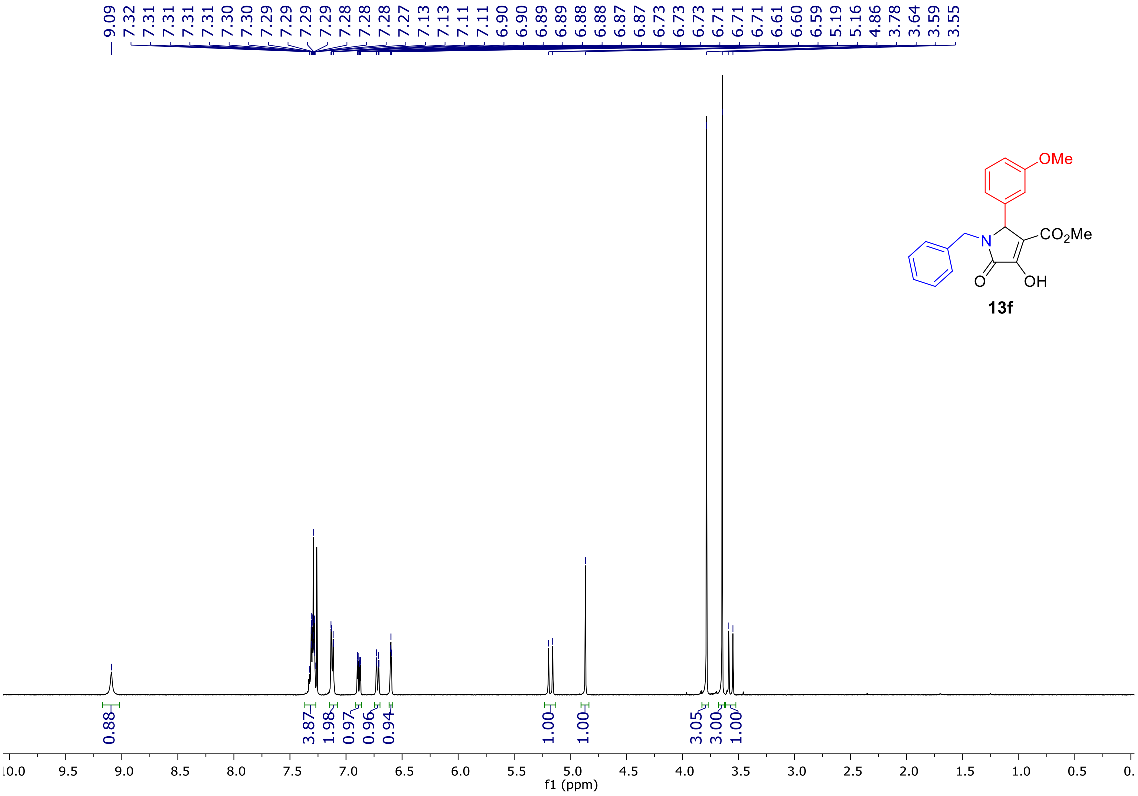

¹³C {¹H} NMR (101 MHz, $CDCl_3$)

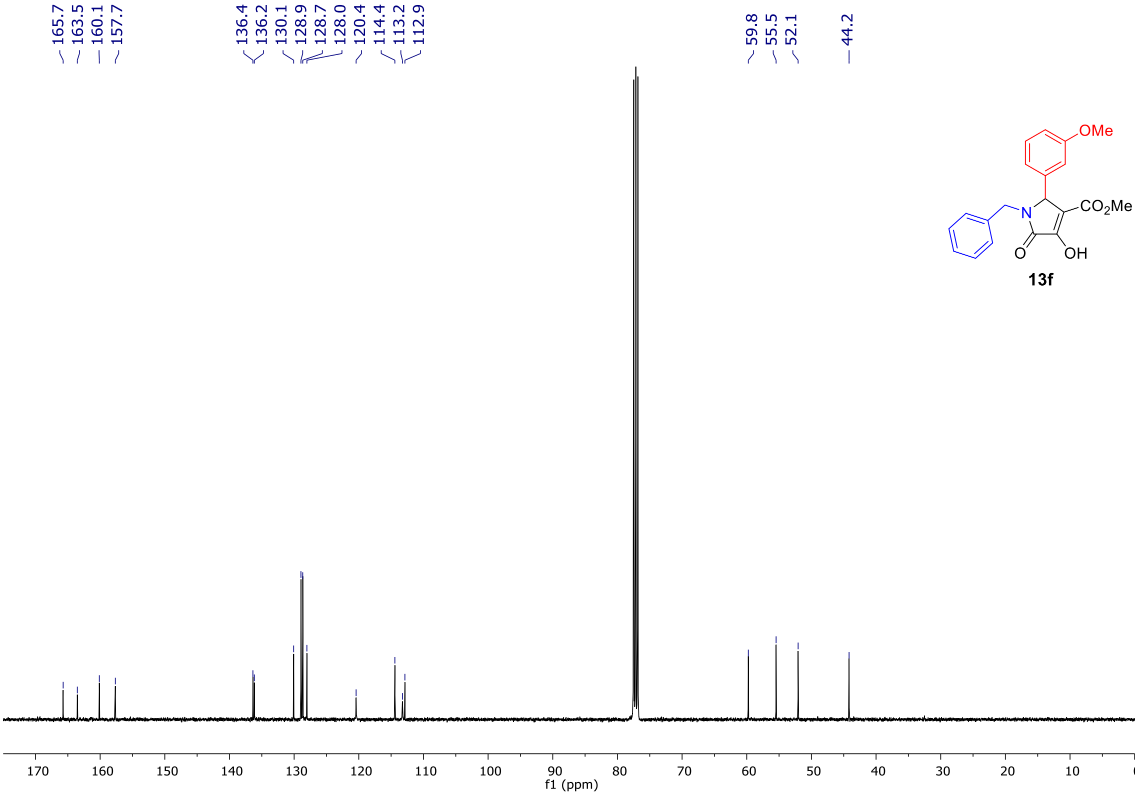

*Methyl 4-hydroxy-2-(4-hydroxy-3-methoxyphenyl)-5-oxo-1-(p-tolyl)-2,5-dihydro-1H-pyrrole-3-carboxylate (**13g**).*

^{1}H NMR (400 MHz, $CDCl_3$)

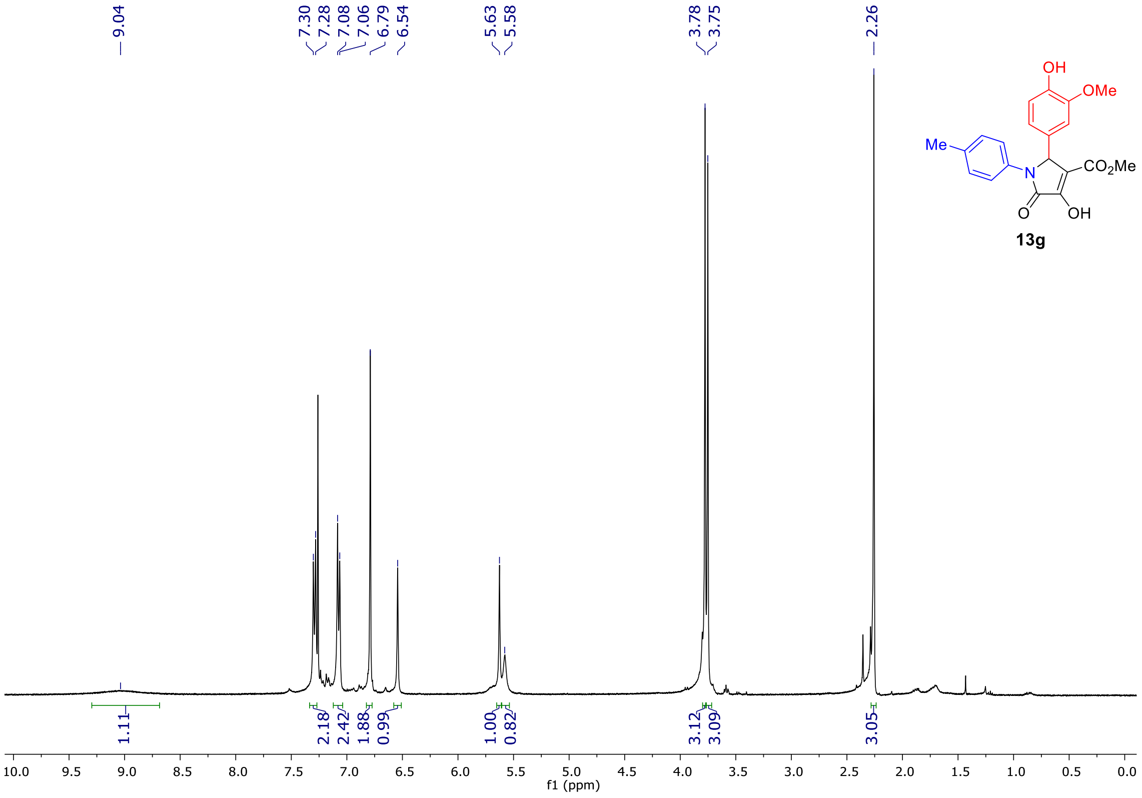

^{13}C {^{1}H} NMR (101 MHz, $CDCl_3$)

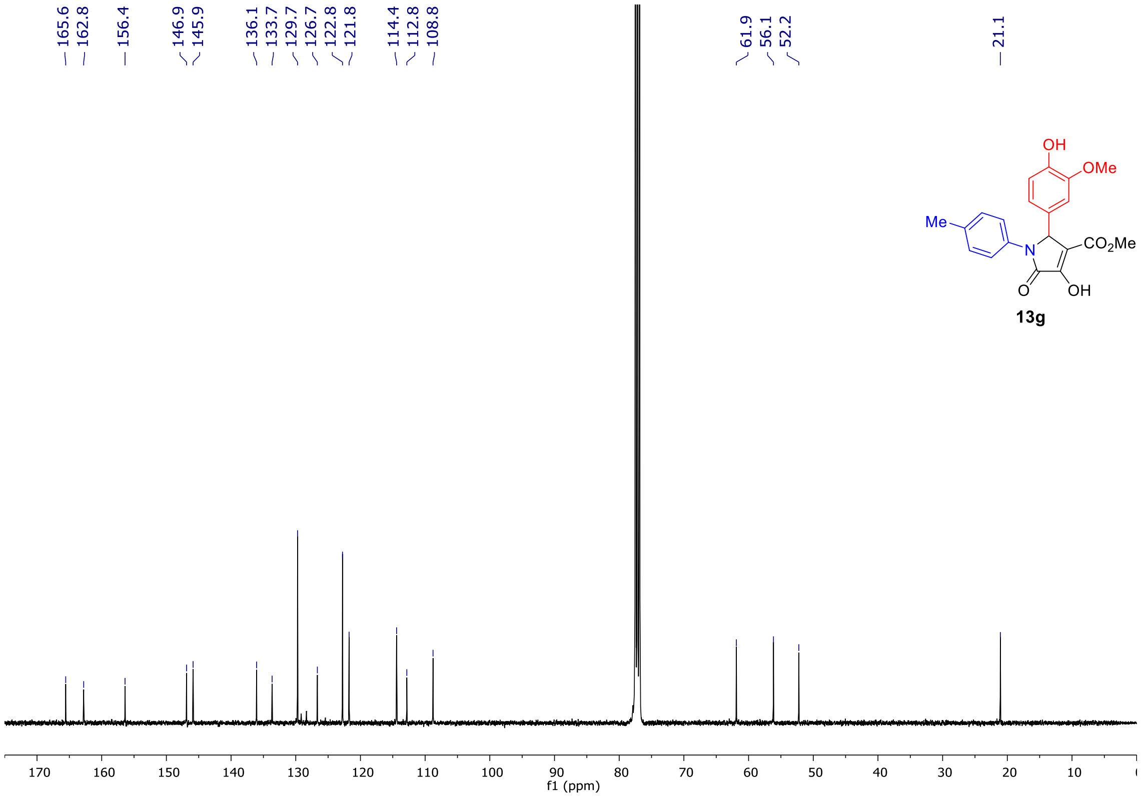

3. HPLC chromatograms of compounds 4, 8, 9, 12 and 13

*Ethyl 5-oxo-2-phenyl-1-(p-tolyl)-4-(p-tolylamino)-2,5-dihydro-1H-pyrrole-3-carboxylate (**4a**).*

Purity: 98.6%.

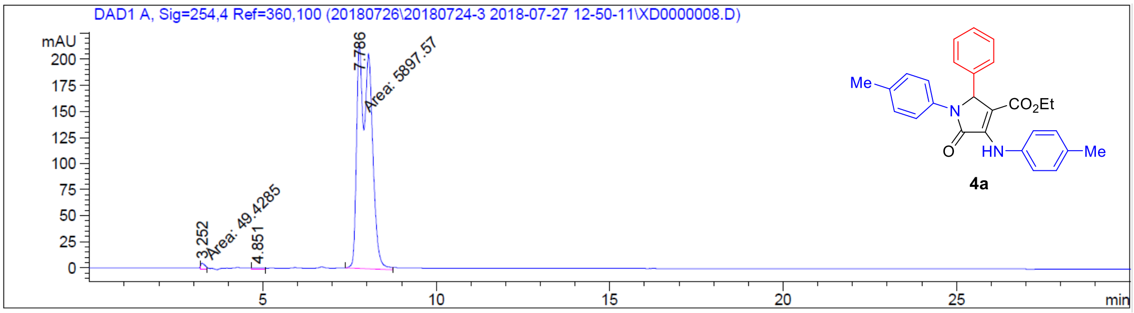

Peak #	RetTime [min]	Type	Width [min]	Area [mAU*s]	Height [mAU]	Area %
1	3.252	MM	0.1446	49.42852	5.69859	0.8270
2	4.851	VB	0.2778	29.82328	1.39299	0.4990
3	7.786	MM	0.4557	5897.57227	215.67889	98.6740

*Ethyl 1-(4-methoxyphenyl)-4-((4-methoxyphenyl)amino)-5-oxo-2-phenyl-2,5-dihydro-1H-pyrrole-3-carboxylate (**4b**).*

Purity: 99.2%.

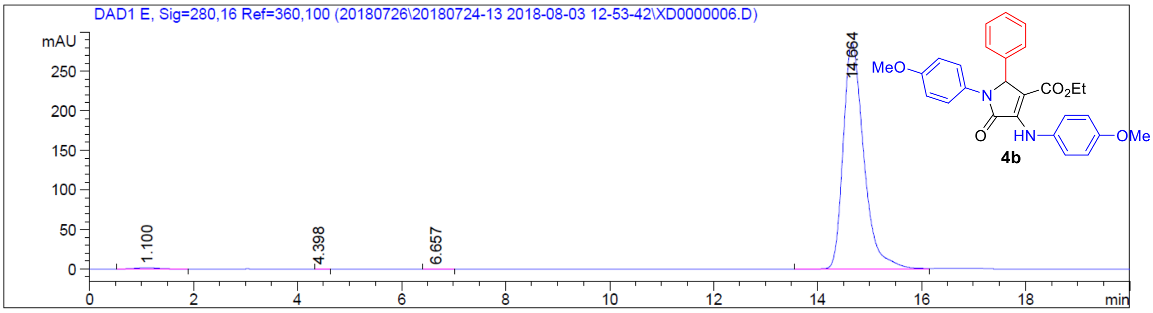

Peak #	RetTime [min]	Type	Width [min]	Area [mAU*s]	Height [mAU]	Area %
1	1.100	BB	0.4579	53.18528	1.80404	0.6679
2	4.398	VV	0.2038	5.74272	3.56738e-1	0.0721
3	6.657	BB	0.1593	1.99646	1.85901e-1	0.0251
4	14.664	VV	0.4186	7901.75244	285.79895	99.2349

*Ethyl 1-benzyl-4-(benzylamino)-5-oxo-2-phenyl-2,5-dihydro-1H-pyrrole-3-carboxylate (**4c**).*

Purity: 99.8%.

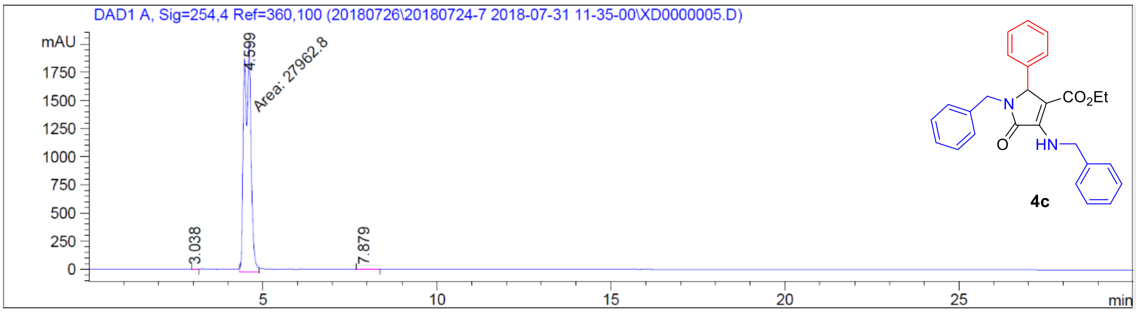

Peak #	RetTime [min]	Type	Width [min]	Area [mAU*s]	Height [mAU]	Area %
1	3.038	BV	0.1576	4.41431	3.68958e-1	0.0158
2	4.599	MM	0.2293	2.79628e4	2032.88660	99.8965
3	7.879	BB	0.1723	24.54811	2.13283	0.0877

*Isopropyl 5-oxo-2-phenyl-1-(p-tolyl)-4-(p-tolylamino)-2,5-dihydro-1H-pyrrole-3-carboxylate (**4d**).*

Purity: 98.5%.

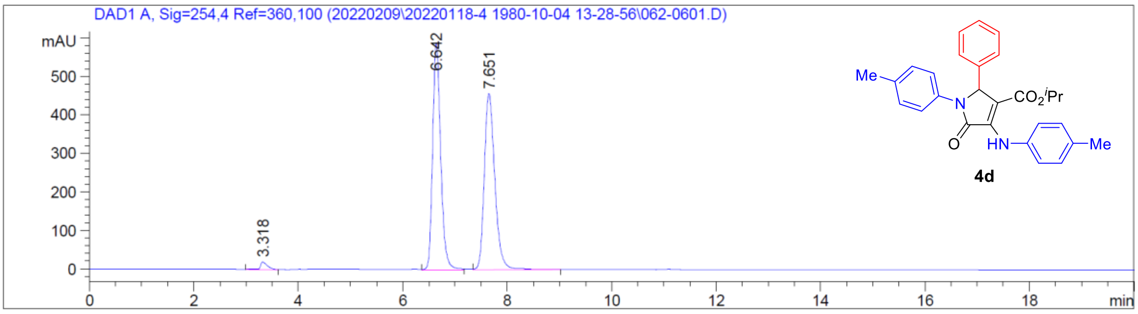

Peak #	RetTime [min]	Type	Width [min]	Area [mAU*s]	Height [mAU]	Area %
1	3.318	BV	0.1421	186.90810	19.44891	1.4491
2	6.642	VV	0.1646	6286.14111	588.47589	48.7376
3	7.651	VV	0.2158	6424.87354	457.63943	49.8132

*Methyl 5-oxo-2-phenyl-1-(p-tolyl)-4-(p-tolylamino)-2,5-dihydro-1H-pyrrole-3-carboxylate (**4e**).*

Purity: 98.0%.

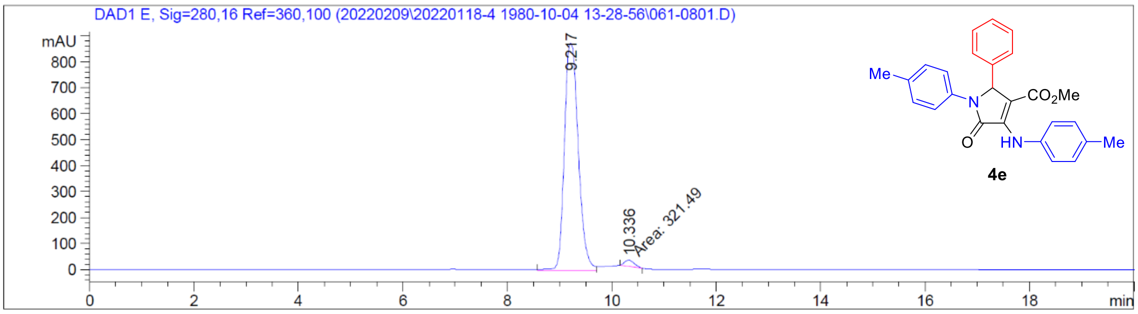

Peak #	RetTime [min]	Type	Width [min]	Area [mAU*s]	Height [mAU]	Area %
1	9.217	VV	0.2868	1.60668e4	872.68652	98.0383
2	10.336	MM	0.2181	321.48987	24.56453	1.9617

*Methyl 5-oxo-1-(p-tolyl)-4-(p-tolylamino)-2-(4-(trifluoromethyl)phenyl)-2,5-dihydro-1H-pyrrole-3-carboxylatecarboxylate (**4f**).*

Purity: 98.7%.

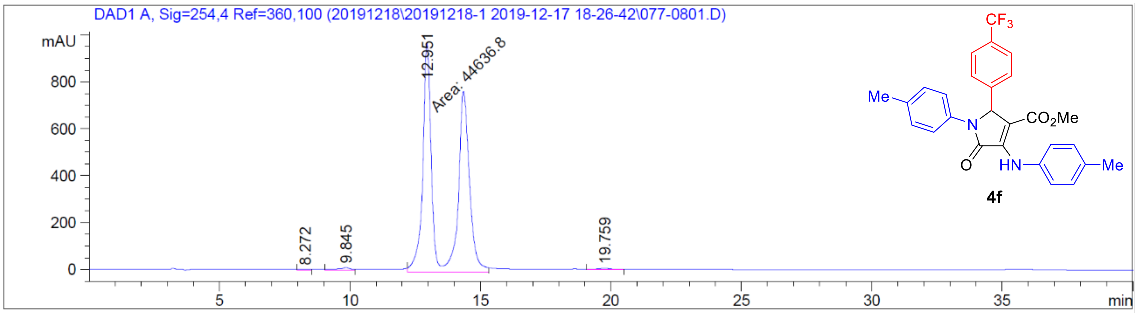

Peak #	RetTime [min]	Type	Width [min]	Area [mAU*s]	Height [mAU]	Area %
1	8.272	BV	0.4165	86.56400	2.81680	0.1915
2	9.845	VV	0.3672	303.55914	11.16373	0.6717
3	12.951	MM	0.7615	4.46368e4	976.93579	98.7702
4	19.759	VB	0.4850	165.66716	5.02045	0.3666

*Methyl 1-benzyl-4-(benzylamino)-5-oxo-2-(4-(trifluoromethyl)phenyl)-2,5-dihydro-1H-pyrrole-3-carboxylate (**4g**).*

Purity: 97.3%.

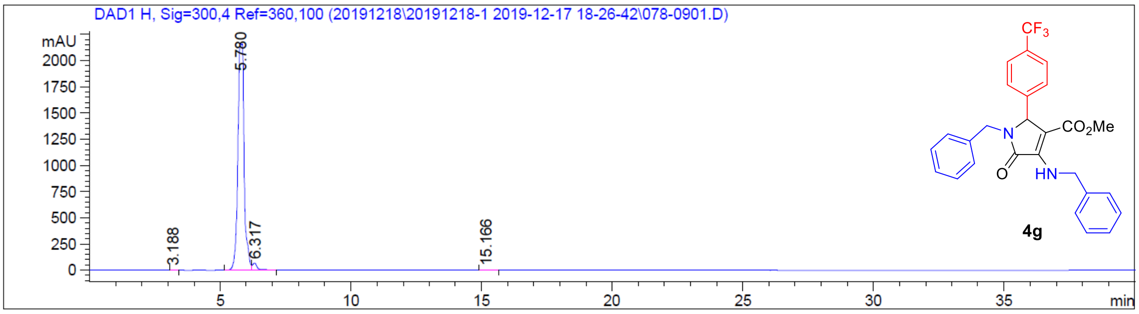

Peak #	RetTime [min]	Type	Width [min]	Area [mAU*s]	Height [mAU]	Area %
1	3.188	BV	0.1586	16.21976	1.44847	0.0437
2	5.780	VV	0.2188	3.61324e4	2164.44165	97.3409
3	6.317	VV	0.2012	967.76196	68.35409	2.6072
4	15.166	BB	0.3057	3.05224	1.54996e-1	8.223e-3

*Methyl 2-(4-hydroxyphenyl)-5-oxo-1-(p-tolyl)-4-(p-tolylamino)-2,5-dihydro-1H-pyrrole-3-carboxylate (**4h**).*

Purity: 99.1%.

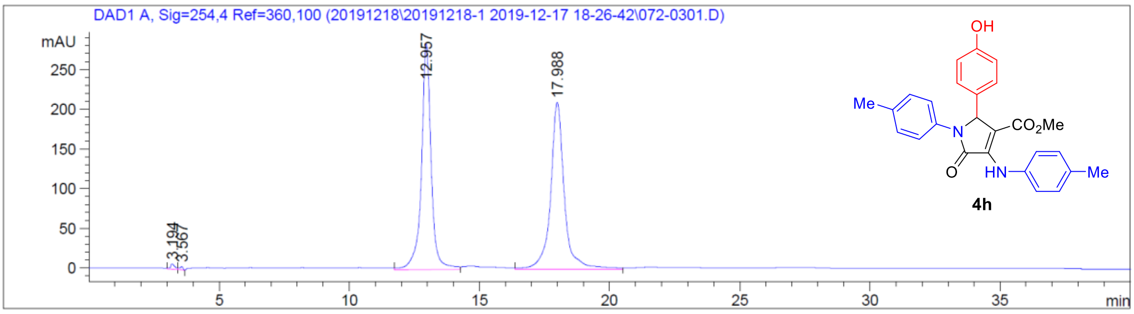

Peak #	RetTime [min]	Type	Width [min]	Area [mAU*s]	Height [mAU]	Area %
1	3.194	BV	0.1682	76.18906	6.52672	0.4947
2	3.567	VV	0.1419	39.42101	4.03697	0.2560
3	12.957	VV	0.3862	7466.75781	284.46246	48.4854
4	17.988	VB	0.5419	7817.65088	210.19771	50.7639

*Methyl 1-benzyl-4-(benzylamino)-2-(4-hydroxyphenyl)-5-oxo-2,5-dihydro-1H-pyrrole-3-carboxylate (**4i**).*
Purity: 99.6%.

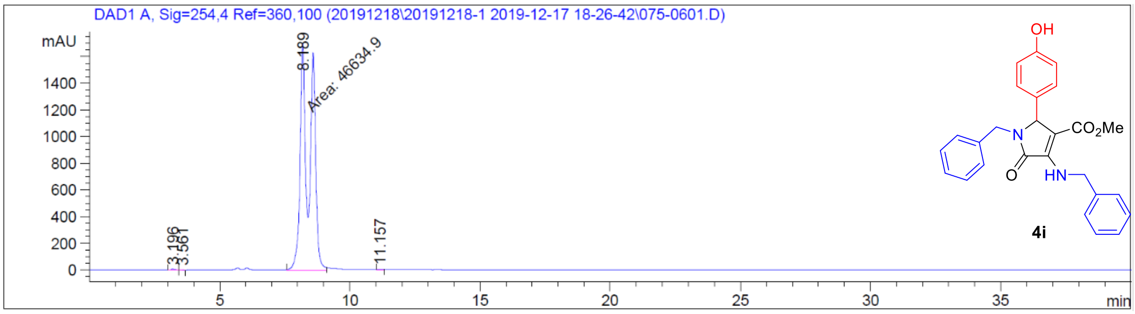

Peak #	RetTime [min]	Type	Width [min]	Area [mAU*s]	Height [mAU]	Area %
1	3.196	BV	0.1763	86.73330	7.01241	0.1854
2	3.561	VV	0.1394	35.73888	3.74411	0.0764
3	8.189	MM	0.4563	4.66349e4	1703.26184	99.6652
4	11.157	VV	0.2401	34.17477	2.03268	0.0730

*Methyl 2-(3-methoxyphenyl)-5-oxo-1-(p-tolyl)-4-(p-tolylamino)-2,5-dihydro-1H-pyrrole-3-carboxylate (**4j**).*

Purity: 97.9%.

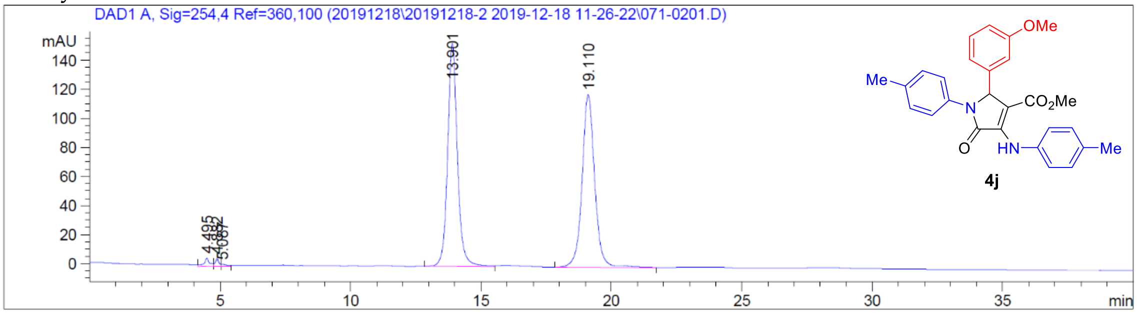

Peak #	RetTime [min]	Type	Width [min]	Area [mAU*s]	Height [mAU]	Area %
1	4.495	VV	0.2060	88.43433	5.79530	1.0598
2	4.882	VV	0.1412	53.31352	5.40036	0.6389
3	5.087	VB	0.2061	24.76991	1.56999	0.2968
4	13.901	BB	0.3966	4035.34888	153.56879	48.3596
5	19.110	VB	0.5193	4142.60303	118.66932	49.6449

*Methyl 1-benzyl-4-(benzylamino)-2-(3-methoxyphenyl)-5-oxo-2,5-dihydro-1H-pyrrole-3-carboxylate (**4k**).*

Purity: 99.5%.

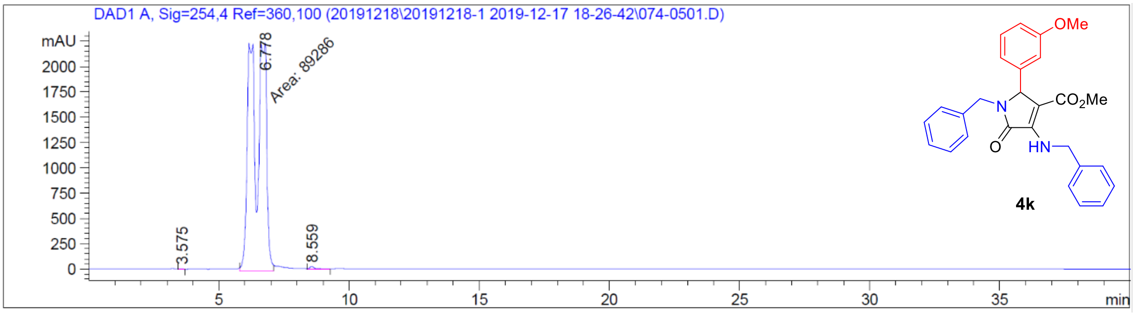

Peak #	RetTime [min]	Type	Width [min]	Area [mAU*s]	Height [mAU]	Area %
1	3.575	VV	0.1369	31.79663	3.34504	0.0354
2	6.778	MM	0.6590	8.92860e4	2257.98657	99.5165
3	8.559	VB	0.2299	401.96927	25.22869	0.4480

*Methyl 2-(4-hydroxy-3-methoxyphenyl)-5-oxo-1-(p-tolyl)-4-(p-tolylamino)-2,5-dihydro-1H-pyrrole-3-carboxylate (**4l**).*

Purity: 99.6%.

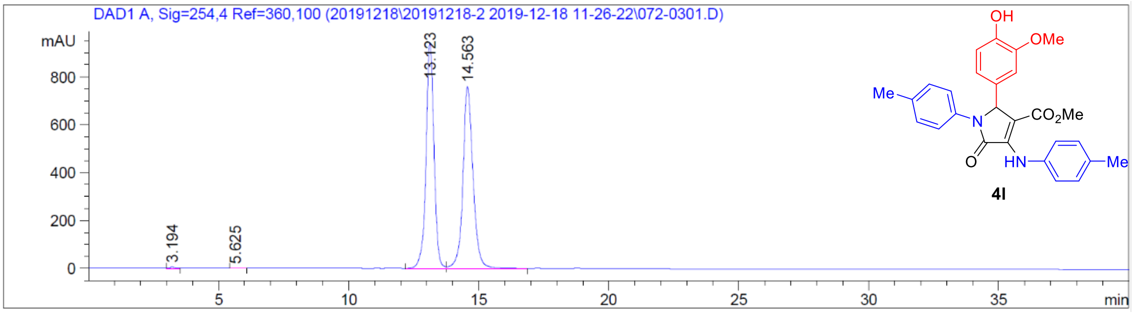

Peak #	RetTime [min]	Type	Width [min]	Area [mAU*s]	Height [mAU]	Area %
1	3.194	BV	0.1942	109.05160	7.94268	0.2680
2	5.625	BB	0.2868	6.36809	2.87126e-1	0.0157
3	13.123	VV	0.3236	1.99908e4	942.09021	49.1296
4	14.563	VB	0.4041	2.05838e4	759.78418	50.5867

*Methyl 1-benzyl-4-(benzylamino)-2-(4-hydroxy-3-methoxyphenyl)-5-oxo-2,5-dihydro-1H-pyrrole-3-carboxylate (**4m**).*

Purity: 96.1%.

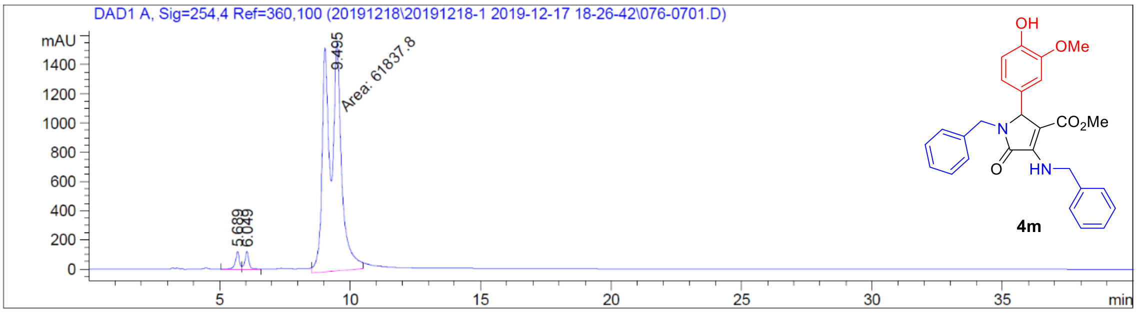

Peak #	RetTime [min]	Type	Width [min]	Area [mAU*s]	Height [mAU]	Area %
1	5.689	BV	0.1567	1289.76282	120.65879	2.0039
2	6.049	VB	0.1528	1236.02344	119.37547	1.9204
3	9.495	MM	0.6592	6.18378e4	1563.49512	96.0758

*5-oxo-2-phenyl-N,1-di-p-tolyl-4-(p-tolylamino)-2,5-dihydro-1H-pyrrole-3-carboxamide (**8a**).*

Purity: 98.8%.

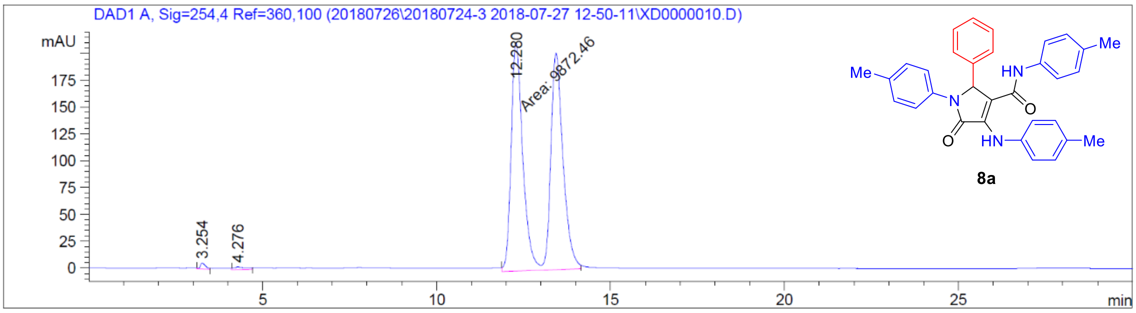

Peak #	RetTime [min]	Type	Width [min]	Area [mAU*s]	Height [mAU]	Area %
1	3.254	VV	0.1695	56.88197	5.28663	0.5695
2	4.276	VV	0.3097	59.38241	2.40771	0.5945
3	12.280	MM	0.7731	9872.45508	212.83772	98.8360

*N,1-bis(4-methoxyphenyl)-4-((4-methoxyphenyl)amino)-5-oxo-2-phenyl-2,5-dihydro-1H-pyrrole-3-carboxamide (**8b**).*

Purity: 98.9%.

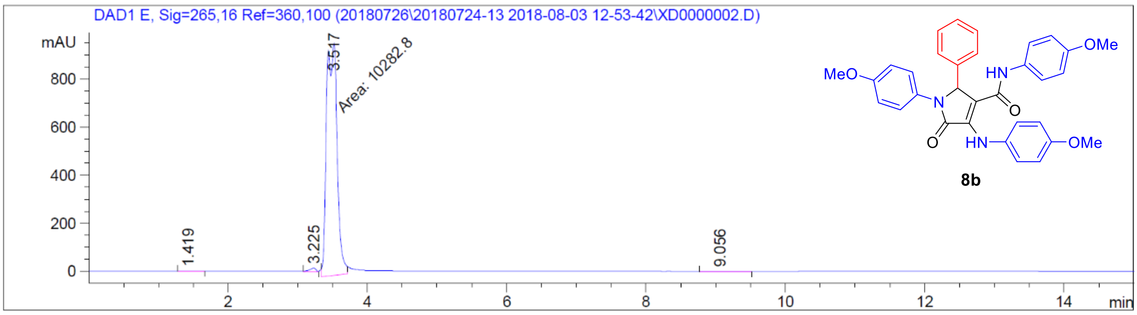

Peak #	RetTime [min]	Type	Width [min]	Area [mAU*s]	Height [mAU]	Area %
1	1.419	VV	0.2873	19.57364	9.23241e-1	0.1884
2	3.225	VV	0.0949	81.19623	13.36656	0.7817
3	3.517	MM	0.1775	1.02828e4	965.26508	98.9918
4	9.056	VV	0.3188	3.95310	1.60825e-1	0.0381

Ethyl 4-amino-1-benzyl-5-oxo-2-phenyl-2,5-dihydro-1H-pyrrole-3-carboxylate **9**.

Purity: 99.6%.

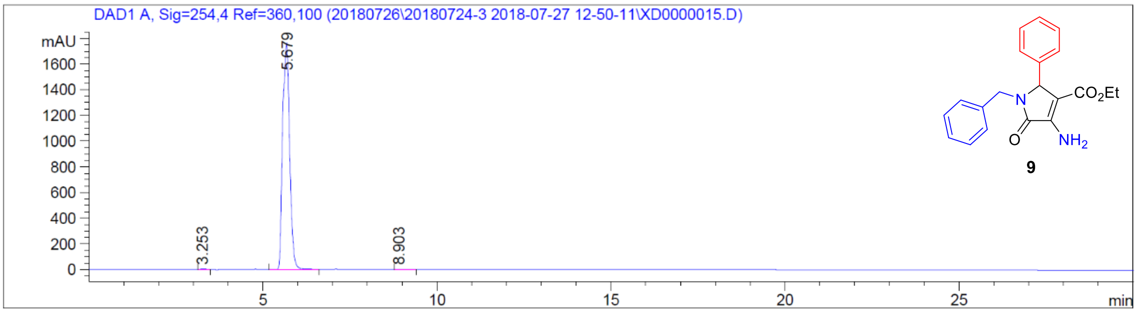

Peak #	RetTime [min]	Type	Width [min]	Area [mAU*s]	Height [mAU]	Area %
1	3.253	BV	0.1621	66.79533	6.59757	0.2664
2	5.679	VV	0.1951	2.49947e4	1766.63623	99.6860
3	8.903	VB	0.3524	11.93234	4.60309e-1	0.0476

Diethyl (4-hydroxy-5-oxo-2-phenyl-1-(p-tolyl)-2,5-dihydro-1H-pyrrol-3-yl)phosphonate (***12a***).

Purity: 98.9%.

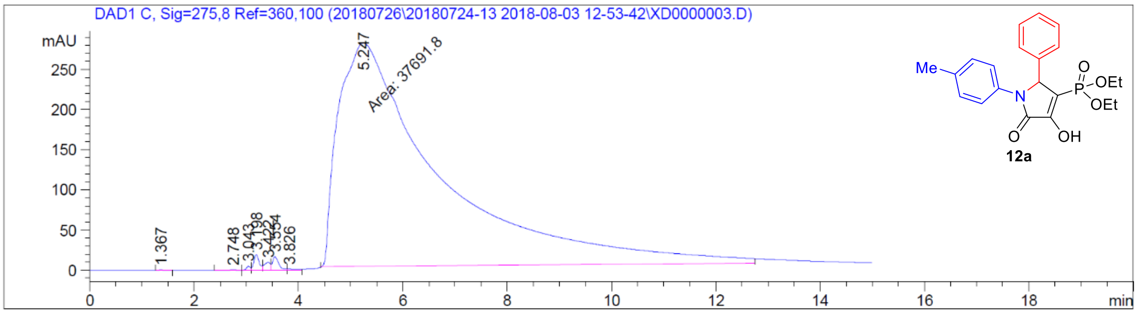

Peak #	RetTime [min]	Type	Width [min]	Area [mAU*s]	Height [mAU]	Area %
1	1.367	BB	0.1410	1.39523	1.41597e-1	3.662e-3
2	2.748	VV	0.2634	2.80496	1.43924e-1	7.363e-3
3	3.043	VV	0.0732	23.83507	5.03325	0.0626
4	3.198	VV	0.1097	138.10333	19.23956	0.3625
5	3.422	VV	0.1256	80.38291	9.80991	0.2110
6	3.554	VV	0.1274	139.91254	16.75740	0.3673
7	3.826	VV	0.1738	18.70990	1.38340	0.0491
8	5.247	MM	2.2598	3.76918e4	277.98685	98.9365

Diethyl (4-hydroxy-1-(4-methoxyphenyl)-5-oxo-2-phenyl-2,5-dihydro-1H-pyrrol-3-yl)phosphonate (***12b***).

Purity: 99.8%.

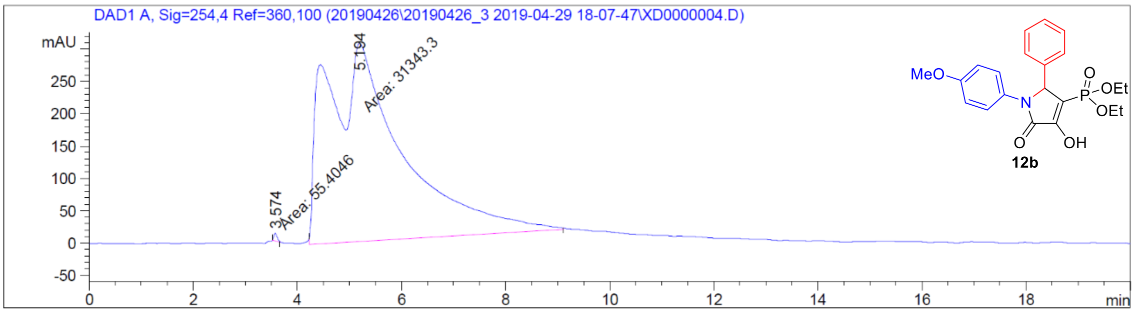

Peak #	RetTime [min]	Type	Width [min]	Area [mAU*s]	Height [mAU]	Area %
1	3.574	MM	0.0752	55.40464	12.27632	0.1765
2	5.194	MM	1.7053	3.13433e4	306.32495	99.8235

*Diethyl (1-(2-fluorophenyl)-4-hydroxy-5-oxo-2-phenyl-2,5-dihydro-1H-pyrrol-3-yl)phosphonate (**12c**).*

Purity: 98.8%.

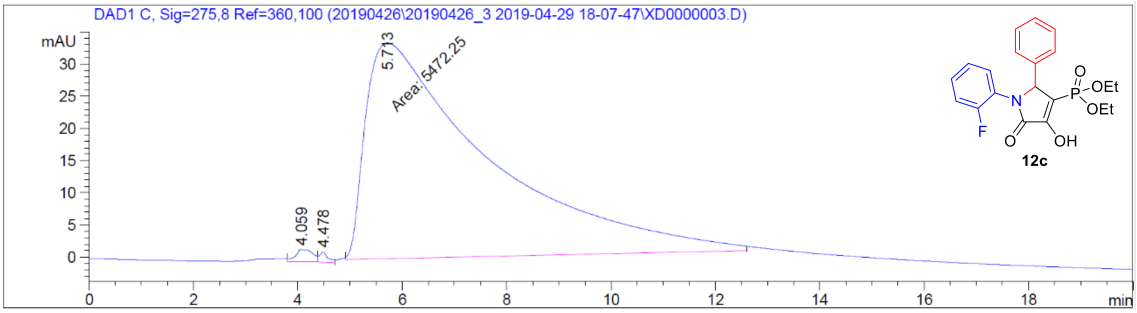

Peak #	RetTime [min]	Type	Width [min]	Area [mAU*s]	Height [mAU]	Area %
1	4.059	VV	0.3023	45.59560	1.91129	0.8238
2	4.478	VV	0.1508	17.12268	1.60044	0.3094
3	5.713	MM	2.7221	5472.25391	33.50546	98.8669

*Diethyl (4-hydroxy-2-(4-nitrophenyl)-5-oxo-1-(p-tolyl)-2,5-dihydro-1H-pyrrol-3-yl)phosphonate (**12d**).*

Purity: 99.8%.

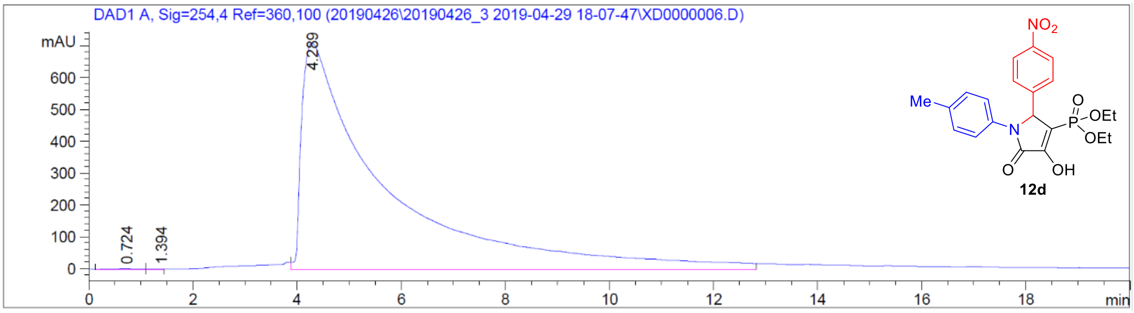

Peak #	RetTime [min]	Type	Width [min]	Area [mAU*s]	Height [mAU]	Area %
1	0.724	BV	0.3866	64.83792	2.10954	0.0821
2	1.394	VV	0.1683	14.24921	1.09146	0.0180
3	4.289	VV	1.4432	7.89353e4	711.03595	99.8999

*Diethyl (2-(4-fluorophenyl)-4-hydroxy-5-oxo-1-(p-tolyl)-2,5-dihydro-1H-pyrrol-3-yl)phosphonate (**12e**).*

Purity: 99.8%.

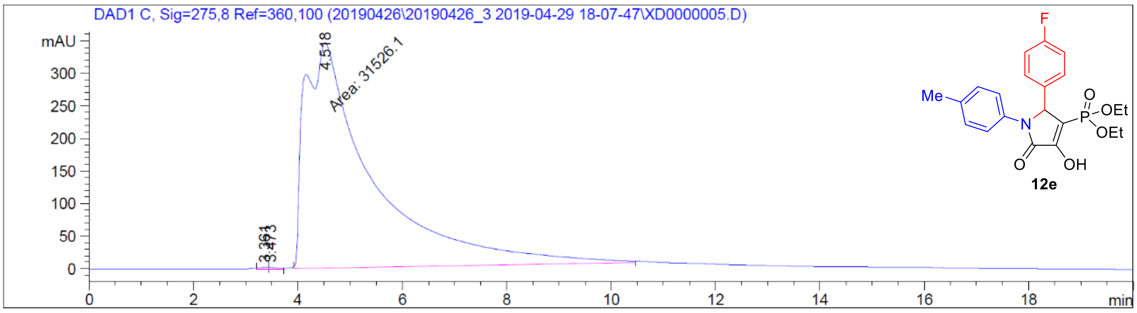

Peak #	RetTime [min]	Type	Width [min]	Area [mAU*s]	Height [mAU]	Area %
1	3.361	VV	0.1494	35.38084	3.29164	0.1120
2	3.473	VV	0.1507	34.84195	3.01838	0.1103
3	4.518	MM	1.5187	3.15261e4	345.97363	99.7778

*Diethyl (4-hydroxy-5-oxo-2-(thiophen-2-yl)-1-(p-tolyl)-2,5-dihydro-1H-pyrrol-3-yl)phosphonate (**12f**).*

Purity: 99.8%.

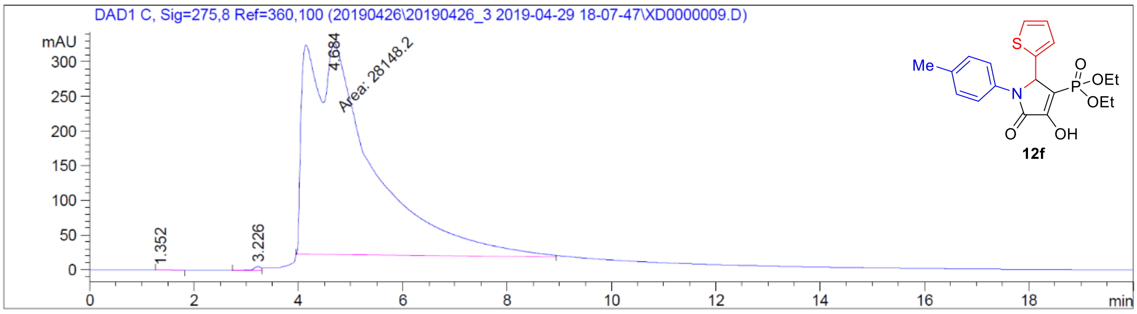

Peak #	RetTime [min]	Type	Width [min]	Area [mAU*s]	Height [mAU]	Area %
1	1.352	BB	0.0914	3.53416	5.77341e-1	0.0125
2	3.226	VV	0.1344	46.79567	5.43802	0.1660
3	4.684	MM	1.5370	2.81482e4	305.22226	99.8215

*Ethyl 3-(diethoxyphosphoryl)-4-hydroxy-5-oxo-1-(p-tolyl)-2,5-dihydro-1H-pyrrole-2-carboxylate (**12g**).*

Purity: 99.5%.

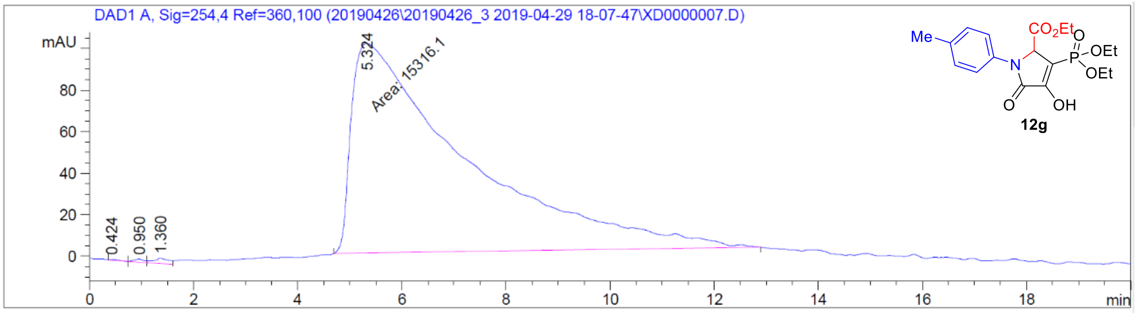

Peak #	RetTime [min]	Type	Width [min]	Area [mAU*s]	Height [mAU]	Area %
1	0.424	VV	0.1908	5.54936	3.58149e-1	0.0361
2	0.950	VV	0.1942	19.33325	1.42585	0.1256
3	1.360	VV	0.2400	48.92009	2.61863	0.3179
4	5.324	MM	2.5311	1.53161e4	100.85185	99.5204

*Diethyl (4-hydroxy-2-isopropyl-5-oxo-1-(p-tolyl)-2,5-dihydro-1H-pyrrol-3-yl)phosphonate (**12h**).*

Purity: 99.3%.

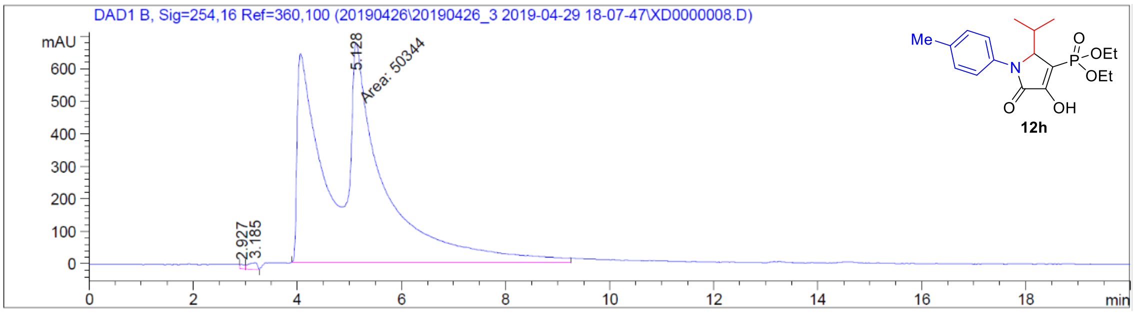

Peak #	RetTime [min]	Type	Width [min]	Area [mAU*s]	Height [mAU]	Area %
1	2.927	VV	0.0963	87.27385	13.34818	0.1723
2	3.185	VV	0.1709	234.29451	19.40592	0.4624
3	5.128	MM	1.2445	5.03440e4	674.23309	99.3653

*Diisopropyl (4-hydroxy-5-oxo-2-phenyl-1-(p-tolyl)-2,5-dihydro-1H-pyrrol-3-yl)phosphonate (**12i**).*

Purity: 98.7%.

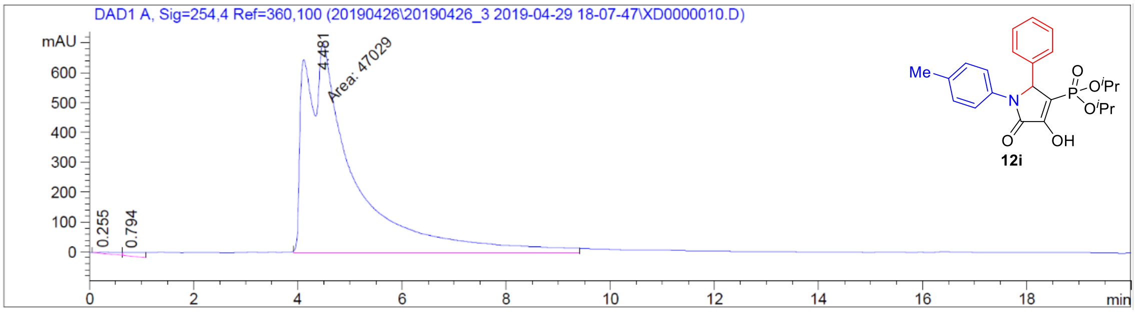

Peak #	RetTime [min]	Type	Width [min]	Area [mAU*s]	Height [mAU]	Area %
1	0.255	BV	0.5083	200.12527	4.98580	0.4204
2	0.794	VV	0.3707	373.67450	13.18335	0.7850
3	4.481	MM	1.1138	4.70290e4	703.75299	98.7946

*Diisopropyl (2-(4-fluorophenyl)-4-hydroxy-5-oxo-1-(p-tolyl)-2,5-dihydro-1H-pyrrol-3-yl)phosphonatephosphonate (**12j**).*

Purity: 99.9%.

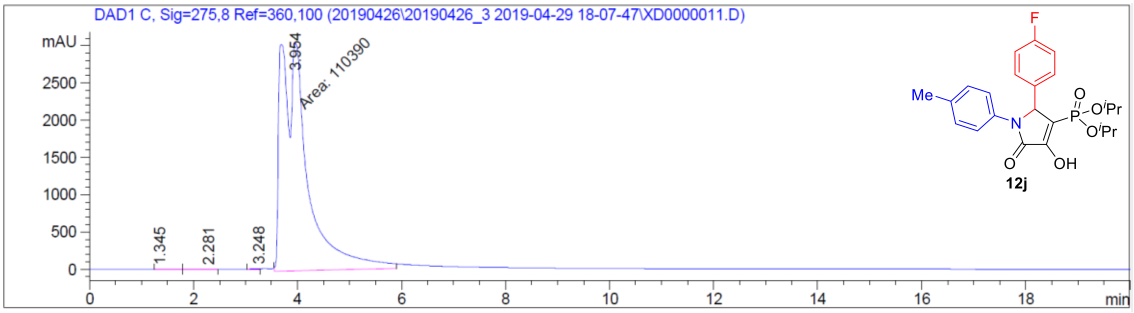

Peak #	RetTime [min]	Type	Width [min]	Area [mAU*s]	Height [mAU]	Area %
1	1.345	BV	0.1001	4.23250	6.16152e-1	3.832e-3
2	2.281	VV	0.1630	3.62124	3.08345e-1	3.279e-3
3	3.248	VV	0.1116	49.06279	5.98652	0.0444
4	3.954	MM	0.6033	1.10390e5	3049.63867	99.9485

*Ethyl 3-(diisopropoxyphosphoryl)-4-hydroxy-5-oxo-1-(p-tolyl)-2,5-dihydro-1H-pyrrole-2-carboxylate (**12k**).*

Purity: 98.5%.

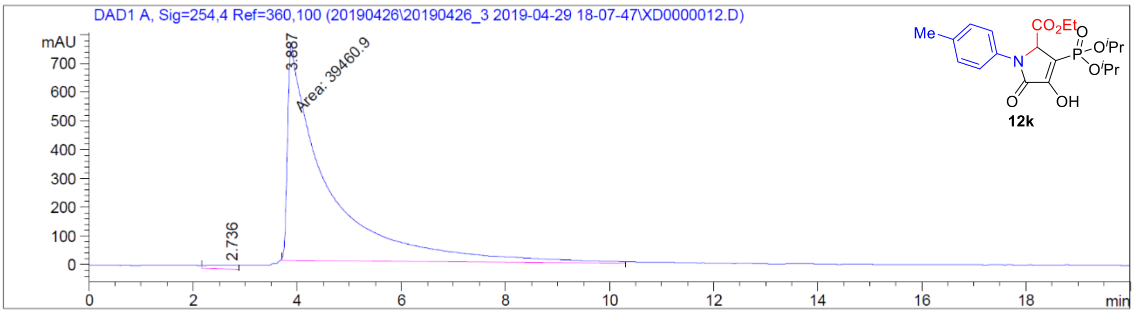

Peak #	RetTime [min]	Type	Width [min]	Area [mAU*s]	Height [mAU]	Area %
1	2.736	VV	0.4601	560.34741	15.06060	1.4001
2	3.887	MM	0.8726	3.94609e4	753.68414	98.5999

4-(diphenylphosphoryl)-3-hydroxy-5-phenyl-1-(p-tolyl)-1,5-dihydro-2H-pyrrol-2-onephosphonate (***12l***).

Purity: 99.4%.

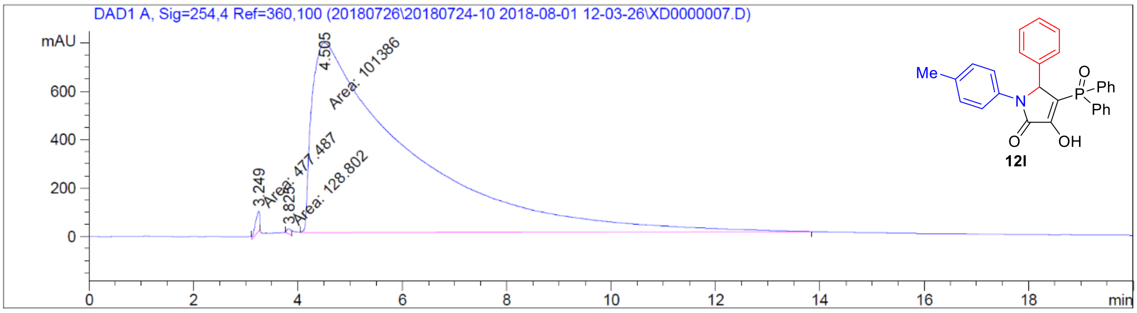

Peak #	RetTime [min]	Type	Width [min]	Area [mAU*s]	Height [mAU]	Area %
1	3.249	MM	0.0956	477.48669	83.28705	0.4682
2	3.825	MM	0.1030	128.80191	20.84165	0.1263
3	4.505	MM	2.1535	1.01386e5	784.65271	99.4056

4-(diphenylphosphoryl)-1-(2-fluorophenyl)-3-hydroxy-5-phenyl-1,5-dihydro-2H-pyrrol-2-one (***12n***).

Purity: 98.9%.

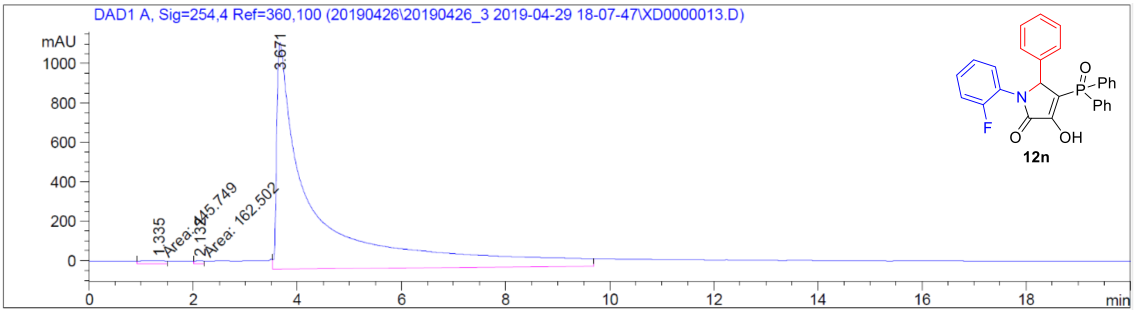

Peak #	RetTime [min]	Type	Width [min]	Area [mAU*s]	Height [mAU]	Area %
1	1.335	MM	0.5523	445.74890	13.45080	0.7702
2	2.132	MM	0.1965	162.50189	13.78104	0.2808
3	3.671	VV	0.6242	5.72681e4	1147.54175	98.9491

4-(diphenylphosphoryl)-5-(4-fluorophenyl)-3-hydroxy-1-(p-tolyl)-1,5-dihydro-2H-pyrrol-2-one (***12o***).

Purity: 99.3%.

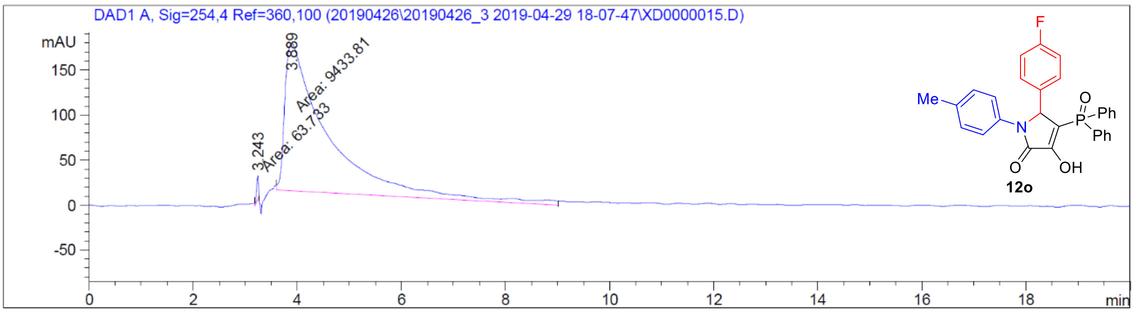

Peak #	RetTime [min]	Type	Width [min]	Area [mAU*s]	Height [mAU]	Area %
1	3.243	MM	0.0401	63.73304	26.46947	0.6710
2	3.889	MM	0.9605	9433.81055	163.69989	99.3290

4-(diphenylphosphoryl)-3-hydroxy-5-(perfluorophenyl)-1-(p-tolyl)-1,5-dihydro-2H-pyrrol-2-one (***12p***).

Purity: 99.7%.

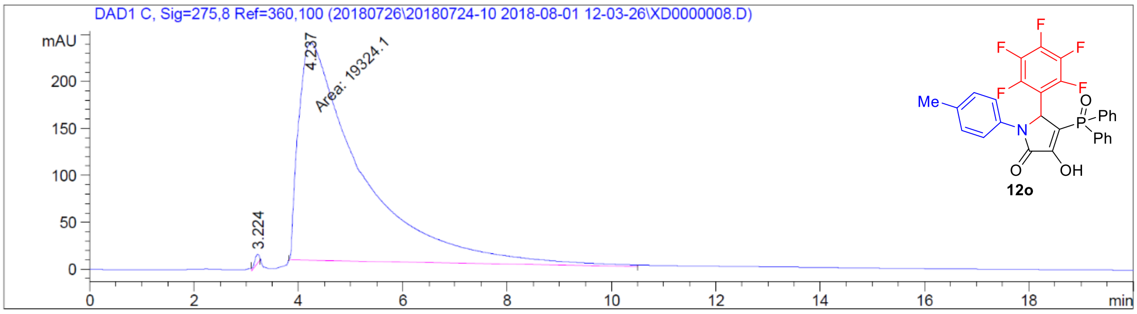

Peak #	RetTime [min]	Type	Width [min]	Area [mAU*s]	Height [mAU]	Area %
1	3.224	MM	0.1010	56.92308	9.39428	0.2937
2	4.237	MM	1.3892	1.93241e4	231.83881	99.7063

Ethyl 4-hydroxy-5-oxo-2-phenyl-1-(p-tolyl)-2,5-dihydro-1H-pyrrole-3-carboxylate (***13a***).

Purity: 99.6%.

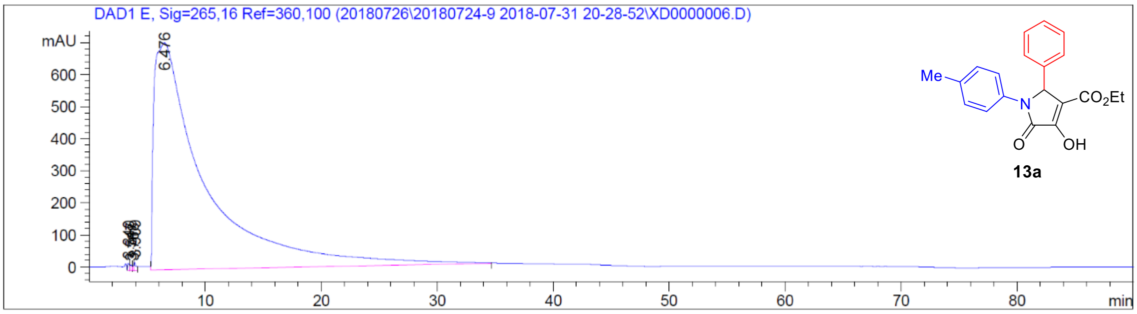

Peak #	RetTime [min]	Type	Width [min]	Area [mAU*s]	Height [mAU]	Area %
1	3.340	VV	0.1511	238.70406	21.91062	0.1094
2	3.547	VV	0.1427	174.84656	15.85531	0.0801
3	3.768	VV	0.0956	98.30384	14.39922	0.0450
4	3.909	VV	0.1649	299.03857	24.07861	0.1370
5	6.476	VB	3.8806	2.17440e5	706.90686	99.6285

Ethyl 4-hydroxy-1-(4-methoxyphenyl)-5-oxo-2-phenyl-2,5-dihydro-1H-pyrrole-3-carboxylate (***13b***).

Purity: 99.6%.

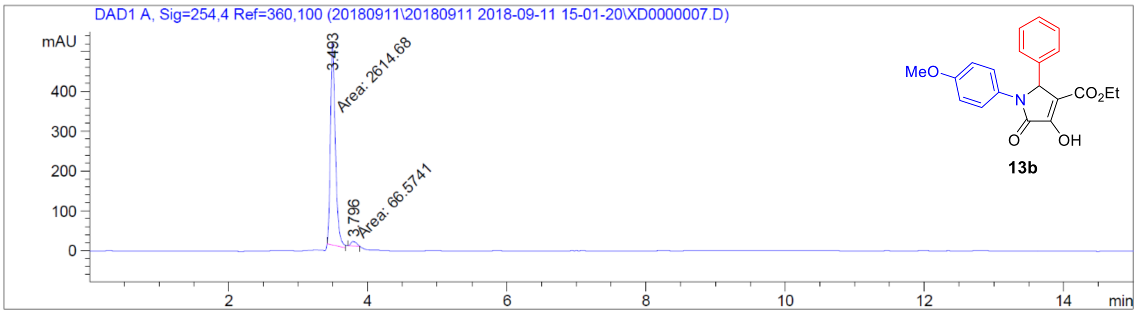

Peak #	RetTime [min]	Type	Width [min]	Area [mAU*s]	Height [mAU]	Area %
1	3.493	MM	0.0856	2614.67969	509.27289	97.5171
2	3.796	MM	0.1006	66.57410	11.02417	2.4829

Ethyl 1-benzyl-4-hydroxy-5-oxo-2-phenyl-2,5-dihydro-1H-pyrrole-3-carboxylate (***13c***).

Purity: 99.7%.

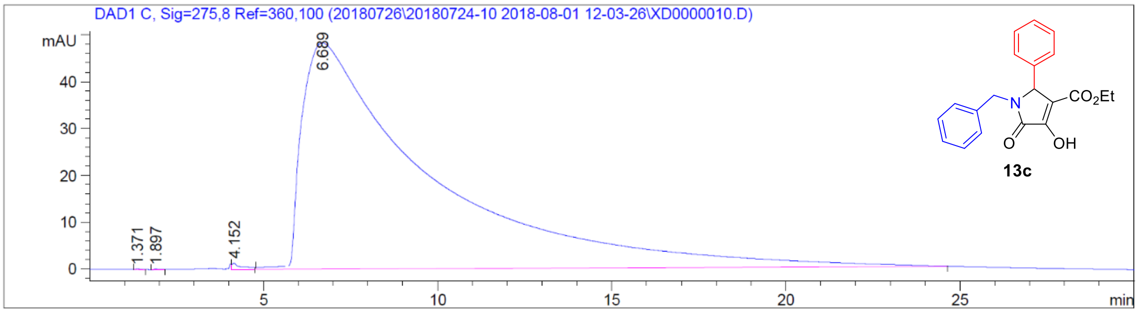

Peak #	RetTime [min]	Type	Width [min]	Area [mAU*s]	Height [mAU]	Area %
1	1.371	BB	0.1226	1.43211	1.72886e-1	0.0116
2	1.897	BB	0.1385	1.45628	1.43417e-1	0.0118
3	4.152	VB	0.2377	25.46545	1.36442	0.2060
4	6.689	BB	3.3625	1.23325e4	48.24081	99.7706

Methyl 1-benzyl-4-hydroxy-5-oxo-2-(4-(trifluoromethyl)phenyl)-2,5-dihydro-1H-pyrrole-3-carboxylate (***13d***).

Purity: 98.8%.

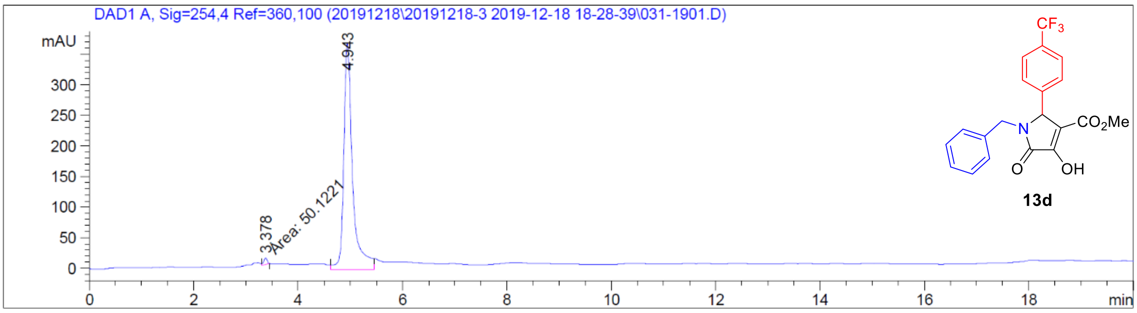

Peak #	RetTime [min]	Type	Width [min]	Area [mAU*s]	Height [mAU]	Area %
1	3.378	MM	0.0774	50.12209	10.79185	1.1405
2	4.943	VV	0.1728	4344.70361	370.60904	98.8595

Methyl 4-hydroxy-2-(3-methoxyphenyl)-5-oxo-1-(p-tolyl)-2,5-dihydro-1H-pyrrole-3-carboxylate (***13e***).

Purity: 99.5%.

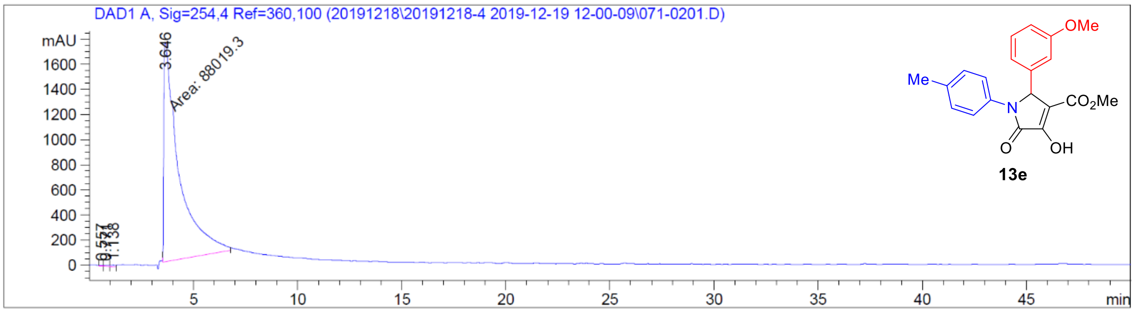

Peak #	RetTime [min]	Type	Width [min]	Area [mAU*s]	Height [mAU]	Area %
1	0.557	VV	0.1912	65.08168	5.02184	0.0736
2	0.771	VV	0.2482	132.48325	7.01853	0.1499
3	1.138	VV	0.2230	177.20050	10.71431	0.2005
4	3.646	MM	0.8402	8.80192e4	1745.97107	99.5760

*Methyl 1-benzyl-4-hydroxy-2-(3-methoxyphenyl)-5-oxo-2,5-dihydro-1H-pyrrole-3-carboxylate (**13f**).*

Purity: 99.5%.

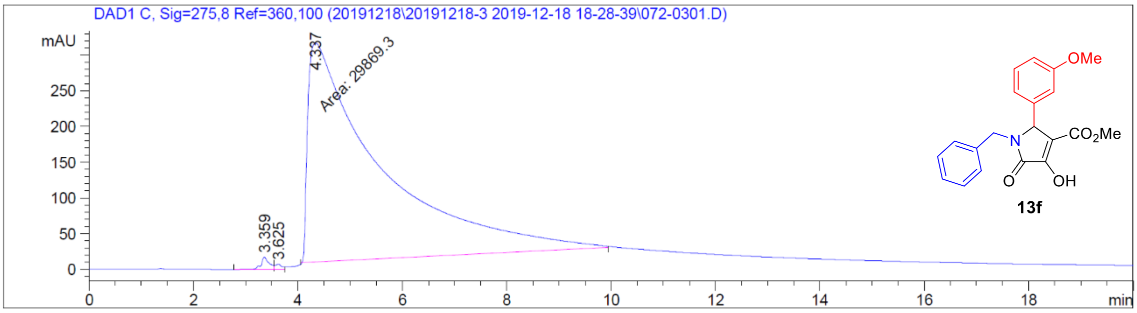

Peak #	RetTime [min]	Type	Width [min]	Area [mAU*s]	Height [mAU]	Area %
1	3.359	BV	0.1521	197.03485	17.66278	0.6538
2	3.625	VV	0.1269	68.24085	7.58840	0.2265
3	4.337	MM	1.6210	2.98693e4	307.11392	99.1197

*Methyl 4-hydroxy-2-(4-hydroxy-3-methoxyphenyl)-5-oxo-1-(p-tolyl)-2,5-dihydro-1H-pyrrole-3-carboxylate (**13g**).*

Purity: 99.1%.

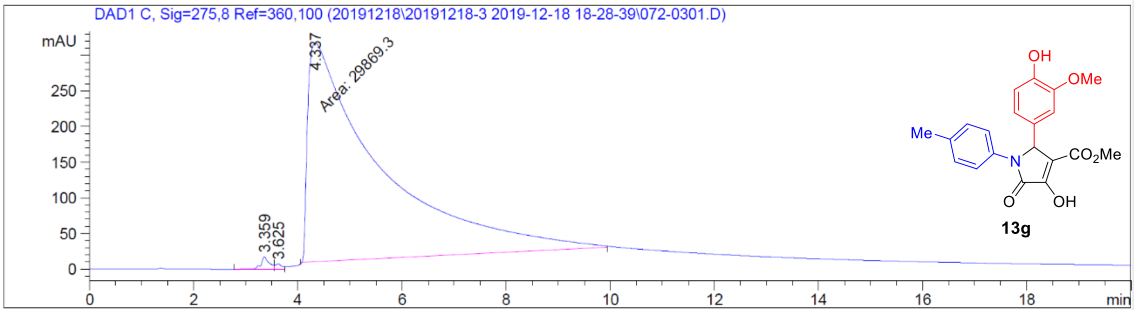

Peak #	RetTime [min]	Type	Width [min]	Area [mAU*s]	Height [mAU]	Area %
1	3.359	BV	0.1521	197.03485	17.66278	0.6538
2	3.625	VV	0.1269	68.24085	7.58840	0.2265
3	4.337	MM	1.6210	2.98693e4	307.11392	99.1197

4. Flow cytometric assays on A-549 cells after addition of compounds 4l and 12a.

Non-treated cells. FL1 negative, FL3 negative.

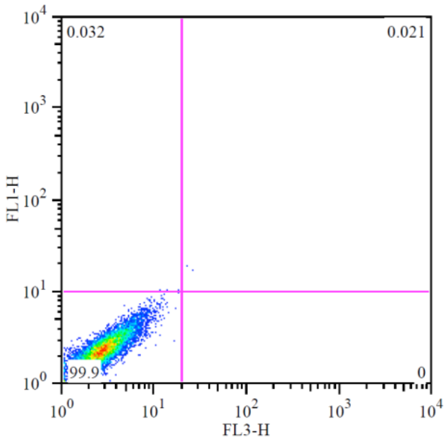

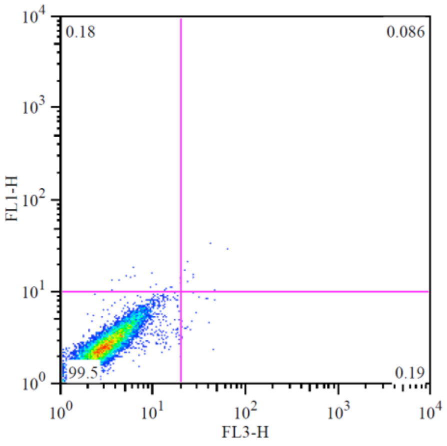

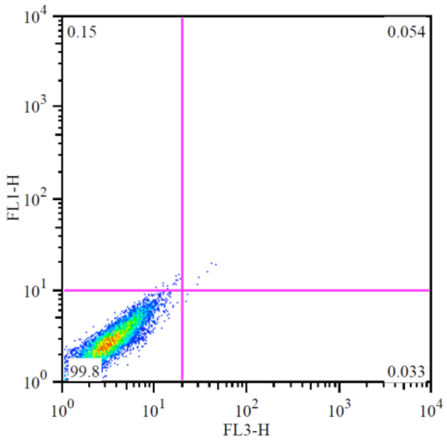

Necrotic cells, treated with Ethanol: FL1 positive, FL3 negative.

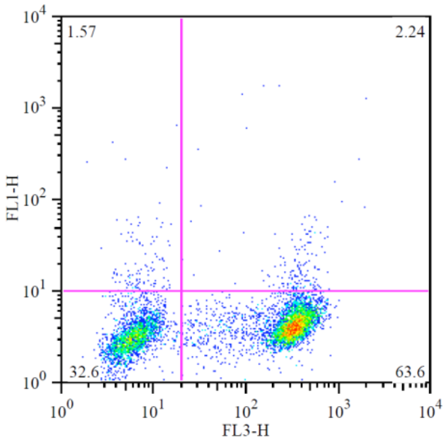

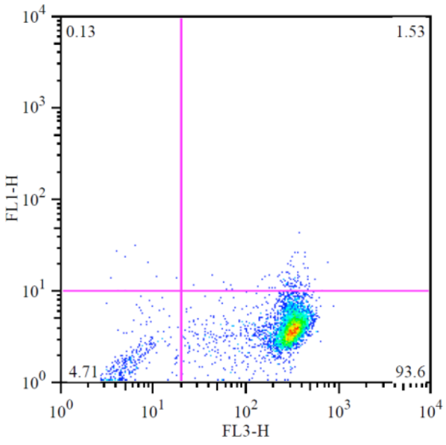

Early apoptotic cells, treated with 1 μM of Camptothecin. FL1 negative, FL3 positive.

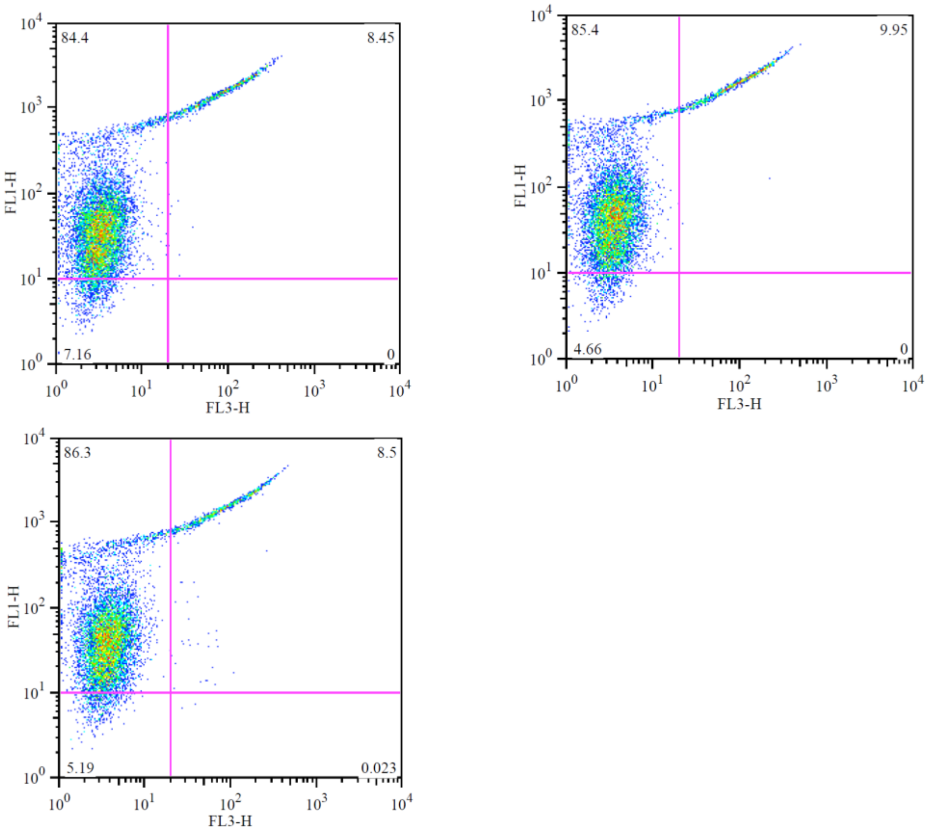

Apoptosis detection, cells treated with 1 μM of 4l for 24h.

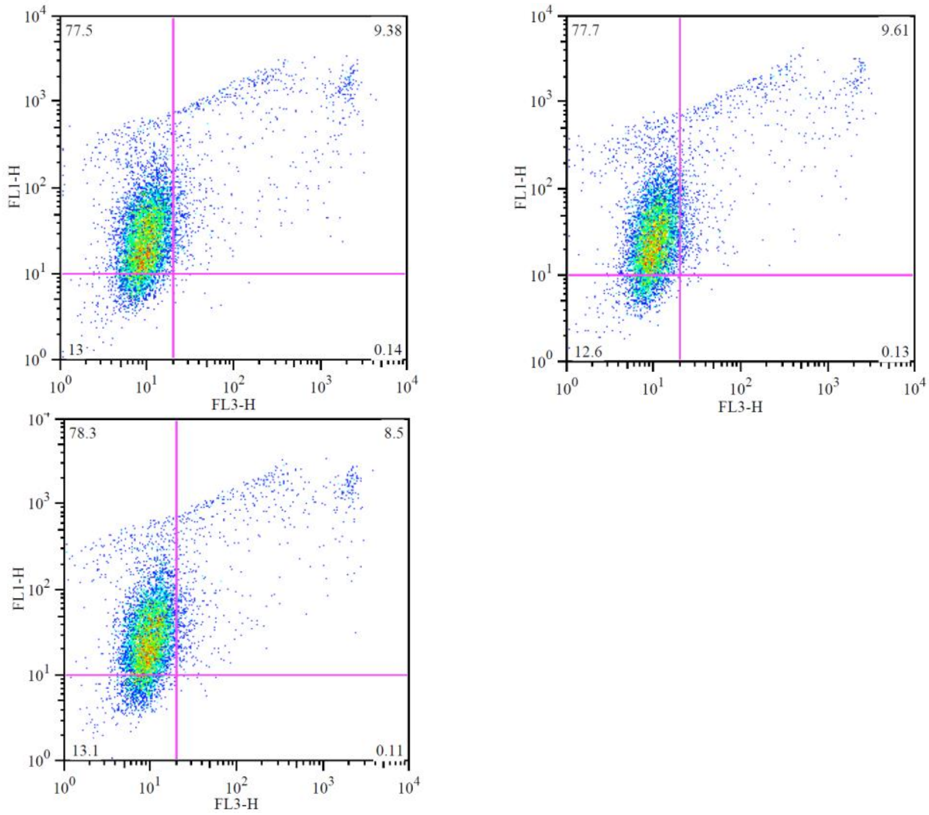

Apoptosis detection, cells treated with 5 μM of 12a for 24h.

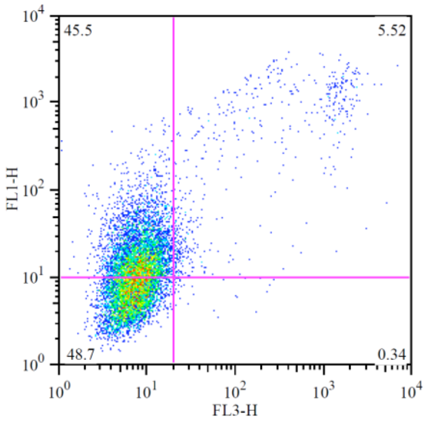

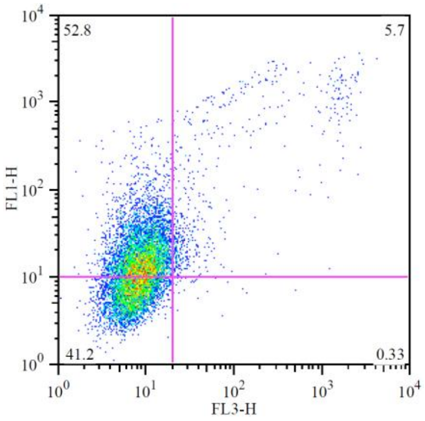

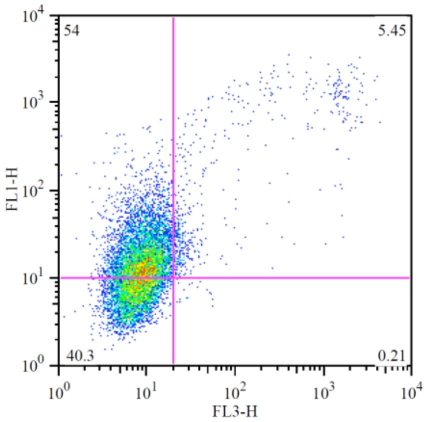

5. Antiproliferative activity in RKO cell line.

Table S1.

**Entry**	**Cmpd.**	**IC_{50}(μM)**
		**RKO (colon)**
1	**4a**	>50
2	**4b**	>50
3	**4c**	>50
4	**4k**	>50
5	**8a**	>50
6	**13a**	>50
7	**13b**	>50
8	**13c**	>50
9	**12b**	>50
10	**12d**	>50
11	**12f**	>50
12	**12i**	33.62 ± 0,41
13	**12l**	>50
14	**12m**	>50
15	**12p**	>50
17	Doxorubicin	<0.1

6. Calculation of Lipinski's rule of five and prediction of ADME properties.

In order to determine the the medicinal chemistry friendliness of compounds compounds **4**, **8**, **9**, **12** and **13,**some calculations had been made to predict ADME parameter; the results are shown in the following table. Lipinski rules evaluate if a compound is likely to be an orally active drug in humans, and according to these rules, an orally active drug has no more than one violation of the following criteria: MW ≤ 500, LogP ≤ 4.15, N or O atoms ≤ 10 and NH or OH atoms ≤ 5.[3] These calculations were performed using *SwissADME* program from the Swiss Institute of Bioinformatics (http://www.swissadme.ch/).

Table S2.

Cpd.	MW (g/mol)	LogP	HBD	HBA	RB	TPSA (Å^2)	Lipinski (Violations)	GI Abs.	BBB Permeation
**4a**	426.51	4.60	1	3	7	58.64	Yes (0)	High	Yes
**4b**	458.51	3.95	1	5	9	77.10	Yes (0)	High	Yes
**4c**	426.51	4.05	1	3	9	58.64	Yes (0)	High	Yes
**4d**	440.53	4.88	1	3	7	58.64	Yes (0)	High	Yes
**4e**	412.48	4.31	1	3	6	58.64	Yes (0)	High	Yes
**4f**	480.48	5.35	1	6	7	58.64	Yes (1)	High	No
**4g**	480.48	4.73	1	6	9	58.64	Yes (0)	High	Yes
**4h**	428.48	3.91	2	4	6	78.87	Yes (0)	High	No
**4i**	428.48	3.34	2	4	8	78.87	Yes (0)	High	No
**4j**	442.51	4.29	1	4	7	67.87	Yes (0)	High	Yes
**4k**	442.51	3.70	1	4	9	67.87	Yes (0)	High	Yes
**4l**	458.51	3.95	2	5	7	88.10	Yes (0)	High	No
**4m**	458.51	3.37	2	5	9	88.10	Yes (0)	High	No
**8a**	487.59	5.29	2	2	7	61.44	Yes (1)	High	No
**8b**	535.59	4.38	2	5	10	89.13	Yes (1)	High	No
**9**	336.38	2.35	1	3	6	72.63	Yes (0)	High	Yes
**12a**	401.39	3.08	1	5	7	85.88	Yes (0)	High	No
**12b**	417.39	2.76	1	6	8	95.11	Yes (0)	High	No
**12c**	405.36	3.02	1	6	7	85.88	Yes (0)	High	No
**12d**	446.39	2.39	1	7	8	131.70	Yes (0)	Low	No
**12e**	419.38	3.39	1	6	7	85.88	Yes (0)	High	No
**12f**	407.42	3.07	1	5	7	114.12	Yes (0)	High	No
**12g**	397.36	2.05	1	7	9	112.18	Yes (0)	High	No
**12h**	367.38	2.79	1	5	7	85.88	Yes (0)	High	No
**12i**	429.45	3.71	1	5	7	85.88	Yes (0)	High	No
**12j**	447.44	4.02	1	6	7	85.88	Yes (0)	High	No
**12k**	425.41	2.73	1	7	9	112.18	Yes (0)	High	No
**12l**	465.48	4.59	1	3	5	67.42	Yes (0)	High	No
**12m**	481.48	4.23	1	4	6	76.65	Yes (0)	High	No
**12n**	469.44	4.51	1	4	5	67.42	Yes (0)	High	No
**12o**	483.47	4.86	1	4	5	67.42	Yes (0)	High	No
**12p**	555.43	6.07	1	8	5	67.42	No (2)	Low	No
**13a**	337.37	2.98	1	4	5	66.84	Yes (0)	High	Yes

[3] Lipinski, C.A.; Lombardo, F.; Dominy, B. W.; Feeney, P. J. Experimental and computational approaches to estimate solubility and permeability in drug discovery and development settings. *Adv. Drug. Deliv. Rev.* **2001**, *64*, 4-17. DOI: 10.1016/s0169-409x(00)00129-0.

**13b**	353.37	2.56	1	5	6	76.07	Yes (0)	High	Yes
**13c**	337.37	2.64	1	4	6	66.84	Yes (0)	High	Yes
**13d**	391.34	3.35	1	7	6	66.84	Yes (0)	High	Yes
**13e**	353.37	2.67	1	5	5	76.07	Yes (0)	High	Yes
**13f**	353.37	2.34	1	5	6	76.07	Yes (0)	High	Yes
**13g**	369.37	2.25	2	6	5	96.30	Yes (0)	High	No

*LogP= Partition Coefficient, Lipophilicity.; HBD= Num. H-bond donors.; HBA= Num. H-bond acceptors; RB= Num. rotatable bonds.; GI Abs= Gastro-Intestinal Absortion.; BBB Permeation= Blood-Brain-Barrier Permeation.
